# Psoriasis and gut microbiota: A Mendelian randomization study

**DOI:** 10.1111/jcmm.18023

**Published:** 2023-12-25

**Authors:** Ruonan Wu, Li Zhao, Zewen Wu, Xinling Liu, Jingxuan Li, Tong Wang, Liyun Zhang

**Affiliations:** ^1^ School of Public Health Shanxi Medical University Taiyuan China; ^2^ School of Pharmacy Shanxi Medical University Taiyuan China; ^3^ Shanxi Bethune Hospital, Shanxi Academy of Medical Sciences, Tongji Shanxi Hospital Third Hospital of Shanxi Medical University Taiyuan China; ^4^ Tongji Hospital, Tongji Medical College Huazhong University of Science and Technology Wuhan China; ^5^ Third Clinical College Shanxi University of Chinese Medicine Jinzhong China; ^6^ Department of Health Statistics and Epidemiology, School of Public Health Shanxi Medical University Taiyuan China

**Keywords:** gut microbiota, GWAS, mendelian randomization, psoriasis

## Abstract

In recent years, an increasing number of observational studies have revealed an association between gut microbiota composition and psoriasis patients. However, whether this association reflects a causal relationship remains unclear. This study aimed to identify the causal relationship between gut microbiota and psoriasis through relevant research. In order to determine whether gut microbiota and psoriasis are causally related, we conducted a Mendelian randomization analysis using summary statistics from genome‐wide association studies (GWAS). As the exposure factor, we used summary statistics data from a GWAS study conducted by the MiBioGen Consortium, including 18,340 individuals with whole‐genome gut microbiota composition, and data from the FinnGen GWAS study on psoriasis, including 9267 patients and 364,071 controls as the disease outcome. Two‐sample Mendelian randomization analysis was subsequently performed with inverse variance weighted, MR‐Egger and weighted median, while sensitivity analyses were conducted to address heterogeneity and horizontal pleiotropy. The IVW results confirmed the causal relationship between certain gut microbiota groups and psoriasis. Specifically, *family Veillonellaceae* (OR = 1.162, 95% CI: 1.038–1.301, *p* = 0.009), *genus Candidatus Soleaferrea* (OR = 1.123, 95% CI: 1.011–1.247, *p* = 0.030) and *genus Eubacterium fissicatena group* (OR = 0.831, 95% CI: 0.755–0.915, *p* = 0.00016) showed significant associations. Sensitivity analysis did not reveal any abnormalities in SNPs. Currently, we have found some causal relationship between the gut microbiota and psoriasis. However, the study needs further RCTs for further validation.

## 1 BACKGROUND

As the prevalence of autoimmune diseases continues to rise, the role of gut microbiota in these diseases has become a major area of research. Recent advances have shown associations between gut microbiota and the onset or exacerbation of autoimmune diseases, suggesting potential causal relationships. Moreover, microbial dysbiosis is a common phenomenon observed in human autoimmune diseases and mouse models of autoimmune diseases. However, observational studies are often confounded by various factors and reverse causality, making it challenging to draw definitive conclusions about the aetiology of psoriasis. Over the years, Mendelian randomization (MR) analysis has become a focus, providing a unique perspective and insights into the aetiology of autoimmune diseases, including psoriasis, and offering the possibility of identifying potential therapeutic interventions. In MR analysis,^1^ genetic variations are used as instrumental variables to explore the associations between exposure factors and disease or other outcomes. Therefore, taking into account the inherent assumptions and limitations of MR analysis can provide a valuable approach to explore causal relationships between different risk factors and autoimmune diseases like psoriasis. This study primarily investigates the results of MR in psoriasis research, focusing on the impact of gut microbiota on psoriasis risk.

Psoriasis is a chronic inflammatory skin disease characterized by autoimmune pathogenesis, affecting approximately 2% of the global population.^2^ It is caused by chronic inflammation, resulting in uncontrolled growth and defective differentiation of keratinocytes. It exhibits autoimmune pathogenic features and a strong genetic susceptibility. The majority of psoriasis cases involve chronic plaque psoriasis^3^, ^4^ (referred to as psoriasis vulgaris). The typical clinical symptoms include well‐defined erythematous, pruritic plaques that can cover large areas of the skin.^5^, ^6^, ^7^, ^8^

Microbiota,^9^ which has been present in the human environment since birth, plays a crucial role in human pathology and physiology. Microbiota refers to the microbial communities living on the surface of animal skin or mucosa, which interact not only with each other but also with the host.^10^, ^11^ These interactions can be beneficial, symbiotic, harmful or pathogenic. Among various human microbiota, the gut microbiota and skin microbiota are among the most extensively studied. Gut microbiota bacteria are predominantly from the *phyla Actinobacteria*, *Bacteroidetes*, *Firmicutes* and *Proteobacteria*, and their composition is affected by host factors such as age and diet. The adult gut harbours a vast number of different bacterial species collectively known as the gut microbiota, which is unevenly distributed throughout the gastrointestinal tract, with the main concentration in the lower gut. It is more diverse in the oral cavity and intestines than in the stomach, mainly due to the acidic environment of the latter. Oxygen‐requiring bacteria are primarily found in the small intestine, while anaerobic bacteria are mainly present in the colon. The gut microbiota provides significant metabolic and immune benefits to the host. It participates in the breakdown of complex carbohydrates that are difficult to digest and is essential for the production of certain nutrients (e.g. vitamin K). It has a profound impact on the host's immune system.^12^ The presence of microbes in the gastrointestinal tract induces immune tolerance to dietary and environmental antigens by competitively binding to epithelial cells or triggering protective immune responses, maintaining the host in a state of equilibrium. Changes in gut microbiota can alter gut permeability, leading to the translocation of bacterial metabolites into the bloodstream, subsequently modulating the host's immune response.

In recent years, there have been reports of an increasing incidence and prevalence of psoriasis. While the reasons for this trend are likely multifactorial, it is speculated that the rising incidence of autoimmune diseases is attributed to changes in diet and the widespread use of antibiotics, leading to significant alterations in the microbial communities residing in the gut (collectively known as gut microbiota). It has been shown that gut microbiota diversity affects immune development and disease risk, particularly in autoimmune diseases like psoriasis.^13^ In patients with psoriasis, the composition of the gut microbiota varies depending on the severity and status of the disease. Various studies have reported different findings regarding the gut microbiota composition in psoriasis subjects. For instance, the levels of *the genus Prevotella (species)* have been found to be higher or lower in psoriasis subjects compared to healthy controls, both reflecting gut ecological dysbiosis. Based on a study using microbial and inflammation‐related variables, microbial dysbiosis may lead to abnormal immune responses in psoriasis. The dysbiosis of the gut microbiota is associated with the extent of irregular inflammation‐related markers in psoriasis, particularly IL‐2 receptor, which is positively correlated with *the genus Coprococcus* and negatively correlated with *the genus Dialister*. The relative abundance of *the genus* *Coprococcus* and *the genus Dialister* can serve as predictors of psoriasis activity. Additionally, complement 3 is negatively correlated with levels of *Escherichia*, which tend to be higher in psoriasis patients. Among 21 patients with psoriasis in Brazil, gut microbiota composition and diversity were studied, and patients with psoriasis showed reduced levels of *Lachnospira* and *Akkermansia muciniphila*, as compared to the control group. Another study also highlighted the reduction of *Akkermansia*, which was examined using 16S rDNA sequencing in 14 psoriasis patients. It has been suggested that these changes are related to the metabolism and production of butyric acid in the colonic microbiome. Butyrate is connected to several inflammatory factors, including lipopolysaccharides, TNF‐α, IL‐10 and IL‐1β. The abundance of faecal bacteria is lower in psoriasis patients, and there are differences in beta diversity community composition, with higher levels of *Methanobrevibacter* and *Methanomassiliicoccus luminyensis*. Despite the limited number of subjects included in such studies, the potential functional capabilities of gut microbiota in psoriasis patients appear to be diminished due to gut ecological dysbiosis. With the advancement of sequencing technologies and projects such as the Human Microbiome Project, the Human Intestinal Metagenomic Project and the National Microbiome Initiative (NMI), more researchers are exploring the relationship between gut microbiota and the human host. However, due to limited sample sizes, different racial confounders, biases in cohort and cross‐sectional studies, on the basis of observational studies alone, it is difficult to draw definitive conclusions about the causal relationship between gut microbiota and psoriasis. In many academic investigations of the correlation between the gut microbiota and disease, MR has been used as a convenient tool for investigating potential protective and risk factors.^14^, ^15^, ^16^ We used pooled datasets from genome‐wide association studies (GWAS) of gut microbiota and psoriasis for analysis.

## 2 MATERIALS AND METHODS

### 2.1 Data sources

**FIGURE 1:**
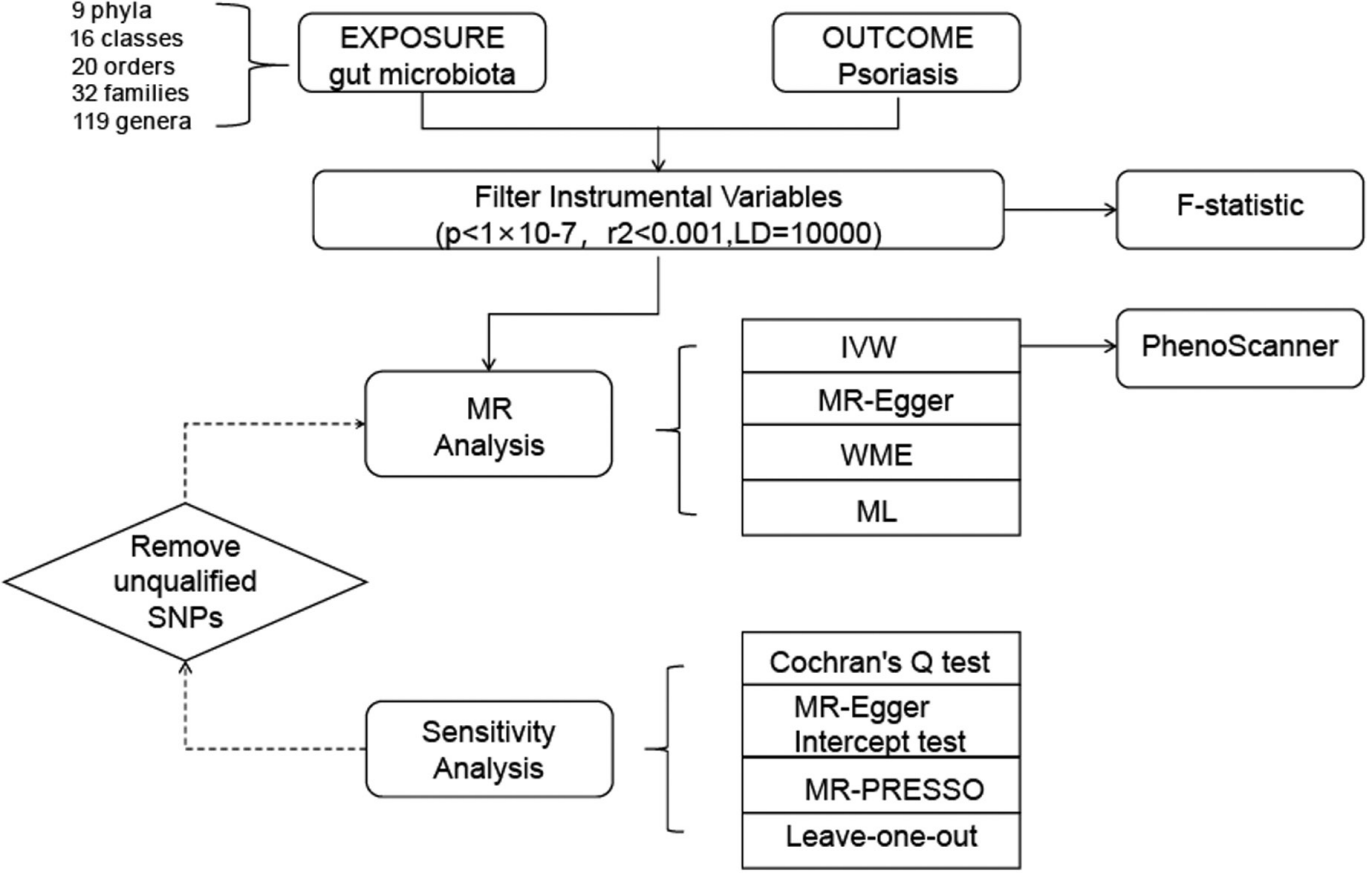
Workflow of Mendelian randomization (MR) analysis.

Our workflow diagram is presented in Figure 1. The MiBioGen group released 99 the biggest genome‐wide meta‐analysis of gut microbiota composition, which 100 included genetic variation data for the gut microbiota.^17^ The dataset included genetic variation data of gut microbiota. This study included genetic data from 18,340 individuals and 16S rRNA gene typing data (Table S1). All individuals were of European descent. In addition, data from the FinnGen project (https://www.finngen.fi/en/) with the code ‘L12_PSORIASIS’ were used, which consisted of 373,338 samples, including 9267 psoriasis cases and 364,071 controls, with a total of 20,170,177 SNPs.

### 2.2 Instrumental variable (IV) selection

Mendelian randomization (MR) is an important method for inferring causal relationships between traits. It uses genetic variation as instrumental variables (IVs)^18^ in a model based on GWAS data to infer the causal relationship between exposure and outcome. Gut microbiota and psoriasis were considered exposure and outcome, respectively. The selected IVs must meet three key assumptions: (1) genetic variation is associated with the exposure factor; (2) any confounders between genetic variation and the exposure‐outcome association are unrelated; (3) the genetic variation does not influence the outcome except through its association with the exposure factor. To meet these criteria, we first used single nucleotide polymorphisms (SNPs) as IVs and set a significance threshold of a *p* < 1 × 10 − 7 to exclude SNPs with low significance and remove highly correlated SNPs to ensure their independence. Furthermore, to ensure the independence of SNPs and avoid linkage disequilibrium (LD) bias,^18^ we set an LD threshold of *r* ^2^ < 0.001 and a distance of 10,000 kb. Additionally, we used the Phenome‐wide scan online tool to assess whether these SNPs were associated with confounders in psoriasis and manually removed SNPs associated with these confounders.

### 2.3 Statistical analysis

Based on the current established statistical data, further analysis was conducted using R software, and data filtering was performed using packages such as TwoSampleMR and MR‐PRESSO. Previous studies have shown that the IVW method is widely used in MR research^19^, ^20^, ^21^ and has the strongest test efficacy. In order to eliminate the influence of research methods on the results and to improve the accuracy of the results, this study used the fixed‐effects model and the random‐effect model of the IVW analysis method. The random‐effect model reduces the bias of the results due to the presence of heterogeneity. All SNPs included in the IVW method must satisfy the three assumptions of IVS selection, especially the exclusivity assumption, which requires that genetic variation affects the outcome only through the exposure factors in the study. Although known confounding SNPs were excluded as much as possible during the course of the study, estimates of causal effects may still be biassed by genetic pleiotropy due to many unknown factors. Therefore, this study used MR‐Egger regression, weighted median estimator (WME) method to test the stability of the results. MR‐Egger regression modifies the IVW method to both test for pleiotropy and correct for pleiotropy bias. However, the MR‐Egger regression method is less statistically valid for causal estimation, and some researchers have pointed out that the MR‐Egger regression method can only be used as a sensitivity analysis to test whether it violates the core assumptions of the instrumental variables and not as an alternative to the IVW method. The WME method gives consistent results when faced with the fact that some of the genetic variations in the analyses are not valid IVs.

### 2.4 Sensitivity analysis

Sensitivity analyses include heterogeneity test and multiplicity test. Heterogeneity test is the possibility of heterogeneity in the MR analysis method due to the fact that there may be differences between the studies, such as different platforms for gene annotation and analysis, different inclusion and exclusion criteria, etc. In this study, the heterogeneity test of IVW method and MR‐Egger regression method was performed using Cochran's *Q* test. If the result of the heterogeneity test is *p* > 0.05, there is no statistically significant, at which point there is no effect of heterogeneity on the results of the study. As the intercept term in MR‐Egger regression approaches zero, the magnitude of horizontal pleiotropy is smaller. Horizontal pleiotropy was considered not to exist if the test for horizontal pleiotropy resulted in *p* > 0.05. To determine whether there were any outlier SNPs contributing to the overall results, we used the MR‐PRESSO outlier test. We also performed a ‘leave‐one‐out’ analysis, removing one SNP from the analysis, we calculate the Mendelian randomization effect of the remaining SNPs. Through the presentation of forest plots, we could visually judge the effect of each SNP on the results and thus judge the stability of the MR analysis results. Furthermore, we conducted MR Steiger tests to verify causal direction.

## 3 RESULTS

**FIGURE 2:**
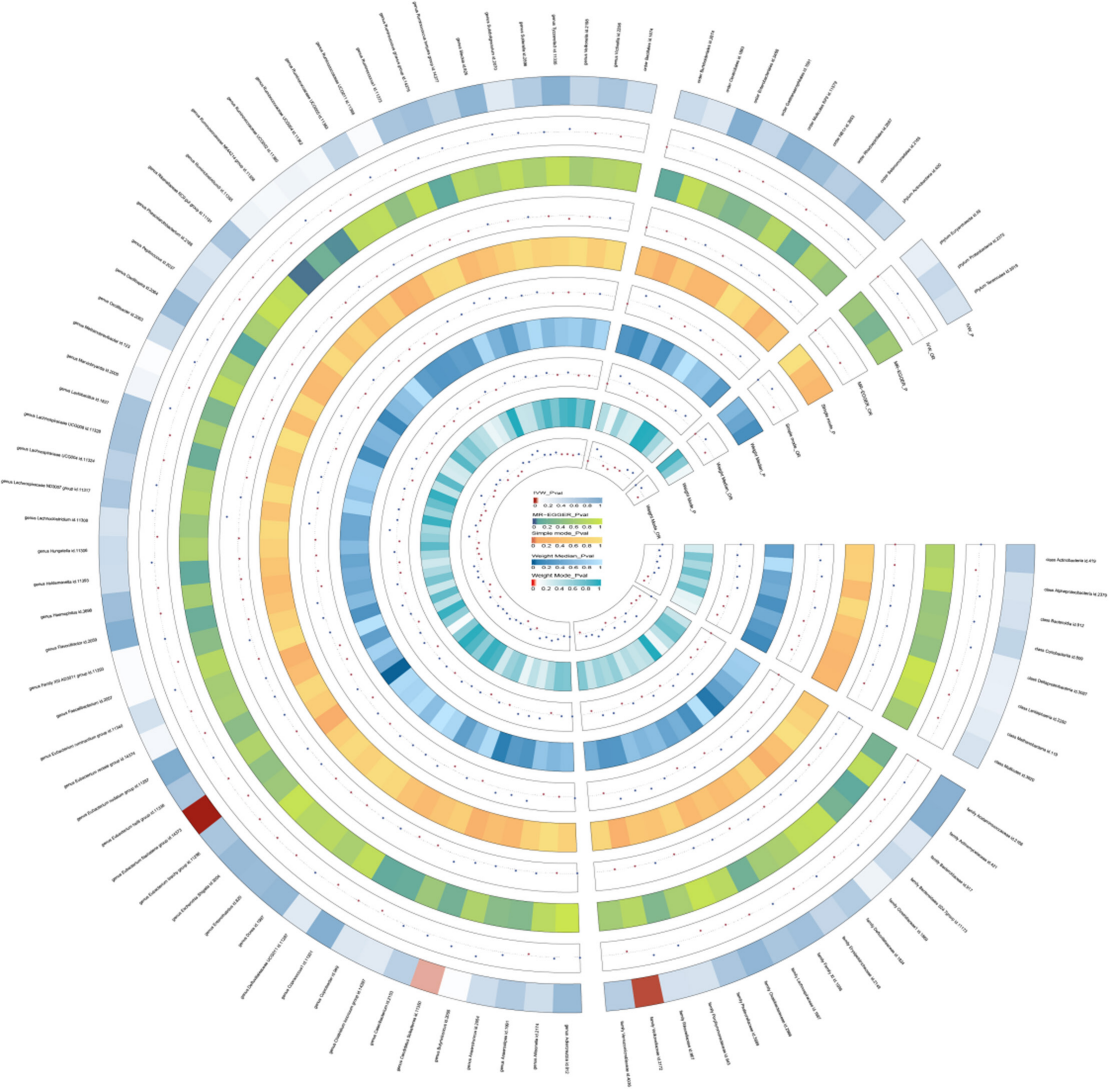
A circus plot showing four methods results of all gut microbiota.

Based on the described methodology, the strength of instrumental variables (IVs) was assessed. We initially selected SNPs closely associated with the gut microbiome from the GWAS database (Table S2), and we also performed linkage disequilibrium (LD) analysis^21^ to eliminate any potential linkage ambiguity. Additionally, we utilized phenoscanner to search for confounding factors related to the studied phenotype. During the screening process using the software, we removed any potential risk factors that could impact the analysis process and outcomes, ensuring the reliability of our IV selection (Table S4). Ultimately, we identified 1266 SNPs from a total of 196 gut microbiome variables as instrumental variables. To visually represent the strength of the IVs and make the compiled data more intuitive, we created a circular heat map to present this part of the data (Figure 2). For detailed information, please refer to Table S3.

**FIGURE 3:**
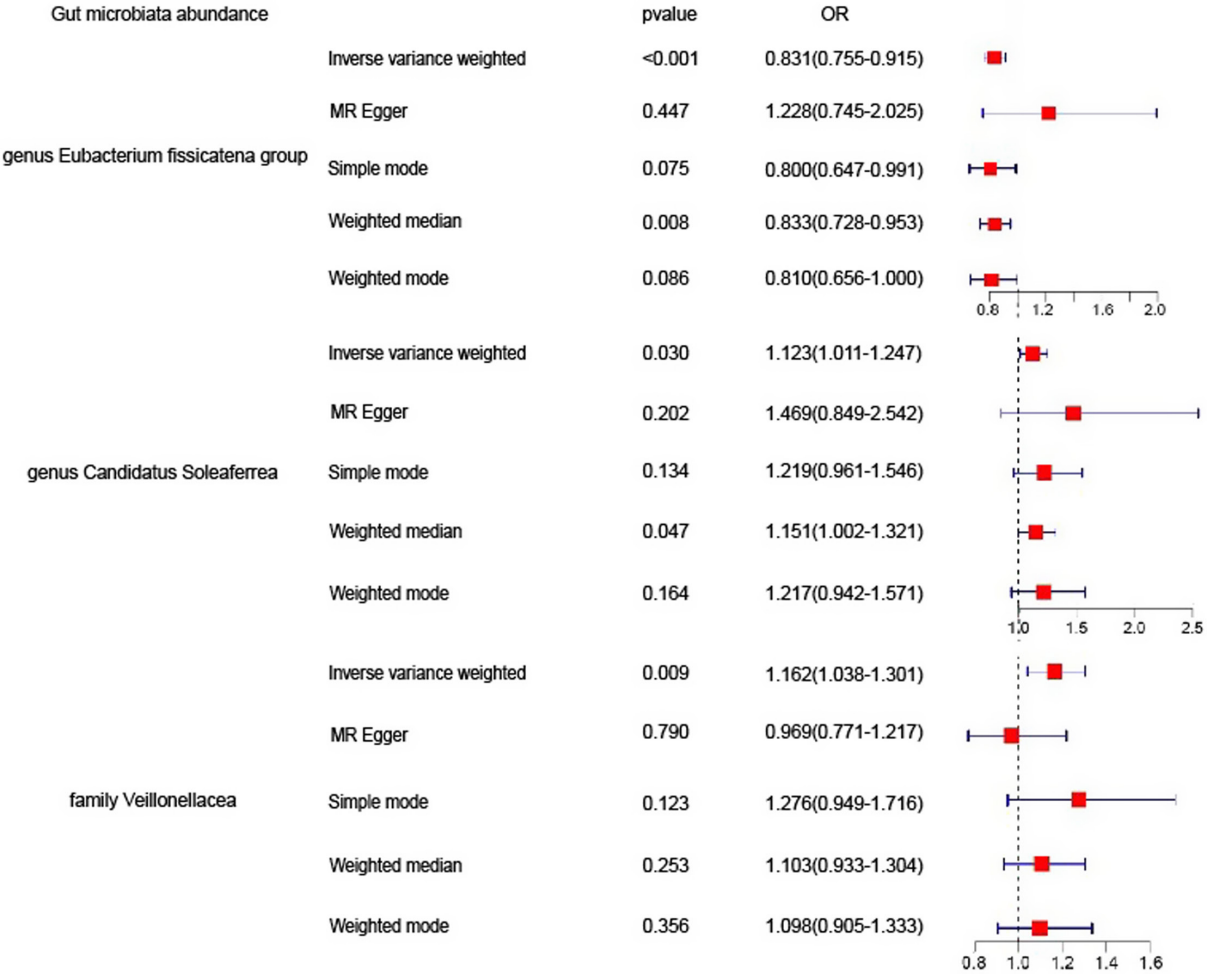
Significant results for IVW analysis.

**FIGURE 4:**
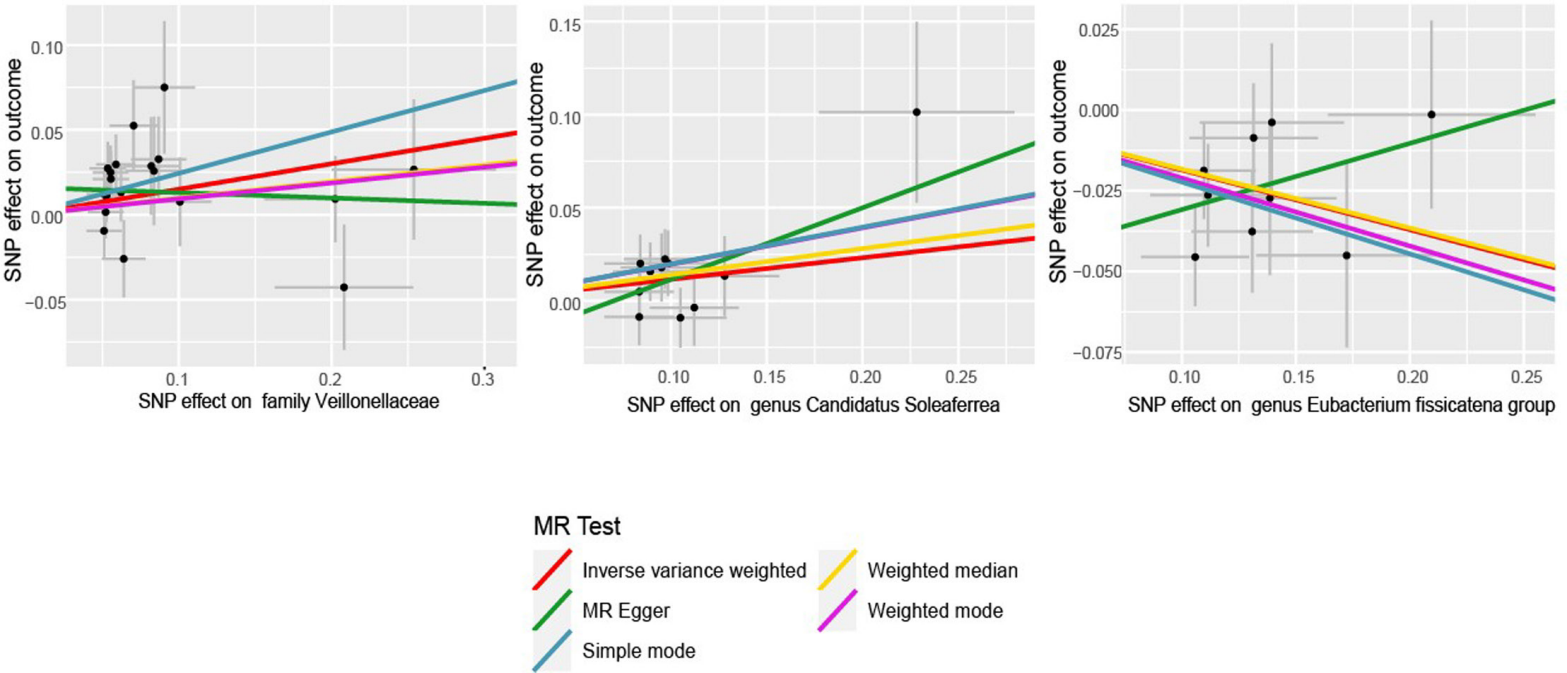
Scatter plots of the Mendelian randomization (MR) analysis.

**FIGURE 5:**
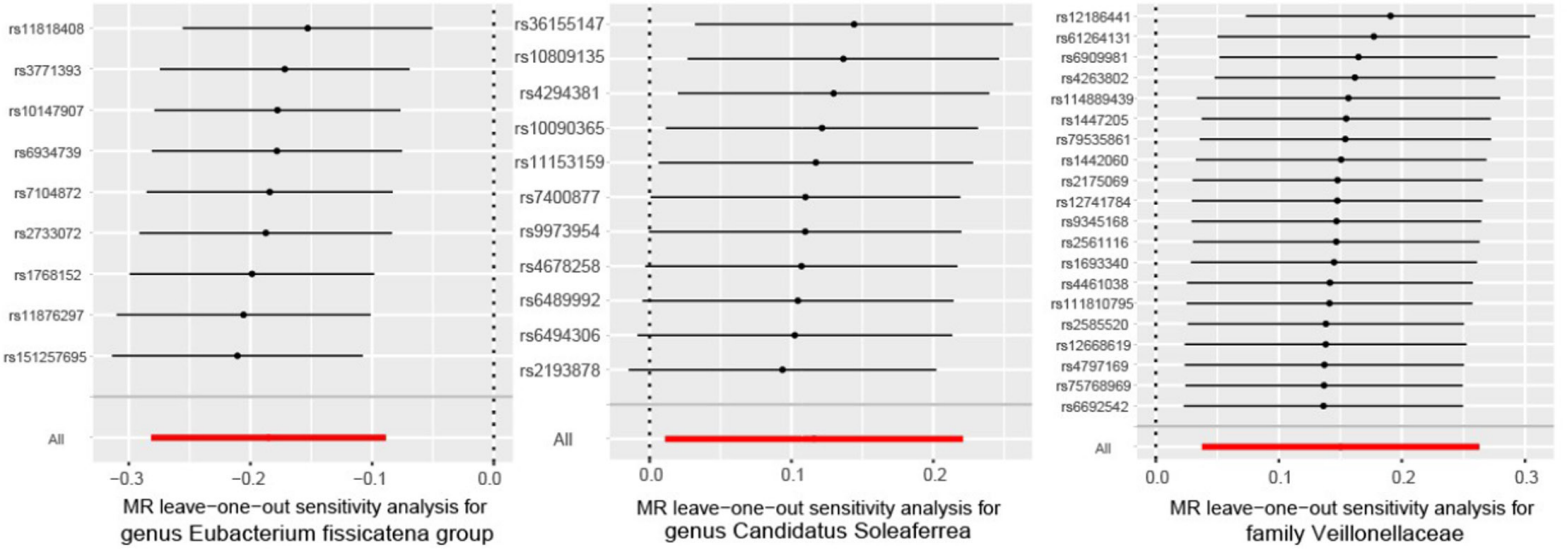
The leave‐one‐out results.

The gut microbiome exhibited significant IVW analysis results for *the family Veillonellaceae* (OR = 1.162, 95% CI: 1.038–1.301, *p* = 0.009), *genus Candidatus Soleaferrea* (OR = 1.123, 95% CI: 1.011–1.247, *p* = 0.030), and *genus Eubacterium fissicatena group* (OR = 0.831, 95% CI: 0.755–0.915, *p* = 0.00016) (Figure 3). Table 1 presents the sensitivity analysis results,^20^ which primarily assess the reliability of the MR analysis. In the Cochran's *Q* Test, none of the gut microbiome variables showed significance, indicating no heterogeneity among the instrumental variables (IVs). The pleiotropy test results revealed that after conducting the MR‐PRESSO test and removing SNPs that cause bias, the data were free from any outliers. In the MR‐Egger analysis, we examined whether the remaining IVs still exhibited pleiotropy, and the results indicated that the intercept analysis did not yield meaningful results, suggesting the absence of horizontal pleiotropy (Figure 4). Leave‐one‐out analysis, which involved sequentially removing each SNP, revealed that no single SNP had a substantial influence on the robustness of the results. Therefore, the correlation analysis between the gut microbiome and psoriasis yielded robust results (Figure 5).

**TABLE 1:** Sensitivity analysis of significant gut microbiota.

Gut microbiota	*Q*_*p*val (IVW)	MR‐PRESSO	MR‐egger test		Steiger test
			Intercept	Pleiotropy test	
*Family Veillonellaceae*	0.427	0.412	0.016	0.091	3.24E‐85
*Genus Candidatus Soleaferrea*	0.650	0.650	−0.027	0.353	1.29E‐58
*Genus Eubacterium fissicatena group*	0.529	0.594	−0.051	0.168	3.23E‐38

Abbreviation: MR, Mendelian randomization.

## 4 DISCUSSION

Psoriasis is an inflammatory skin disease characterized by chronicity and relapse, with clinical features of erythema, scales and well‐defined borders, typically affecting the scalp and extensor surfaces of the limbs.^22^, ^23^, ^24^, ^25^, ^26^, ^27^ Around 2% of the world's population is affected, with a higher prevalence in China, where millions are affected. Studies have shown that dysbiosis of the gut microbiota leading to intestinal barrier damage and bacterial translocation can promote the development of chronic systemic inflammatory diseases, making it a potential trigger for psoriasis.^28^, ^29^ This is due to the release of potent inflammatory mediators such as lipopolysaccharides (LPS) and lipoteichoic acid (LTA) by *gram‐negative* and *gram‐positive bacteria*, respectively. Evidence indicates that barrier integrity impairment and bacterial translocation are associated with the development of psoriasis.

In our study, MR analysis of the gut microbiota suggested that this is causally related to psoriasis. We employed the extensive GWAS meta‐analysis data on the gut microbiome obtained from the MiBioGen consortium and statistical information on psoriasis published by FinnGen to examine potential causal relationships. Mendelian randomization and sensitivity analyses of selected eligible instrumental variables revealed causal relationships between several gut microbiomes and psoriasis, with results both robust across different MR approaches and demonstrated interactions between these two traits. Specifically, the *Veillonella species* of the *family* *Veillonellaceae* ^30^, ^31^ and a potential novel species of fermentative anaerobic clostridium (*Candidatus Soleaferrea*) were associated with an increased occurrence of psoriasis (OR > 1). On the other hand, *the Eubacterium species* of *gram‐positive bacteria* ^32^ were associated with a reduced incidence of psoriasis (OR < 1). There are few studies on the relationship between identified microorganisms and psoriasis.

The potential novel species *Candidatus Soleaferrea*, associated with psoriasis, has previously been reported to be positively correlated with glucagon‐like peptide 2 (GLP‐2). Metabolites secreted by the gut microbiota, including GLP‐2, are considered to be nutrient hormones involved in maintaining intestinal epithelial morphology and function. Although there is limited knowledge about the ability of this genus to alter the abundance of GLP‐2 and its impact on immune‐related diseases, research suggests that it may exert anti‐inflammatory effects through metabolite secretion and protection of intestinal homeostasis, warranting further investigation.

Studies have shown^33^ that *Veillonella species* can metabolize glucose and lactose into short‐chain fatty acids. These short‐chain fatty acids cannot synthesize mucins but can increase intestinal mucosal permeability, promoting the formation of intestinal inflammation. Previous studies have found that their gut microbiomes are similar. A 2014 study by British scholars found^34^, ^35^ a significant increase in the *family Veillonellaceae* in the intestines of inflammatory bowel disease patients, consistent with our findings. Regarding the gram‐positive bacteria of *the genus Eubacterium*, research results indicate that the abundance of *Bacteroidetes* and *Firmicutes* is significantly reduced, leading to an increased *Firmicutes/Bacteroidetes (F/B)* ratio. It has been suggested that an elevated *F/B* ratio can affect carbohydrate metabolism and promote chronic low‐grade inflammation, which may be a contributing factor to increased risk of obesity and type 2 diabetes in psoriasis patients.

Our study has the following advantages and innovations: To begin with, MR analysis uses the principle of Mendelian randomized free segregation, which excludes the influence of acquired factors on the study's results (e.g. social and natural environments, etc.) on the results of the study at the genetic level, and the genes appeared prior to the disease, with a clear causal sequence, which can effectively make up for the shortcomings of traditional observational studies that are susceptible to the interference of confounding factors and reverse causality in the etiological inference of complex diseases. factors and reverse causality in the inference of complex diseases in traditional observational studies. Lastly, we performed a pooled analysis of data on gut microbiota and psoriasis, which will improve reliability and generalizability. This study also has some limitations, which include the following: 1. The data obtained in this study were those after meta‐analysis and after the Mendelian Randomized Polymorphism Residuals and Outliers (MR‐PRESSO) method to exclude outliers that may lead to horizontal pleiotropy, it is still not completely certain that the selected SNPs comply with the exclusionary assumption. This study used multiple statistical methods to minimize the impact of outlier data on the findings. 2. Ethnicity factors can affect the study of gene levels. In this study, the GWAS data used were mainly derived from European populations, which does limit the generalization of the present findings to other ethnic groups, and follow‐up studies are needed to further validate them. 3. In the future, we can use more precise screening criteria to improve the reliability and accuracy of the MR analysis' instrumental variables. In conclusion, our MR analysis of two independent samples established a causal relationship between psoriasis and the gut microbiome. *The Veillonella species* of the *family* *Veillonellaceae* and *Eubacterium* *species* were found to influence the occurrence of psoriasis, providing new insights for the diagnosis and treatment of psoriasis.

## AUTHOR CONTRIBUTIONS

**Ruonan Wu:** Writing – original draft (equal). **Li Zhao:** Resources (equal). **Zewen Wu:** Resources (equal). **Xinling Liu:** Conceptualization (equal). **Jingxuan Li:** Conceptualization (equal). **Tong Wang:** Writing – review and editing (equal). **Liyun Zhang:** Funding acquisition (equal).

## FUNDING INFORMATION

This work was supported by grants from the National Natural Science Foundation of China (No. 81771768 to Liyun Zhang). This work was supported by the basic research project of Shanxi Science and Technology Department (grant number 202103021224342) and Key Research and Development Projects of Shanxi Province (2021XM01). Shanxi Province Clinical Diagnosis and Treatment Technology Innovation Center for Immunology and Rheumatic Diseases Research Project (CXZX‐202307).

## CONFLICT OF INTEREST STATEMENT

The authors declare that this research was conducted in the absence of any commercial or financial relationships which could be construed as potential conflicts of interest.

## Supporting information


Tables S1–S4.

## Data Availability

This study does not include data that require ethical approval and consent.

## References

[1] Breban M . Gut microbiota and inflammatory joint diseases. Joint Bone Spine. 2016;83(6):645‐649. 27238190 10.1016/j.jbspin.2016.04.005

[2] Boehncke WH , Schön MP . Psoriasis. Lancet. 2015;386(9997):983‐994. 26025581 10.1016/S0140-6736(14)61909-7

[3] Damiani G , Bragazzi NL , Mccormick TS , et al. Gut microbiota and nutrient interactions with skin in psoriasis: a comprehensive review of animal and human studies. World J Clin Cases. 2020;8(6):1002‐1012. 32258071 10.12998/wjcc.v8.i6.1002 PMC7103976

[4] Buhaș MC , Gavrilaș LI , Candrea R , et al. Gut microbiota in psoriasis. Nutrients. 2022;14(14):2970. 35889927 10.3390/nu14142970 PMC9321451

[5] Olejniczak‐Staruch I , Ciążyńska M , Sobolewska‐Sztychny D , Narbutt J , Skibińska M , Lesiak A . Alterations of the skin and gut microbiome in psoriasis and psoriatic arthritis. Int J Mol Sci. 2021;22(8):3998. 33924414 10.3390/ijms22083998 PMC8069836

[6] Visser M , Kell DB , Pretorius E . Bacterial dysbiosis and translocation in psoriasis vulgaris. Front Cell Infect Microbiol. 2019;9:7. 30778377 10.3389/fcimb.2019.00007 PMC6369634

[7] Stec A , Sikora M , Maciejewska M , et al. Bacterial metabolites: a link between gut microbiota and dermatological diseases. Int J Mol Sci. 2023;24(4):3494. 36834904 10.3390/ijms24043494 PMC9961773

[8] Paine A , Ritchlin C . Bone remodeling in psoriasis and psoriatic arthritis: an update. Curr Opin Rheumatol. 2016;28(1):66‐75. 26555451 10.1097/BOR.0000000000000232

[9] Adak A , Khan MR . An insight into gut microbiota and its functionalities. Cell Mol Life Sci. 2019;76(3):473‐493. 30317530 10.1007/s00018-018-2943-4 PMC11105460

[10] Yu Y , Dunaway S , Champer J , Kim J , Alikhan A . Changing our microbiome: probiotics in dermatology. Br J Dermatol. 2020;182(1):39‐46. 31049923 10.1111/bjd.18088

[11] Rozas M , Hart DRA , Fabrega MJ , et al. From dysbiosis to healthy skin: major contributions of *Cutibacterium acnes* to skin homeostasis. Microorganisms. 2021;9(3):628. 33803499 10.3390/microorganisms9030628 PMC8003110

[12] Stoll ML . Gut microbes, immunity, and spondyloarthritis. Clin Immunol. 2015;159(2):134‐142. 25967460 10.1016/j.clim.2015.05.001

[13] Sikora M , Stec A , Chrabaszcz M , et al. Gut microbiome in psoriasis: An updated review. Pathogens. 2020;9(6):463. 32545459 10.3390/pathogens9060463 PMC7350295

[14] Freidin MB , Stalteri MA , Wells PM , et al. An association between chronic widespread pain and the gut microbiome. Rheumatology (Oxford). 2021;60(8):3727‐3737. 33331911 10.1093/rheumatology/keaa847 PMC8328510

[15] Girault JM , Ménigot S . Palindromic vectors, Symmetropy and Symmentropy as symmetry descriptors of binary data. Entropy (Basel). 2022;24(1):82. 35052108 10.3390/e24010082 PMC8774538

[16] Guo P , Zhang K , Ma X , He P . *Clostridium* species as probiotics: potentials and challenges. J Anim Sci Biotechnol. 2020;11:24. 32099648 10.1186/s40104-019-0402-1 PMC7031906

[17] Wang J , Kurilshikov A , Radjabzadeh D , et al. Meta‐analysis of human genome‐microbiome association studies: the MiBioGen consortium initiative. Microbiome. 2018;6(1):101. 29880062 10.1186/s40168-018-0479-3 PMC5992867

[18] Burgess S , Small DS , Thompson SG . A review of instrumental variable estimators for Mendelian randomization. Stat Methods Med Res. 2017;26(5):2333‐2355. 26282889 10.1177/0962280215597579 PMC5642006

[19] Bowden J , Holmes MV . Meta‐analysis and Mendelian randomization: a review. Res Synth Methods. 2019;10(4):486‐496. 30861319 10.1002/jrsm.1346 PMC6973275

[20] Roze D . Causes and consequences of linkage disequilibrium among transposable elements within eukaryotic genomes. Gentics. 2023;224(2):iyad058. 10.1093/genetics/iyad058 37019818

[21] Wang C , Wu W , Yang H , et al. Mendelian randomization analyses for PCOS: evidence, opportunities, and challenges. Trends Genet. 2022;38(5):468‐482. 35094873 10.1016/j.tig.2022.01.005

[22] Sekula P , Del GMF , Pattaro C , Köttgen A . Mendelian randomization as an approach to assess causality using observational data. J Am Soc Nephrol. 2016;27(11):3253‐3265. 27486138 10.1681/ASN.2016010098 PMC5084898

[23] Thye AY , Bah YR , Law JW , et al. Gut‐skin axis: unravelling the connection between the gut microbiome and psoriasis. Biomedicine. 2022;10(5):1037. 10.3390/biomedicines10051037 PMC9138548 35625774

[24] Madden SK , Flanagan KL , Jones G . How lifestyle factors and their associated pathogenetic mechanisms impact psoriasis. Clin Nutr. 2020;39(4):1026‐1040. 31155371 10.1016/j.clnu.2019.05.006

[25] Colucci R , Moretti S . Implication of human bacterial gut microbiota on immune‐mediated and autoimmune dermatological diseases and their comorbidities: a narrative review. Dermatol Ther (Heidelb). 2021;11(2):363‐384. 33507493 10.1007/s13555-021-00485-0 PMC8018919

[26] Hedin C , Sonkoly E , Eberhardson M , Ståhle M . Inflammatory bowel disease and psoriasis: modernizing the multidisciplinary approach. J Intern Med. 2021;290(2):257‐278. 33942408 10.1111/joim.13282

[27] Fragoulis GE , Liava C , Daoussis D , Akriviadis E , Garyfallos A , Dimitroulas T . Inflammatory bowel diseases and spondyloarthropathies: from pathogenesis to treatment. World J Gastroenterol. 2019;25(18):2162‐2176. 31143068 10.3748/wjg.v25.i18.2162 PMC6526158

[28] Dumas E , Venken K , Rosenbaum JT , Elewaut D . Intestinal Microbiota, HLA‐B27, and Spondyloarthritis: Dangerous Liaisons. Rheum Dis Clin North Am. 2020;46(2):213‐224. 32340697 10.1016/j.rdc.2020.01.007

[29] Li B , Yang B , Liu X , et al. Microbiota‐assisted therapy for systemic inflammatory arthritis: advances and mechanistic insights. Cell Mol Life Sci. 2022;79(9):470. 35932328 10.1007/s00018-022-04498-6 PMC11072763

[30] Jiraskova ZZ , Reiss Z , Tlaskalova‐Hogenova H , Rob F . Paradoxical reactions to anti‐TNFα and anti‐IL‐17 treatment in psoriasis patients: are skin and/or gut microbiota involved? Dermatol Ther (Heidelb). 2023;13(4):911‐933. 36929119 10.1007/s13555-023-00904-4 PMC10060503

[31] Luo YX , Sun ML , Shi PL , Liu P , Chen YY , Peng X . Research progress in the relationship between Veillonella and oral diseases. Hua Xi Kou Qiang Yi Xue Za Zhi. 2020;38(5):576‐582. 33085245 10.7518/hxkq.2020.05.018 PMC7573782

[32] Megrian D , Taib N , Witwinowski J , Beloin C , Gribaldo S . One or two membranes? Diderm Firmicutes challenge the Gram‐positive/Gram‐negative divide. Mol Microbiol. 2020;113(3):659‐671. 31975449 10.1111/mmi.14469

[33] Mukherjee A , Lordan C , Ross RP , Cotter PD . Gut microbes from the phylogenetically diverse genus *Eubacterium* and their various contributions to gut health. Gut Microbes. 2020;12(1):1802866. 32835590 10.1080/19490976.2020.1802866 PMC7524325

[34] Rogler G , Singh A , Kavanaugh A , Rubin DT . Extraintestinal manifestations of inflammatory bowel disease: current concepts, treatment, and implications for disease management. Gastroenterology. 2021;161(4):1118‐1132. 34358489 10.1053/j.gastro.2021.07.042 PMC8564770

[35] Lamb CA , Kennedy NA , Raine T , et al. British Society of Gastroenterology consensus guidelines on the management of inflammatory bowel disease in adults. Gut. 2019;68(Suppl 3):s1‐s106. 31562236 10.1136/gutjnl-2019-318484 PMC6872448

