# Supplementary material for: Psoriasis and gut microbiota: A Mendelian randomization study
Source: J Cell Mol Med. 2023 Dec 25;28(1):e18023. doi: 10.1111/jcmm.18023 (PMC10805496; doi:10.1111/jcmm.18023)
Supplement: Supplementary file 1 — Tables S1–S4. [file JCMM-28-e18023-s001.docx]

Supplementary Table 1:Summary of gut microbiota information.

| id | | trait | year | pmid | population | sample_size | nsnp |
| --- | --- | --- | --- | --- | --- | --- | --- |
| FAMILY | ebi-a-GCST90016924 | family Acidaminococcaceae id.2167 | 2021 | 33462485 | European | 14306 | 5547067 |
|  | ebi-a-GCST90016925 | family Actinomycetaceae id.422 | 2021 | 33462485 | European | 14306 | 5424030 |
|  | ebi-a-GCST90016927 | family Bacteroidaceae id.918 | 2021 | 33462485 | European | 14306 | 5729148 |
|  | ebi-a-GCST90016928 | family Bacteroidales S24 7group id.11174 | 2021 | 33462485 | European | 14306 | 5432872 |
|  | ebi-a-GCST90016931 | family Clostridiaceae1 id.1870 | 2021 | 33462485 | European | 14306 | 5598586 |
|  | ebi-a-GCST90016934 | family Defluviitaleaceae id.1925 | 2021 | 33462485 | European | 14306 | 5440708 |
|  | ebi-a-GCST90016937 | family Erysipelotrichaceae id.2150 | 2021 | 33462485 | European | 14306 | 5720820 |
|  | ebi-a-GCST90016938 | family Family XI id.1937 | 2021 | 33462485 | European | 14306 | 4330602 |
|  | ebi-a-GCST90016940 | family Lachnospiraceae id.1988 | 2021 | 33462485 | European | 14306 | 5729268 |
|  | ebi-a-GCST90016943 | family Oxalobacteraceae id.2967 | 2021 | 33462485 | European | 14306 | 5323881 |
|  | ebi-a-GCST90016944 | family Pasteurellaceae id.3690 | 2021 | 33462485 | European | 14306 | 5491834 |
|  | ebi-a-GCST90016947 | family Porphyromonadaceae id.944 | 2021 | 33462485 | European | 14306 | 5716664 |
|  | ebi-a-GCST90016950 | family Rikenellaceae id.968 | 2021 | 33462485 | European | 14306 | 5665279 |
|  | ebi-a-GCST90016956 | family Veillonellaceae id.2173 | 2021 | 33462485 | European | 14306 | 5688519 |
|  | ebi-a-GCST90016957 | family Verrucomicrobiaceae id.4037 | 2021 | 33462485 | European | 14306 | 5518011 |
|  | ebi-a-GCST90016953 | unknown family id.1000001215 | 2021 | 33462485 | European | 14306 | 5355169 |
|  | ebi-a-GCST90016954 | unknown family id.1000005472 | 2021 | 33462485 | European | 14306 | 5488779 |
| CLASS | ebi-a-GCST90016908 | class Actinobacteria id.420 | 2021 | 33462485 | European | 14306 | 5684804 |
|  | ebi-a-GCST90016909 | class Alphaproteobacteria id.2380 | 2021 | 33462485 | European | 14306 | 5470627 |
|  | ebi-a-GCST90016911 | class Bacteroidia id.913 | 2021 | 33462485 | European | 14306 | 5729267 |
|  | ebi-a-GCST90016914 | class Coriobacteriia id.810 | 2021 | 33462485 | European | 14306 | 5698092 |
|  | ebi-a-GCST90016915 | class Deltaproteobacteria id.3088 | 2021 | 33462485 | European | 14306 | 5616854 |
|  | ebi-a-GCST90016918 | class Lentisphaeria id.2251 | 2021 | 33462485 | European | 14306 | 5282234 |
|  | ebi-a-GCST90016920 | class Methanobacteria id.120 | 2021 | 33462485 | European | 14306 | 5118613 |
|  | ebi-a-GCST90016921 | class Mollicutes id.3921 | 2021 | 33462485 | European | 14306 | 5510407 |
|  | ebi-a-GCST90016960 | genus Adlercreutzia id.813 | 2021 | 33462485 | European | 14306 | 5437332 |
|  | ebi-a-GCST90016963 | genus Allisonella id.2175 | 2021 | 33462485 | European | 14306 | 4116508 |
|  | ebi-a-GCST90016966 | genus Anaerostipes id.1992 | 2021 | 33462485 | European | 14306 | 5691265 |
|  | ebi-a-GCST90016967 | genus Anaerotruncus id.2055 | 2021 | 33462485 | European | 14306 | 5646976 |
|  | ebi-a-GCST90016972 | genus Blautia id.1993 | 2021 | 33462485 | European | 14306 | 335714 |
|  | ebi-a-GCST90016973 | genus Butyricicoccus id.2056 | 2021 | 33462485 | European | 14306 | 5648432 |
|  | ebi-a-GCST90016976 | genus Candidatus Soleaferrea id.11351 | 2021 | 33462485 | European | 14306 | 5372145 |
|  | ebi-a-GCST90016977 | genus Catenibacterium id.2154 | 2021 | 33462485 | European | 14306 | 4089327 |
|  | ebi-a-GCST90016979 | genus Clostridium innocuum group id.14398 | 2021 | 33462485 | European | 14306 | 5231958 |
|  | ebi-a-GCST90016982 | genus Coprobacter id.950 | 2021 | 33462485 | European | 14306 | 5394658 |
|  | ebi-a-GCST90016983 | genus Coprococcus1 id.11302 | 2021 | 33462485 | European | 14306 | 5649452 |
|  | ebi-a-GCST90016986 | genus Defluviitaleaceae UCG011 id.11288 | 2021 | 33462485 | European | 14306 | 5440169 |
|  | ebi-a-GCST90016989 | genus Dorea id.1998 | 2021 | 33462485 | European | 14306 | 5702812 |
|  | ebi-a-GCST90016992 | genus Enterorhabdus id.821 | 2021 | 33462485 | European | 14306 | 5416385 |
|  | ebi-a-GCST90016995 | genus Escherichia Shigella id.3505 | 2021 | 33462485 | European | 14306 | 5537082 |
|  | ebi-a-GCST90016996 | genus Eubacterium brachy group id.11297 | 2021 | 33462485 | European | 14306 | 5221253 |
|  | ebi-a-GCST90016999 | genus Eubacterium fissicatena group id.14374 | 2021 | 33462485 | European | 14306 | 5159588 |
|  | ebi-a-GCST90017000 | genus Eubacterium hallii group id.11339 | 2021 | 33462485 | European | 14306 | 5686593 |
|  | ebi-a-GCST90017001 | genus Eubacterium nodatum group id.11298 | 2021 | 33462485 | European | 14306 | 5050598 |
|  | ebi-a-GCST90017003 | genus Eubacterium rectale group id.14375 | 2021 | 33462485 | European | 14306 | 5708381 |
|  | ebi-a-GCST90017004 | genus Eubacterium ruminantium group id.11341 | 2021 | 33462485 | European | 14306 | 5427661 |
|  | ebi-a-GCST90017007 | genus Faecalibacterium id.2058 | 2021 | 33462485 | European | 14306 | 5720140 |
|  | ebi-a-GCST90017008 | genus Family XIII AD3011 group id.11294 | 2021 | 33462485 | European | 14306 | 5577002 |
|  | ebi-a-GCST90017010 | genus Flavonifractor id.2060 | 2021 | 33462485 | European | 14306 | 5510045 |
|  | ebi-a-GCST90017013 | genus Haemophilus id.3699 | 2021 | 33462485 | European | 14306 | 5481944 |
|  | ebi-a-GCST90017014 | genus Holdemanella id.11394 | 2021 | 33462485 | European | 14306 | 5421763 |
|  | ebi-a-GCST90017017 | genus Hungatella id.11307 | 2021 | 33462485 | European | 14306 | 5211608 |
|  | ebi-a-GCST90017020 | genus Lachnoclostridium id.11309 | 2021 | 33462485 | European | 14306 | 5714724 |
|  | ebi-a-GCST90017023 | genus Lachnospiraceae ND3007 group id.11318 | 2021 | 33462485 | European | 14306 | 5629458 |
|  | ebi-a-GCST90017026 | genus Lachnospiraceae UCG004 id.11325 | 2021 | 33462485 | European | 14306 | 5633244 |
|  | ebi-a-GCST90017027 | genus Lachnospiraceae UCG008 id.11329 | 2021 | 33462485 | European | 14306 | 5401028 |
|  | ebi-a-GCST90017030 | genus Lactobacillus id.1838 | 2021 | 33462485 | European | 14306 | 5398287 |
|  | ebi-a-GCST90017032 | genus Marvinbryantia id.2006 | 2021 | 33462485 | European | 14306 | 5507087 |
|  | ebi-a-GCST90017033 | genus Methanobrevibacter id.124 | 2021 | 33462485 | European | 14306 | 5092705 |
|  | ebi-a-GCST90017036 | genus Oscillibacter id.2064 | 2021 | 33462485 | European | 14306 | 5450090 |
|  | ebi-a-GCST90017037 | genus Oscillospira id.2065 | 2021 | 33462485 | European | 14306 | 5487907 |
|  | ebi-a-GCST90017042 | genus Peptococcus id.2038 | 2021 | 33462485 | European | 14306 | 5350051 |
|  | ebi-a-GCST90017043 | genus Phascolarctobacterium id.2169 | 2021 | 33462485 | European | 14306 | 5509695 |
|  | ebi-a-GCST90017046 | genus Rikenellaceae RC9 gut group id.11192 | 2021 | 33462485 | European | 14306 | 4945903 |
|  | ebi-a-GCST90017049 | genus Ruminiclostridium5 id.11356 | 2021 | 33462485 | European | 14306 | 5653350 |
|  | ebi-a-GCST90017052 | genus Ruminococcaceae NK4A214 group id.11359 | 2021 | 33462485 | European | 14306 | 5616365 |
|  | ebi-a-GCST90017053 | genus Ruminococcaceae UCG002 id.11361 | 2021 | 33462485 | European | 14306 | 5694947 |
|  | ebi-a-GCST90017055 | genus Ruminococcaceae UCG004 id.11363 | 2021 | 33462485 | European | 14306 | 5478416 |
|  | ebi-a-GCST90017056 | genus Ruminococcaceae UCG005 id.11364 | 2021 | 33462485 | European | 14306 | 5638483 |
|  | ebi-a-GCST90017059 | genus Ruminococcaceae UCG011 id.11369 | 2021 | 33462485 | European | 14306 | 5176480 |
|  | ebi-a-GCST90017065 | genus Ruminococcus gnavus group id.14377 | 2021 | 33462485 | European | 14306 | 5381061 |
|  | ebi-a-GCST90017066 | genus Ruminococcus torques group id.14378 | 2021 | 33462485 | European | 14306 | 5709224 |
|  | ebi-a-GCST90017062 | genus Ruminococcus1 id.11374 | 2021 | 33462485 | European | 14306 | 5653204 |
|  | ebi-a-GCST90017069 | genus Slackia id.826 | 2021 | 33462485 | European | 14306 | 5369110 |
|  | ebi-a-GCST90017071 | genus Subdoligranulum id.2071 | 2021 | 33462485 | European | 14306 | 5708796 |
|  | ebi-a-GCST90017072 | genus Sutterella id.2897 | 2021 | 33462485 | European | 14306 | 5586151 |
|  | ebi-a-GCST90017075 | genus Tyzzerella3 id.11336 | 2021 | 33462485 | European | 14306 | 5326934 |
|  | ebi-a-GCST90017088 | genus Veillonella id.2199 | 2021 | 33462485 | European | 14306 | 5486191 |
|  | ebi-a-GCST90017089 | genus Victivallis id.2257 | 2021 | 33462485 | Mixed | 14306 | 4291501 |
|  | ebi-a-GCST90017078 | unknown genus id.1000005473 | 2021 | 33462485 | European | 14306 | 5488779 |
|  | ebi-a-GCST90017079 | unknown genus id.1000005480 | 2021 | 33462485 | European | 14306 | 5432872 |
|  | ebi-a-GCST90017081 | unknown genus id.1869 | 2021 | 33462485 | European | 14306 | 5493572 |
|  | ebi-a-GCST90017082 | unknown genus id.2002 | 2021 | 33462485 | European | 14306 | 5485285 |
|  | ebi-a-GCST90017084 | unknown genus id.2072 | 2021 | 33462485 | European | 14306 | 5546384 |
|  | ebi-a-GCST90017085 | unknown genus id.2756 | 2021 | 33462485 | European | 14306 | 5443849 |
|  | ebi-a-GCST90017091 | order Bacillales id.1675 | 2021 | 33462485 | European | 14306 | 4738158 |
|  | ebi-a-GCST90017094 | order Burkholderiales id.2875 | 2021 | 33462485 | European | 14306 | 5707170 |
|  | ebi-a-GCST90017095 | order Clostridiales id.1864 | 2021 | 33462485 | European | 14306 | 5729268 |
|  | ebi-a-GCST90017098 | order Enterobacteriales id.3469 | 2021 | 33462485 | European | 14306 | 5598485 |
|  | ebi-a-GCST90017100 | order Gastranaerophilales id.1592 | 2021 | 33462485 | European | 14306 | 5355169 |
|  | ebi-a-GCST90017103 | order Mollicutes RF9 id.11580 | 2021 | 33462485 | European | 14306 | 5488779 |
|  | ebi-a-GCST90017104 | order NB1n id.3954 | 2021 | 33462485 | European | 14306 | 5316331 |
|  | ebi-a-GCST90017106 | order Rhodospirillales id.2668 | 2021 | 33462485 | European | 14306 | 5447392 |
|  | ebi-a-GCST90017107 | order Selenomonadales id.2166 | 2021 | 33462485 | European | 14306 | 5721008 |
| PHYLUM | ebi-a-GCST90017110 | phylum Actinobacteria id.401 | 2021 | 33462485 | European | 14306 | 5706452 |
|  | ebi-a-GCST90017113 | phylum Euryarchaeota id.56 | 2021 | 33462485 | European | 14306 | 5165248 |
|  | ebi-a-GCST90017116 | phylum Proteobacteria id.2376 | 2021 | 33462485 | European | 14306 | 5728442 |
|  | ebi-a-GCST90017117 | phylum Tenericutes id.3920 | 2021 | 33462485 | European | 14306 | 5510407 |

| Supplementary Table 2: All IVs used for MR analysis | | | | | | | | | | | | | |
| --- | --- | --- | --- | --- | --- | --- | --- | --- | --- | --- | --- | --- | --- |
| id | genus | SNP | effect_allele | other_allele | eaf | exposure | | | outcome | | | R² | F |
|  |  |  |  |  |  | beta | se | pval | beta | se | pval |  |  |
| ebi-a-GCST90016908 | Gut microbiota abundance (class Actinobacteria id.419) | rs10841473 | G | C | 0.2704 | -0.0583765 | 0.0123353 | 2.59E-06 | -0.00428621 | 0.0177846 | 0.80955 | 0.001344614 | 24.69073021 |
|  |  | rs11655079 | T | C | 0.2495 | -0.0560674 | 0.0124286 | 5.93E-06 | 0.0195504 | 0.0192009 | 0.308581 | 0.001177259 | 21.61402369 |
|  |  | rs11745923 | G | T | 0.3966 | 0.0563759 | 0.0115529 | 1.58E-06 | 0.0105362 | 0.0153114 | 0.491373 | 0.00152116 | 27.93753538 |
|  |  | rs12049045 | A | G | 0.3817 | 0.0510452 | 0.0114325 | 8.63E-06 | 0.00476858 | 0.0153806 | 0.756531 | 0.001229876 | 22.58123274 |
|  |  | rs12899991 | T | A | 0.84 | -0.0719923 | 0.0154661 | 4.03E-06 | -0.0452272 | 0.0174001 | 0.00934265 | 0.001393161 | 25.58343139 |
|  |  | rs134366 | A | G | 0.9404 | -0.111877 | 0.023501 | 1.50E-06 | -0.0166347 | 0.0292878 | 0.570053 | 0.001403041 | 25.76512357 |
|  |  | rs1376754 | G | A | 0.4702 | 0.0509242 | 0.0112503 | 6.71E-06 | -0.00601791 | 0.0149758 | 0.687801 | 0.001292031 | 23.72392038 |
|  |  | rs1515761 | C | T | 0.8668 | -0.0762317 | 0.0169884 | 4.96E-06 | 0.0386635 | 0.0285012 | 0.174922 | 0.001341913 | 24.64106521 |
|  |  | rs182549 | T | C | 0.5089 | -0.111489 | 0.012066 | 3.79E-20 | -0.022952 | 0.015417 | 0.136555 | 0.006212929 | 114.6449811 |
|  |  | rs4945008 | G | A | 0.6998 | -0.0540741 | 0.0121393 | 5.39E-06 | 0.0285119 | 0.0153362 | 0.0630101 | 0.001228551 | 22.55688216 |
|  |  | rs6660520 | G | A | 0.7853 | -0.0711483 | 0.0134495 | 1.11E-07 | 0.00560764 | 0.0168293 | 0.738977 | 0.001706973 | 31.35599768 |
|  |  | rs72767435 | T | C | 0.0676 | -0.126352 | 0.0273626 | 2.57E-06 | -0.00476303 | 0.032842 | 0.884688 | 0.002012534 | 36.98027013 |
|  |  | rs7322849 | T | C | 0.0964 | 0.0944467 | 0.0192976 | 6.21E-07 | -0.027651 | 0.0261122 | 0.289632 | 0.001554021 | 28.54198832 |
|  |  | rs7700479 | T | A | 0.0795 | -0.0769653 | 0.0173933 | 8.62E-06 | 0.00509064 | 0.0242509 | 0.833733 | 0.000866984 | 15.91253999 |
|  |  | rs80083040 | T | G | 0.0517 | 0.155784 | 0.0348017 | 8.62E-06 | -0.0754834 | 0.0361527 | 0.036807 | 0.002379644 | 43.74200212 |
|  |  | rs857444 | C | T | 0.3608 | 0.0509553 | 0.011539 | 8.92E-06 | 0.0187018 | 0.0154955 | 0.227463 | 0.001197601 | 21.98793315 |
|  |  | rs961091 | G | A | 0.4334 | 0.0501255 | 0.011255 | 8.68E-06 | 0.030489 | 0.0155099 | 0.0493253 | 0.001233994 | 22.6569332 |
| ebi-a-GCST90016909 | Gut microbiota abundance (class Alphaproteobacteria id.2379) | rs12977163 | G | C | 0.3678 | 0.0690234 | 0.0153829 | 7.14E-06 | -0.0189016 | 0.0153839 | 0.219199 | 0.002215588 | 40.71966176 |
|  |  | rs140912403 | C | T | 0.0646 | -0.160677 | 0.0317708 | 6.20E-07 | 0.0322757 | 0.0319171 | 0.311903 | 0.003120091 | 57.39531361 |
|  |  | rs17061716 | G | C | 0.1372 | 0.0927341 | 0.0207569 | 9.33E-06 | 0.00351153 | 0.0205246 | 0.864154 | 0.002035978 | 37.41194181 |
|  |  | rs34569731 | G | A | 0.665 | -0.0705145 | 0.0157564 | 7.38E-06 | -0.00826958 | 0.0155429 | 0.594692 | 0.002215406 | 40.71631671 |
|  |  | rs62285697 | C | T | 0.1998 | 0.0805256 | 0.0180912 | 9.76E-06 | 0.0053995 | 0.0173786 | 0.75603 | 0.002073442 | 38.10178774 |
|  |  | rs76784716 | A | G | 0.0716 | 0.133271 | 0.0267738 | 5.09E-07 | 0.00635328 | 0.0236345 | 0.788074 | 0.002361291 | 43.40383854 |
|  |  | rs7960664 | A | G | 0.8489 | -0.0969489 | 0.0215149 | 8.84E-06 | 0.0234915 | 0.0266644 | 0.378316 | 0.00241122 | 44.3238195 |
|  |  | rs9813022 | A | G | 0.3022 | -0.0751277 | 0.0153867 | 1.05E-06 | 0.0149184 | 0.0154449 | 0.334088 | 0.002380431 | 43.75650403 |
| ebi-a-GCST90016911 | Gut microbiota abundance (class Bacteroidia id.912) | rs11146701 | A | G | 0.4254 | 0.0473596 | 0.010541 | 7.08E-06 | -0.0102332 | 0.0157448 | 0.51573 | 0.001096501 | 20.12971325 |
|  |  | rs13291169 | C | G | 0.16 | 0.0689676 | 0.0148765 | 3.75E-06 | -0.0148227 | 0.0215657 | 0.491875 | 0.001278555 | 23.47616126 |
|  |  | rs17343978 | A | C | 0.2604 | -0.0552057 | 0.0120278 | 8.36E-06 | -0.00396946 | 0.0185028 | 0.830131 | 0.001173912 | 21.55250784 |
|  |  | rs2032750 | C | T | 0.5487 | 0.0508393 | 0.0106807 | 1.92E-06 | 0.00901216 | 0.0150186 | 0.548461 | 0.001280057 | 23.50377711 |
|  |  | rs2363574 | T | C | 0.9682 | 0.222741 | 0.0508055 | 9.93E-06 | 0.0224635 | 0.0392957 | 0.567556 | 0.00305508 | 56.19573135 |
|  |  | rs4916508 | A | G | 0.5646 | 0.0466729 | 0.0105312 | 8.47E-06 | 0.0247486 | 0.0151277 | 0.101845 | 0.001070999 | 19.66102763 |
|  |  | rs55773148 | G | A | 0.0527 | -0.121514 | 0.0236759 | 3.90E-07 | -0.0480929 | 0.033121 | 0.146492 | 0.001474283 | 27.07531365 |
|  |  | rs62531359 | T | G | 0.1451 | 0.0655728 | 0.0149891 | 9.09E-06 | -0.0264494 | 0.0195598 | 0.176302 | 0.001066744 | 19.58284017 |
|  |  | rs62575403 | C | T | 0.0437 | 0.140055 | 0.0311128 | 7.06E-06 | 0.0035879 | 0.0370022 | 0.922755 | 0.001639468 | 30.11392667 |
|  |  | rs72706335 | T | C | 0.0656 | -0.22241 | 0.0493458 | 7.66E-06 | 0.0167974 | 0.0482513 | 0.727748 | 0.006064225 | 111.8842437 |
|  |  | rs73975615 | G | A | 0.0109 | -0.207018 | 0.0442633 | 1.22E-06 | -0.22653 | 0.0823904 | 0.00596912 | 0.000924087 | 16.96158341 |
|  |  | rs7546249 | A | T | 0.7545 | 0.0567113 | 0.0118386 | 1.55E-06 | 0.0172464 | 0.0163851 | 0.292538 | 0.001191461 | 21.8750807 |
|  |  | rs7631304 | G | A | 0.17 | -0.0645771 | 0.0132957 | 8.37E-07 | -0.01941 | 0.0211645 | 0.35909 | 0.001176831 | 21.60615294 |
|  |  | rs79585701 | A | C | 0.1292 | 0.0646823 | 0.0149628 | 9.99E-06 | -0.0462634 | 0.0214411 | 0.0309514 | 0.000941417 | 17.27996472 |
|  |  | rs929878 | T | C | 0.7555 | 0.0548532 | 0.012153 | 4.73E-06 | 0.0111885 | 0.018311 | 0.541183 | 0.001111597 | 20.40714553 |
| ebi-a-GCST90016914 | Gut microbiota abundance (class Coriobacteriia id.809) | rs11073596 | G | T | 0.66 | -0.0510212 | 0.0114339 | 8.14E-06 | -0.0126817 | 0.015483 | 0.412746 | 0.001168299 | 21.44933524 |
|  |  | rs11250875 | T | C | 0.2087 | 0.06075 | 0.0130939 | 4.83E-06 | 0.022352 | 0.018169 | 0.218611 | 0.001218951 | 22.38040029 |
|  |  | rs11656361 | A | C | 0.1292 | 0.0773006 | 0.017553 | 8.02E-06 | -0.00819402 | 0.019405 | 0.672833 | 0.001344549 | 24.6895373 |
|  |  | rs12974142 | G | A | 0.1083 | 0.0789796 | 0.0177205 | 8.51E-06 | -0.0166416 | 0.0292011 | 0.568747 | 0.001204778 | 22.11987106 |
|  |  | rs13307134 | T | C | 0.7644 | -0.0565686 | 0.0126265 | 7.80E-06 | -0.00301417 | 0.0200982 | 0.880787 | 0.001152595 | 21.1606812 |
|  |  | rs1397793 | A | G | 0.6143 | 0.0498855 | 0.0112446 | 9.77E-06 | -0.0112868 | 0.0164043 | 0.49143 | 0.001179258 | 21.65076393 |
|  |  | rs1816223 | G | A | 0.7893 | 0.0586265 | 0.0129006 | 4.84E-06 | -0.00901566 | 0.0187172 | 0.630035 | 0.001143206 | 20.98810894 |
|  |  | rs240104 | T | C | 0.2445 | -0.0603429 | 0.0126849 | 1.52E-06 | -0.0057517 | 0.0166576 | 0.729876 | 0.001345227 | 24.70200869 |
|  |  | rs2442778 | A | G | 0.9543 | 0.116408 | 0.0258545 | 9.03E-06 | 0.0197925 | 0.0338791 | 0.559079 | 0.001181944 | 21.70013115 |
|  |  | rs3025411 | A | G | 0.1044 | 0.0926543 | 0.0209468 | 8.27E-06 | 0.0368885 | 0.0244324 | 0.131089 | 0.001605372 | 29.48665244 |
|  |  | rs34739816 | G | T | 0.0815 | 0.0965017 | 0.0207666 | 3.88E-06 | 0.0491239 | 0.0319262 | 0.123885 | 0.001394237 | 25.60322033 |
|  |  | rs67561917 | A | G | 0.1421 | -0.0714367 | 0.0154113 | 5.39E-06 | 0.00563572 | 0.0194232 | 0.771699 | 0.001244238 | 22.84526396 |
|  |  | rs719099 | A | G | 0.1362 | 0.0778402 | 0.0155815 | 5.43E-07 | -0.0554344 | 0.0250969 | 0.0271875 | 0.0014257 | 26.1818164 |
|  |  | rs76779974 | C | G | 0.1123 | 0.0774888 | 0.0171877 | 5.63E-06 | 0.0147221 | 0.0257109 | 0.566916 | 0.001197165 | 21.97991681 |
|  |  | rs80046645 | C | G | 0.0408 | 0.25584 | 0.0561995 | 4.66E-06 | 0.0193247 | 0.0383843 | 0.614646 | 0.00512314 | 94.4319288 |
|  |  | rs8010111 | A | G | 0.9354 | 0.103377 | 0.0229286 | 6.90E-06 | -0.0125111 | 0.0271903 | 0.645422 | 0.00129154 | 23.71488201 |
| ebi-a-GCST90016915 | Gut microbiota abundance (class Deltaproteobacteria id.3087) | rs1035691 | G | A | 0.662 | 0.055176 | 0.0121602 | 9.65E-06 | -0.00918445 | 0.0153375 | 0.549292 | 0.001362401 | 25.0178029 |
|  |  | rs11599763 | C | T | 0.6103 | 0.0544219 | 0.0117397 | 3.94E-06 | 0.00224988 | 0.0153461 | 0.88344 | 0.001408806 | 25.87113057 |
|  |  | rs16851319 | G | C | 0.1869 | -0.0705516 | 0.0150849 | 5.68E-06 | 0.00778671 | 0.0198029 | 0.694164 | 0.001512854 | 27.78474931 |
|  |  | rs17084793 | G | A | 0.161 | -0.0710762 | 0.015953 | 5.69E-06 | -0.032265 | 0.0212576 | 0.129062 | 0.001364791 | 25.06174626 |
|  |  | rs17791387 | A | G | 0.1412 | -0.0735881 | 0.0154246 | 1.60E-06 | -0.0266306 | 0.0255078 | 0.296476 | 0.001313324 | 24.11540836 |
|  |  | rs2692012 | G | A | 0.9364 | -0.110335 | 0.0253336 | 3.14E-06 | -0.0168675 | 0.0328869 | 0.608026 | 0.001450024 | 26.62914839 |
|  |  | rs2838334 | G | A | 0.3231 | 0.0561722 | 0.0124124 | 5.45E-06 | 0.0095013 | 0.015705 | 0.545189 | 0.001380176 | 25.34463959 |
|  |  | rs3935584 | C | T | 0.496 | -0.0523484 | 0.011566 | 7.50E-06 | -0.00471038 | 0.0150215 | 0.753843 | 0.00137009 | 25.15917708 |
|  |  | rs4506934 | C | T | 0.1123 | -0.0936514 | 0.02012 | 3.59E-06 | 0.00581981 | 0.0232425 | 0.802282 | 0.001748657 | 32.12303604 |
|  |  | rs55744759 | A | G | 0.1163 | -0.0779693 | 0.0170738 | 7.31E-06 | -0.0265751 | 0.0236051 | 0.260241 | 0.001249574 | 22.94334979 |
|  |  | rs6058181 | C | T | 0.1233 | 0.0825716 | 0.0165958 | 3.40E-07 | 0.0136931 | 0.0202432 | 0.498768 | 0.001474027 | 27.07061245 |
|  |  | rs62020470 | A | G | 0.2594 | -0.0585411 | 0.0129358 | 4.85E-06 | 0.0132957 | 0.0195083 | 0.495528 | 0.001316756 | 24.17851578 |
|  |  | rs9928243 | C | A | 0.4254 | -0.0538457 | 0.0117718 | 5.02E-06 | -0.00813879 | 0.014998 | 0.587365 | 0.001417409 | 26.02933874 |
| ebi-a-GCST90016918 | Gut microbiota abundance (class Lentisphaeria id.2250) | rs1002941 | A | G | 0.7207 | -0.105025 | 0.0233484 | 8.15E-06 | 0.00934795 | 0.0174008 | 0.591119 | 0.004440592 | 81.79478615 |
|  |  | rs11770843 | C | T | 0.2594 | 0.109431 | 0.0234879 | 1.91E-06 | 0.00529519 | 0.0159982 | 0.740653 | 0.004601129 | 84.76552096 |
|  |  | rs17114848 | G | A | 0.1093 | 0.152377 | 0.0324332 | 4.06E-06 | 0.0233436 | 0.0252949 | 0.35608 | 0.004520854 | 83.27991044 |
|  |  | rs2031282 | A | G | 0.17 | 0.122368 | 0.0270329 | 4.38E-06 | -0.0234506 | 0.0198117 | 0.236542 | 0.004225642 | 77.81866268 |
|  |  | rs2731834 | G | C | 0.7763 | -0.109438 | 0.023693 | 4.24E-06 | 0.00387909 | 0.017498 | 0.824557 | 0.004159699 | 76.59918194 |
|  |  | rs2825714 | A | G | 0.1571 | -0.13741 | 0.0289246 | 1.72E-06 | 0.0380424 | 0.0199626 | 0.0566918 | 0.005000563 | 92.16118436 |
|  |  | rs62570196 | C | T | 0.0686 | -0.21635 | 0.0439866 | 1.08E-06 | -0.0289432 | 0.0371324 | 0.435709 | 0.005981418 | 110.3472741 |
|  |  | rs72640280 | A | G | 0.0537 | 0.220207 | 0.0486196 | 5.18E-06 | -0.0517948 | 0.0325869 | 0.111961 | 0.00492828 | 90.82239428 |
|  |  | rs73113483 | T | A | 0.1233 | -0.131217 | 0.0288713 | 8.66E-06 | 0.0175423 | 0.0228302 | 0.44226 | 0.003722411 | 68.51661432 |
|  |  | rs77599476 | A | G | 0.0656 | 0.230292 | 0.0480168 | 1.86E-06 | 0.0127411 | 0.0329627 | 0.699104 | 0.006501662 | 120.0077218 |
| ebi-a-GCST90016920 | Gut microbiota abundance (class Methanobacteria id.119) | rs10202904 | G | T | 0.5328 | 0.121754 | 0.0235357 | 3.01E-07 | 0.00541439 | 0.0152871 | 0.723204 | 0.007380122 | 136.3428985 |
|  |  | rs10424197 | A | G | 0.675 | 0.111274 | 0.0247517 | 9.28E-06 | -0.0166304 | 0.0172857 | 0.336004 | 0.00543256 | 100.166445 |
|  |  | rs11018665 | A | T | 0.341 | 0.111465 | 0.0250516 | 6.52E-06 | -0.0129026 | 0.017481 | 0.460459 | 0.005584018 | 102.9747397 |
|  |  | rs12825290 | C | G | 0.0606 | -0.216781 | 0.0493496 | 6.08E-06 | 0.0251756 | 0.0314588 | 0.423553 | 0.005350515 | 98.64555322 |
|  |  | rs4257531 | G | A | 0.1143 | 0.164498 | 0.0364959 | 7.44E-06 | -0.00082383 | 0.0245215 | 0.973199 | 0.005478783 | 101.0234116 |
|  |  | rs6508769 | C | T | 0.835 | -0.153519 | 0.0344519 | 8.23E-06 | 0.0221915 | 0.0211458 | 0.293969 | 0.006494185 | 119.8688217 |
|  |  | rs6776814 | T | C | 0.0895 | -0.199566 | 0.0411825 | 1.63E-06 | -0.0645808 | 0.0528799 | 0.221983 | 0.006490917 | 119.8081089 |
|  |  | rs73068003 | G | T | 0.1223 | -0.15814 | 0.0351803 | 8.45E-06 | 0.0111615 | 0.0252916 | 0.658986 | 0.005368909 | 98.9864975 |
|  |  | rs73457410 | A | G | 0.0666 | 0.21534 | 0.0436695 | 1.41E-06 | -0.0085037 | 0.0306354 | 0.781336 | 0.005765294 | 106.3370206 |
|  |  | rs75208022 | C | T | 0.0765 | -0.227245 | 0.048763 | 5.92E-06 | -0.0155913 | 0.0255498 | 0.541708 | 0.007296541 | 134.7874436 |
|  |  | rs894996 | C | A | 0.0716 | 0.216999 | 0.0449076 | 1.88E-06 | -0.0376031 | 0.0292061 | 0.197917 | 0.006260278 | 115.5241903 |
| ebi-a-GCST90016921 | Gut microbiota abundance (class Mollicutes id.3920) | rs10108398 | G | A | 0.2913 | 0.0769142 | 0.0153953 | 1.09E-06 | -0.0112563 | 0.0167303 | 0.501067 | 0.002442564 | 44.90141482 |
|  |  | rs11890098 | A | G | 0.2555 | 0.074438 | 0.0153389 | 9.57E-07 | -0.0173315 | 0.0167206 | 0.299951 | 0.002108021 | 38.73855625 |
|  |  | rs12566890 | T | G | 0.0954 | -0.101147 | 0.0230978 | 3.65E-06 | 0.0279033 | 0.0222866 | 0.210563 | 0.001765798 | 32.43847946 |
|  |  | rs17214486 | C | A | 0.4016 | 0.06099 | 0.0135623 | 6.61E-06 | -0.0220373 | 0.0160201 | 0.168945 | 0.001787856 | 32.84442557 |
|  |  | rs2464826 | A | C | 0.1262 | 0.0944239 | 0.0211806 | 8.39E-06 | -0.00940638 | 0.0236645 | 0.691006 | 0.00196637 | 36.1303403 |
|  |  | rs28537087 | G | A | 0.164 | 0.0820873 | 0.0188312 | 8.07E-06 | -0.00937581 | 0.0174352 | 0.590749 | 0.001847703 | 33.94589169 |
|  |  | rs3768491 | G | A | 0.7137 | 0.0681052 | 0.0149061 | 4.23E-06 | -0.00487214 | 0.0164616 | 0.767252 | 0.001895517 | 34.82599615 |
|  |  | rs4885016 | C | T | 0.8429 | 0.0819606 | 0.0181628 | 7.27E-06 | 0.0360183 | 0.0220069 | 0.101697 | 0.001779068 | 32.68268953 |
|  |  | rs6043847 | T | C | 0.0696 | -0.114937 | 0.0248606 | 4.55E-06 | -0.00375842 | 0.0319114 | 0.906245 | 0.001710916 | 31.4285466 |
|  |  | rs72901605 | T | C | 0.1859 | -0.0841852 | 0.0178119 | 3.26E-06 | -0.0451736 | 0.023464 | 0.0542001 | 0.002145155 | 39.42241579 |
|  |  | rs74603314 | T | C | 0.0457 | 0.221639 | 0.0462918 | 1.56E-06 | -0.0621365 | 0.0382965 | 0.104694 | 0.00428473 | 78.91149744 |
|  |  | rs78169027 | A | G | 0.1083 | -0.108283 | 0.0237289 | 5.88E-06 | -0.0391958 | 0.0313705 | 0.211502 | 0.002264633 | 41.62309585 |
| ebi-a-GCST90016924 | Gut microbiota abundance (family Acidaminococcaceae id.2166) | rs262812 | T | C | 0.2664 | -0.065675 | 0.0142257 | 3.25E-06 | 0.0210439 | 0.0163152 | 0.197109 | 0.001685869 | 30.96766437 |
|  |  | rs2933324 | G | A | 0.7465 | 0.0662557 | 0.0140257 | 2.24E-06 | -0.00593082 | 0.017875 | 0.740045 | 0.001661438 | 30.5181629 |
|  |  | rs45497800 | T | C | 0.0825 | -0.117863 | 0.0257351 | 5.86E-06 | -0.0269436 | 0.0214644 | 0.20938 | 0.002103028 | 38.64659739 |
|  |  | rs6427992 | C | G | 0.6074 | 0.0595857 | 0.01296 | 4.24E-06 | -0.0373246 | 0.0154869 | 0.0159492 | 0.001693321 | 31.10478196 |
|  |  | rs6589457 | G | A | 0.9453 | -0.165911 | 0.0349516 | 2.32E-06 | 0.060669 | 0.0345953 | 0.0794859 | 0.002846671 | 52.3512875 |
|  |  | rs6923842 | T | C | 0.169 | -0.0796106 | 0.0169192 | 2.21E-06 | -0.0201822 | 0.0235181 | 0.390806 | 0.001780162 | 32.70282648 |
|  |  | rs74540770 | G | A | 0.0646 | -0.108969 | 0.0243533 | 7.09E-06 | 0.00815377 | 0.0277614 | 0.76898 | 0.001435046 | 26.35369156 |
|  |  | rs78702810 | T | C | 0.0467 | -0.143755 | 0.0323244 | 9.16E-06 | -0.0564469 | 0.0244515 | 0.0209701 | 0.001840019 | 33.8044755 |
| ebi-a-GCST90016925 | Gut microbiota abundance (family Actinomycetaceae id.421) | rs2889192 | T | G | 0.7932 | -0.0887537 | 0.0195009 | 3.64E-06 | 0.000394924 | 0.0210648 | 0.985042 | 0.00258426 | 47.51294178 |
|  |  | rs34583783 | G | T | 0.1024 | 0.123756 | 0.026427 | 5.48E-06 | 0.0603456 | 0.0313429 | 0.0541876 | 0.002815434 | 51.7751951 |
|  |  | rs35011108 | A | G | 0.0636 | 0.241826 | 0.0503796 | 1.83E-06 | -0.00895066 | 0.0299121 | 0.764763 | 0.006965535 | 128.6299639 |
|  |  | rs4073240 | G | A | 0.3827 | 0.0747546 | 0.0164684 | 6.05E-06 | -0.0128924 | 0.0153738 | 0.401697 | 0.002640344 | 48.54681592 |
| ebi-a-GCST90016927 | Gut microbiota abundance (family Bacteroidaceae id.917) | rs11585893 | A | G | 0.1481 | -0.0740746 | 0.0147633 | 1.80E-06 | 0.00599408 | 0.0174738 | 0.731574 | 0.001384562 | 25.42529467 |
|  |  | rs13207588 | A | G | 0.2326 | -0.059205 | 0.0131195 | 7.48E-06 | 0.0204746 | 0.0190039 | 0.281307 | 0.001251348 | 22.97597946 |
|  |  | rs1340391 | T | C | 0.1918 | -0.0592004 | 0.0132244 | 6.73E-06 | -0.0313543 | 0.0218332 | 0.150978 | 0.001086543 | 19.94668968 |
|  |  | rs17619981 | T | G | 0.0656 | 0.0880978 | 0.0187002 | 2.69E-06 | -0.041907 | 0.021827 | 0.0548631 | 0.000951474 | 17.46474207 |
|  |  | rs2023437 | T | C | 0.1243 | -0.0782325 | 0.0167634 | 5.02E-06 | 0.00963557 | 0.0227758 | 0.67225 | 0.001332389 | 24.46593935 |
|  |  | rs2366421 | T | A | 0.2336 | -0.0528153 | 0.0117124 | 7.65E-06 | -0.0232883 | 0.0194524 | 0.231232 | 0.000998798 | 18.33427706 |
|  |  | rs28757219 | T | A | 0.1521 | 0.0818398 | 0.0170319 | 1.29E-06 | -0.0885734 | 0.0232757 | 0.000141576 | 0.001727559 | 31.7348062 |
|  |  | rs495004 | C | G | 0.2097 | -0.0607475 | 0.0129865 | 3.42E-06 | -0.0158114 | 0.0178392 | 0.375438 | 0.001223143 | 22.45746468 |
|  |  | rs66710942 | T | C | 0.5845 | -0.0488038 | 0.0107412 | 5.86E-06 | 0.0179229 | 0.0149861 | 0.23171 | 0.001156892 | 21.23965743 |
|  |  | rs6795673 | C | T | 0.4115 | 0.0538565 | 0.0105251 | 3.38E-07 | 0.00364644 | 0.014969 | 0.807542 | 0.001404826 | 25.79794191 |
|  |  | rs9507307 | C | T | 0.1899 | 0.0604456 | 0.0129128 | 2.13E-06 | 0.0145233 | 0.0174346 | 0.404836 | 0.001124147 | 20.63780155 |
| ebi-a-GCST90016928 | Gut microbiota abundance (family Bacteroidales S24 7group id.11173) | rs10872669 | G | A | 0.8926 | 0.123071 | 0.0275665 | 9.49E-06 | -0.0198698 | 0.0260147 | 0.444993 | 0.00290404 | 53.40939153 |
|  |  | rs11135366 | C | G | 0.2863 | 0.0842142 | 0.0183739 | 8.78E-06 | 0.00621061 | 0.0175443 | 0.723341 | 0.002898262 | 53.30282054 |
|  |  | rs12748533 | G | T | 0.2972 | -0.0821108 | 0.0172701 | 2.59E-06 | 0.00986244 | 0.0165929 | 0.552259 | 0.002816509 | 51.79502046 |
|  |  | rs17043785 | T | C | 0.0596 | -0.176187 | 0.0347133 | 5.12E-07 | 0.018799 | 0.0263503 | 0.475584 | 0.003479658 | 64.03278593 |
|  |  | rs1850003 | A | G | 0.3072 | 0.084048 | 0.0177806 | 2.41E-06 | 0.0184336 | 0.0177341 | 0.298599 | 0.003006864 | 55.30617878 |
|  |  | rs61508842 | T | C | 0.1223 | 0.122574 | 0.0272389 | 7.83E-06 | -0.0397513 | 0.026607 | 0.135172 | 0.003225517 | 59.34092683 |
|  |  | rs6831034 | T | A | 0.1978 | -0.0956546 | 0.0205228 | 6.10E-06 | -0.00872804 | 0.0262278 | 0.739302 | 0.002903693 | 53.40298354 |
|  |  | rs738193 | T | C | 0.3549 | 0.0847328 | 0.0165856 | 3.82E-07 | -0.0193929 | 0.0156681 | 0.215815 | 0.003287503 | 60.48507417 |
|  |  | rs78609301 | A | G | 0.2167 | -0.0867065 | 0.0195709 | 7.09E-06 | 0.00222525 | 0.0163298 | 0.891608 | 0.002552233 | 46.92260893 |
|  |  | rs941000 | T | C | 0.5915 | -0.0850266 | 0.0163382 | 3.16E-07 | -0.0171337 | 0.0155648 | 0.270985 | 0.003493707 | 64.29220995 |
| ebi-a-GCST90016931 | Gut microbiota abundance (family Clostridiaceae1 id.1869) | rs10875374 | T | C | 0.5517 | 0.0536759 | 0.0119436 | 8.10E-06 | 0.022907 | 0.0149646 | 0.125832 | 0.001425149 | 26.17168793 |
|  |  | rs12186080 | G | A | 0.161 | 0.0748236 | 0.016246 | 5.34E-06 | 0.0169156 | 0.0200929 | 0.39986 | 0.001512499 | 27.77821707 |
|  |  | rs12341505 | G | A | 0.1113 | 0.0814503 | 0.0179338 | 4.54E-06 | -0.0299715 | 0.0257269 | 0.244025 | 0.001312398 | 24.0983899 |
|  |  | rs12490337 | C | G | 0.2455 | -0.0616944 | 0.013693 | 6.91E-06 | -0.0372985 | 0.0186789 | 0.0458437 | 0.001410043 | 25.89387221 |
|  |  | rs2795528 | G | A | 0.0487 | -0.180969 | 0.0390911 | 3.81E-06 | 0.00134341 | 0.0325871 | 0.967117 | 0.003034484 | 55.81573632 |
|  |  | rs2817172 | C | T | 0.4125 | 0.0563367 | 0.012392 | 5.27E-06 | 0.0122389 | 0.0152727 | 0.422925 | 0.001538313 | 28.25304043 |
|  |  | rs4723021 | T | C | 0.0577 | -0.106376 | 0.0241944 | 7.42E-06 | -0.0257727 | 0.0300634 | 0.391291 | 0.001230502 | 22.59274544 |
|  |  | rs550843 | T | C | 0.1491 | -0.0734787 | 0.0168383 | 7.09E-06 | -0.0192911 | 0.0167143 | 0.248433 | 0.001369964 | 25.15686012 |
|  |  | rs56188186 | A | G | 0.0755 | 0.0967773 | 0.0217471 | 8.24E-06 | 0.0297826 | 0.0350938 | 0.396073 | 0.001307467 | 24.00772632 |
|  |  | rs62397761 | A | G | 0.2863 | 0.0615805 | 0.0136198 | 9.08E-06 | 0.0171083 | 0.0159795 | 0.284331 | 0.001549721 | 28.46288956 |
|  |  | rs881532 | G | A | 0.5229 | 0.0533035 | 0.0119011 | 7.90E-06 | -0.0332862 | 0.0149414 | 0.0258946 | 0.001417652 | 26.03380158 |
| ebi-a-GCST90016934 | Gut microbiota abundance (family Defluviitaleaceae id.1924) | rs112893842 | T | C | 0.0974 | 0.110761 | 0.0232545 | 2.75E-06 | 0.0148179 | 0.0260614 | 0.569643 | 0.002157039 | 39.64129087 |
|  |  | rs1582238 | C | T | 0.6282 | -0.0801923 | 0.0167061 | 1.69E-06 | -0.000712156 | 0.0156324 | 0.963664 | 0.003004019 | 55.25368064 |
|  |  | rs17051335 | C | T | 0.0795 | -0.134314 | 0.0292201 | 4.58E-06 | -0.0347899 | 0.0247313 | 0.159512 | 0.002640362 | 48.54714143 |
|  |  | rs1908593 | T | C | 0.5378 | 0.070223 | 0.0156601 | 7.86E-06 | 0.0171382 | 0.0151752 | 0.258749 | 0.002451543 | 45.0668766 |
|  |  | rs28696126 | T | A | 0.8082 | 0.107106 | 0.0238173 | 5.56E-06 | -0.00434422 | 0.0224426 | 0.846512 | 0.003556518 | 65.45221423 |
|  |  | rs4344384 | T | G | 0.5258 | -0.0708803 | 0.0156121 | 5.86E-06 | 0.0116074 | 0.0149832 | 0.43852 | 0.00250532 | 46.05794973 |
|  |  | rs4677103 | A | G | 0.171 | 0.0977203 | 0.0197033 | 9.42E-07 | -0.0582774 | 0.0195206 | 0.00283178 | 0.002707386 | 49.7828305 |
|  |  | rs540220 | C | T | 0.9404 | 0.123954 | 0.0289814 | 9.48E-06 | -0.0330216 | 0.026315 | 0.209531 | 0.001722305 | 31.63811269 |
|  |  | rs55658617 | T | C | 0.0696 | 0.177314 | 0.0361844 | 1.41E-06 | 0.0146385 | 0.0402815 | 0.716303 | 0.00407188 | 74.97542994 |
|  |  | rs72731813 | C | T | 0.0815 | -0.149751 | 0.0293415 | 2.76E-07 | -0.00468596 | 0.0348772 | 0.893121 | 0.003357424 | 61.77585429 |
|  |  | rs9608282 | T | G | 0.0656 | 0.139014 | 0.0299429 | 4.61E-06 | 0.00850417 | 0.0421103 | 0.839956 | 0.002369102 | 43.54776009 |
|  |  | rs9725395 | A | G | 0.0875 | -0.138357 | 0.0295185 | 3.41E-06 | 0.0091969 | 0.0236063 | 0.696835 | 0.003056843 | 56.22827587 |
| ebi-a-GCST90016937 | Gut microbiota abundance (family Erysipelotrichaceae id.2149) | rs1074800 | G | A | 0.6014 | -0.0492438 | 0.010887 | 6.15E-06 | 0.000496156 | 0.0151705 | 0.97391 | 0.001162609 | 21.34474685 |
|  |  | rs10781552 | C | T | 0.3121 | -0.0552212 | 0.0116074 | 2.33E-06 | -0.0271953 | 0.0166794 | 0.103002 | 0.001309365 | 24.04261736 |
|  |  | rs17530232 | A | G | 0.0765 | 0.10305 | 0.0224648 | 2.79E-06 | -0.0159299 | 0.0332775 | 0.632154 | 0.00150046 | 27.55677701 |
|  |  | rs1884466 | C | T | 0.4513 | -0.0475363 | 0.0106939 | 9.53E-06 | 0.0197784 | 0.0150194 | 0.187886 | 0.001119131 | 20.54562292 |
|  |  | rs2300774 | A | G | 0.5924 | -0.0524152 | 0.0106784 | 8.95E-07 | -0.0108801 | 0.0150411 | 0.46946 | 0.001326764 | 24.36252359 |
|  |  | rs290833 | T | G | 0.3688 | -0.0497374 | 0.0111376 | 8.03E-06 | -0.0130702 | 0.01501 | 0.383882 | 0.001151739 | 21.14494241 |
|  |  | rs35161940 | T | C | 0.1272 | -0.0806041 | 0.0167641 | 1.85E-06 | 0.03931 | 0.0243186 | 0.105995 | 0.001442601 | 26.49262823 |
|  |  | rs4078432 | T | C | 0.8191 | 0.0608929 | 0.0133764 | 4.23E-06 | 0.00428123 | 0.019847 | 0.829212 | 0.001098851 | 20.17289646 |
|  |  | rs56970041 | T | G | 0.1113 | 0.0724145 | 0.0164471 | 5.40E-06 | 0.000470306 | 0.0311033 | 0.987936 | 0.001037365 | 19.04294604 |
|  |  | rs62504403 | C | T | 0.2356 | 0.0681159 | 0.0127883 | 1.12E-07 | 0.012769 | 0.0188635 | 0.49846 | 0.001671179 | 30.69738054 |
|  |  | rs7234058 | T | C | 0.0974 | -0.0945759 | 0.0194091 | 9.12E-07 | -0.0170341 | 0.026048 | 0.513144 | 0.001572698 | 28.88555839 |
|  |  | rs7826267 | G | T | 0.9254 | 0.0839071 | 0.0199131 | 9.28E-06 | 0.00314475 | 0.0304893 | 0.91785 | 0.000972066 | 17.84309047 |
|  |  | rs8003149 | C | T | 0.2823 | 0.0538798 | 0.0116888 | 4.08E-06 | 0.011063 | 0.0158896 | 0.486278 | 0.001176348 | 21.59727312 |
| ebi-a-GCST90016938 | Gut microbiota abundance (family Family XI id.1936) | rs10759623 | C | T | 0.1809 | -0.162115 | 0.032151 | 5.78E-07 | -0.0199761 | 0.0179458 | 0.265651 | 0.007788465 | 143.9459952 |
|  |  | rs11547158 | A | G | 0.1203 | -0.177599 | 0.0372795 | 2.70E-06 | -0.0288195 | 0.0220088 | 0.19038 | 0.006675922 | 123.2458353 |
|  |  | rs17379710 | T | C | 0.4354 | -0.116383 | 0.0252125 | 3.97E-06 | 0.00128668 | 0.0151601 | 0.932363 | 0.00665945 | 122.9397127 |
|  |  | rs2155352 | A | G | 0.2286 | -0.150538 | 0.0302319 | 6.63E-07 | 0.0169866 | 0.0176584 | 0.336071 | 0.007992417 | 147.7457906 |
|  |  | rs2156611 | T | C | 0.492 | -0.112486 | 0.0251099 | 9.43E-06 | 0.0144439 | 0.0149404 | 0.333659 | 0.006324931 | 116.7248521 |
|  |  | rs3733511 | A | G | 0.2942 | 0.128253 | 0.0274718 | 3.39E-06 | -0.0207495 | 0.0163989 | 0.205765 | 0.00683108 | 126.1299523 |
|  |  | rs488164 | G | T | 0.6163 | -0.117998 | 0.025516 | 4.80E-06 | -0.000166114 | 0.0156465 | 0.991529 | 0.006585113 | 121.5582838 |
|  |  | rs6025153 | C | G | 0.6262 | -0.118619 | 0.0261969 | 7.07E-06 | 0.0137302 | 0.0153586 | 0.371337 | 0.006587049 | 121.5942459 |
|  |  | rs697771 | A | G | 0.4324 | -0.117677 | 0.0251491 | 3.19E-06 | -0.0179932 | 0.0150352 | 0.231409 | 0.006797375 | 125.5033602 |
|  |  | rs78222136 | G | C | 0.0805 | 0.224835 | 0.0511672 | 8.41E-06 | -0.0158691 | 0.0264218 | 0.548102 | 0.007483512 | 138.2673646 |
| ebi-a-GCST90016940 | Gut microbiota abundance (family Lachnospiraceae id.1987) | rs10402491 | C | T | 0.1203 | 0.0661701 | 0.014851 | 7.58E-06 | -0.00735402 | 0.0199892 | 0.712948 | 0.000926731 | 17.01016108 |
|  |  | rs11139361 | C | T | 0.6372 | -0.0494463 | 0.0110503 | 4.26E-06 | 0.0123117 | 0.016019 | 0.44215 | 0.001130422 | 20.75314031 |
|  |  | rs112040820 | A | G | 0.2704 | 0.0549584 | 0.0117086 | 2.42E-06 | 0.0111469 | 0.01713 | 0.515223 | 0.001191762 | 21.88061489 |
|  |  | rs11841382 | G | T | 0.0736 | -0.0718128 | 0.017462 | 9.58E-06 | 0.0412731 | 0.0271064 | 0.12785 | 0.000703251 | 12.90528413 |
|  |  | rs11979110 | T | C | 0.4473 | -0.0500924 | 0.0105069 | 1.82E-06 | 0.000464267 | 0.0149731 | 0.975264 | 0.001240686 | 22.77997087 |
|  |  | rs1205443 | A | G | 0.336 | 0.0501415 | 0.0112157 | 7.29E-06 | 0.0254653 | 0.0157459 | 0.105822 | 0.001121843 | 20.59545772 |
|  |  | rs12760724 | A | C | 0.3439 | -0.0484566 | 0.0107934 | 7.27E-06 | -0.00585423 | 0.0159288 | 0.713227 | 0.001059591 | 19.45138242 |
|  |  | rs13005175 | G | A | 0.9115 | -0.0993483 | 0.0218395 | 8.37E-06 | 0.0382613 | 0.0364181 | 0.293438 | 0.001592395 | 29.2479147 |
|  |  | rs2159863 | A | G | 0.1978 | -0.0585831 | 0.0128742 | 3.70E-06 | -0.0140428 | 0.0195135 | 0.471745 | 0.00108914 | 19.99442285 |
|  |  | rs2910921 | C | T | 0.9553 | -0.160302 | 0.035832 | 8.42E-06 | 0.031717 | 0.0448939 | 0.479885 | 0.002194599 | 40.3330715 |
|  |  | rs3127230 | C | T | 0.334 | -0.0503537 | 0.0112201 | 6.20E-06 | 0.00107846 | 0.0163086 | 0.947276 | 0.001128011 | 20.70883185 |
|  |  | rs35524804 | T | C | 0.2674 | -0.0607481 | 0.0125216 | 2.45E-06 | -0.0363609 | 0.0182607 | 0.0464579 | 0.001445852 | 26.55241697 |
|  |  | rs7359994 | C | T | 0.668 | 0.0503142 | 0.0112482 | 5.36E-06 | 0.0107888 | 0.0152219 | 0.478468 | 0.00112286 | 20.61415702 |
|  |  | rs79086868 | T | C | 0.1093 | 0.0776723 | 0.0164472 | 3.01E-06 | -0.0447554 | 0.0247684 | 0.0707685 | 0.001174665 | 21.56633562 |
|  |  | rs959845 | T | C | 0.5547 | 0.0493666 | 0.0107801 | 5.17E-06 | 0.0126294 | 0.0153456 | 0.410507 | 0.001203947 | 22.10458889 |
|  |  | rs9929145 | G | A | 0.0616 | -0.125721 | 0.0245233 | 2.84E-07 | -0.021952 | 0.0346779 | 0.526717 | 0.001827319 | 33.57071951 |
| ebi-a-GCST90016943 | Gut microbiota abundance (family Oxalobacteraceae id.2966) | rs111966731 | T | C | 0.0716 | 0.204031 | 0.0445746 | 4.56E-06 | -0.0264869 | 0.0267929 | 0.322869 | 0.005534399 | 102.0546198 |
|  |  | rs11246212 | C | T | 0.8748 | -0.13607 | 0.0291797 | 4.51E-06 | 0.00908736 | 0.0215504 | 0.673259 | 0.004055719 | 74.67664443 |
|  |  | rs12002250 | A | C | 0.0596 | 0.196145 | 0.044523 | 5.53E-06 | -0.000634662 | 0.0374146 | 0.986466 | 0.004312642 | 79.42776363 |
|  |  | rs12509763 | C | G | 0.1163 | -0.16332 | 0.0360823 | 8.05E-06 | -0.0374172 | 0.0253536 | 0.139995 | 0.005482685 | 101.0957568 |
|  |  | rs1569853 | T | C | 0.1382 | -0.140043 | 0.0281704 | 7.45E-07 | 0.0059721 | 0.0229575 | 0.794758 | 0.004671618 | 86.07022137 |
|  |  | rs17138946 | G | T | 0.0467 | -0.189444 | 0.0430248 | 8.09E-06 | 0.0246011 | 0.0298055 | 0.409152 | 0.003195495 | 58.78684538 |
|  |  | rs36057338 | G | T | 0.0755 | 0.181604 | 0.0398742 | 6.26E-06 | -0.0345599 | 0.0415957 | 0.406058 | 0.004603993 | 84.81853308 |
|  |  | rs4428215 | G | A | 0.2604 | 0.12561 | 0.0230053 | 4.88E-08 | 0.0155808 | 0.0171502 | 0.363618 | 0.006077379 | 112.12842 |
|  |  | rs561239 | A | G | 0.2823 | 0.105525 | 0.0236769 | 7.19E-06 | 0.0249123 | 0.0185407 | 0.179059 | 0.004512264 | 83.12096876 |
|  |  | rs6000536 | C | T | 0.2107 | -0.118383 | 0.0241355 | 7.39E-07 | -0.019569 | 0.0200312 | 0.328605 | 0.004661389 | 85.88086969 |
|  |  | rs62435498 | C | A | 0.0646 | 0.181521 | 0.0400608 | 7.46E-06 | 0.00178344 | 0.0247706 | 0.942603 | 0.003982113 | 73.31594907 |
|  |  | rs736744 | T | C | 0.5845 | -0.105642 | 0.0200648 | 1.49E-07 | 0.0213294 | 0.0151084 | 0.15802 | 0.005420742 | 99.94736279 |
|  |  | rs7993559 | A | C | 0.5378 | -0.0921059 | 0.0201114 | 5.04E-06 | 0.0123683 | 0.015151 | 0.41431 | 0.004217505 | 77.66817793 |
|  |  | rs80330081 | A | C | 0.0656 | -0.18756 | 0.0423688 | 6.64E-06 | -0.0229248 | 0.0244965 | 0.349356 | 0.004312679 | 79.42845306 |
|  |  | rs934049 | G | A | 0.2276 | 0.109751 | 0.0238636 | 4.21E-06 | -0.0176932 | 0.0184133 | 0.336605 | 0.004235079 | 77.99318142 |
| ebi-a-GCST90016944 | Gut microbiota abundance (family Pasteurellaceae id.3689) | rs10965428 | C | A | 0.0795 | -0.119902 | 0.0258219 | 4.29E-06 | 0.0281458 | 0.0325777 | 0.38761 | 0.002104136 | 38.66700348 |
|  |  | rs111582866 | G | A | 0.1014 | -0.113957 | 0.0256403 | 7.07E-06 | 0.0223591 | 0.0265012 | 0.398838 | 0.002366554 | 43.50081041 |
|  |  | rs12050685 | A | G | 0.3598 | -0.0670948 | 0.015239 | 9.19E-06 | 0.0114458 | 0.0165942 | 0.490354 | 0.002073884 | 38.10992815 |
|  |  | rs12191680 | C | G | 0.165 | 0.102214 | 0.0197189 | 3.05E-07 | 0.0414789 | 0.0248326 | 0.0948528 | 0.002878864 | 52.94503381 |
|  |  | rs16970009 | A | G | 0.0437 | 0.187381 | 0.0429572 | 7.32E-06 | -0.114196 | 0.0535348 | 0.0329155 | 0.002934653 | 53.97405393 |
|  |  | rs4822728 | T | C | 0.4374 | 0.0685479 | 0.0149032 | 4.72E-06 | 0.01051 | 0.0149417 | 0.481805 | 0.00231258 | 42.50639596 |
|  |  | rs62568866 | T | A | 0.0994 | -0.118018 | 0.0259206 | 7.23E-06 | -0.00346839 | 0.0242699 | 0.886362 | 0.002493704 | 45.84385672 |
|  |  | rs6972479 | A | G | 0.2425 | -0.0781862 | 0.0175366 | 7.75E-06 | -0.00692529 | 0.0185512 | 0.708921 | 0.00224587 | 41.27746541 |
|  |  | rs72756943 | G | A | 0.0855 | 0.13988 | 0.0303029 | 3.35E-06 | 0.00897154 | 0.0300648 | 0.765394 | 0.003059786 | 56.28257014 |
|  |  | rs73139353 | A | C | 0.0646 | -0.222573 | 0.0484634 | 8.71E-06 | -0.0122419 | 0.0264494 | 0.643475 | 0.005986939 | 110.4497446 |
|  |  | rs731534 | C | G | 0.1362 | -0.0986502 | 0.0216316 | 9.46E-06 | -0.0108144 | 0.0237233 | 0.648494 | 0.002289899 | 42.08853819 |
|  |  | rs76022354 | C | T | 0.0328 | 0.24289 | 0.0500406 | 1.83E-06 | -0.0312682 | 0.0344624 | 0.36424 | 0.003743169 | 68.90013185 |
|  |  | rs78909003 | T | C | 0.0497 | -0.241159 | 0.0498372 | 2.05E-06 | -0.033352 | 0.0329506 | 0.31145 | 0.005493562 | 101.2974311 |
|  |  | rs9382510 | C | T | 0.2455 | -0.0881865 | 0.0169965 | 2.48E-07 | 0.0186942 | 0.0171228 | 0.274932 | 0.002881011 | 52.984633 |
|  |  | rs9895850 | T | C | 0.0497 | -0.176406 | 0.041017 | 9.08E-06 | 0.0129611 | 0.0364378 | 0.722061 | 0.002939502 | 54.06351478 |
|  |  | rs9938097 | C | T | 0.6332 | 0.0710386 | 0.0158024 | 8.23E-06 | -0.0151802 | 0.015366 | 0.323196 | 0.00234417 | 43.08838734 |
| ebi-a-GCST90016947 | Gut microbiota abundance (family Porphyromonadaceae id.943) | rs10119172 | C | G | 0.1511 | -0.0737324 | 0.014259 | 1.32E-07 | 0.0292248 | 0.0196128 | 0.1362 | 0.001394658 | 25.61095765 |
|  |  | rs10762312 | A | G | 0.7386 | 0.052368 | 0.0118814 | 8.70E-06 | -0.0379512 | 0.0161543 | 0.0188092 | 0.001058953 | 19.43967356 |
|  |  | rs10858364 | G | T | 0.2763 | 0.055316 | 0.012085 | 4.31E-06 | 0.0171206 | 0.0175984 | 0.330629 | 0.001223689 | 22.46749865 |
|  |  | rs17065783 | A | G | 0.2237 | -0.059133 | 0.0122235 | 1.79E-06 | 0.0287257 | 0.020444 | 0.159994 | 0.001214466 | 22.29795918 |
|  |  | rs1980561 | A | G | 0.3857 | -0.0485289 | 0.0109284 | 8.95E-06 | 0.00823398 | 0.0149696 | 0.582287 | 0.001115992 | 20.48792391 |
|  |  | rs35233670 | T | C | 0.4414 | -0.0473576 | 0.010602 | 7.91E-06 | -0.0114308 | 0.014945 | 0.444356 | 0.001105968 | 20.30369944 |
|  |  | rs35961441 | A | C | 0.0736 | 0.0915257 | 0.0207306 | 8.37E-06 | -0.0334853 | 0.0389511 | 0.389967 | 0.001142332 | 20.97204762 |
|  |  | rs6953849 | A | G | 0.1471 | 0.0718149 | 0.0150724 | 2.44E-06 | 0.0151963 | 0.0194921 | 0.435619 | 0.001294106 | 23.76206932 |
|  |  | rs7330827 | T | C | 0.0755 | -0.103842 | 0.0237355 | 8.05E-06 | 0.00379264 | 0.0318338 | 0.905165 | 0.001505324 | 27.64624585 |
|  |  | rs864093 | A | C | 0.2565 | -0.0527939 | 0.0117499 | 9.60E-06 | -0.0158385 | 0.0183382 | 0.38776 | 0.00106308 | 19.51550224 |
| ebi-a-GCST90016950 | Gut microbiota abundance (family Rikenellaceae id.967) | rs10217435 | C | T | 0.0875 | -0.0883756 | 0.0197506 | 6.51E-06 | -0.00482013 | 0.0215179 | 0.822753 | 0.001247199 | 22.89969144 |
|  |  | rs10832801 | A | C | 0.2674 | -0.0534763 | 0.0122666 | 7.50E-06 | -0.011541 | 0.0166904 | 0.489268 | 0.00112042 | 20.56931592 |
|  |  | rs1939881 | G | A | 0.0577 | -0.105589 | 0.0206213 | 5.64E-07 | -0.0398263 | 0.0321024 | 0.214753 | 0.001212362 | 22.25928262 |
|  |  | rs2447496 | A | G | 0.7167 | 0.0549203 | 0.0121904 | 6.09E-06 | 0.0047493 | 0.0169768 | 0.779669 | 0.001224841 | 22.48868248 |
|  |  | rs2833282 | G | A | 0.1282 | 0.0711137 | 0.0156966 | 4.31E-06 | -0.0382246 | 0.0209618 | 0.0682213 | 0.001130424 | 20.75317839 |
|  |  | rs36021379 | A | G | 0.1342 | -0.0656005 | 0.0144903 | 7.20E-06 | -0.0153561 | 0.0205215 | 0.454285 | 0.001000033 | 18.35696528 |
|  |  | rs4264350 | T | C | 0.3936 | -0.0526393 | 0.0108555 | 1.35E-06 | -0.00598054 | 0.0150048 | 0.690206 | 0.00132271 | 24.28797364 |
|  |  | rs59663348 | G | A | 0.1948 | 0.0570915 | 0.0125048 | 6.12E-06 | 0.00882102 | 0.017349 | 0.611142 | 0.001022505 | 18.76989684 |
|  |  | rs62532512 | A | C | 0.5169 | 0.0504257 | 0.0107399 | 2.76E-06 | -0.00108025 | 0.0151182 | 0.943037 | 0.001269923 | 23.31746192 |
|  |  | rs67281112 | G | C | 0.2167 | 0.0635814 | 0.0137451 | 3.63E-06 | 0.00900441 | 0.0185989 | 0.628288 | 0.001372389 | 25.2014544 |
|  |  | rs6744030 | C | T | 0.1382 | 0.0696747 | 0.0157103 | 9.32E-06 | -0.0566782 | 0.018139 | 0.00178008 | 0.001156364 | 21.22996142 |
|  |  | rs6837275 | A | G | 0.2535 | 0.0570355 | 0.011887 | 1.45E-06 | 0.0218455 | 0.0163823 | 0.182373 | 0.001231199 | 22.60556043 |
|  |  | rs74474130 | T | G | 0.0656 | 0.137747 | 0.0295688 | 3.61E-06 | 0.00443963 | 0.0404873 | 0.912683 | 0.002326114 | 42.75573007 |
|  |  | rs77885767 | C | T | 0.0477 | -0.156209 | 0.0336482 | 2.85E-06 | -0.085058 | 0.0356691 | 0.0170958 | 0.00221684 | 40.74272398 |
|  |  | rs9389714 | C | T | 0.1789 | -0.0636841 | 0.0143416 | 8.79E-06 | 0.0113525 | 0.0264982 | 0.668342 | 0.001191512 | 21.87601254 |
|  |  | rs9578457 | G | A | 0.0567 | -0.141478 | 0.0315848 | 3.99E-06 | -0.0071575 | 0.0349711 | 0.837831 | 0.002141119 | 39.34808074 |
|  |  | rs9603208 | G | T | 0.1034 | 0.0821173 | 0.0159596 | 1.92E-07 | 0.026891 | 0.0249758 | 0.281622 | 0.001250313 | 22.95693496 |
| ebi-a-GCST90016953 | Gut microbiota abundance (unknown family id.1000001214) | rs11150282 | T | C | 0.3211 | 0.098235 | 0.0197127 | 7.36E-07 | -0.00447128 | 0.0156208 | 0.774695 | 0.00420735 | 77.48036549 |
|  |  | rs113884518 | T | C | 0.0487 | -0.20591 | 0.0455376 | 7.74E-06 | -0.00587112 | 0.0483315 | 0.903314 | 0.003928541 | 72.32572624 |
|  |  | rs1221147 | A | T | 0.8439 | -0.126305 | 0.0279161 | 4.86E-06 | 0.0274885 | 0.0209781 | 0.190079 | 0.004203054 | 77.40092491 |
|  |  | rs16851659 | G | C | 0.3887 | -0.0895168 | 0.0186555 | 1.27E-06 | 0.0199539 | 0.0157364 | 0.204793 | 0.003808097 | 70.09983421 |
|  |  | rs28678345 | T | C | 0.0467 | 0.213083 | 0.0471377 | 8.06E-06 | -0.0329631 | 0.0349631 | 0.345786 | 0.004042724 | 74.43639538 |
|  |  | rs367480 | A | G | 0.5726 | 0.0841649 | 0.0185948 | 7.52E-06 | -0.0206672 | 0.0156938 | 0.187872 | 0.003467192 | 63.80258105 |
|  |  | rs4129395 | G | A | 0.4264 | 0.0904264 | 0.0185253 | 1.22E-06 | -0.009335 | 0.0149468 | 0.532266 | 0.003999879 | 73.64434334 |
|  |  | rs73074665 | A | T | 0.0855 | 0.164631 | 0.0356037 | 3.62E-06 | 0.0687311 | 0.0303448 | 0.023512 | 0.004238411 | 78.05480648 |
|  |  | rs789069 | A | C | 0.172 | -0.10413 | 0.0234459 | 6.50E-06 | 0.00231585 | 0.0214539 | 0.914039 | 0.00308845 | 56.81144774 |
|  |  | rs79790072 | T | C | 0.0567 | 0.226103 | 0.0488017 | 3.54E-06 | -0.0351942 | 0.043692 | 0.420528 | 0.005468592 | 100.8344663 |
|  |  | rs8028558 | A | G | 0.4046 | 0.08346 | 0.0188504 | 9.78E-06 | -0.00739528 | 0.015589 | 0.635221 | 0.003355996 | 61.74949005 |
|  |  | rs9864379 | T | C | 0.1133 | -0.160521 | 0.0292752 | 4.66E-08 | -0.0331073 | 0.0211651 | 0.117761 | 0.005177264 | 95.43476171 |
| ebi-a-GCST90016954 | Gut microbiota abundance (unknown family id.1000005471) | rs10071529 | C | G | 0.9125 | -0.125105 | 0.0278674 | 8.64E-06 | -0.00261483 | 0.024051 | 0.913424 | 0.002499311 | 45.94719676 |
|  |  | rs11779863 | G | A | 0.2068 | -0.0772768 | 0.0172171 | 6.69E-06 | 0.0198621 | 0.0205852 | 0.334609 | 0.001959122 | 35.9969027 |
|  |  | rs12566890 | T | G | 0.0954 | -0.103134 | 0.0241775 | 8.11E-06 | 0.0279033 | 0.0222866 | 0.210563 | 0.001835856 | 33.72785182 |
|  |  | rs13100746 | C | T | 0.4095 | 0.0638979 | 0.0142685 | 7.29E-06 | -0.0233567 | 0.0150067 | 0.119608 | 0.00197459 | 36.28167629 |
|  |  | rs17235252 | T | C | 0.0944 | -0.122002 | 0.0255463 | 2.16E-06 | -0.0209725 | 0.0231656 | 0.365291 | 0.002544909 | 46.78761649 |
|  |  | rs3932485 | C | T | 0.4284 | 0.0626209 | 0.0141623 | 9.93E-06 | 0.00510654 | 0.0153008 | 0.738574 | 0.001920482 | 35.28556804 |
|  |  | rs515984 | C | T | 0.826 | 0.0875451 | 0.0190685 | 6.61E-06 | -0.0197828 | 0.0233624 | 0.397118 | 0.002203043 | 40.48860099 |
|  |  | rs62188991 | G | C | 0.1113 | -0.110864 | 0.0241355 | 5.27E-06 | -0.0258113 | 0.0322986 | 0.424206 | 0.002431428 | 44.69620376 |
|  |  | rs638542 | A | G | 0.7137 | 0.070592 | 0.0157049 | 5.17E-06 | -0.00465961 | 0.0164752 | 0.77731 | 0.00203647 | 37.4209933 |
|  |  | rs74603314 | T | C | 0.0457 | 0.230767 | 0.0489701 | 2.28E-06 | -0.0621365 | 0.0382965 | 0.104694 | 0.004644923 | 85.57609412 |
|  |  | rs7706512 | A | G | 0.496 | -0.0657351 | 0.0138979 | 2.27E-06 | 0.00869044 | 0.0149476 | 0.560974 | 0.002160413 | 39.70343696 |
|  |  | rs7801843 | A | G | 0.1292 | -0.0869495 | 0.0194509 | 9.47E-06 | -0.0398097 | 0.0208393 | 0.0560919 | 0.00170116 | 31.24902773 |
|  |  | rs7853673 | A | G | 0.4861 | 0.0624389 | 0.0139833 | 6.73E-06 | 0.037232 | 0.014983 | 0.0129572 | 0.001947802 | 35.78849487 |
|  |  | rs949341 | A | G | 0.6799 | -0.0656602 | 0.0147216 | 7.73E-06 | -0.0125214 | 0.0167211 | 0.453954 | 0.001876571 | 34.477267 |
| ebi-a-GCST90016956 | Gut microbiota abundance (family Veillonellaceae id.2172) | rs111810795 | C | T | 0.1074 | -0.0867108 | 0.0180573 | 1.73E-06 | -0.0327524 | 0.0251573 | 0.192949 | 0.001441576 | 26.4737848 |
|  |  | rs114889439 | A | G | 0.0527 | -0.253809 | 0.0539169 | 6.19E-06 | -0.0266296 | 0.0412904 | 0.51897 | 0.006431943 | 118.7125221 |
|  |  | rs12186441 | G | A | 0.0368 | 0.208096 | 0.0453999 | 4.53E-06 | -0.0427974 | 0.0369309 | 0.246517 | 0.003069882 | 56.46885801 |
|  |  | rs12668619 | A | G | 0.3549 | 0.055301 | 0.0117654 | 2.57E-06 | 0.0249467 | 0.0159526 | 0.117865 | 0.001400326 | 25.71517916 |
|  |  | rs12741784 | C | T | 0.336 | -0.0621452 | 0.0119266 | 1.28E-07 | -0.0131704 | 0.0171071 | 0.441372 | 0.001723267 | 31.65581883 |
|  |  | rs1442060 | A | G | 0.506 | 0.0514024 | 0.0112125 | 4.51E-06 | 0.00720596 | 0.0150752 | 0.632649 | 0.001320913 | 24.25494354 |
|  |  | rs1447205 | C | G | 0.6382 | 0.0520689 | 0.0115656 | 7.35E-06 | 0.00156645 | 0.0155953 | 0.919992 | 0.001252023 | 22.98837217 |
|  |  | rs1693340 | T | C | 0.1262 | 0.0818934 | 0.018245 | 9.25E-06 | 0.028678 | 0.0288098 | 0.31953 | 0.001479106 | 27.16401783 |
|  |  | rs2175069 | G | A | 0.6143 | 0.0526752 | 0.0114886 | 4.64E-06 | 0.0113439 | 0.0154514 | 0.462848 | 0.001314839 | 24.1432598 |
|  |  | rs2561116 | T | G | 0.1133 | -0.083617 | 0.0187159 | 7.89E-06 | -0.0257913 | 0.0319906 | 0.420119 | 0.001404836 | 25.79813363 |
|  |  | rs2585520 | G | T | 0.0825 | -0.0904972 | 0.0200428 | 5.27E-06 | -0.0750678 | 0.0390027 | 0.0542688 | 0.001239825 | 22.76412979 |
|  |  | rs4263802 | A | G | 0.6163 | -0.050942 | 0.0114921 | 7.45E-06 | 0.00952593 | 0.0158874 | 0.54878 | 0.001227343 | 22.53467348 |
|  |  | rs4461038 | G | A | 0.6938 | 0.0554965 | 0.0119467 | 3.73E-06 | 0.0210796 | 0.0164693 | 0.200569 | 0.001308581 | 24.02820432 |
|  |  | rs4797169 | T | C | 0.2505 | 0.0587799 | 0.0128297 | 4.49E-06 | 0.0296386 | 0.0175953 | 0.0920937 | 0.00129738 | 23.82225273 |
|  |  | rs61264131 | A | C | 0.0497 | 0.202367 | 0.0464388 | 6.75E-06 | 0.00923689 | 0.0253521 | 0.715601 | 0.003868357 | 71.21340192 |
|  |  | rs6692542 | G | A | 0.6272 | -0.0534397 | 0.0118254 | 8.68E-06 | -0.0273683 | 0.0157403 | 0.0820805 | 0.001335488 | 24.522928 |
|  |  | rs6909981 | C | T | 0.2018 | -0.0638696 | 0.0141765 | 5.48E-06 | 0.0259992 | 0.0227899 | 0.253943 | 0.001314169 | 24.13094633 |
|  |  | rs75768969 | C | G | 0.161 | -0.0703773 | 0.0156855 | 7.19E-06 | -0.0525007 | 0.0268445 | 0.0504964 | 0.001338083 | 24.57064259 |
|  |  | rs79535861 | A | C | 0.0885 | 0.100666 | 0.0207016 | 1.58E-06 | 0.00760408 | 0.0261402 | 0.771131 | 0.001634916 | 30.03019472 |
|  |  | rs9345168 | A | C | 0.5338 | -0.0509102 | 0.0113253 | 8.49E-06 | -0.0120943 | 0.0150218 | 0.420753 | 0.001290002 | 23.68661557 |
| ebi-a-GCST90016957 | Gut microbiota abundance (family Verrucomicrobiaceae id.4036) | rs11184341 | C | G | 0.7217 | -0.0655301 | 0.014223 | 4.14E-06 | 0.000619331 | 0.0167557 | 0.970515 | 0.00172497 | 31.68716089 |
|  |  | rs111862613 | T | C | 0.166 | 0.0907065 | 0.0196746 | 3.73E-06 | -0.0161549 | 0.019977 | 0.418704 | 0.002278143 | 41.87197399 |
|  |  | rs117107102 | A | G | 0.0447 | 0.204683 | 0.0431572 | 2.92E-06 | -0.0175139 | 0.0354893 | 0.62166 | 0.003578004 | 65.84904888 |
|  |  | rs11729256 | T | C | 0.2386 | 0.0749798 | 0.0150177 | 6.73E-07 | -0.020361 | 0.0198965 | 0.306145 | 0.002042687 | 37.53547069 |
|  |  | rs12908520 | G | A | 0.4563 | 0.0619104 | 0.0130946 | 2.15E-06 | 0.0130387 | 0.0151002 | 0.387873 | 0.00190181 | 34.94183572 |
|  |  | rs2602429 | T | C | 0.7783 | -0.0745516 | 0.0156195 | 2.70E-06 | 0.0307546 | 0.0170892 | 0.0719167 | 0.001918036 | 35.24052883 |
|  |  | rs4242783 | A | G | 0.7127 | -0.0687992 | 0.0147694 | 2.75E-06 | -0.00533905 | 0.016681 | 0.748918 | 0.001938381 | 35.6150674 |
|  |  | rs4936098 | G | A | 0.66 | -0.0648691 | 0.0135928 | 1.13E-06 | -0.00318203 | 0.015683 | 0.839215 | 0.00188855 | 34.69776683 |
|  |  | rs61779207 | G | A | 0.1779 | -0.0758811 | 0.0167762 | 6.63E-06 | 0.00454658 | 0.018047 | 0.801096 | 0.001684216 | 30.93726082 |
|  |  | rs74542928 | T | C | 0.0785 | 0.112124 | 0.0236422 | 1.65E-06 | 0.025264 | 0.034997 | 0.470362 | 0.00181883 | 33.41448354 |
|  |  | rs9349825 | A | G | 0.2793 | -0.0704354 | 0.0147128 | 2.51E-06 | -0.039773 | 0.0191289 | 0.0375985 | 0.001997273 | 36.69929018 |
|  |  | rs941682 | G | A | 0.3111 | -0.0631526 | 0.0143767 | 9.58E-06 | 0.017921 | 0.0167318 | 0.284134 | 0.001709498 | 31.40246146 |
| ebi-a-GCST90016960 | Gut microbiota abundance (genus Adlercreutzia id.812) | rs1046175 | G | C | 0.8738 | -0.112758 | 0.0255994 | 6.36E-06 | -0.0290317 | 0.0294283 | 0.323876 | 0.002804117 | 51.56649471 |
|  |  | rs11604400 | C | T | 0.1153 | -0.102524 | 0.0234834 | 9.74E-06 | -0.0272722 | 0.0244942 | 0.26553 | 0.002144403 | 39.4085708 |
|  |  | rs12522517 | A | T | 0.1272 | -0.104884 | 0.0234695 | 4.41E-06 | -0.0163149 | 0.0212314 | 0.442229 | 0.002442589 | 44.90186678 |
|  |  | rs13231526 | C | A | 0.0636 | 0.143237 | 0.0311654 | 4.81E-06 | -0.0114922 | 0.0273939 | 0.674837 | 0.002443762 | 44.92349422 |
|  |  | rs2147798 | G | C | 0.8042 | -0.0922959 | 0.0192121 | 1.40E-06 | -0.00689741 | 0.0173082 | 0.690257 | 0.002682697 | 49.32762257 |
|  |  | rs2717140 | C | T | 0.1113 | -0.119225 | 0.0251081 | 2.05E-06 | -0.00235171 | 0.0250619 | 0.925239 | 0.002811998 | 51.71183229 |
|  |  | rs55719207 | G | A | 0.4175 | -0.0699243 | 0.0158036 | 9.61E-06 | 0.0148949 | 0.0153491 | 0.331845 | 0.002378147 | 43.71441536 |
|  |  | rs6664405 | T | C | 0.1441 | -0.0953084 | 0.0210752 | 5.23E-06 | -0.00106808 | 0.021324 | 0.960052 | 0.002240678 | 41.18181987 |
|  |  | rs7680684 | T | C | 0.675 | 0.083381 | 0.0168901 | 9.77E-07 | 0.00798038 | 0.0157516 | 0.612408 | 0.003050362 | 56.1086832 |
|  |  | rs80078995 | A | T | 0.1342 | -0.11324 | 0.0232586 | 1.57E-06 | 0.035941 | 0.0212226 | 0.0903566 | 0.002979887 | 54.8084933 |
|  |  | rs9490822 | C | T | 0.4473 | -0.0734512 | 0.0155789 | 2.54E-06 | -0.00154722 | 0.0150082 | 0.917891 | 0.002667572 | 49.04877637 |
|  |  | rs9915817 | C | T | 0.671 | -0.0749219 | 0.016833 | 8.22E-06 | -0.00478248 | 0.016547 | 0.772563 | 0.002478369 | 45.56124941 |
| ebi-a-GCST90016963 | Gut microbiota abundance (genus Allisonella id.2174) | rs1901739 | G | T | 0.505 | -0.115769 | 0.0248626 | 3.59E-06 | -0.0167119 | 0.0149829 | 0.264681 | 0.006700561 | 123.7037641 |
|  |  | rs35110698 | T | C | 0.1491 | -0.146322 | 0.0320852 | 5.72E-06 | -0.0253679 | 0.0212164 | 0.231823 | 0.005432571 | 100.1666515 |
|  |  | rs35778461 | C | T | 0.1918 | 0.146679 | 0.0297186 | 1.21E-06 | 0.00740078 | 0.0178123 | 0.677785 | 0.006670115 | 123.137914 |
|  |  | rs594561 | T | C | 0.5586 | -0.112231 | 0.0251681 | 9.41E-06 | 0.0063173 | 0.0149352 | 0.672309 | 0.006211392 | 114.6164295 |
|  |  | rs602075 | G | A | 0.7694 | -0.168974 | 0.0296976 | 3.57E-08 | -0.0189133 | 0.0169881 | 0.265568 | 0.010131675 | 187.6963345 |
|  |  | rs6742198 | A | G | 0.8042 | -0.149152 | 0.0316484 | 3.35E-06 | -0.00363676 | 0.0175606 | 0.835933 | 0.007005916 | 129.380916 |
|  |  | rs685403 | G | C | 0.1083 | -0.175203 | 0.0404474 | 4.88E-06 | 0.0114844 | 0.0221776 | 0.604571 | 0.005928711 | 109.369124 |
|  |  | rs76904847 | G | A | 0.1899 | 0.148523 | 0.0334857 | 6.09E-06 | 0.000615905 | 0.0197755 | 0.975154 | 0.00678705 | 125.311409 |
|  |  | rs7898615 | T | G | 0.1153 | 0.167966 | 0.0373586 | 8.87E-06 | -0.0179378 | 0.0218528 | 0.411733 | 0.005755699 | 106.1590316 |
| ebi-a-GCST90016966 | Gut microbiota abundance (genus Anaerostipes id.1991) | rs10502061 | A | G | 0.0944 | 0.0835752 | 0.0192017 | 7.94E-06 | -0.002458 | 0.0233892 | 0.916303 | 0.001194245 | 21.92624109 |
|  |  | rs13376554 | A | T | 0.0308 | 0.197389 | 0.0458642 | 7.72E-06 | 0.0297598 | 0.0476986 | 0.532684 | 0.002326162 | 42.75662295 |
|  |  | rs2014785 | T | C | 0.3887 | 0.0515687 | 0.0112199 | 4.68E-06 | -0.0248692 | 0.0151765 | 0.101283 | 0.001263779 | 23.20451346 |
|  |  | rs2396460 | C | T | 0.5606 | 0.0512653 | 0.0109555 | 2.91E-06 | -0.0207167 | 0.0150178 | 0.16775 | 0.001294763 | 23.77413853 |
|  |  | rs2804244 | G | A | 0.5447 | 0.053089 | 0.0110983 | 2.04E-06 | -0.00748393 | 0.0153933 | 0.626839 | 0.001397958 | 25.67164093 |
|  |  | rs3900776 | G | A | 0.0706 | -0.110013 | 0.0236302 | 2.75E-06 | 0.00769546 | 0.0454106 | 0.865432 | 0.001588274 | 29.17209881 |
|  |  | rs60983350 | G | A | 0.4354 | -0.0539901 | 0.0116575 | 4.42E-06 | 0.0111303 | 0.0159981 | 0.486599 | 0.001433137 | 26.3185753 |
|  |  | rs62157625 | T | C | 0.1133 | 0.088573 | 0.0185548 | 1.45E-06 | -0.018631 | 0.0229945 | 0.417803 | 0.001576302 | 28.95185606 |
|  |  | rs62215703 | G | A | 0.2197 | 0.0644965 | 0.0136702 | 1.98E-06 | 0.0102212 | 0.0182001 | 0.574389 | 0.001426244 | 26.19182591 |
|  |  | rs6474958 | G | A | 0.5974 | 0.0500065 | 0.0112001 | 6.74E-06 | 0.00350662 | 0.0161206 | 0.8278 | 0.001202879 | 22.08495857 |
|  |  | rs6726833 | C | A | 0.0944 | -0.0877497 | 0.0189408 | 3.32E-06 | 0.00447496 | 0.027704 | 0.871678 | 0.001316527 | 24.17429346 |
|  |  | rs6854026 | C | T | 0.4881 | 0.0508482 | 0.0109075 | 3.20E-06 | 0.00967415 | 0.0151158 | 0.52217 | 0.001292037 | 23.72403501 |
|  |  | rs7193624 | T | C | 0.8509 | -0.0750642 | 0.0150723 | 5.35E-07 | 0.00728113 | 0.0271348 | 0.788444 | 0.001429723 | 26.25579768 |
|  |  | rs7823228 | C | G | 0.7843 | 0.0621085 | 0.0136614 | 5.84E-06 | 0.014585 | 0.0199425 | 0.464563 | 0.001305162 | 23.9653403 |
|  |  | rs78735375 | A | C | 0.0527 | -0.137429 | 0.0305304 | 5.33E-06 | -0.0363553 | 0.038132 | 0.340384 | 0.001885753 | 34.64628191 |
| ebi-a-GCST90016967 | Gut microbiota abundance (genus Anaerotruncus id.2054) | rs10150232 | A | G | 0.2535 | 0.0567088 | 0.0124877 | 6.68E-06 | 0.0228851 | 0.0187028 | 0.221095 | 0.001217135 | 22.34701762 |
|  |  | rs11018566 | A | G | 0.0517 | -0.156465 | 0.0366032 | 6.14E-06 | -0.0117487 | 0.0334728 | 0.725595 | 0.002400494 | 44.12619109 |
|  |  | rs115414803 | A | C | 0.0586 | -0.144356 | 0.0317524 | 6.83E-06 | -0.0322963 | 0.0311544 | 0.299897 | 0.002299172 | 42.25937995 |
|  |  | rs12056802 | C | G | 0.0825 | 0.0774194 | 0.0177178 | 6.13E-06 | -0.0086304 | 0.022418 | 0.700255 | 0.000907381 | 16.65466254 |
|  |  | rs1272208 | T | G | 0.7197 | 0.0611743 | 0.0129831 | 4.28E-06 | 0.0276449 | 0.0176065 | 0.116379 | 0.001509881 | 27.73006004 |
|  |  | rs1431492 | C | T | 0.1829 | -0.0654996 | 0.0146188 | 7.36E-06 | 0.01715 | 0.0205683 | 0.404389 | 0.001282319 | 23.54536554 |
|  |  | rs17734739 | T | C | 0.1899 | 0.0660052 | 0.014908 | 7.43E-06 | -0.0272488 | 0.0215186 | 0.20541 | 0.001340448 | 24.61412508 |
|  |  | rs34449434 | A | C | 0.4563 | -0.0497004 | 0.0113402 | 9.85E-06 | -0.0142087 | 0.0156639 | 0.364353 | 0.001225631 | 22.503193 |
|  |  | rs4669806 | G | T | 0.2495 | 0.0576389 | 0.0122994 | 2.42E-06 | -0.00327138 | 0.0182368 | 0.857637 | 0.001244178 | 22.84416323 |
|  |  | rs6494922 | A | G | 0.0726 | 0.0903106 | 0.0202257 | 6.62E-06 | 0.0159482 | 0.0333256 | 0.632255 | 0.001098275 | 20.16231375 |
|  |  | rs6563550 | T | C | 0.0785 | 0.0877135 | 0.0176745 | 2.35E-07 | 0.031534 | 0.0279076 | 0.2585 | 0.001113084 | 20.43447656 |
|  |  | rs7155595 | C | A | 0.3091 | 0.0539336 | 0.0118903 | 7.55E-06 | -0.027644 | 0.016318 | 0.0902506 | 0.001242404 | 22.81155476 |
|  |  | rs8005030 | C | T | 0.3022 | 0.0554447 | 0.0117854 | 2.28E-06 | 0.0109358 | 0.0158771 | 0.490962 | 0.001296509 | 23.8062449 |
|  |  | rs9347879 | T | C | 0.4602 | 0.050618 | 0.011049 | 4.22E-06 | -0.00560959 | 0.0149385 | 0.707281 | 0.001272974 | 23.37354681 |
| ebi-a-GCST90016972 | Gut microbiota abundance (genus Blautia id.1992) | rs115043014 | G | A | 0.0437 | -0.206605 | 0.043992 | 5.19E-06 | -0.00593953 | 0.0579228 | 0.918326 | 0.003567691 | 65.65856867 |
|  |  | rs2788271 | G | T | 0.835 | 0.0575596 | 0.0133492 | 7.16E-06 | -0.00311158 | 0.0198181 | 0.875239 | 0.000912927 | 16.7565489 |
| ebi-a-GCST90016973 | Gut microbiota abundance (genus Butyricicoccus id.2055) | rs10084203 | G | A | 0.7584 | -0.0549699 | 0.0123563 | 8.59E-06 | -0.00674597 | 0.0221886 | 0.761106 | 0.001107325 | 20.32863807 |
|  |  | rs12034718 | G | A | 0.835 | -0.0701199 | 0.0158213 | 9.58E-06 | -0.0698705 | 0.0181342 | 0.000116692 | 0.001354824 | 24.87847478 |
|  |  | rs12585793 | T | C | 0.0527 | -0.262206 | 0.0564729 | 5.79E-06 | -0.0330287 | 0.0448479 | 0.46145 | 0.006864571 | 126.7526045 |
|  |  | rs2017189 | T | G | 0.5785 | 0.0506956 | 0.011024 | 3.87E-06 | 0.00106977 | 0.0149735 | 0.943044 | 0.001253347 | 23.01272801 |
|  |  | rs4962426 | T | G | 0.8022 | -0.0614216 | 0.0135979 | 7.38E-06 | -0.0126206 | 0.0188395 | 0.502922 | 0.00119724 | 21.98130265 |
|  |  | rs56221232 | T | C | 0.1083 | 0.0828027 | 0.0167401 | 7.62E-07 | 0.0133172 | 0.024834 | 0.591786 | 0.001324239 | 24.31608622 |
|  |  | rs62478070 | T | G | 0.0437 | 0.224039 | 0.0494959 | 5.94E-06 | 0.00317905 | 0.0474063 | 0.946534 | 0.004195202 | 77.2557109 |
|  |  | rs7322368 | C | T | 0.9155 | -0.0815733 | 0.0183167 | 5.52E-06 | -0.0132428 | 0.0260682 | 0.611449 | 0.001029535 | 18.89907014 |
|  |  | rs75238760 | T | A | 0.1938 | 0.0619423 | 0.0139942 | 6.80E-06 | -0.0112653 | 0.0221426 | 0.610918 | 0.00119895 | 22.01274461 |
| ebi-a-GCST90016976 | Gut microbiota abundance (genus Candidatus Soleaferrea id.11350) | rs10090365 | G | A | 0.4414 | 0.0834413 | 0.0180989 | 4.17E-06 | 0.00489136 | 0.0149423 | 0.743402 | 0.003433408 | 63.17874917 |
|  |  | rs10809135 | C | T | 0.5089 | -0.0834858 | 0.0182426 | 5.47E-06 | 0.00860586 | 0.0151955 | 0.571162 | 0.003483835 | 64.10991889 |
|  |  | rs11153159 | G | C | 0.1292 | -0.12805 | 0.0285446 | 4.42E-06 | -0.0133988 | 0.0214154 | 0.531536 | 0.003689522 | 67.90900479 |
|  |  | rs2193878 | T | A | 0.0487 | 0.228255 | 0.0509241 | 9.46E-06 | 0.101355 | 0.0485255 | 0.036735 | 0.004827442 | 88.95505438 |
|  |  | rs36155147 | T | C | 0.6382 | -0.104966 | 0.0240995 | 5.41E-06 | 0.00906227 | 0.0160394 | 0.572074 | 0.005088065 | 93.78210596 |
|  |  | rs4294381 | C | T | 0.8201 | -0.112197 | 0.023186 | 1.37E-06 | 0.00361186 | 0.0205463 | 0.860458 | 0.003714415 | 68.36889834 |
|  |  | rs4678258 | T | C | 0.2763 | 0.0986136 | 0.0215646 | 5.53E-06 | 0.020169 | 0.0175363 | 0.25009 | 0.003889046 | 71.5957648 |
|  |  | rs6489992 | A | G | 0.3847 | -0.0840437 | 0.0187031 | 7.89E-06 | -0.020072 | 0.015461 | 0.194206 | 0.00334387 | 61.52562739 |
|  |  | rs6494306 | A | G | 0.1968 | -0.0969304 | 0.0214221 | 5.80E-06 | -0.0224354 | 0.0159997 | 0.160845 | 0.00297029 | 54.63144249 |
|  |  | rs7400877 | C | T | 0.7495 | 0.0951085 | 0.0212799 | 9.29E-06 | 0.0179082 | 0.01829 | 0.327517 | 0.003396628 | 62.49965843 |
|  |  | rs9973954 | G | A | 0.6938 | -0.0892075 | 0.0195401 | 5.95E-06 | -0.0157521 | 0.015685 | 0.315245 | 0.003381211 | 62.215001 |
| ebi-a-GCST90016977 | Gut microbiota abundance (genus Catenibacterium id.2153) | rs12404911 | C | T | 0.17 | 0.140717 | 0.0304142 | 2.80E-06 | -0.00945839 | 0.0191658 | 0.621657 | 0.00558792 | 103.0470875 |
|  |  | rs212393 | A | G | 0.6899 | 0.135254 | 0.0286209 | 3.62E-06 | 0.0438815 | 0.0184437 | 0.0173496 | 0.007827411 | 144.6714673 |
|  |  | rs73128290 | A | G | 0.2634 | 0.129726 | 0.0284563 | 4.29E-06 | 0.00879985 | 0.0163421 | 0.590249 | 0.006530276 | 120.539356 |
|  |  | rs7742829 | C | T | 0.4195 | 0.11411 | 0.0251099 | 5.61E-06 | -0.0116404 | 0.0150477 | 0.439184 | 0.006341786 | 117.0379044 |
| ebi-a-GCST90016979 | Gut microbiota abundance (genus Clostridium innocuum group id.14397) | rs10506058 | A | G | 0.3966 | 0.0997048 | 0.0221926 | 8.92E-06 | 0.0418067 | 0.0151564 | 0.00580938 | 0.004757953 | 87.66846395 |
|  |  | rs1942371 | G | A | 0.1302 | -0.157938 | 0.034187 | 4.06E-06 | -0.0118405 | 0.0229277 | 0.605556 | 0.005649808 | 104.194851 |
|  |  | rs1948423 | T | A | 0.3082 | -0.108859 | 0.023425 | 3.49E-06 | -0.00639213 | 0.0157331 | 0.684532 | 0.005053263 | 93.13737743 |
|  |  | rs40656 | C | T | 0.1889 | 0.142664 | 0.0311021 | 8.62E-06 | 0.00516209 | 0.0187193 | 0.78273 | 0.006236848 | 115.0891088 |
|  |  | rs4869133 | G | A | 0.0885 | -0.180591 | 0.0409505 | 7.24E-06 | 0.0130512 | 0.0197455 | 0.508629 | 0.005261652 | 96.99855239 |
|  |  | rs61267978 | T | C | 0.1243 | 0.14708 | 0.0320875 | 5.59E-06 | 0.0160634 | 0.0229001 | 0.483019 | 0.00470938 | 86.76923601 |
|  |  | rs6577484 | G | A | 0.1481 | 0.160425 | 0.0360857 | 8.41E-06 | -0.0156288 | 0.0245616 | 0.524575 | 0.006494082 | 119.8669013 |
|  |  | rs6890185 | C | T | 0.6889 | -0.113424 | 0.0233137 | 1.12E-06 | 0.00372511 | 0.0160007 | 0.81591 | 0.005514373 | 101.6832847 |
|  |  | rs71564433 | T | A | 0.1789 | -0.126746 | 0.0274657 | 7.80E-06 | 0.000809925 | 0.0187806 | 0.965601 | 0.004719597 | 86.95837757 |
|  |  | rs77845139 | A | G | 0.2952 | -0.114993 | 0.0257186 | 8.41E-06 | -0.0126377 | 0.0172783 | 0.464523 | 0.005502437 | 101.4619717 |
| ebi-a-GCST90016982 | Gut microbiota abundance (genus Coprobacter id.949) | rs11532348 | C | T | 0.1829 | -0.103931 | 0.0226826 | 5.71E-06 | 0.0206774 | 0.020957 | 0.323808 | 0.003228562 | 59.39713631 |
|  |  | rs12684609 | T | C | 0.1978 | 0.100827 | 0.0220366 | 6.10E-06 | -0.00360247 | 0.0188522 | 0.848455 | 0.00322621 | 59.35372634 |
|  |  | rs12996055 | A | C | 0.1918 | 0.0921817 | 0.0209405 | 8.08E-06 | -0.0297662 | 0.0170556 | 0.0809431 | 0.002634431 | 48.43780648 |
|  |  | rs143662916 | C | T | 0.0577 | 0.253264 | 0.0540141 | 3.07E-06 | -0.0789684 | 0.0440929 | 0.0732993 | 0.006974963 | 128.8052881 |
|  |  | rs189356 | G | A | 0.5219 | 0.0781125 | 0.0171916 | 6.26E-06 | 0.015017 | 0.0151414 | 0.321303 | 0.003044929 | 56.00844214 |
|  |  | rs213863 | T | C | 0.6909 | 0.0887412 | 0.0188377 | 2.35E-06 | -0.0103787 | 0.0155427 | 0.504291 | 0.003363526 | 61.88850318 |
|  |  | rs28402691 | T | C | 0.167 | 0.110695 | 0.0251199 | 9.56E-06 | 0.0235584 | 0.0210677 | 0.263472 | 0.003409161 | 62.73104973 |
|  |  | rs305411 | A | G | 0.1352 | 0.129218 | 0.0264518 | 1.01E-06 | -0.0102178 | 0.024726 | 0.679429 | 0.003904527 | 71.88187549 |
|  |  | rs3828477 | G | T | 0.2813 | -0.0912179 | 0.019569 | 2.89E-06 | -0.0265507 | 0.0157821 | 0.0925039 | 0.003364399 | 61.90462314 |
|  |  | rs5011652 | C | G | 0.7545 | -0.0899597 | 0.020001 | 5.51E-06 | 0.00600351 | 0.0168534 | 0.721676 | 0.002998035 | 55.14329173 |
|  |  | rs55672356 | T | A | 0.0527 | -0.193354 | 0.0413815 | 2.74E-06 | -0.083305 | 0.0428928 | 0.0521171 | 0.003732798 | 68.7085218 |
|  |  | rs72821405 | T | C | 0.0885 | -0.147365 | 0.0319978 | 4.76E-06 | 0.0372594 | 0.0264828 | 0.159449 | 0.003503633 | 64.47552469 |
|  |  | rs74919520 | G | A | 0.1282 | 0.125703 | 0.0276296 | 5.76E-06 | 0.0158038 | 0.0240804 | 0.511634 | 0.003532045 | 65.00021631 |
|  |  | rs76001613 | C | G | 0.0696 | 0.21591 | 0.0493019 | 9.13E-06 | -0.0871073 | 0.0305324 | 0.00433162 | 0.006037463 | 111.3874865 |
| ebi-a-GCST90016983 | Gut microbiota abundance (genus Coprococcus1 id.11301) | rs1010560 | C | A | 0.2962 | 0.0580232 | 0.0122722 | 1.96E-06 | -0.0384539 | 0.0169111 | 0.0229731 | 0.001403679 | 25.77683978 |
|  |  | rs12794898 | G | T | 0.0944 | 0.0903299 | 0.0197212 | 4.92E-06 | -0.0180614 | 0.0224945 | 0.422018 | 0.001395088 | 25.61885602 |
|  |  | rs12886051 | G | C | 0.3429 | -0.0521782 | 0.0118051 | 8.01E-06 | -0.0253859 | 0.016407 | 0.1218 | 0.001226894 | 22.52642446 |
|  |  | rs1519491 | T | C | 0.4085 | 0.0499197 | 0.0113558 | 8.95E-06 | -0.00644019 | 0.0152888 | 0.673583 | 0.001204261 | 22.11037083 |
|  |  | rs1576241 | A | G | 0.4394 | -0.0510343 | 0.0109535 | 3.33E-06 | -0.0230469 | 0.0153792 | 0.133984 | 0.001283121 | 23.56009539 |
|  |  | rs2907920 | G | A | 0.7097 | -0.0561173 | 0.0126824 | 7.65E-06 | -0.00741379 | 0.0166699 | 0.656506 | 0.001297614 | 23.82655492 |
|  |  | rs4277593 | G | A | 0.4831 | -0.0585649 | 0.0109914 | 1.14E-07 | 0.0131061 | 0.0151848 | 0.388079 | 0.001712965 | 31.46624464 |
|  |  | rs56405618 | A | G | 0.1113 | -0.08963 | 0.0186508 | 1.57E-06 | -0.0171432 | 0.0234836 | 0.465385 | 0.001589231 | 29.18971434 |
|  |  | rs73031725 | T | C | 0.0606 | 0.167591 | 0.0355224 | 1.98E-06 | 0.0485436 | 0.0426648 | 0.255208 | 0.003197824 | 58.82982431 |
|  |  | rs73167075 | T | C | 0.2396 | 0.0573137 | 0.0127573 | 8.57E-06 | -0.0130917 | 0.0183062 | 0.474517 | 0.001196949 | 21.97596315 |
|  |  | rs74101919 | T | C | 0.1869 | -0.0718988 | 0.0144651 | 1.03E-06 | 0.0373151 | 0.0240928 | 0.121429 | 0.001571182 | 28.85767931 |
|  |  | rs7784490 | G | C | 0.6113 | 0.0518543 | 0.0113136 | 4.55E-06 | 0.0125905 | 0.0158544 | 0.427119 | 0.001277816 | 23.46257943 |
|  |  | rs946513 | T | C | 0.9453 | -0.205899 | 0.0460018 | 8.62E-06 | -0.0030125 | 0.0359944 | 0.9333 | 0.004384251 | 80.75244216 |
| ebi-a-GCST90016986 | Gut microbiota abundance (genus Defluviitaleaceae UCG011 id.11287) | rs112893842 | T | C | 0.0974 | 0.11381 | 0.0232806 | 1.45E-06 | 0.0148179 | 0.0260614 | 0.569643 | 0.00227743 | 41.85885073 |
|  |  | rs1582238 | C | T | 0.6282 | -0.0805036 | 0.0167257 | 1.57E-06 | -0.000712156 | 0.0156324 | 0.963664 | 0.003027387 | 55.68479905 |
|  |  | rs28696126 | T | A | 0.8082 | 0.106503 | 0.0238473 | 6.63E-06 | -0.00434422 | 0.0224426 | 0.846512 | 0.003516585 | 64.71471168 |
|  |  | rs2892880 | G | A | 0.2604 | 0.0817755 | 0.0181686 | 6.83E-06 | 0.00131847 | 0.0172494 | 0.939073 | 0.002575813 | 47.35723823 |
|  |  | rs4344384 | T | G | 0.5258 | -0.0715978 | 0.0156309 | 4.83E-06 | 0.0116074 | 0.0149832 | 0.43852 | 0.002556298 | 46.9975327 |
|  |  | rs4677103 | A | G | 0.171 | 0.0977912 | 0.0197263 | 9.60E-07 | -0.0582774 | 0.0195206 | 0.00283178 | 0.002711316 | 49.85529206 |
|  |  | rs55658617 | T | C | 0.0696 | 0.174369 | 0.0362226 | 2.15E-06 | 0.0146385 | 0.0402815 | 0.716303 | 0.003937744 | 72.49582152 |
|  |  | rs72731813 | C | T | 0.0815 | -0.147383 | 0.0293796 | 4.33E-07 | -0.00468596 | 0.0348772 | 0.893121 | 0.003252082 | 59.83126446 |
|  |  | rs9608282 | T | G | 0.0656 | 0.142939 | 0.0299793 | 2.52E-06 | 0.00850417 | 0.0421103 | 0.839956 | 0.002504772 | 46.04784234 |
|  |  | rs9725395 | A | G | 0.0875 | -0.13834 | 0.029554 | 3.52E-06 | 0.0091969 | 0.0236063 | 0.696835 | 0.003056092 | 56.21441677 |
| ebi-a-GCST90016989 | Gut microbiota abundance (genus Dorea id.1997) | rs11150408 | G | T | 0.5547 | -0.0488254 | 0.0109063 | 7.06E-06 | -0.00874306 | 0.0150326 | 0.560831 | 0.001177694 | 21.62201739 |
|  |  | rs12216169 | T | A | 0.0835 | 0.0881722 | 0.0193683 | 5.33E-06 | -0.0167176 | 0.0231836 | 0.47085 | 0.001189905 | 21.84647338 |
|  |  | rs12537781 | T | C | 0.2296 | -0.055544 | 0.0125223 | 9.15E-06 | -0.00281562 | 0.017499 | 0.872171 | 0.001091421 | 20.03635342 |
|  |  | rs13279148 | G | A | 0.166 | 0.0715309 | 0.0150878 | 2.25E-06 | 0.0445331 | 0.023952 | 0.0629898 | 0.001416744 | 26.01711892 |
|  |  | rs1899291 | T | C | 0.8718 | -0.0697217 | 0.0150285 | 4.57E-06 | -0.00473161 | 0.020626 | 0.818558 | 0.001086603 | 19.94779754 |
|  |  | rs3005511 | G | A | 0.664 | -0.0516127 | 0.0112852 | 5.29E-06 | 0.0256746 | 0.0162663 | 0.114475 | 0.00118864 | 21.82322877 |
|  |  | rs345219 | G | T | 0.6243 | 0.0497368 | 0.0112621 | 8.80E-06 | 0.0185905 | 0.0150937 | 0.218073 | 0.001160433 | 21.3047497 |
|  |  | rs3752849 | G | A | 0.0328 | 0.163846 | 0.036608 | 7.68E-06 | 0.0337933 | 0.0343726 | 0.325535 | 0.001703303 | 31.28845712 |
|  |  | rs4793307 | C | T | 0.2455 | 0.0574165 | 0.0122472 | 4.01E-06 | 0.00035348 | 0.0176126 | 0.983988 | 0.001221277 | 22.42316192 |
|  |  | rs62503162 | A | G | 0.0875 | -0.0974091 | 0.0194379 | 7.47E-07 | 0.0150123 | 0.0368035 | 0.683343 | 0.0015152 | 27.82790383 |
|  |  | rs62583469 | T | A | 0.1988 | -0.0634136 | 0.0142227 | 5.78E-06 | 0.00737361 | 0.0217769 | 0.734913 | 0.001281009 | 23.52127146 |
|  |  | rs73729431 | C | T | 0.0606 | -0.13745 | 0.0299983 | 3.17E-06 | 0.110412 | 0.0531656 | 0.0378234 | 0.002151011 | 39.53027275 |
| ebi-a-GCST90016992 | Gut microbiota abundance (genus Enterorhabdus id.820) | rs10098492 | T | C | 0.0905 | 0.132323 | 0.0293688 | 6.41E-06 | 0.0258432 | 0.0320295 | 0.419749 | 0.002882385 | 53.00996716 |
|  |  | rs114731706 | T | G | 0.0835 | 0.182308 | 0.0382298 | 2.17E-06 | -0.0237981 | 0.0422508 | 0.573259 | 0.005086984 | 93.7620836 |
|  |  | rs2051957 | C | T | 0.2346 | 0.0843291 | 0.0189864 | 8.90E-06 | 0.0120129 | 0.0187929 | 0.522677 | 0.002553885 | 46.95306177 |
|  |  | rs3017103 | G | A | 0.8101 | -0.0980897 | 0.0208995 | 2.94E-06 | 0.0309563 | 0.0190932 | 0.104949 | 0.002960332 | 54.4477498 |
|  |  | rs73331712 | T | C | 0.0427 | 0.26199 | 0.055123 | 4.85E-06 | -0.00223425 | 0.0375618 | 0.952568 | 0.005611453 | 103.4835251 |
|  |  | rs77655283 | G | A | 0.0686 | 0.132988 | 0.0298306 | 5.88E-06 | -0.00727854 | 0.0292581 | 0.803539 | 0.002260035 | 41.53840865 |
|  |  | rs7923280 | T | A | 0.6054 | -0.0860019 | 0.0169148 | 5.24E-07 | -0.00873176 | 0.016706 | 0.601203 | 0.003533829 | 65.03318055 |
| ebi-a-GCST90016995 | Gut microbiota abundance (genus Escherichia Shigella id.3504) | rs112767262 | T | C | 0.1958 | 0.0732963 | 0.0163589 | 8.21E-06 | -0.0388891 | 0.0180693 | 0.0313798 | 0.001691885 | 31.07836929 |
|  |  | rs113127095 | A | G | 0.0517 | 0.151009 | 0.0323434 | 3.33E-06 | -0.0438056 | 0.0377863 | 0.246334 | 0.002236001 | 41.09567243 |
|  |  | rs113513883 | A | G | 0.0577 | 0.172283 | 0.038041 | 5.28E-06 | 0.0152933 | 0.0420697 | 0.716214 | 0.003227601 | 59.3794015 |
|  |  | rs1154904 | G | A | 0.5358 | 0.0613376 | 0.0130644 | 3.04E-06 | 0.0266749 | 0.0149282 | 0.0739571 | 0.001871507 | 34.38404085 |
|  |  | rs11706043 | T | A | 0.1571 | 0.075657 | 0.0164155 | 5.87E-06 | 0.00560359 | 0.0181101 | 0.757004 | 0.001515935 | 27.84141457 |
|  |  | rs117092367 | A | T | 0.0885 | 0.117362 | 0.0264533 | 9.65E-06 | -0.0262707 | 0.0301716 | 0.383912 | 0.002222209 | 40.84163112 |
|  |  | rs118526 | A | C | 0.6074 | 0.0594401 | 0.013598 | 8.00E-06 | 0.00295795 | 0.0163767 | 0.856666 | 0.001685055 | 30.95269991 |
|  |  | rs2267739 | G | C | 0.0855 | 0.115505 | 0.0237628 | 1.42E-06 | 0.010435 | 0.0235386 | 0.657537 | 0.002086322 | 38.3389648 |
|  |  | rs2798105 | A | G | 0.1183 | -0.100851 | 0.0222045 | 8.24E-06 | -0.0329724 | 0.0252178 | 0.191041 | 0.002121759 | 38.99154233 |
|  |  | rs35555519 | C | G | 0.1143 | 0.101976 | 0.0222883 | 4.92E-06 | -0.0349165 | 0.0250711 | 0.163711 | 0.002105517 | 38.69244405 |
|  |  | rs4731451 | G | A | 0.3718 | -0.0609885 | 0.0135157 | 7.47E-06 | 0.0040602 | 0.0160939 | 0.800824 | 0.001737534 | 31.91835079 |
|  |  | rs57024273 | T | C | 0.3181 | 0.0626172 | 0.0139919 | 9.70E-06 | -0.0110003 | 0.0171357 | 0.520903 | 0.00170099 | 31.2458951 |
|  |  | rs592299 | T | C | 0.4682 | -0.0592004 | 0.0129486 | 4.77E-06 | 0.00674313 | 0.0150455 | 0.654021 | 0.001745256 | 32.0604494 |
|  |  | rs73208162 | A | G | 0.0586 | -0.119296 | 0.024839 | 2.19E-06 | -0.0761561 | 0.0418621 | 0.0688795 | 0.001570195 | 28.83951822 |
|  |  | rs7502686 | G | C | 0.0626 | -0.136384 | 0.0302576 | 5.90E-06 | -0.00148324 | 0.0310221 | 0.961866 | 0.002183012 | 40.11965597 |
| ebi-a-GCST90016996 | Gut microbiota abundance (genus Eubacterium brachy group id.11296) | rs112617308 | T | C | 0.0795 | -0.170873 | 0.0362844 | 2.38E-06 | -0.00632775 | 0.0259114 | 0.80707 | 0.004273344 | 78.70088944 |
|  |  | rs12151423 | G | A | 0.5209 | -0.101329 | 0.0227324 | 9.27E-06 | 0.0119002 | 0.0149464 | 0.42592 | 0.005124813 | 94.46292876 |
|  |  | rs13139592 | T | C | 0.1223 | -0.145997 | 0.0327192 | 7.97E-06 | 0.000102402 | 0.022058 | 0.996296 | 0.004576046 | 84.30130465 |
|  |  | rs1384962 | G | A | 0.7893 | -0.120888 | 0.0266264 | 6.99E-06 | -0.00973616 | 0.0161184 | 0.545818 | 0.004860747 | 89.57176467 |
|  |  | rs2913110 | T | C | 0.6223 | -0.105143 | 0.022942 | 4.56E-06 | -0.00346037 | 0.0156755 | 0.825287 | 0.005196818 | 95.79708914 |
|  |  | rs4862235 | A | G | 0.5557 | -0.104806 | 0.0225751 | 3.73E-06 | 0.0195397 | 0.0150426 | 0.193959 | 0.005423991 | 100.0075959 |
|  |  | rs62348779 | T | C | 0.0815 | -0.201481 | 0.043286 | 3.78E-06 | -0.0149981 | 0.028221 | 0.595106 | 0.00607764 | 112.133265 |
|  |  | rs6591893 | A | G | 0.6521 | -0.108225 | 0.0240319 | 7.34E-06 | 0.00473745 | 0.0157588 | 0.763702 | 0.005314395 | 97.97605508 |
|  |  | rs720439 | G | A | 0.7485 | 0.111927 | 0.0251252 | 7.03E-06 | -0.00458972 | 0.0174377 | 0.792392 | 0.004716605 | 86.90299149 |
|  |  | rs73199919 | T | C | 0.0567 | -0.236717 | 0.0531335 | 8.16E-06 | -0.0167198 | 0.0348512 | 0.631406 | 0.00599407 | 110.5820861 |
|  |  | rs9613196 | T | A | 0.0765 | -0.238718 | 0.0528561 | 4.99E-06 | 0.0320115 | 0.0374532 | 0.392714 | 0.008051905 | 148.8544032 |
| ebi-a-GCST90016999 | Gut microbiota abundance (genus Eubacterium fissicatena group id.14373) | rs10147907 | T | G | 0.0686 | 0.172263 | 0.039601 | 8.27E-06 | -0.0450489 | 0.0285022 | 0.113983 | 0.003792053 | 69.80335934 |
|  |  | rs11818408 | G | A | 0.4771 | 0.10585 | 0.0237114 | 8.20E-06 | -0.0455393 | 0.0152683 | 0.00285812 | 0.00559036 | 103.0923457 |
|  |  | rs11876297 | T | C | 0.2157 | 0.131469 | 0.0281712 | 2.67E-06 | -0.00859208 | 0.0169194 | 0.611577 | 0.005848023 | 107.8718837 |
|  |  | rs151257695 | A | G | 0.0805 | 0.20951 | 0.0454847 | 3.10E-06 | -0.0013951 | 0.0291516 | 0.96183 | 0.006498111 | 119.9417537 |
|  |  | rs1768152 | C | T | 0.8628 | -0.139489 | 0.0316186 | 8.70E-06 | 0.00381346 | 0.0244329 | 0.875971 | 0.004606533 | 84.86553382 |
|  |  | rs2733072 | G | A | 0.4652 | 0.109644 | 0.0228306 | 1.49E-06 | -0.0187195 | 0.015006 | 0.212226 | 0.005981786 | 110.3540987 |
|  |  | rs3771393 | T | C | 0.7157 | -0.130842 | 0.0266671 | 7.38E-07 | 0.0376375 | 0.0189086 | 0.0465361 | 0.006966782 | 128.6531466 |
|  |  | rs6934739 | A | G | 0.2952 | 0.111463 | 0.0252788 | 9.75E-06 | -0.0263745 | 0.0158731 | 0.0965962 | 0.005169799 | 95.29644667 |
|  |  | rs7104872 | G | A | 0.1779 | 0.138612 | 0.0291906 | 2.73E-06 | -0.0272584 | 0.0238715 | 0.253505 | 0.005619947 | 103.6410529 |
| ebi-a-GCST90017000 | Gut microbiota abundance (genus Eubacterium hallii group id.11338) | rs10501370 | C | T | 0.0527 | -0.115589 | 0.0252489 | 5.42E-06 | -0.0119592 | 0.0308182 | 0.697974 | 0.001334016 | 24.49587021 |
|  |  | rs10798999 | C | T | 0.2157 | 0.0601661 | 0.0126752 | 2.61E-06 | -0.0015551 | 0.0169212 | 0.926776 | 0.001224803 | 22.4879723 |
|  |  | rs117748144 | T | C | 0.0427 | -0.126582 | 0.028712 | 7.86E-06 | 0.0581397 | 0.033891 | 0.0862561 | 0.001309935 | 24.05310101 |
|  |  | rs13116360 | T | C | 0.0626 | 0.154124 | 0.0297185 | 2.94E-07 | 0.0144305 | 0.0301818 | 0.632566 | 0.002787853 | 51.26656623 |
|  |  | rs17074066 | T | C | 0.0915 | -0.0813986 | 0.0189272 | 9.35E-06 | -0.0084549 | 0.0534331 | 0.874273 | 0.001101564 | 20.22276465 |
|  |  | rs17474256 | G | A | 0.1233 | 0.0810805 | 0.0184573 | 9.45E-06 | 0.0337107 | 0.0252025 | 0.181029 | 0.001421271 | 26.10036452 |
|  |  | rs281379 | A | G | 0.4602 | -0.0499523 | 0.0112153 | 9.33E-06 | -0.0347879 | 0.0151986 | 0.0220861 | 0.001239711 | 22.76203944 |
|  |  | rs28584818 | A | G | 0.0686 | 0.126115 | 0.0268627 | 4.43E-06 | -0.0570415 | 0.0277249 | 0.0396461 | 0.002032469 | 37.34731545 |
|  |  | rs60254196 | G | A | 0.4592 | 0.0522832 | 0.0111865 | 2.70E-06 | -0.013978 | 0.01504 | 0.352685 | 0.001357666 | 24.93072311 |
|  |  | rs630939 | C | T | 0.3996 | -0.0508914 | 0.0114352 | 9.16E-06 | 0.0321214 | 0.0151136 | 0.0335591 | 0.001242753 | 22.81796871 |
|  |  | rs6550770 | C | T | 0.9483 | 0.198087 | 0.0443545 | 4.82E-06 | -0.00458205 | 0.0372402 | 0.902075 | 0.003847497 | 70.82790179 |
|  |  | rs74018587 | C | T | 0.0368 | 0.208943 | 0.043822 | 3.70E-06 | 0.0148551 | 0.0382827 | 0.697988 | 0.003094924 | 56.93090678 |
|  |  | rs78056098 | G | T | 0.3608 | -0.0507411 | 0.0113758 | 8.29E-06 | -0.00540704 | 0.0155932 | 0.728774 | 0.001187553 | 21.80324171 |
|  |  | rs949971 | T | G | 0.3837 | -0.0540164 | 0.0116101 | 3.29E-06 | -0.00354515 | 0.0159417 | 0.824016 | 0.001379956 | 25.34060185 |
| ebi-a-GCST90017001 | Gut microbiota abundance (genus Eubacterium nodatum group id.11297) | rs10263623 | C | T | 0.1044 | 0.193496 | 0.0439023 | 8.91E-06 | -0.0401335 | 0.0383628 | 0.295489 | 0.007001459 | 129.2980337 |
|  |  | rs10458299 | T | C | 0.0805 | -0.187753 | 0.041969 | 8.37E-06 | 0.0100074 | 0.0282691 | 0.723336 | 0.005218568 | 96.20013421 |
|  |  | rs11006576 | A | G | 0.5129 | -0.110167 | 0.024593 | 7.99E-06 | 0.0228789 | 0.0150497 | 0.128455 | 0.006064345 | 111.8864691 |
|  |  | rs113893692 | C | T | 0.0984 | -0.185107 | 0.0403573 | 5.76E-06 | 0.0180962 | 0.0228556 | 0.428499 | 0.006079735 | 112.1721657 |
|  |  | rs34297067 | A | G | 0.1392 | -0.186907 | 0.0341475 | 6.60E-08 | -0.0371892 | 0.0211211 | 0.0782799 | 0.008371873 | 154.8195334 |
|  |  | rs61841040 | G | T | 0.166 | 0.160621 | 0.0341609 | 3.56E-06 | 0.0037408 | 0.0187494 | 0.84186 | 0.007143463 | 131.9393238 |
|  |  | rs6818880 | G | A | 0.5447 | 0.110081 | 0.0245964 | 7.83E-06 | 0.00235779 | 0.0150693 | 0.875667 | 0.006010488 | 110.8868177 |
|  |  | rs77910827 | C | T | 0.1034 | 0.201788 | 0.0413568 | 9.05E-07 | 0.0155206 | 0.0241845 | 0.52103 | 0.007549878 | 139.5028948 |
|  |  | rs7827125 | C | T | 0.2823 | 0.1223 | 0.0271331 | 7.17E-06 | -0.00859106 | 0.0166636 | 0.606162 | 0.006060895 | 111.8224298 |
|  |  | rs7880204 | T | C | 0.2475 | -0.125454 | 0.0275303 | 6.84E-06 | 0.0027371 | 0.0173883 | 0.874922 | 0.005862471 | 108.139966 |
|  |  | rs9425984 | T | C | 0.2386 | -0.130234 | 0.0292347 | 7.21E-06 | -0.0163015 | 0.0180614 | 0.366762 | 0.006162573 | 113.7100073 |
| ebi-a-GCST90017003 | Gut microbiota abundance (genus Eubacterium rectale group id.14374) | rs10248854 | C | A | 0.3598 | -0.0527827 | 0.0113464 | 4.21E-06 | 0.0138344 | 0.0153052 | 0.366048 | 0.001283483 | 23.56675377 |
|  |  | rs10797540 | A | G | 0.4344 | 0.0503179 | 0.0108389 | 3.53E-06 | -0.00158621 | 0.0150439 | 0.916027 | 0.001244154 | 22.8437218 |
|  |  | rs10892089 | C | G | 0.1968 | -0.0635522 | 0.0140072 | 6.22E-06 | 0.0112845 | 0.0192401 | 0.557532 | 0.00127685 | 23.44481552 |
|  |  | rs143694765 | T | C | 0.0944 | 0.0870025 | 0.019779 | 9.75E-06 | -0.0400926 | 0.0249104 | 0.107513 | 0.001294201 | 23.7638206 |
|  |  | rs16960159 | C | G | 0.0477 | -0.156596 | 0.0336234 | 4.10E-06 | -0.0186171 | 0.0253251 | 0.462262 | 0.002227837 | 40.94530149 |
|  |  | rs2884897 | A | G | 0.0527 | -0.129364 | 0.0289021 | 6.44E-06 | 0.017171 | 0.0409453 | 0.674949 | 0.001670918 | 30.69257072 |
|  |  | rs314726 | T | C | 0.4573 | 0.0528824 | 0.0109486 | 1.38E-06 | -0.0161539 | 0.0150767 | 0.283966 | 0.001388076 | 25.48992475 |
|  |  | rs35398954 | A | G | 0.1213 | -0.0901402 | 0.0174602 | 5.40E-07 | 0.00711728 | 0.0203786 | 0.726901 | 0.001732082 | 31.81803182 |
|  |  | rs3980709 | A | T | 0.161 | -0.0622701 | 0.0140572 | 6.86E-06 | 0.0409757 | 0.0196929 | 0.0374585 | 0.001047555 | 19.23021382 |
|  |  | rs59427698 | A | G | 0.2028 | -0.0576035 | 0.0130853 | 5.37E-06 | 0.0136164 | 0.0191099 | 0.476134 | 0.001072909 | 19.69614159 |
|  |  | rs62547233 | A | G | 0.3072 | 0.0536319 | 0.0120201 | 9.90E-06 | 0.0165096 | 0.0165633 | 0.318881 | 0.00122435 | 22.47964633 |
| ebi-a-GCST90017004 | Gut microbiota abundance (genus Eubacterium ruminantium group id.11340) | rs10131724 | C | A | 0.9364 | 0.199832 | 0.0414577 | 2.39E-06 | 0.00247759 | 0.025408 | 0.92232 | 0.004756402 | 87.6397565 |
|  |  | rs10923018 | G | A | 0.4722 | 0.0726438 | 0.0160924 | 6.80E-06 | -0.011266 | 0.01513 | 0.456503 | 0.002630404 | 48.36356607 |
|  |  | rs112375806 | T | A | 0.1074 | 0.143141 | 0.0293757 | 5.82E-06 | -0.0204272 | 0.0216395 | 0.34518 | 0.003928432 | 72.323707 |
|  |  | rs11637981 | T | G | 0.5159 | 0.073258 | 0.0160888 | 5.44E-06 | -0.0151 | 0.0150548 | 0.315862 | 0.002680654 | 49.28995785 |
|  |  | rs13025464 | C | T | 0.6173 | 0.0737077 | 0.0163786 | 6.97E-06 | 0.0121118 | 0.0152828 | 0.428061 | 0.002566909 | 47.19311564 |
|  |  | rs139749 | C | T | 0.3419 | -0.0845439 | 0.0171791 | 8.59E-07 | -0.00386108 | 0.0158277 | 0.807275 | 0.003216515 | 59.17478348 |
|  |  | rs16891896 | G | A | 0.0586 | -0.174787 | 0.0390573 | 2.38E-06 | -0.0104538 | 0.026712 | 0.695538 | 0.0033707 | 62.02094506 |
|  |  | rs17519472 | C | T | 0.1541 | 0.107804 | 0.0233984 | 4.70E-06 | -9.52E-05 | 0.0217216 | 0.996501 | 0.003029852 | 55.73027986 |
|  |  | rs209813 | G | A | 0.166 | -0.103488 | 0.0236391 | 9.23E-06 | 0.00989159 | 0.0211943 | 0.640706 | 0.002965406 | 54.54134747 |
|  |  | rs2116427 | A | G | 0.2565 | 0.0911461 | 0.0182353 | 4.67E-07 | 0.0348983 | 0.0171283 | 0.0416035 | 0.003168652 | 58.2914422 |
|  |  | rs2229917 | A | G | 0.0666 | 0.153538 | 0.0323922 | 2.16E-06 | -0.0636486 | 0.0372266 | 0.0873092 | 0.002930919 | 53.9051798 |
|  |  | rs2418654 | C | T | 0.4781 | -0.0748879 | 0.0165852 | 6.17E-06 | 0.0204619 | 0.0152824 | 0.180596 | 0.002798719 | 51.46695586 |
|  |  | rs2817174 | C | T | 0.4294 | -0.0734306 | 0.0163687 | 7.87E-06 | 0.0150231 | 0.0153075 | 0.326387 | 0.002642275 | 48.58240042 |
|  |  | rs57340348 | T | C | 0.2147 | -0.0979429 | 0.0212166 | 4.93E-06 | -0.0123659 | 0.0186932 | 0.508281 | 0.003234771 | 59.51173943 |
|  |  | rs606117 | G | A | 0.668 | -0.0833243 | 0.018056 | 4.82E-06 | -0.0122013 | 0.0167919 | 0.467458 | 0.003079554 | 56.6473183 |
|  |  | rs6676699 | T | G | 0.7783 | 0.0888124 | 0.0196447 | 6.38E-06 | -0.0315468 | 0.0163972 | 0.0543651 | 0.002722011 | 50.05248758 |
|  |  | rs7000472 | G | A | 0.6382 | 0.0762282 | 0.016523 | 4.07E-06 | -0.0206778 | 0.0152141 | 0.174109 | 0.002683408 | 49.34073466 |
|  |  | rs72836424 | C | T | 0.0954 | -0.139825 | 0.030069 | 2.62E-06 | -0.0289058 | 0.0238782 | 0.226066 | 0.003374463 | 62.09041561 |
|  |  | rs73139629 | A | C | 0.1322 | -0.115098 | 0.0247909 | 5.36E-06 | 0.0171327 | 0.0259103 | 0.508465 | 0.003039602 | 55.91015701 |
| ebi-a-GCST90017007 | Gut microbiota abundance (genus Faecalibacterium id.2057) | rs10927394 | G | T | 0.0268 | -0.232262 | 0.0512364 | 7.02E-06 | 0.0415205 | 0.0550972 | 0.451097 | 0.002813994 | 51.7486478 |
|  |  | rs114946999 | C | T | 0.0984 | -0.0861565 | 0.01896 | 5.70E-06 | 0.0135898 | 0.022545 | 0.546653 | 0.001317089 | 24.18462975 |
|  |  | rs11776390 | T | C | 0.0895 | -0.078354 | 0.017183 | 6.40E-06 | -0.0487089 | 0.0300885 | 0.105479 | 0.001000588 | 18.36716221 |
|  |  | rs12320842 | C | G | 0.1322 | 0.094833 | 0.0163962 | 7.57E-09 | -0.00591869 | 0.0219611 | 0.787539 | 0.002063479 | 37.9183235 |
|  |  | rs1271565 | C | T | 0.2724 | -0.0576251 | 0.0119649 | 1.30E-06 | -0.0105312 | 0.0170212 | 0.536108 | 0.001316295 | 24.17002936 |
|  |  | rs12753492 | A | C | 0.1491 | 0.0641305 | 0.0149933 | 8.80E-06 | -0.0103034 | 0.023764 | 0.664601 | 0.001043555 | 19.15670582 |
|  |  | rs2835874 | T | C | 0.0696 | -0.0866356 | 0.0196496 | 7.54E-06 | 0.0240889 | 0.0401176 | 0.548201 | 0.000972079 | 17.84333603 |
|  |  | rs28376661 | C | G | 0.3738 | 0.0504817 | 0.0109368 | 3.66E-06 | 0.0214108 | 0.0162732 | 0.188273 | 0.001193027 | 21.90386237 |
|  |  | rs61875484 | C | G | 0.0905 | 0.0817953 | 0.018369 | 9.18E-06 | 0.00430671 | 0.0255359 | 0.866069 | 0.001101382 | 20.21941255 |
|  |  | rs6910935 | G | A | 0.9463 | -0.134864 | 0.0277032 | 1.38E-06 | -0.0312882 | 0.0312017 | 0.315972 | 0.001848524 | 33.96101876 |
|  |  | rs75499067 | C | T | 0.0636 | 0.227585 | 0.0465522 | 1.76E-06 | 0.0237346 | 0.0286864 | 0.408021 | 0.006169299 | 113.8348776 |
|  |  | rs79656633 | T | C | 0.1054 | 0.145631 | 0.0323019 | 8.14E-06 | 0.0294602 | 0.0251364 | 0.241192 | 0.003999513 | 73.63759255 |
|  |  | rs9536330 | T | C | 0.4085 | -0.048317 | 0.010797 | 5.33E-06 | -0.0319358 | 0.0150173 | 0.0334534 | 0.001128176 | 20.71185196 |
| ebi-a-GCST90017008 | Gut microbiota abundance (genus Family XIII AD3011 group id.11293) | rs11126423 | T | C | 0.8867 | -0.0904399 | 0.0196301 | 5.91E-06 | -0.0274635 | 0.0263063 | 0.296489 | 0.001643451 | 30.18721571 |
|  |  | rs11736617 | G | A | 0.1203 | -0.0759493 | 0.0172101 | 9.02E-06 | -0.0186712 | 0.0328615 | 0.569914 | 0.001220893 | 22.41611188 |
|  |  | rs12812672 | T | C | 0.0736 | -0.096065 | 0.020828 | 2.56E-06 | -0.00276875 | 0.0288531 | 0.923552 | 0.001258452 | 23.1065753 |
|  |  | rs12911842 | A | T | 0.1243 | -0.0811976 | 0.0183335 | 6.91E-06 | 0.0226736 | 0.0267521 | 0.396692 | 0.001435301 | 26.35837418 |
|  |  | rs149302 | T | C | 0.2127 | -0.0645635 | 0.0143225 | 7.48E-06 | 0.00662691 | 0.0177105 | 0.70827 | 0.001396085 | 25.63719886 |
|  |  | rs16840310 | G | A | 0.6282 | 0.0608052 | 0.0122149 | 6.75E-07 | -0.0311072 | 0.0151848 | 0.0405033 | 0.001727105 | 31.72644744 |
|  |  | rs16940167 | C | T | 0.169 | 0.0732552 | 0.0159898 | 3.91E-06 | -0.0414722 | 0.0190095 | 0.0291353 | 0.001507282 | 27.68227046 |
|  |  | rs17156849 | G | A | 0.0626 | -0.112891 | 0.0245292 | 4.19E-06 | 0.0479383 | 0.031729 | 0.130822 | 0.001495712 | 27.46944926 |
|  |  | rs62029761 | A | G | 0.0676 | 0.128753 | 0.0276002 | 3.89E-06 | 0.0215708 | 0.0329525 | 0.512723 | 0.002089747 | 38.40202749 |
|  |  | rs62200412 | C | T | 0.171 | -0.0800851 | 0.0163834 | 5.80E-07 | -0.00809531 | 0.0172068 | 0.638018 | 0.001818378 | 33.40615406 |
|  |  | rs72730932 | C | A | 0.164 | -0.0899559 | 0.0177107 | 6.89E-07 | 0.01888 | 0.0257207 | 0.462925 | 0.002218909 | 40.78083614 |
|  |  | rs739451 | C | T | 0.1759 | 0.0649589 | 0.0147534 | 7.88E-06 | 0.00185689 | 0.0184517 | 0.91984 | 0.001223357 | 22.46139183 |
|  |  | rs9276029 | A | G | 0.168 | -0.0811384 | 0.0185669 | 8.93E-06 | 0.0778107 | 0.0190591 | 4.45E-05 | 0.001840414 | 33.81173596 |
|  |  | rs9837139 | A | G | 0.0815 | 0.10752 | 0.0240482 | 8.71E-06 | 0.0284661 | 0.0268594 | 0.289226 | 0.001730794 | 31.79432214 |
|  |  | rs9852893 | G | C | 0.7435 | -0.0657834 | 0.0129056 | 3.88E-07 | 0.016489 | 0.0168555 | 0.327947 | 0.001650559 | 30.31798679 |
| ebi-a-GCST90017010 | Gut microbiota abundance (genus Flavonifractor id.2059) | rs114873521 | C | T | 0.0765 | -0.130067 | 0.0294087 | 7.13E-06 | -0.00402948 | 0.0291428 | 0.890029 | 0.002390356 | 43.9393782 |
|  |  | rs11642826 | G | C | 0.0636 | 0.14686 | 0.0325191 | 6.65E-06 | -0.0512876 | 0.0259383 | 0.0480087 | 0.002568949 | 47.23072896 |
|  |  | rs11811696 | T | C | 0.1103 | -0.116063 | 0.0241012 | 2.07E-06 | -0.0107906 | 0.0272953 | 0.692601 | 0.002643849 | 48.6114282 |
|  |  | rs12030302 | G | A | 0.5089 | 0.0692276 | 0.0137467 | 5.61E-07 | 0.0142374 | 0.0149927 | 0.342302 | 0.002395471 | 44.03362994 |
|  |  | rs12038887 | C | G | 0.1093 | 0.0942719 | 0.0211646 | 9.37E-06 | 0.00792667 | 0.0220323 | 0.719015 | 0.001730399 | 31.78705198 |
|  |  | rs34066017 | A | G | 0.2455 | 0.0764294 | 0.0159789 | 1.52E-06 | -0.0110859 | 0.0185561 | 0.550223 | 0.002164022 | 39.76989517 |
|  |  | rs6761463 | C | G | 0.835 | 0.0833919 | 0.0184596 | 8.11E-06 | 0.013855 | 0.021749 | 0.524097 | 0.001916232 | 35.20733309 |
|  |  | rs806808 | C | T | 0.5239 | -0.0667272 | 0.0136557 | 1.18E-06 | -0.0125352 | 0.0151088 | 0.406731 | 0.002221173 | 40.82254372 |
| ebi-a-GCST90017013 | Gut microbiota abundance (genus Haemophilus id.3698) | rs10781340 | A | G | 0.834 | -0.0948915 | 0.0203221 | 4.32E-06 | 0.0136175 | 0.0226909 | 0.548421 | 0.002493209 | 45.83474985 |
|  |  | rs111582866 | G | A | 0.1014 | -0.124265 | 0.026016 | 1.27E-06 | 0.0223591 | 0.0265012 | 0.398838 | 0.002814051 | 51.74969939 |
|  |  | rs12191680 | C | G | 0.165 | 0.106544 | 0.0200323 | 1.47E-07 | 0.0414789 | 0.0248326 | 0.0948528 | 0.00312794 | 57.54014539 |
|  |  | rs12876183 | T | A | 0.2753 | 0.0749201 | 0.016711 | 9.62E-06 | 0.000127135 | 0.0157906 | 0.993576 | 0.002239707 | 41.16393813 |
|  |  | rs35509 | G | A | 0.0785 | 0.128249 | 0.0268779 | 2.01E-06 | -0.0174329 | 0.0373159 | 0.640379 | 0.002379595 | 43.74109105 |
|  |  | rs4822728 | T | C | 0.4374 | 0.0705861 | 0.0151388 | 3.48E-06 | 0.01051 | 0.0149417 | 0.481805 | 0.002452149 | 45.07804859 |
|  |  | rs56310940 | G | C | 0.0825 | -0.108393 | 0.0247052 | 7.23E-06 | 0.0257811 | 0.0226955 | 0.255974 | 0.001778658 | 32.67515133 |
|  |  | rs76022354 | C | T | 0.0328 | 0.244638 | 0.0505504 | 1.83E-06 | -0.0312682 | 0.0344624 | 0.36424 | 0.003797239 | 69.89919752 |
|  |  | rs78909003 | T | C | 0.0497 | -0.246256 | 0.0503924 | 1.67E-06 | -0.033352 | 0.0329506 | 0.31145 | 0.005728234 | 105.6495415 |
|  |  | rs9328464 | T | C | 0.492 | 0.0723095 | 0.0148799 | 1.42E-06 | 0.00411678 | 0.0150208 | 0.784029 | 0.002613663 | 48.05494465 |
|  |  | rs9382510 | C | T | 0.2455 | -0.093521 | 0.0172649 | 7.12E-08 | 0.0186942 | 0.0171228 | 0.274932 | 0.003240105 | 59.61017993 |
|  |  | rs9574096 | A | T | 0.3618 | -0.0736375 | 0.0155227 | 2.18E-06 | -0.01004 | 0.0154644 | 0.51619 | 0.00250411 | 46.03565037 |
|  |  | rs9895850 | T | C | 0.0497 | -0.192957 | 0.0416755 | 2.14E-06 | 0.0129611 | 0.0364378 | 0.722061 | 0.003516966 | 64.72174975 |
| ebi-a-GCST90017014 | Gut microbiota abundance (genus Holdemanella id.11393) | rs12415649 | G | C | 0.2376 | 0.0839384 | 0.0190567 | 7.88E-06 | -0.0110453 | 0.0180755 | 0.541157 | 0.002552588 | 46.92914623 |
|  |  | rs12513188 | G | A | 0.2217 | 0.0903906 | 0.0195276 | 4.65E-06 | -0.0263676 | 0.0168988 | 0.118684 | 0.002819611 | 51.85223699 |
|  |  | rs17586763 | T | C | 0.0179 | -0.227285 | 0.0510135 | 7.72E-06 | 0.0929417 | 0.0347507 | 0.00748342 | 0.001816269 | 33.36735398 |
|  |  | rs1830029 | G | C | 0.8201 | 0.0954096 | 0.021093 | 5.35E-06 | 0.0157145 | 0.0178989 | 0.379967 | 0.002686038 | 49.38922261 |
|  |  | rs1926302 | G | A | 0.1531 | -0.107972 | 0.0231421 | 7.50E-06 | 0.0119948 | 0.0179859 | 0.504836 | 0.003023149 | 55.60662099 |
|  |  | rs34187114 | C | A | 0.166 | -0.104505 | 0.022601 | 5.13E-06 | -0.0308334 | 0.0236946 | 0.193162 | 0.003023976 | 55.62186255 |
|  |  | rs35228298 | G | A | 0.2465 | 0.0934832 | 0.0202886 | 7.30E-06 | -0.00777311 | 0.0204318 | 0.703618 | 0.003246365 | 59.7257286 |
|  |  | rs4541991 | T | C | 0.2356 | -0.0927334 | 0.0194435 | 2.10E-06 | 0.00557315 | 0.0160044 | 0.727671 | 0.003097407 | 56.97673639 |
|  |  | rs607782 | C | T | 0.6183 | 0.0854214 | 0.0172515 | 7.19E-07 | 0.0161712 | 0.0155107 | 0.297144 | 0.003444172 | 63.37750043 |
|  |  | rs62113381 | T | C | 0.159 | -0.105447 | 0.0232029 | 5.54E-06 | 0.00914439 | 0.0221748 | 0.680063 | 0.002973662 | 54.69365035 |
|  |  | rs73011279 | T | C | 0.2078 | -0.0961669 | 0.0199336 | 1.36E-06 | -0.0214998 | 0.0178553 | 0.228546 | 0.00304482 | 56.00643692 |
|  |  | rs75764681 | T | C | 0.0626 | -0.283103 | 0.0598999 | 1.94E-06 | 0.0115202 | 0.0365242 | 0.752447 | 0.009406287 | 174.1304098 |
|  |  | rs761624 | G | C | 0.6918 | -0.0959801 | 0.0179537 | 1.38E-07 | 0.00803258 | 0.0162628 | 0.621361 | 0.003928308 | 72.32142166 |
|  |  | rs8113760 | G | A | 0.3519 | 0.078999 | 0.01734 | 4.62E-06 | 0.00894822 | 0.0161845 | 0.580341 | 0.002846653 | 52.35094053 |
| ebi-a-GCST90017017 | Gut microbiota abundance (genus Hungatella id.11306) | rs10044993 | A | C | 0.8827 | -0.139547 | 0.0316748 | 8.07E-06 | 0.0251212 | 0.0268277 | 0.349071 | 0.004032572 | 74.24872085 |
|  |  | rs13128780 | T | C | 0.1451 | -0.149725 | 0.0312777 | 1.75E-06 | 0.0155159 | 0.019115 | 0.416955 | 0.005561621 | 102.5593973 |
|  |  | rs13249325 | T | G | 0.3668 | -0.100023 | 0.0225883 | 9.69E-06 | -0.020776 | 0.0149748 | 0.165321 | 0.004647292 | 85.61994558 |
|  |  | rs17092615 | G | A | 0.1243 | 0.152235 | 0.0337867 | 7.38E-06 | 0.045223 | 0.0216888 | 0.0370612 | 0.005045283 | 92.98955085 |
|  |  | rs72759041 | G | T | 0.2346 | -0.126025 | 0.0282242 | 3.86E-06 | -0.00728545 | 0.0184668 | 0.693201 | 0.005703742 | 105.1952275 |
| ebi-a-GCST90017020 | Gut microbiota abundance (genus Lachnoclostridium id.11308) | rs1031599 | T | G | 0.9155 | 0.078627 | 0.0175644 | 6.31E-06 | -0.0522592 | 0.0304916 | 0.0865486 | 0.000956508 | 17.55723158 |
|  |  | rs12566975 | T | C | 0.493 | -0.0468097 | 0.0105787 | 9.57E-06 | 0.0155112 | 0.0149979 | 0.301032 | 0.001095359 | 20.10872465 |
|  |  | rs1528479 | A | G | 0.6302 | 0.0497799 | 0.0111919 | 9.64E-06 | -0.0233377 | 0.0154843 | 0.131763 | 0.001155004 | 21.20494796 |
|  |  | rs1997204 | C | T | 0.9473 | 0.108075 | 0.0242022 | 5.97E-06 | -0.0114537 | 0.0360771 | 0.750881 | 0.001166215 | 21.41102119 |
|  |  | rs2385421 | A | G | 0.1044 | 0.0746186 | 0.0180734 | 7.14E-06 | 0.0179923 | 0.0231541 | 0.437119 | 0.001041211 | 19.11362972 |
|  |  | rs3821998 | C | A | 0.0885 | -0.0864066 | 0.0192519 | 6.72E-06 | 0.0194863 | 0.0244945 | 0.4263 | 0.001204547 | 22.11562331 |
|  |  | rs4738679 | A | G | 0.6352 | 0.0520267 | 0.011404 | 4.42E-06 | -0.00169214 | 0.0153561 | 0.912256 | 0.001254434 | 23.03270678 |
|  |  | rs6112314 | A | C | 0.3966 | -0.0561715 | 0.0108174 | 2.43E-07 | -0.00943628 | 0.0158332 | 0.551187 | 0.00151015 | 27.73501263 |
|  |  | rs615997 | T | C | 0.499 | 0.0511752 | 0.0106491 | 2.03E-06 | 0.0208864 | 0.0149157 | 0.161425 | 0.001309445 | 24.04409251 |
|  |  | rs62285313 | A | G | 0.0855 | 0.0864203 | 0.0181565 | 1.58E-06 | 0.00876535 | 0.0253955 | 0.729978 | 0.001167915 | 21.4422741 |
|  |  | rs72829893 | G | T | 0.0656 | 0.117472 | 0.0268103 | 5.58E-06 | -0.011696 | 0.02454 | 0.633642 | 0.001691747 | 31.07582717 |
|  |  | rs78068103 | A | G | 0.0915 | 0.0886199 | 0.0194248 | 3.67E-06 | -0.0254887 | 0.0233872 | 0.275777 | 0.001305685 | 23.97496178 |
|  |  | rs789029 | C | T | 0.1889 | -0.0641288 | 0.0137974 | 3.75E-06 | -0.00791164 | 0.0215204 | 0.713147 | 0.001260209 | 23.13887271 |
| ebi-a-GCST90017023 | Gut microbiota abundance (genus Lachnospiraceae ND3007 group id.11317) | rs2861203 | G | A | 0.3191 | 0.0572304 | 0.0127279 | 7.37E-06 | -0.0106623 | 0.0163887 | 0.515313 | 0.001423291 | 26.13751125 |
|  |  | rs72776675 | T | C | 0.175 | -0.0647067 | 0.014797 | 8.72E-06 | -0.0137084 | 0.0199708 | 0.492448 | 0.001208984 | 22.19718171 |
|  |  | rs9932954 | A | G | 0.4284 | -0.0561864 | 0.0115984 | 1.25E-06 | -0.0112564 | 0.0158294 | 0.477016 | 0.001546088 | 28.39605683 |
| ebi-a-GCST90017026 | Gut microbiota abundance (genus Lachnospiraceae UCG004 id.11324) | rs11128180 | A | G | 0.2237 | 0.0648288 | 0.0140126 | 4.52E-06 | 0.00050708 | 0.0176903 | 0.977132 | 0.001459693 | 26.80698057 |
|  |  | rs12072562 | T | C | 0.0517 | 0.133104 | 0.0303566 | 7.07E-06 | -0.0352063 | 0.0383591 | 0.35872 | 0.001737195 | 31.91211452 |
|  |  | rs12673420 | G | A | 0.4414 | 0.0554351 | 0.0118375 | 2.98E-06 | -0.00661804 | 0.0149447 | 0.657885 | 0.00151542 | 27.83194339 |
|  |  | rs12747809 | A | G | 0.66 | 0.062197 | 0.0125685 | 8.65E-07 | 0.00263639 | 0.0165989 | 0.873803 | 0.001736168 | 31.893219 |
|  |  | rs12894272 | G | A | 0.6998 | -0.0579792 | 0.0125229 | 4.34E-06 | -0.00844367 | 0.0157773 | 0.592525 | 0.001412404 | 25.93730568 |
|  |  | rs233486 | G | A | 0.8757 | 0.0799088 | 0.017766 | 6.28E-06 | 0.00152598 | 0.021519 | 0.943467 | 0.001390099 | 25.52711838 |
|  |  | rs2444793 | T | C | 0.5298 | 0.0542433 | 0.0118188 | 4.77E-06 | 0.0124384 | 0.0153157 | 0.416714 | 0.001465942 | 26.92190988 |
|  |  | rs2706242 | G | C | 0.0934 | -0.0901399 | 0.0199712 | 9.84E-06 | -0.0592684 | 0.0267415 | 0.026668 | 0.001376026 | 25.26833995 |
|  |  | rs2726805 | A | G | 0.4085 | 0.0548397 | 0.0120844 | 6.30E-06 | -0.0103088 | 0.0151329 | 0.495731 | 0.001453339 | 26.6901215 |
|  |  | rs2882478 | G | A | 0.4553 | -0.0577091 | 0.0118338 | 1.21E-06 | 0.0107165 | 0.0150484 | 0.476378 | 0.001651861 | 30.34195639 |
|  |  | rs35182105 | A | G | 0.0726 | -0.109724 | 0.024221 | 4.87E-06 | -0.0573103 | 0.0329805 | 0.082264 | 0.001621201 | 29.77786725 |
|  |  | rs6656451 | T | C | 0.5606 | 0.05436 | 0.011947 | 5.57E-06 | 0.00836554 | 0.0149677 | 0.576225 | 0.001455801 | 26.73540167 |
|  |  | rs7629954 | A | G | 0.0686 | 0.108441 | 0.023825 | 5.77E-06 | -0.0417565 | 0.0360296 | 0.246476 | 0.001502718 | 27.59830779 |
| ebi-a-GCST90017027 | Gut microbiota abundance (genus Lachnospiraceae UCG008 id.11328) | rs10741777 | T | C | 0.2614 | -0.0973842 | 0.0194791 | 7.69E-07 | 0.00199948 | 0.0161821 | 0.901663 | 0.00366203 | 67.40112897 |
|  |  | rs10793103 | T | C | 0.6958 | -0.0974344 | 0.0181279 | 9.35E-08 | -0.00934764 | 0.0150469 | 0.534445 | 0.004018817 | 73.99444126 |
|  |  | rs10801803 | G | A | 0.162 | -0.117029 | 0.0243085 | 1.40E-06 | 0.0253641 | 0.0214233 | 0.236433 | 0.00371857 | 68.44566543 |
|  |  | rs13024781 | T | C | 0.503 | -0.0798769 | 0.0168846 | 2.29E-06 | -0.00981865 | 0.0150164 | 0.513202 | 0.003190045 | 58.68625205 |
|  |  | rs57091572 | A | G | 0.1402 | -0.110443 | 0.0235801 | 2.86E-06 | 0.0185352 | 0.0221237 | 0.402143 | 0.002940708 | 54.08574585 |
|  |  | rs61944774 | A | G | 0.0577 | 0.179838 | 0.0393797 | 6.34E-06 | -0.0497183 | 0.0335259 | 0.138079 | 0.003516883 | 64.72021502 |
|  |  | rs62277846 | C | T | 0.2356 | 0.102292 | 0.0212319 | 1.59E-06 | 0.00939235 | 0.0189881 | 0.62085 | 0.003768854 | 69.37470559 |
|  |  | rs67078837 | T | C | 0.4583 | -0.0845848 | 0.0170693 | 7.68E-07 | -0.00523616 | 0.0151611 | 0.729817 | 0.003552412 | 65.37637713 |
|  |  | rs75356640 | G | A | 0.0885 | 0.136523 | 0.0303128 | 9.83E-06 | 0.0108504 | 0.0228494 | 0.634882 | 0.003007056 | 55.3097212 |
|  |  | rs955844 | A | C | 0.1998 | 0.112064 | 0.0228369 | 1.81E-06 | -0.010186 | 0.0215202 | 0.635984 | 0.004015654 | 73.93596106 |
|  |  | rs9873555 | G | C | 0.1571 | -0.121162 | 0.0233467 | 2.41E-07 | -0.00131896 | 0.0232829 | 0.954824 | 0.0038879 | 71.57458762 |
| ebi-a-GCST90017030 | Gut microbiota abundance (genus Lactobacillus id.1837) | rs12693845 | C | T | 0.3569 | -0.0805447 | 0.0177429 | 8.96E-06 | -0.0125419 | 0.015432 | 0.416378 | 0.002978029 | 54.7742235 |
|  |  | rs1530559 | G | A | 0.5676 | 0.0804001 | 0.0178207 | 4.93E-06 | 0.0230764 | 0.0150843 | 0.12606 | 0.003173009 | 58.37184559 |
|  |  | rs16861661 | G | A | 0.0497 | -0.183147 | 0.038148 | 1.28E-06 | 0.0308403 | 0.0303926 | 0.310235 | 0.003168449 | 58.28770085 |
|  |  | rs62314653 | C | A | 0.0626 | 0.187692 | 0.0394585 | 2.24E-06 | 0.0211438 | 0.0317947 | 0.506045 | 0.004134479 | 76.13284763 |
|  |  | rs7399658 | G | A | 0.2157 | -0.107134 | 0.0221883 | 3.12E-06 | 0.0213777 | 0.019514 | 0.273293 | 0.003883444 | 71.49222377 |
|  |  | rs768253 | T | G | 0.3907 | -0.079195 | 0.0171791 | 4.25E-06 | 0.0265497 | 0.0150595 | 0.0779022 | 0.002986071 | 54.92257036 |
|  |  | rs77478751 | A | G | 0.0537 | -0.219886 | 0.0475759 | 7.33E-06 | 0.0107621 | 0.0233212 | 0.64446 | 0.004913922 | 90.55649354 |
|  |  | rs921925 | A | C | 0.2624 | 0.0985077 | 0.0203229 | 9.72E-07 | 0.00731558 | 0.0182518 | 0.688557 | 0.003756255 | 69.14192289 |
| ebi-a-GCST90017032 | Gut microbiota abundance (genus Marvinbryantia id.2005) | rs11620597 | T | C | 0.0527 | 0.119479 | 0.0271688 | 7.80E-06 | 0.00407374 | 0.0479944 | 0.932357 | 0.001425316 | 26.17476091 |
|  |  | rs1187983 | C | T | 0.1223 | -0.0935456 | 0.0193174 | 2.02E-06 | -0.0258596 | 0.0244575 | 0.290361 | 0.001878665 | 34.51579734 |
|  |  | rs146541147 | G | A | 0.0706 | 0.118845 | 0.0268423 | 6.86E-06 | 0.0642636 | 0.0418171 | 0.124348 | 0.001853528 | 34.05311831 |
|  |  | rs2724813 | G | A | 0.7992 | 0.0840769 | 0.0167551 | 6.28E-07 | 0.0149517 | 0.0175609 | 0.394535 | 0.002268833 | 41.70047384 |
|  |  | rs2842896 | C | T | 0.5278 | -0.0649396 | 0.0131146 | 7.25E-07 | 0.0161639 | 0.015246 | 0.28905 | 0.002102057 | 38.62872946 |
|  |  | rs2863363 | G | A | 0.6322 | -0.0634861 | 0.0136323 | 3.11E-06 | 0.0152746 | 0.0173654 | 0.379077 | 0.001874362 | 34.43660009 |
|  |  | rs3125832 | A | C | 0.2624 | 0.0679323 | 0.015012 | 5.03E-06 | 0.0476177 | 0.018028 | 0.00825829 | 0.001786353 | 32.81677033 |
|  |  | rs61884471 | G | A | 0.0845 | 0.124426 | 0.0248431 | 1.01E-06 | -0.0061086 | 0.0239825 | 0.798946 | 0.002395341 | 44.03123152 |
|  |  | rs72948274 | A | C | 0.0577 | -0.126354 | 0.0272212 | 3.26E-06 | -0.0699681 | 0.030553 | 0.0220181 | 0.001736093 | 31.89184092 |
|  |  | rs8006832 | G | T | 0.1113 | -0.0952415 | 0.0216699 | 6.58E-06 | 0.000296021 | 0.0258086 | 0.990849 | 0.001794456 | 32.96588842 |
| ebi-a-GCST90017033 | Gut microbiota abundance (genus Methanobrevibacter id.123) | rs10202904 | G | T | 0.5328 | 0.112811 | 0.0239106 | 3.09E-06 | 0.00541439 | 0.0152871 | 0.723204 | 0.006335778 | 116.9263141 |
|  |  | rs11018665 | A | T | 0.341 | 0.113013 | 0.0254343 | 7.03E-06 | -0.0129026 | 0.017481 | 0.460459 | 0.005740194 | 105.8714064 |
|  |  | rs1334944 | T | C | 0.2853 | 0.115197 | 0.0255492 | 7.61E-06 | 0.000467384 | 0.016691 | 0.97766 | 0.005411752 | 99.7806969 |
|  |  | rs4779844 | C | G | 0.6759 | -0.109639 | 0.0247979 | 9.28E-06 | 0.0347024 | 0.0157462 | 0.027534 | 0.005266494 | 97.08828428 |
|  |  | rs4802933 | G | A | 0.8221 | 0.135628 | 0.0308139 | 9.74E-06 | -0.0228314 | 0.0177585 | 0.198562 | 0.005380583 | 99.20289407 |
|  |  | rs6776814 | T | C | 0.0895 | -0.188956 | 0.0419905 | 8.05E-06 | -0.0645808 | 0.0528799 | 0.221983 | 0.00581908 | 107.334886 |
|  |  | rs76029318 | T | C | 0.0646 | 0.222849 | 0.0454319 | 1.08E-06 | -0.0100264 | 0.0308017 | 0.74479 | 0.006001796 | 110.7254942 |
|  |  | rs894996 | C | A | 0.0716 | 0.214213 | 0.0456045 | 3.82E-06 | -0.0376031 | 0.0292061 | 0.197917 | 0.006100561 | 112.5587651 |
| ebi-a-GCST90017036 | Gut microbiota abundance (genus Oscillibacter id.2063) | rs11627628 | T | C | 0.0726 | 0.143961 | 0.0290223 | 1.01E-06 | 0.0178654 | 0.0283219 | 0.528172 | 0.002790766 | 51.32028913 |
|  |  | rs11990279 | T | C | 0.2396 | -0.0824934 | 0.0180457 | 4.94E-06 | 0.00450894 | 0.0186834 | 0.809296 | 0.00247969 | 45.58558643 |
|  |  | rs12417956 | C | G | 0.2445 | 0.0785431 | 0.0174075 | 6.03E-06 | 0.0189345 | 0.0166806 | 0.256325 | 0.002279079 | 41.88922196 |
|  |  | rs12649930 | T | G | 0.0944 | 0.121589 | 0.0259612 | 4.09E-06 | 0.0404576 | 0.0247282 | 0.10182 | 0.002527708 | 46.4705813 |
|  |  | rs133832 | A | C | 0.3131 | -0.0795527 | 0.0162411 | 1.15E-06 | -0.0198875 | 0.0166509 | 0.232328 | 0.002722177 | 50.05554968 |
|  |  | rs137917150 | T | A | 0.0616 | -0.174992 | 0.0388394 | 4.62E-06 | 0.0307339 | 0.0263231 | 0.242983 | 0.003540259 | 65.15193341 |
|  |  | rs16866406 | A | G | 0.1392 | 0.0988765 | 0.0208795 | 3.08E-06 | 0.0134112 | 0.020822 | 0.519517 | 0.002342921 | 43.0653855 |
|  |  | rs16934185 | A | G | 0.1044 | -0.129568 | 0.028157 | 4.38E-06 | -0.012481 | 0.0246435 | 0.612532 | 0.003139353 | 57.75074696 |
|  |  | rs234108 | A | G | 0.4801 | 0.0749553 | 0.0152632 | 9.16E-07 | -0.00995757 | 0.0152415 | 0.51355 | 0.002804699 | 51.57722325 |
|  |  | rs36095275 | C | T | 0.3618 | -0.0752368 | 0.0156861 | 1.40E-06 | -0.00263558 | 0.0152319 | 0.862628 | 0.002614063 | 48.06231851 |
|  |  | rs4506202 | G | A | 0.4841 | 0.0711323 | 0.0152261 | 3.21E-06 | -0.00459733 | 0.0150105 | 0.759395 | 0.002527344 | 46.46385916 |
|  |  | rs61883564 | A | G | 0.1243 | -0.101351 | 0.0221016 | 3.39E-06 | 0.0407528 | 0.0217498 | 0.0609705 | 0.00223621 | 41.09952283 |
|  |  | rs6901560 | C | G | 0.1988 | 0.0855032 | 0.018693 | 6.21E-06 | -0.00380813 | 0.0191761 | 0.842586 | 0.002328907 | 42.8071814 |
|  |  | rs75453768 | G | T | 0.0865 | 0.12212 | 0.0268629 | 5.35E-06 | 0.00955219 | 0.025416 | 0.70704 | 0.00235683 | 43.32164915 |
|  |  | rs761240 | G | T | 0.9334 | 0.17664 | 0.0388812 | 2.04E-06 | -0.00204426 | 0.0351732 | 0.953653 | 0.003879271 | 71.41511242 |
|  |  | rs9393920 | G | A | 0.5646 | 0.0744662 | 0.0151081 | 9.92E-07 | 0.0281525 | 0.0153666 | 0.0669422 | 0.002726325 | 50.13203018 |
| ebi-a-GCST90017037 | Gut microbiota abundance (genus Oscillospira id.2064) | rs12206468 | G | A | 0.0636 | -0.133016 | 0.0269733 | 1.04E-06 | -0.0192906 | 0.0279607 | 0.490246 | 0.002107445 | 38.72794652 |
|  |  | rs12925026 | T | C | 0.0726 | 0.13559 | 0.030678 | 9.31E-06 | 0.0538832 | 0.032402 | 0.0963208 | 0.002475649 | 45.51111666 |
|  |  | rs1954532 | C | T | 0.7913 | 0.0826246 | 0.0175252 | 2.27E-06 | -0.0461681 | 0.0187309 | 0.0137085 | 0.002254822 | 41.44237932 |
|  |  | rs28889936 | A | C | 0.1083 | 0.114051 | 0.0252838 | 3.37E-06 | 0.0392783 | 0.0253582 | 0.121397 | 0.002512323 | 46.18700945 |
|  |  | rs62422654 | C | T | 0.1471 | 0.0898456 | 0.0198088 | 6.47E-06 | 0.0319194 | 0.0183815 | 0.0824765 | 0.00202551 | 37.21919175 |
|  |  | rs72866977 | A | C | 0.0696 | -0.130569 | 0.0281674 | 5.63E-06 | -0.00460195 | 0.0278829 | 0.868908 | 0.002207949 | 40.57897022 |
|  |  | rs73038677 | T | A | 0.1819 | -0.08321 | 0.0168666 | 1.09E-06 | 0.0100213 | 0.0217327 | 0.644714 | 0.002060725 | 37.86761778 |
|  |  | rs751183 | C | T | 0.7545 | 0.0774322 | 0.0172216 | 6.85E-06 | -0.0174947 | 0.0193577 | 0.366122 | 0.002221181 | 40.82269022 |
|  |  | rs8076323 | A | G | 0.2575 | 0.0715392 | 0.0156539 | 5.61E-06 | -0.0208124 | 0.0159215 | 0.191147 | 0.001957005 | 35.95792009 |
| ebi-a-GCST90017042 | Gut microbiota abundance (genus Peptococcus id.2037) | rs10031059 | C | T | 0.7793 | 0.121167 | 0.0225844 | 1.24E-07 | -0.0218663 | 0.0176194 | 0.214593 | 0.005050167 | 93.08002695 |
|  |  | rs11001941 | G | A | 0.0676 | -0.195611 | 0.0392217 | 1.33E-06 | -0.0138633 | 0.0266306 | 0.602659 | 0.004823536 | 88.88272787 |
|  |  | rs11030569 | A | T | 0.0775 | -0.17402 | 0.0374133 | 3.13E-06 | -0.0196571 | 0.0226196 | 0.384832 | 0.004330085 | 79.75042115 |
|  |  | rs12069354 | C | T | 0.0666 | 0.16763 | 0.0379501 | 9.28E-06 | 0.0238834 | 0.0306806 | 0.436302 | 0.003493619 | 64.29058769 |
|  |  | rs2054133 | A | G | 0.5258 | -0.0895433 | 0.0188332 | 2.14E-06 | 0.00167671 | 0.0156966 | 0.914932 | 0.003998327 | 73.6156615 |
|  |  | rs34282744 | G | C | 0.0616 | 0.191797 | 0.0399832 | 1.84E-06 | 0.00791355 | 0.0276382 | 0.774628 | 0.004252872 | 78.32226397 |
|  |  | rs36121075 | A | G | 0.1282 | -0.14067 | 0.030628 | 6.99E-06 | 0.0135125 | 0.0201043 | 0.501509 | 0.004423213 | 81.47325489 |
|  |  | rs413827 | G | A | 0.2127 | 0.110229 | 0.0237523 | 3.30E-06 | -0.00988367 | 0.01749 | 0.572004 | 0.004069391 | 74.92941819 |
|  |  | rs5770862 | T | C | 0.0666 | 0.162018 | 0.0356813 | 3.22E-06 | -0.035749 | 0.0258193 | 0.166179 | 0.003263612 | 60.0440821 |
|  |  | rs6918730 | A | G | 0.9026 | -0.135311 | 0.0289742 | 1.15E-06 | 0.0637744 | 0.0313211 | 0.0417359 | 0.003219219 | 59.22469074 |
|  |  | rs7033353 | G | T | 0.5805 | -0.090152 | 0.018995 | 2.22E-06 | -0.0318156 | 0.0149927 | 0.0338322 | 0.003958357 | 72.87681582 |
|  |  | rs72850165 | T | C | 0.1083 | -0.134304 | 0.0300423 | 5.74E-06 | 0.0165587 | 0.0279468 | 0.553509 | 0.003483815 | 64.1095496 |
|  |  | rs74592222 | G | A | 0.1173 | 0.137957 | 0.0302965 | 8.55E-06 | 0.0348335 | 0.0233875 | 0.136381 | 0.003941201 | 72.55972197 |
|  |  | rs75754569 | C | G | 0.1103 | 0.181434 | 0.0319429 | 1.10E-08 | -0.0185293 | 0.0257853 | 0.472388 | 0.006460802 | 119.2486338 |
|  |  | rs7766680 | G | C | 0.2485 | 0.0976832 | 0.0214155 | 3.51E-06 | -0.0281407 | 0.017842 | 0.114746 | 0.003563897 | 65.58849171 |
|  |  | rs77681628 | C | T | 0.0586 | 0.200307 | 0.0387328 | 2.69E-07 | -0.0424903 | 0.0275693 | 0.123264 | 0.004426842 | 81.54040204 |
| ebi-a-GCST90017043 | Gut microbiota abundance (genus Phascolarctobacterium id.2168) | rs12618201 | A | G | 0.3469 | 0.0641657 | 0.0138202 | 3.38E-06 | 0.0241968 | 0.0150482 | 0.107846 | 0.001865606 | 34.27542113 |
|  |  | rs1264476 | G | T | 0.8002 | -0.0767335 | 0.0166041 | 4.30E-06 | 0.0175962 | 0.0191164 | 0.357324 | 0.001882756 | 34.5911063 |
|  |  | rs56069061 | G | A | 0.1014 | -0.111306 | 0.0230694 | 1.87E-06 | 0.00912756 | 0.0299244 | 0.760351 | 0.002257727 | 41.49589271 |
|  |  | rs56157888 | A | C | 0.17 | 0.0954864 | 0.0193976 | 1.09E-06 | -0.00448091 | 0.0181097 | 0.804574 | 0.002573002 | 47.30541952 |
|  |  | rs6427992 | C | G | 0.6074 | 0.0652458 | 0.0137508 | 2.09E-06 | -0.0373246 | 0.0154869 | 0.0159492 | 0.0020303 | 37.30738529 |
|  |  | rs74540770 | G | A | 0.0646 | -0.121014 | 0.0258621 | 3.60E-06 | 0.00815377 | 0.0277614 | 0.76898 | 0.001769828 | 32.51265143 |
|  |  | rs75882962 | T | C | 0.167 | 0.0968661 | 0.0190575 | 3.19E-07 | -0.0478333 | 0.0226392 | 0.0346139 | 0.002610569 | 47.99790743 |
|  |  | rs76124218 | C | G | 0.0517 | -0.159361 | 0.0344532 | 2.67E-06 | -0.0217237 | 0.0401638 | 0.588592 | 0.002490178 | 45.77888067 |
|  |  | rs7982713 | G | A | 0.2286 | 0.0726921 | 0.0163201 | 9.72E-06 | 0.00637929 | 0.0166911 | 0.702317 | 0.001863633 | 34.23910279 |
| ebi-a-GCST90017046 | Gut microbiota abundance (genus Rikenellaceae RC9 gut group id.11191) | rs12501673 | A | G | 0.325 | 0.116395 | 0.0262253 | 6.29E-06 | -0.0125147 | 0.0168918 | 0.458769 | 0.005944096 | 109.6546209 |
|  |  | rs17032291 | T | C | 0.1412 | -0.169579 | 0.0368251 | 6.61E-06 | -0.016365 | 0.0226136 | 0.469263 | 0.006974304 | 128.7930268 |
|  |  | rs17582787 | A | G | 0.1292 | -0.157739 | 0.0339924 | 3.55E-06 | -0.0109882 | 0.0200919 | 0.58445 | 0.005598724 | 103.2474637 |
|  |  | rs2074881 | T | C | 0.1789 | -0.142236 | 0.032391 | 9.45E-06 | 0.0229573 | 0.0222564 | 0.30231 | 0.00594368 | 109.6469175 |
|  |  | rs2900503 | T | G | 0.829 | 0.172329 | 0.0326671 | 1.55E-07 | 0.00503422 | 0.0202637 | 0.803798 | 0.008419715 | 155.7117757 |
|  |  | rs2998141 | C | T | 0.7783 | 0.136346 | 0.0293073 | 4.42E-06 | 0.0078234 | 0.0176402 | 0.657407 | 0.006415456 | 118.40626 |
|  |  | rs4270579 | A | G | 0.6958 | 0.118021 | 0.0271041 | 5.46E-06 | 0.00635753 | 0.0159976 | 0.691068 | 0.005896472 | 108.7708602 |
|  |  | rs4717843 | G | T | 0.3618 | -0.119376 | 0.0260548 | 4.72E-06 | 0.0200486 | 0.0150006 | 0.181378 | 0.006580962 | 121.4811496 |
|  |  | rs7193937 | C | G | 0.7107 | -0.124153 | 0.027741 | 6.19E-06 | -0.0165305 | 0.0157298 | 0.293303 | 0.006338393 | 116.9748885 |
|  |  | rs7712231 | A | G | 0.165 | 0.156284 | 0.0350462 | 7.97E-06 | -0.0103687 | 0.0220553 | 0.638265 | 0.006730223 | 124.2550931 |
|  |  | rs80309088 | G | A | 0.1292 | 0.17405 | 0.0383423 | 4.56E-06 | -0.0283519 | 0.0229387 | 0.216464 | 0.006816461 | 125.8581781 |
|  |  | rs9887954 | G | A | 0.4364 | -0.114849 | 0.0249013 | 4.81E-06 | 0.00358351 | 0.0152393 | 0.814092 | 0.006488438 | 119.7620444 |
| ebi-a-GCST90017049 | Gut microbiota abundance (genus Ruminiclostridium5 id.11355) | rs10827477 | A | G | 0.3151 | -0.0547387 | 0.0115163 | 2.19E-06 | -0.00590792 | 0.0156899 | 0.706514 | 0.001293286 | 23.74698741 |
|  |  | rs113753996 | T | C | 0.1372 | 0.0820689 | 0.0174463 | 3.99E-06 | -0.0216984 | 0.0193622 | 0.262435 | 0.001594599 | 29.28845852 |
|  |  | rs1223978 | C | T | 0.5199 | -0.0484169 | 0.0108254 | 8.16E-06 | -0.00401693 | 0.0150669 | 0.789772 | 0.001170241 | 21.48503043 |
|  |  | rs1492620 | T | C | 0.0805 | -0.0830505 | 0.0180073 | 3.53E-06 | 0.00888459 | 0.0224188 | 0.691884 | 0.001021086 | 18.74380508 |
|  |  | rs243585 | C | G | 0.3012 | -0.0585939 | 0.0121017 | 1.33E-06 | -0.00102966 | 0.0167744 | 0.951054 | 0.001445249 | 26.54133457 |
|  |  | rs2482038 | C | A | 0.4384 | 0.0518991 | 0.0108777 | 1.70E-06 | 0.00214001 | 0.015224 | 0.888211 | 0.001326317 | 24.35430028 |
|  |  | rs2791343 | T | C | 0.34 | 0.051717 | 0.0113369 | 5.54E-06 | 0.00293075 | 0.0152451 | 0.847553 | 0.001200382 | 22.03906155 |
|  |  | rs2801960 | G | C | 0.6819 | -0.0520975 | 0.0115111 | 6.21E-06 | 0.00229833 | 0.016388 | 0.888467 | 0.001177465 | 21.61781312 |
|  |  | rs2833828 | G | A | 0.4533 | 0.0489617 | 0.0108702 | 6.82E-06 | 0.0148496 | 0.0152085 | 0.328866 | 0.001188168 | 21.8145398 |
|  |  | rs4955951 | A | G | 0.0924 | -0.0713749 | 0.0165826 | 9.96E-06 | -0.00916665 | 0.0232912 | 0.6939 | 0.000854452 | 15.68233368 |
|  |  | rs6121460 | G | A | 0.0805 | 0.0932955 | 0.0199206 | 2.64E-06 | 0.0197723 | 0.0275505 | 0.47296 | 0.001288543 | 23.65979292 |
|  |  | rs73002572 | G | C | 0.0547 | 0.181548 | 0.0414078 | 8.82E-06 | -0.0635614 | 0.0238138 | 0.00760554 | 0.003408552 | 62.71980942 |
|  |  | rs79968837 | A | G | 0.0686 | -0.0950311 | 0.0193507 | 1.15E-06 | -0.0239027 | 0.0339217 | 0.481031 | 0.001154043 | 21.18728506 |
|  |  | rs8053158 | G | A | 0.8797 | 0.0740543 | 0.0159151 | 5.90E-06 | -0.0347624 | 0.022912 | 0.129214 | 0.001160729 | 21.31018091 |
| ebi-a-GCST90017052 | Gut microbiota abundance (genus Ruminococcaceae NK4A214 group id.11358) | rs11241747 | T | C | 0.6093 | -0.0533858 | 0.0120035 | 6.59E-06 | 0.0113124 | 0.01647 | 0.492177 | 0.001356926 | 24.91711572 |
|  |  | rs114244418 | C | G | 0.0408 | -0.175266 | 0.0372779 | 3.59E-06 | 0.000548134 | 0.03829 | 0.988578 | 0.002404333 | 44.19692898 |
|  |  | rs11586410 | G | A | 0.1342 | -0.0863422 | 0.0169937 | 3.66E-07 | -0.0226478 | 0.0206823 | 0.273503 | 0.001732393 | 31.82374625 |
|  |  | rs12642039 | C | T | 0.6342 | 0.0553003 | 0.0119397 | 3.43E-06 | -0.00501051 | 0.0155537 | 0.747345 | 0.00141891 | 26.05694762 |
|  |  | rs12731 | A | G | 0.4513 | -0.0527684 | 0.0115053 | 4.87E-06 | -0.00868014 | 0.0154196 | 0.573484 | 0.001379044 | 25.32383263 |
|  |  | rs13087692 | G | T | 0.6948 | -0.0574623 | 0.012594 | 8.69E-06 | -0.0205813 | 0.016356 | 0.20827 | 0.001400362 | 25.7158515 |
|  |  | rs136761 | A | G | 0.5706 | 0.0587521 | 0.0119155 | 8.15E-07 | -0.0137215 | 0.0155608 | 0.377887 | 0.001691495 | 31.07118301 |
|  |  | rs147475196 | A | G | 0.0775 | -0.133769 | 0.0295196 | 4.72E-06 | 0.0154678 | 0.0245 | 0.527819 | 0.002558639 | 47.04068413 |
|  |  | rs34576931 | G | C | 0.0915 | -0.0873312 | 0.0194886 | 4.72E-06 | 0.0485542 | 0.0294983 | 0.0997631 | 0.001267987 | 23.28187078 |
|  |  | rs35559912 | T | C | 0.0656 | -0.0925071 | 0.0203674 | 4.89E-06 | 0.0220393 | 0.0230201 | 0.338369 | 0.0010491 | 19.25859604 |
|  |  | rs4814689 | C | T | 0.0666 | -0.1083 | 0.0230708 | 4.55E-06 | 0.0502382 | 0.036372 | 0.167207 | 0.00145824 | 26.7802527 |
|  |  | rs5994253 | A | G | 0.1461 | -0.0811302 | 0.0157621 | 2.35E-07 | 0.000993044 | 0.0213877 | 0.962967 | 0.001642299 | 30.16602694 |
|  |  | rs62027366 | T | C | 0.2296 | 0.0615389 | 0.0137612 | 6.58E-06 | -0.00519601 | 0.0186399 | 0.780432 | 0.001339731 | 24.60094601 |
|  |  | rs6681678 | T | C | 0.9503 | 0.100174 | 0.024 | 9.05E-06 | 0.0146551 | 0.041493 | 0.723942 | 0.000947888 | 17.39886712 |
|  |  | rs73158814 | C | G | 0.0736 | -0.109257 | 0.0227458 | 2.20E-06 | 0.0294832 | 0.0404796 | 0.4664 | 0.001627814 | 29.89953226 |
|  |  | rs7573569 | T | C | 0.0716 | 0.107739 | 0.0233638 | 3.23E-06 | -0.0769952 | 0.031226 | 0.0136729 | 0.001543206 | 28.3430591 |
| ebi-a-GCST90017053 | Gut microbiota abundance (genus Ruminococcaceae UCG002 id.11360) | rs10916131 | C | T | 0.1382 | -0.069333 | 0.0146753 | 2.87E-06 | 0.012721 | 0.020308 | 0.531049 | 0.00114505 | 21.02200114 |
|  |  | rs10927423 | C | A | 0.1829 | -0.0713617 | 0.0147712 | 8.50E-07 | 0.00612336 | 0.0196 | 0.754724 | 0.001522121 | 27.95521311 |
|  |  | rs10964441 | G | A | 0.0527 | -0.14906 | 0.034486 | 7.45E-06 | -0.0562476 | 0.0245893 | 0.0221677 | 0.002218454 | 40.77245695 |
|  |  | rs113147300 | A | G | 0.1322 | -0.075842 | 0.0164562 | 7.69E-06 | 0.0114901 | 0.0217996 | 0.598139 | 0.001319777 | 24.23405949 |
|  |  | rs11607472 | A | G | 0.1074 | -0.0780229 | 0.0176325 | 7.19E-06 | 0.00509589 | 0.0298149 | 0.864289 | 0.001167173 | 21.42863452 |
|  |  | rs116974815 | C | A | 0.0577 | -0.189731 | 0.0396566 | 2.03E-06 | -0.0174344 | 0.0294137 | 0.55336 | 0.003914458 | 72.06542018 |
|  |  | rs11750293 | G | T | 0.2813 | -0.0578304 | 0.0120512 | 1.76E-06 | -0.000821035 | 0.0155601 | 0.957919 | 0.001352259 | 24.83129729 |
|  |  | rs12463378 | A | G | 0.4175 | -0.0522061 | 0.0112086 | 2.96E-06 | -0.00923665 | 0.0162888 | 0.570677 | 0.001325638 | 24.34181596 |
|  |  | rs15256 | C | T | 0.0865 | 0.0732376 | 0.016834 | 9.46E-06 | 0.0356585 | 0.0232637 | 0.125327 | 0.000847662 | 15.55761867 |
|  |  | rs362417 | G | C | 0.2664 | -0.054863 | 0.0120984 | 7.80E-06 | -0.00591266 | 0.0182556 | 0.746028 | 0.001176475 | 21.59960695 |
|  |  | rs55793120 | T | C | 0.0507 | 0.137396 | 0.027414 | 4.81E-07 | 0.0237911 | 0.0316748 | 0.45259 | 0.001817145 | 33.38347 |
|  |  | rs57079348 | T | G | 0.1213 | -0.0765729 | 0.0172819 | 7.22E-06 | 0.00718127 | 0.0323432 | 0.824288 | 0.001249918 | 22.94968632 |
|  |  | rs6542556 | G | A | 0.6551 | -0.050974 | 0.011406 | 7.86E-06 | 0.00580566 | 0.0154076 | 0.706318 | 0.001174163 | 21.5571041 |
|  |  | rs6793778 | T | C | 0.7376 | 0.0558706 | 0.0125258 | 9.81E-06 | 0.0355995 | 0.017056 | 0.0368689 | 0.001208318 | 22.18495013 |
|  |  | rs7120052 | A | C | 0.1759 | 0.0624796 | 0.0135523 | 1.97E-06 | 0.00858066 | 0.0191002 | 0.653255 | 0.001131755 | 20.77762921 |
|  |  | rs7155595 | C | A | 0.3091 | 0.0569929 | 0.0116986 | 1.15E-06 | -0.027644 | 0.016318 | 0.0902506 | 0.001387349 | 25.47654965 |
|  |  | rs7249614 | G | A | 0.6153 | 0.0492778 | 0.0110807 | 9.07E-06 | -0.0240013 | 0.0154685 | 0.120752 | 0.001149587 | 21.10538277 |
|  |  | rs72874194 | G | C | 0.0994 | -0.0770289 | 0.016725 | 3.47E-06 | 0.00552392 | 0.0216422 | 0.798539 | 0.001062321 | 19.50155708 |
|  |  | rs76847269 | A | G | 0.0567 | 0.163508 | 0.0356151 | 5.17E-06 | 0.0480631 | 0.0476429 | 0.313062 | 0.002859835 | 52.59405544 |
|  |  | rs77564310 | A | C | 0.1789 | -0.0713093 | 0.0140855 | 3.29E-07 | 0.00484077 | 0.0184542 | 0.793082 | 0.001493925 | 27.43658105 |
|  |  | rs79016051 | C | T | 0.0905 | -0.0887747 | 0.0189424 | 2.34E-06 | -0.0136938 | 0.0221778 | 0.536934 | 0.001297358 | 23.82184938 |
|  |  | rs882348 | A | G | 0.1064 | -0.0799946 | 0.0178618 | 5.45E-06 | -0.0101552 | 0.023254 | 0.662325 | 0.001216847 | 22.34173449 |
| ebi-a-GCST90017055 | Gut microbiota abundance (genus Ruminococcaceae UCG004 id.11362) | rs10976229 | T | G | 0.1412 | 0.095976 | 0.0214554 | 7.04E-06 | -0.0374582 | 0.0223646 | 0.0939572 | 0.002233994 | 41.05870853 |
|  |  | rs11961899 | G | A | 0.2714 | -0.0707825 | 0.0161381 | 9.18E-06 | -0.0165337 | 0.0166458 | 0.32058 | 0.001981439 | 36.40777608 |
|  |  | rs12125734 | G | T | 0.0865 | 0.133972 | 0.0257416 | 2.09E-07 | -0.00759318 | 0.025813 | 0.768635 | 0.0028365 | 52.16369313 |
|  |  | rs2248146 | T | C | 0.3221 | 0.0689452 | 0.0153649 | 8.20E-06 | -0.00893387 | 0.0157266 | 0.569986 | 0.002075843 | 38.14598717 |
|  |  | rs3800154 | A | C | 0.2207 | -0.0797959 | 0.0177639 | 6.12E-06 | -0.00371399 | 0.0170235 | 0.827298 | 0.002190273 | 40.25338386 |
|  |  | rs511258 | G | A | 0.2634 | -0.0757474 | 0.0162475 | 4.52E-06 | -0.00518289 | 0.0192226 | 0.787448 | 0.00222645 | 40.91974541 |
|  |  | rs550351 | A | C | 0.4404 | 0.078587 | 0.018003 | 9.43E-06 | -0.0170127 | 0.0151106 | 0.26022 | 0.003044083 | 55.99283347 |
|  |  | rs6769553 | A | G | 0.2843 | 0.0849743 | 0.0157451 | 7.91E-08 | -0.0257134 | 0.0169968 | 0.130321 | 0.002938415 | 54.04344783 |
|  |  | rs7123615 | C | G | 0.169 | -0.0786157 | 0.0179592 | 7.09E-06 | 0.0208438 | 0.0188705 | 0.269347 | 0.001735946 | 31.88914175 |
|  |  | rs7569771 | A | G | 0.2167 | -0.075914 | 0.0170308 | 8.12E-06 | 0.0257717 | 0.01738 | 0.13812 | 0.001956414 | 35.94704912 |
|  |  | rs872501 | G | A | 0.1272 | 0.116125 | 0.025946 | 5.81E-06 | 0.00920268 | 0.02775 | 0.740169 | 0.002994217 | 55.07285482 |
|  |  | rs9818949 | T | G | 0.7813 | -0.0859927 | 0.0188671 | 5.39E-06 | -0.0174688 | 0.0188696 | 0.354568 | 0.002527085 | 46.45908259 |
| ebi-a-GCST90017056 | Gut microbiota abundance (genus Ruminococcaceae UCG005 id.11363) | rs10873449 | C | T | 0.831 | -0.0654843 | 0.0143973 | 4.11E-06 | 0.0149568 | 0.0189691 | 0.430417 | 0.001204459 | 22.11400873 |
|  |  | rs10937802 | A | G | 0.9026 | -0.0755811 | 0.0168226 | 8.17E-06 | 0.0158636 | 0.0229464 | 0.489357 | 0.001004409 | 18.43737527 |
|  |  | rs10950694 | C | T | 0.5765 | -0.0577628 | 0.011412 | 4.30E-07 | 0.00346065 | 0.0156037 | 0.824481 | 0.001629218 | 29.92535437 |
|  |  | rs114279581 | A | G | 0.0477 | -0.146604 | 0.0316034 | 3.22E-06 | 0.0383181 | 0.0282987 | 0.175717 | 0.001952602 | 35.87687444 |
|  |  | rs12288512 | A | G | 0.1799 | 0.066635 | 0.0144356 | 3.10E-06 | -0.0263391 | 0.0174943 | 0.132173 | 0.001310185 | 24.05770099 |
|  |  | rs12458218 | T | C | 0.1769 | 0.0677322 | 0.0144525 | 2.41E-06 | 0.0151474 | 0.019427 | 0.435563 | 0.001335983 | 24.53202286 |
|  |  | rs2893871 | G | A | 0.1521 | -0.073644 | 0.015548 | 3.54E-06 | -0.0455435 | 0.0238638 | 0.05633 | 0.001398874 | 25.6884855 |
|  |  | rs34781347 | G | A | 0.0596 | 0.188685 | 0.0386484 | 6.05E-07 | -0.0199097 | 0.0291231 | 0.494204 | 0.003990834 | 73.477143 |
|  |  | rs35166120 | G | C | 0.8151 | 0.0686153 | 0.0146252 | 3.75E-06 | 0.00157852 | 0.0181235 | 0.930594 | 0.001419122 | 26.06084276 |
|  |  | rs394449 | A | T | 0.1789 | 0.0692909 | 0.0148712 | 2.60E-06 | -0.0217421 | 0.0222043 | 0.327489 | 0.001410551 | 25.90322204 |
|  |  | rs55793120 | T | C | 0.0507 | 0.121548 | 0.0279546 | 7.37E-06 | 0.0237911 | 0.0316748 | 0.45259 | 0.001422123 | 26.11602636 |
|  |  | rs60081663 | C | G | 0.0527 | 0.158082 | 0.0319586 | 9.28E-07 | 0.0350412 | 0.0425473 | 0.410177 | 0.002495129 | 45.87012621 |
|  |  | rs72776570 | C | A | 0.0825 | 0.087067 | 0.0197179 | 5.36E-06 | 0.0253141 | 0.0254211 | 0.319352 | 0.001147618 | 21.06918987 |
|  |  | rs7449320 | C | A | 0.2207 | 0.0599163 | 0.0130836 | 4.81E-06 | -0.0174236 | 0.0177253 | 0.325616 | 0.001234886 | 22.67334427 |
|  |  | rs7555878 | G | A | 0.7197 | -0.0586677 | 0.0125277 | 2.81E-06 | 0.00308288 | 0.0172938 | 0.858515 | 0.001388682 | 25.50105838 |
|  |  | rs7586445 | G | A | 0.1262 | 0.0782349 | 0.0176458 | 8.81E-06 | -0.0164996 | 0.0222549 | 0.458457 | 0.001349903 | 24.78797641 |
|  |  | rs898577 | C | T | 0.9453 | 0.123012 | 0.0286671 | 7.46E-06 | 0.0304527 | 0.0323026 | 0.345818 | 0.001564883 | 28.74180641 |
| ebi-a-GCST90017059 | Gut microbiota abundance (genus Ruminococcaceae UCG011 id.11368) | rs10274562 | C | T | 0.3519 | 0.110917 | 0.0244556 | 6.50E-06 | 0.0131879 | 0.015379 | 0.391157 | 0.00561161 | 103.4864375 |
|  |  | rs12636310 | G | A | 0.2177 | 0.132725 | 0.0282037 | 2.81E-06 | -0.0109274 | 0.0172914 | 0.527416 | 0.006000221 | 110.6962486 |
|  |  | rs12724320 | C | T | 0.2753 | -0.120881 | 0.0249229 | 1.52E-06 | -0.022845 | 0.0153883 | 0.137659 | 0.005830564 | 107.5479453 |
|  |  | rs1416041 | A | C | 0.1342 | -0.182339 | 0.0339898 | 7.04E-08 | -0.0171476 | 0.0184151 | 0.351767 | 0.007726081 | 142.7840256 |
|  |  | rs2729556 | T | C | 0.501 | 0.109097 | 0.0233699 | 3.19E-06 | 0.0381166 | 0.0150191 | 0.011153 | 0.005951054 | 109.7837555 |
|  |  | rs4490371 | T | C | 0.331 | -0.111816 | 0.0248963 | 7.75E-06 | 0.00016123 | 0.0152457 | 0.991562 | 0.005537223 | 102.1069839 |
|  |  | rs79113084 | C | T | 0.1551 | -0.152166 | 0.0317519 | 2.06E-06 | -0.00327694 | 0.0243105 | 0.892774 | 0.006068514 | 111.9638625 |
|  |  | rs9729514 | G | A | 0.8976 | -0.184934 | 0.0394616 | 2.37E-06 | 0.0111608 | 0.0257833 | 0.665108 | 0.006287041 | 116.0211959 |
| ebi-a-GCST90017062 | Gut microbiota abundance (genus Ruminococcus1 id.11373) | rs10167839 | A | G | 0.3439 | 0.0519509 | 0.0116306 | 8.09E-06 | -0.0151609 | 0.0156085 | 0.331386 | 0.001217919 | 22.36143071 |
|  |  | rs10995816 | C | G | 0.1123 | -0.0762002 | 0.0174689 | 8.38E-06 | 0.0349497 | 0.0236544 | 0.139539 | 0.001157679 | 21.25412486 |
|  |  | rs11783695 | G | T | 0.1511 | -0.0734114 | 0.0161398 | 4.73E-06 | -0.0045985 | 0.0205039 | 0.822543 | 0.001382541 | 25.38813624 |
|  |  | rs17781867 | C | T | 0.0924 | 0.0999271 | 0.0211728 | 1.96E-06 | 0.00760961 | 0.0294879 | 0.796362 | 0.0016748 | 30.7640109 |
|  |  | rs3000856 | A | T | 0.8658 | 0.0709997 | 0.0162766 | 9.28E-06 | -0.00831083 | 0.0203053 | 0.682325 | 0.001171421 | 21.50671741 |
|  |  | rs3819978 | C | T | 0.0586 | -0.11504 | 0.0260137 | 8.74E-06 | 0.0212754 | 0.0283604 | 0.453146 | 0.001460157 | 26.81551374 |
|  |  | rs4849717 | T | A | 0.0527 | 0.132764 | 0.0299816 | 8.83E-06 | -0.0283108 | 0.0286703 | 0.323417 | 0.001759903 | 32.33000438 |
|  |  | rs6105066 | T | C | 0.2495 | -0.0607052 | 0.013425 | 5.06E-06 | 0.000896802 | 0.0168635 | 0.957588 | 0.001380076 | 25.34281029 |
|  |  | rs6493760 | T | C | 0.6034 | -0.0535262 | 0.0115885 | 3.38E-06 | -0.000612594 | 0.0156514 | 0.968779 | 0.001371263 | 25.18075488 |
|  |  | rs7117576 | A | G | 0.0885 | 0.0829505 | 0.0170891 | 6.48E-07 | 0.0121032 | 0.0260445 | 0.642137 | 0.001110115 | 20.3799122 |
|  |  | rs7583465 | C | T | 0.3767 | 0.0527654 | 0.0112619 | 2.56E-06 | 0.0142856 | 0.0150219 | 0.341611 | 0.001307438 | 24.00719198 |
|  |  | rs78572139 | G | A | 0.0706 | 0.125038 | 0.0279411 | 5.23E-06 | 0.0281127 | 0.0248645 | 0.258209 | 0.002051736 | 37.70208281 |
|  |  | rs78613526 | G | A | 0.0427 | 0.167497 | 0.0367639 | 5.11E-06 | -0.00279083 | 0.0340354 | 0.934648 | 0.002293612 | 42.15695275 |
| ebi-a-GCST90017065 | Gut microbiota abundance (genus Ruminococcus gnavus group id.14376) | rs11597105 | A | G | 0.1123 | 0.114673 | 0.0250653 | 6.95E-06 | 0.0282082 | 0.0188213 | 0.133941 | 0.002621793 | 48.20481431 |
|  |  | rs11864644 | T | C | 0.0706 | -0.139804 | 0.0318253 | 5.01E-06 | 0.0557042 | 0.0235058 | 0.0177975 | 0.002564936 | 47.1567533 |
|  |  | rs12136548 | C | T | 0.2913 | 0.0901946 | 0.0196476 | 3.10E-06 | 0.0127646 | 0.0165286 | 0.439953 | 0.003358876 | 61.80265777 |
|  |  | rs12989336 | G | A | 0.3052 | -0.0846861 | 0.0187936 | 7.12E-06 | -0.0213515 | 0.0167014 | 0.201101 | 0.003041575 | 55.94657719 |
|  |  | rs13163520 | G | A | 0.1809 | -0.127377 | 0.0233883 | 5.61E-08 | 0.0238681 | 0.0192719 | 0.215533 | 0.004808255 | 88.59979648 |
|  |  | rs2909242 | A | C | 0.6143 | 0.0909965 | 0.018351 | 7.41E-07 | -0.00582898 | 0.0158992 | 0.713903 | 0.003923824 | 72.23853704 |
|  |  | rs3124783 | G | A | 0.8797 | 0.11599 | 0.0249099 | 2.67E-06 | -0.0306753 | 0.0222299 | 0.167614 | 0.00284755 | 52.3674853 |
|  |  | rs4388134 | T | C | 0.7545 | 0.0904997 | 0.0203544 | 9.12E-06 | 0.0351734 | 0.016719 | 0.0353965 | 0.003034136 | 55.80931542 |
|  |  | rs62167033 | T | C | 0.0706 | 0.185289 | 0.0396295 | 3.50E-06 | -0.0413584 | 0.0376786 | 0.272352 | 0.004505434 | 82.99457674 |
|  |  | rs78399089 | T | C | 0.0825 | 0.144445 | 0.0326618 | 6.63E-06 | 0.015115 | 0.0235286 | 0.520606 | 0.003158603 | 58.1059956 |
|  |  | rs934940 | A | C | 0.1849 | -0.105045 | 0.0229588 | 2.74E-06 | -0.0489818 | 0.0219855 | 0.0258863 | 0.003326048 | 61.19661933 |
| ebi-a-GCST90017066 | Gut microbiota abundance (genus Ruminococcus torques group id.14377) | rs10904297 | A | G | 0.0298 | -0.167804 | 0.0389812 | 2.69E-06 | -0.0224128 | 0.0516062 | 0.664067 | 0.001628216 | 29.9069289 |
|  |  | rs10967781 | C | A | 0.3181 | 0.0507994 | 0.0113322 | 8.37E-06 | 0.0114506 | 0.0164712 | 0.486937 | 0.001119519 | 20.55275109 |
|  |  | rs12434631 | A | G | 0.1402 | 0.074697 | 0.0153401 | 2.77E-06 | 0.000344194 | 0.0243114 | 0.988704 | 0.001345184 | 24.70121624 |
|  |  | rs13154778 | T | A | 0.1859 | 0.0563358 | 0.0129526 | 7.16E-06 | 0.0100491 | 0.0172532 | 0.560267 | 0.00096063 | 17.63296872 |
|  |  | rs1475330 | C | T | 0.6988 | -0.0523492 | 0.01182 | 8.13E-06 | -0.00741031 | 0.0175287 | 0.672476 | 0.001153607 | 21.1792814 |
|  |  | rs1972694 | T | A | 0.1839 | -0.0614094 | 0.0137321 | 8.93E-06 | 0.0175249 | 0.0195242 | 0.369401 | 0.001131944 | 20.78110586 |
|  |  | rs35866622 | T | C | 0.4404 | -0.0612024 | 0.0109421 | 2.21E-08 | -0.019196 | 0.0157642 | 0.22334 | 0.001846256 | 33.91926602 |
|  |  | rs4073731 | T | C | 0.1759 | 0.0651948 | 0.0142221 | 4.05E-06 | -0.0232201 | 0.0202841 | 0.252315 | 0.001232258 | 22.625028 |
|  |  | rs73130967 | A | T | 0.1123 | 0.0770375 | 0.0168264 | 3.71E-06 | -0.0134978 | 0.0267915 | 0.614397 | 0.00118326 | 21.72433491 |
|  |  | rs77034621 | T | G | 0.0527 | -0.151553 | 0.0335878 | 6.07E-06 | 0.053752 | 0.058181 | 0.355551 | 0.002293281 | 42.15084592 |
|  |  | rs773123 | T | A | 0.1123 | 0.0824005 | 0.0173862 | 1.59E-06 | 0.0346852 | 0.0227749 | 0.12777 | 0.001353741 | 24.85855928 |
| ebi-a-GCST90017069 | Gut microbiota abundance (genus Slackia id.825) | rs10409783 | G | A | 0.7167 | -0.0950828 | 0.0211239 | 7.70E-06 | 0.00136382 | 0.016579 | 0.934438 | 0.003671283 | 67.57206946 |
|  |  | rs112764253 | T | A | 0.0408 | 0.194714 | 0.0411598 | 3.40E-06 | 0.0832782 | 0.0437671 | 0.0570716 | 0.00296752 | 54.58035399 |
|  |  | rs12440440 | A | G | 0.3091 | 0.0901934 | 0.0190582 | 2.63E-06 | 0.00395356 | 0.0157476 | 0.801769 | 0.003474511 | 63.93773803 |
|  |  | rs13339230 | C | G | 0.0885 | 0.147055 | 0.0330635 | 7.42E-06 | -0.0598265 | 0.026743 | 0.0252802 | 0.003488908 | 64.20359724 |
|  |  | rs16894137 | C | T | 0.1431 | -0.122792 | 0.0263047 | 2.71E-06 | -0.00632209 | 0.0222755 | 0.776553 | 0.00369777 | 68.06138533 |
|  |  | rs35156985 | T | C | 0.0636 | -0.155709 | 0.0348085 | 8.06E-06 | -0.0215861 | 0.0377505 | 0.567451 | 0.002887859 | 53.11093064 |
|  |  | rs4492265 | G | A | 0.6899 | 0.0905757 | 0.0191658 | 2.41E-06 | -0.0209247 | 0.0163151 | 0.199655 | 0.003510276 | 64.59820481 |
|  |  | rs58767323 | G | C | 0.1899 | -0.102833 | 0.0227077 | 4.60E-06 | -0.0239207 | 0.0195653 | 0.221478 | 0.003253558 | 59.85850678 |
|  |  | rs8901 | C | T | 0.3708 | 0.0934594 | 0.0186813 | 6.07E-07 | 0.00709481 | 0.0164569 | 0.666385 | 0.004075721 | 75.046434 |
| ebi-a-GCST90017071 | Gut microbiota abundance (genus Subdoligranulum id.2070) | rs10065321 | T | C | 0.3678 | -0.0512828 | 0.0108105 | 2.10E-06 | -0.00426844 | 0.0151756 | 0.778504 | 0.001223037 | 22.45552031 |
|  |  | rs10497836 | T | C | 0.7197 | 0.0524065 | 0.0118697 | 8.38E-06 | 0.0021697 | 0.018261 | 0.905421 | 0.00110809 | 20.34268998 |
|  |  | rs1667315 | G | A | 0.4374 | 0.0485017 | 0.0107452 | 6.72E-06 | -0.010467 | 0.0153251 | 0.494609 | 0.00115777 | 21.25580206 |
|  |  | rs16962433 | A | T | 0.1083 | 0.0855999 | 0.0189213 | 7.65E-06 | -0.0117301 | 0.0307679 | 0.703022 | 0.001415219 | 25.98907122 |
|  |  | rs2114677 | C | T | 0.0557 | -0.104176 | 0.0230829 | 2.72E-06 | 0.00306701 | 0.0234938 | 0.896135 | 0.001141644 | 20.95938801 |
|  |  | rs2171249 | C | T | 0.0736 | 0.106744 | 0.0233213 | 4.51E-06 | -0.0350645 | 0.0290787 | 0.227876 | 0.001553794 | 28.5378072 |
|  |  | rs3761728 | G | T | 0.7575 | 0.0543458 | 0.0118866 | 3.87E-06 | 0.0363133 | 0.0172326 | 0.035097 | 0.001085066 | 19.91956319 |
|  |  | rs4347804 | G | A | 0.9583 | -0.166063 | 0.0357483 | 2.18E-06 | -0.0280027 | 0.042336 | 0.50833 | 0.002204009 | 40.50638732 |
|  |  | rs6555306 | C | T | 0.8777 | 0.0740777 | 0.0155463 | 2.81E-06 | 0.0509581 | 0.0214082 | 0.0172982 | 0.001178087 | 21.62924885 |
|  |  | rs75158211 | T | C | 0.1113 | -0.072343 | 0.015933 | 7.52E-06 | -0.000508023 | 0.021079 | 0.980772 | 0.001035317 | 19.00532073 |
|  |  | rs76528319 | G | T | 0.0398 | -0.143279 | 0.0310662 | 7.41E-06 | -0.00186907 | 0.0269822 | 0.944775 | 0.001569061 | 28.81866051 |
|  |  | rs76664262 | T | A | 0.0934 | 0.0834078 | 0.0185357 | 4.87E-06 | 0.0223903 | 0.0237994 | 0.346811 | 0.001178164 | 21.63066441 |
| ebi-a-GCST90017072 | Gut microbiota abundance (genus Sutterella id.2896) | rs1145877 | G | A | 0.841 | 0.073548 | 0.0162413 | 7.20E-06 | -0.028222 | 0.0217626 | 0.194695 | 0.001446655 | 26.56718549 |
|  |  | rs11591622 | T | G | 0.2157 | -0.0688382 | 0.0151375 | 6.50E-06 | -0.00657406 | 0.0203457 | 0.746607 | 0.001603324 | 29.44897676 |
|  |  | rs13173038 | A | G | 0.2207 | -0.0718076 | 0.0151627 | 2.73E-06 | -0.0232539 | 0.0173228 | 0.179471 | 0.00177369 | 32.58372898 |
|  |  | rs143438747 | T | C | 0.0547 | -0.145793 | 0.0306867 | 3.28E-06 | -0.0107296 | 0.0283037 | 0.704623 | 0.002198165 | 40.39875629 |
|  |  | rs2050185 | A | G | 0.6223 | -0.0575138 | 0.0128766 | 7.97E-06 | 0.0135702 | 0.0154481 | 0.379706 | 0.001554966 | 28.55937601 |
|  |  | rs2321387 | G | A | 0.5298 | -0.0592876 | 0.0124509 | 1.87E-06 | -0.0203423 | 0.0151093 | 0.178192 | 0.001751267 | 32.17107073 |
|  |  | rs2613606 | T | C | 0.6064 | 0.0556794 | 0.0124117 | 7.20E-06 | -0.00964593 | 0.0152168 | 0.526144 | 0.001479903 | 27.1786906 |
|  |  | rs607327 | T | C | 0.6233 | -0.0578334 | 0.0129053 | 6.63E-06 | -0.00304439 | 0.0154249 | 0.843539 | 0.001570653 | 28.84794114 |
|  |  | rs62501473 | G | A | 0.2286 | 0.0694233 | 0.0149416 | 5.52E-06 | 0.00169061 | 0.0170357 | 0.920948 | 0.001699794 | 31.22390174 |
|  |  | rs7499539 | A | G | 0.3231 | 0.0617478 | 0.0130998 | 2.36E-06 | 0.00487972 | 0.0169723 | 0.77372 | 0.001667763 | 30.63453686 |
|  |  | rs7638039 | T | C | 0.2326 | 0.0645624 | 0.0143887 | 8.66E-06 | 0.00268798 | 0.0173938 | 0.877187 | 0.001488061 | 27.32873566 |
|  |  | rs9350083 | T | G | 0.2992 | -0.0593069 | 0.0133929 | 8.23E-06 | -1.12E-05 | 0.0156104 | 0.999427 | 0.001475014 | 27.08876199 |
| ebi-a-GCST90017075 | Gut microbiota abundance (genus Tyzzerella3 id.11335) | rs10898797 | C | T | 0.1571 | 0.12238 | 0.0274683 | 8.85E-06 | -0.0249726 | 0.0239056 | 0.296192 | 0.00396646 | 73.02660957 |
|  |  | rs112102233 | A | G | 0.0666 | -0.216348 | 0.0477575 | 6.18E-06 | 0.00782778 | 0.0354056 | 0.825023 | 0.005819394 | 107.3407122 |
|  |  | rs1232220 | T | G | 0.8738 | 0.143869 | 0.031828 | 7.91E-06 | 0.011126 | 0.0247917 | 0.653591 | 0.004564948 | 84.09591105 |
|  |  | rs17706273 | T | C | 0.1521 | -0.140368 | 0.0274708 | 5.88E-07 | -0.0418332 | 0.0277664 | 0.13191 | 0.005082063 | 93.67091809 |
|  |  | rs17809157 | A | T | 0.0994 | -0.163819 | 0.0336386 | 1.54E-06 | 0.00707141 | 0.0238048 | 0.766422 | 0.004804817 | 88.53613659 |
|  |  | rs191093 | G | A | 0.0795 | 0.159008 | 0.0353306 | 6.76E-06 | -0.0169817 | 0.0243884 | 0.486239 | 0.003700487 | 68.11157416 |
|  |  | rs4904512 | T | C | 0.1988 | -0.117151 | 0.0250306 | 3.09E-06 | 0.00334659 | 0.0222981 | 0.880698 | 0.004371992 | 80.52563894 |
|  |  | rs55799124 | A | G | 0.2068 | -0.11435 | 0.0238604 | 1.34E-06 | 0.000654639 | 0.0169971 | 0.969277 | 0.004289785 | 79.00500038 |
|  |  | rs67476743 | T | G | 0.2853 | 0.132164 | 0.022208 | 3.74E-09 | 0.0121525 | 0.0168831 | 0.471646 | 0.007123311 | 131.5644492 |
|  |  | rs7019909 | T | C | 0.1014 | 0.144156 | 0.0301626 | 1.76E-06 | -0.0288465 | 0.0229659 | 0.209094 | 0.003787039 | 69.71072379 |
|  |  | rs7333521 | T | C | 0.0427 | -0.207191 | 0.0453121 | 4.88E-06 | 0.0154819 | 0.0424419 | 0.715277 | 0.00350952 | 64.58423459 |
|  |  | rs75091807 | G | T | 0.0905 | -0.184966 | 0.0383029 | 1.71E-06 | -0.0247075 | 0.0312371 | 0.428964 | 0.005632032 | 103.8651686 |
|  |  | rs7561370 | C | T | 0.8777 | -0.131341 | 0.0286292 | 1.52E-06 | -0.0171355 | 0.0210103 | 0.414743 | 0.003703422 | 68.16579716 |
| ebi-a-GCST90017078 | Gut microbiota abundance (unknown genus id.1000005472) | rs10071529 | C | G | 0.9125 | -0.125105 | 0.0278674 | 8.64E-06 | -0.00261483 | 0.024051 | 0.913424 | 0.002499311 | 45.94719676 |
|  |  | rs11779863 | G | A | 0.2068 | -0.0772768 | 0.0172171 | 6.69E-06 | 0.0198621 | 0.0205852 | 0.334609 | 0.001959122 | 35.9969027 |
|  |  | rs12566890 | T | G | 0.0954 | -0.103134 | 0.0241775 | 8.11E-06 | 0.0279033 | 0.0222866 | 0.210563 | 0.001835856 | 33.72785182 |
|  |  | rs13100746 | C | T | 0.4095 | 0.0638979 | 0.0142685 | 7.29E-06 | -0.0233567 | 0.0150067 | 0.119608 | 0.00197459 | 36.28167629 |
|  |  | rs17235252 | T | C | 0.0944 | -0.122002 | 0.0255463 | 2.16E-06 | -0.0209725 | 0.0231656 | 0.365291 | 0.002544909 | 46.78761649 |
|  |  | rs3932485 | C | T | 0.4284 | 0.0626209 | 0.0141623 | 9.93E-06 | 0.00510654 | 0.0153008 | 0.738574 | 0.001920482 | 35.28556804 |
|  |  | rs515984 | C | T | 0.826 | 0.0875451 | 0.0190685 | 6.61E-06 | -0.0197828 | 0.0233624 | 0.397118 | 0.002203043 | 40.48860099 |
|  |  | rs62188991 | G | C | 0.1113 | -0.110864 | 0.0241355 | 5.27E-06 | -0.0258113 | 0.0322986 | 0.424206 | 0.002431428 | 44.69620376 |
|  |  | rs638542 | A | G | 0.7137 | 0.070592 | 0.0157049 | 5.17E-06 | -0.00465961 | 0.0164752 | 0.77731 | 0.00203647 | 37.4209933 |
|  |  | rs74603314 | T | C | 0.0457 | 0.230767 | 0.0489701 | 2.28E-06 | -0.0621365 | 0.0382965 | 0.104694 | 0.004644923 | 85.57609412 |
|  |  | rs7706512 | A | G | 0.496 | -0.0657351 | 0.0138979 | 2.27E-06 | 0.00869044 | 0.0149476 | 0.560974 | 0.002160413 | 39.70343696 |
|  |  | rs7801843 | A | G | 0.1292 | -0.0869495 | 0.0194509 | 9.47E-06 | -0.0398097 | 0.0208393 | 0.0560919 | 0.00170116 | 31.24902773 |
|  |  | rs7853673 | A | G | 0.4861 | 0.0624389 | 0.0139833 | 6.73E-06 | 0.037232 | 0.014983 | 0.0129572 | 0.001947802 | 35.78849487 |
|  |  | rs949341 | A | G | 0.6799 | -0.0656602 | 0.0147216 | 7.73E-06 | -0.0125214 | 0.0167211 | 0.453954 | 0.001876571 | 34.477267 |
| ebi-a-GCST90017079 | Gut microbiota abundance (unknown genus id.1000005479) | rs10872669 | G | A | 0.8926 | 0.123071 | 0.0275665 | 9.49E-06 | -0.0198698 | 0.0260147 | 0.444993 | 0.00290404 | 53.40939153 |
|  |  | rs11135366 | C | G | 0.2863 | 0.0842142 | 0.0183739 | 8.78E-06 | 0.00621061 | 0.0175443 | 0.723341 | 0.002898262 | 53.30282054 |
|  |  | rs12748533 | G | T | 0.2972 | -0.0821108 | 0.0172701 | 2.59E-06 | 0.00986244 | 0.0165929 | 0.552259 | 0.002816509 | 51.79502046 |
|  |  | rs17043785 | T | C | 0.0596 | -0.176187 | 0.0347133 | 5.12E-07 | 0.018799 | 0.0263503 | 0.475584 | 0.003479658 | 64.03278593 |
|  |  | rs1850003 | A | G | 0.3072 | 0.084048 | 0.0177806 | 2.41E-06 | 0.0184336 | 0.0177341 | 0.298599 | 0.003006864 | 55.30617878 |
|  |  | rs61508842 | T | C | 0.1223 | 0.122574 | 0.0272389 | 7.83E-06 | -0.0397513 | 0.026607 | 0.135172 | 0.003225517 | 59.34092683 |
|  |  | rs6831034 | T | A | 0.1978 | -0.0956546 | 0.0205228 | 6.10E-06 | -0.00872804 | 0.0262278 | 0.739302 | 0.002903693 | 53.40298354 |
|  |  | rs738193 | T | C | 0.3549 | 0.0847328 | 0.0165856 | 3.82E-07 | -0.0193929 | 0.0156681 | 0.215815 | 0.003287503 | 60.48507417 |
|  |  | rs78609301 | A | G | 0.2167 | -0.0867065 | 0.0195709 | 7.09E-06 | 0.00222525 | 0.0163298 | 0.891608 | 0.002552233 | 46.92260893 |
|  |  | rs941000 | T | C | 0.5915 | -0.0850266 | 0.0163382 | 3.16E-07 | -0.0171337 | 0.0155648 | 0.270985 | 0.003493707 | 64.29220995 |
| ebi-a-GCST90017081 | Gut microbiota abundance (unknown genus id.1868) | rs10231386 | G | C | 0.0348 | -0.172993 | 0.0390401 | 4.62E-06 | -0.00921744 | 0.0278766 | 0.740908 | 0.002010405 | 36.94107831 |
|  |  | rs1044939 | C | T | 0.1034 | 0.109022 | 0.0242017 | 7.02E-06 | -0.0191915 | 0.024907 | 0.440987 | 0.002203827 | 40.50304675 |
|  |  | rs115372687 | A | G | 0.0865 | 0.108994 | 0.0243264 | 9.13E-06 | 0.0263164 | 0.0264953 | 0.320589 | 0.001877413 | 34.49275805 |
|  |  | rs12634544 | T | C | 0.2545 | -0.0813051 | 0.015882 | 2.60E-07 | 0.0222002 | 0.016357 | 0.174707 | 0.002508424 | 46.115162 |
|  |  | rs12930556 | C | T | 0.4493 | 0.064211 | 0.0139816 | 4.54E-06 | 0.0132348 | 0.0150237 | 0.378357 | 0.00204033 | 37.49206276 |
|  |  | rs17291611 | A | G | 0.173 | -0.0905691 | 0.0190904 | 2.30E-06 | 0.029393 | 0.0213761 | 0.169118 | 0.002347155 | 43.14338688 |
|  |  | rs3010848 | G | A | 0.336 | -0.0696728 | 0.0146631 | 1.78E-06 | -0.0161053 | 0.015657 | 0.303653 | 0.002166027 | 39.80682716 |
|  |  | rs35947731 | C | T | 0.0596 | -0.131218 | 0.0288407 | 5.43E-06 | -0.0337663 | 0.0258789 | 0.191969 | 0.001930082 | 35.46228421 |
|  |  | rs4848741 | G | A | 0.6521 | 0.0682414 | 0.0149403 | 5.53E-06 | 0.0238732 | 0.0174853 | 0.172149 | 0.002112976 | 38.82979283 |
|  |  | rs72819830 | A | G | 0.0487 | 0.226767 | 0.0467216 | 1.61E-06 | -0.0281412 | 0.0455974 | 0.537126 | 0.004764707 | 87.79349989 |
| ebi-a-GCST90017082 | Gut microbiota abundance (unknown genus id.2001) | rs10123122 | G | C | 0.0676 | 0.12759 | 0.028741 | 4.61E-06 | -0.0435752 | 0.039828 | 0.273918 | 0.002052165 | 37.70998497 |
|  |  | rs111373036 | A | G | 0.1948 | -0.0858259 | 0.0179766 | 1.70E-06 | -0.0158933 | 0.0212815 | 0.455175 | 0.002310785 | 42.47331295 |
|  |  | rs11140959 | A | G | 0.0567 | 0.190401 | 0.0432674 | 6.20E-06 | 0.00193188 | 0.0218443 | 0.929528 | 0.003877942 | 71.3905537 |
|  |  | rs12696594 | G | A | 0.494 | 0.0636249 | 0.0140502 | 6.11E-06 | 0.0162645 | 0.0150704 | 0.280484 | 0.002023772 | 37.18719825 |
|  |  | rs2136103 | T | C | 0.3042 | 0.0754533 | 0.0154038 | 6.73E-07 | -0.0184128 | 0.017014 | 0.279157 | 0.002410072 | 44.30268217 |
|  |  | rs2599390 | G | A | 0.17 | 0.094049 | 0.0198355 | 3.03E-06 | -0.0230115 | 0.0226183 | 0.30897 | 0.00249612 | 45.88838235 |
|  |  | rs55736413 | C | T | 0.0507 | 0.158132 | 0.0357614 | 8.45E-06 | -0.0853017 | 0.0356247 | 0.0166452 | 0.002407027 | 44.24656396 |
|  |  | rs7334707 | G | T | 0.9215 | -0.118994 | 0.0266494 | 6.25E-06 | -0.00803969 | 0.0345943 | 0.816228 | 0.002048543 | 37.64329846 |
|  |  | rs79929882 | A | G | 0.1372 | -0.104457 | 0.0232394 | 5.92E-06 | -0.0206067 | 0.0226732 | 0.363426 | 0.002583267 | 47.49464651 |
| ebi-a-GCST90017084 | Gut microbiota abundance (unknown genus id.2071) | rs10200320 | T | C | 0.7127 | -0.0641139 | 0.0142769 | 5.66E-06 | -0.00863863 | 0.0183553 | 0.637902 | 0.001683359 | 30.92149118 |
|  |  | rs11195523 | C | A | 0.7515 | -0.0689278 | 0.0145351 | 2.40E-06 | -0.0112959 | 0.0172941 | 0.513648 | 0.001774493 | 32.59849223 |
|  |  | rs11684166 | A | G | 0.1789 | -0.0769635 | 0.0168349 | 3.49E-06 | 0.0547412 | 0.0197874 | 0.00566657 | 0.001740227 | 31.96792188 |
|  |  | rs11809762 | G | A | 0.1262 | -0.0934634 | 0.0190198 | 1.68E-06 | 0.00688881 | 0.0189234 | 0.71583 | 0.001926569 | 35.39761619 |
|  |  | rs11904514 | A | G | 0.9235 | 0.109498 | 0.0249839 | 7.90E-06 | -0.0241379 | 0.0308863 | 0.434503 | 0.001694106 | 31.11924398 |
|  |  | rs12147596 | C | T | 0.2684 | -0.0719818 | 0.0141609 | 2.86E-07 | -0.00760796 | 0.016164 | 0.637874 | 0.002034846 | 37.39109643 |
|  |  | rs16823675 | C | T | 0.2167 | -0.0767515 | 0.0149973 | 2.33E-07 | -0.0053828 | 0.0185149 | 0.771259 | 0.001999819 | 36.74617389 |
|  |  | rs17086536 | C | A | 0.0964 | -0.100851 | 0.022432 | 3.36E-06 | -0.0120984 | 0.0285588 | 0.671834 | 0.001771918 | 32.55111391 |
|  |  | rs1809136 | C | G | 0.9155 | -0.0994594 | 0.0228638 | 8.37E-06 | 0.013877 | 0.027629 | 0.615484 | 0.001530512 | 28.10955003 |
|  |  | rs2898979 | G | C | 0.1322 | 0.0901515 | 0.0202199 | 7.67E-06 | -0.0317181 | 0.0212525 | 0.135584 | 0.001864777 | 34.26017666 |
|  |  | rs2939766 | A | G | 0.4085 | -0.0591611 | 0.013042 | 7.01E-06 | -0.00692247 | 0.0150504 | 0.64555 | 0.001691412 | 31.06965617 |
|  |  | rs34985298 | G | A | 0.2922 | -0.0623526 | 0.013772 | 8.34E-06 | 0.0137196 | 0.0158385 | 0.386371 | 0.001608162 | 29.53798375 |
|  |  | rs35740166 | C | T | 0.1074 | -0.112246 | 0.0226824 | 8.40E-07 | 0.0132854 | 0.0254734 | 0.601991 | 0.002415644 | 44.40534461 |
|  |  | rs4644504 | T | C | 0.0785 | -0.0969321 | 0.0216146 | 5.82E-06 | -0.0414396 | 0.0298075 | 0.164456 | 0.001359347 | 24.96163119 |
|  |  | rs6007642 | C | T | 0.169 | -0.0791412 | 0.0177958 | 9.96E-06 | -0.00208326 | 0.0179973 | 0.907847 | 0.001759231 | 32.31764098 |
|  |  | rs72700702 | T | C | 0.1372 | -0.091726 | 0.0189005 | 1.59E-06 | -0.0183533 | 0.0238307 | 0.441209 | 0.001991953 | 36.6013479 |
|  |  | rs76532867 | T | C | 0.1054 | 0.112353 | 0.0242364 | 2.56E-06 | 0.0191671 | 0.038181 | 0.615663 | 0.002380504 | 43.75784115 |
| ebi-a-GCST90017085 | Gut microbiota abundance (unknown genus id.2755) | rs1035406 | G | A | 0.0994 | -0.111241 | 0.025156 | 9.18E-06 | 0.00405851 | 0.023366 | 0.862107 | 0.002215532 | 40.71864529 |
|  |  | rs11630875 | T | C | 0.1819 | 0.0925877 | 0.0204021 | 6.93E-06 | 0.0272714 | 0.0224026 | 0.223477 | 0.002551383 | 46.90694135 |
|  |  | rs12977163 | G | C | 0.3678 | 0.0744853 | 0.0163909 | 5.36E-06 | -0.0189016 | 0.0153839 | 0.219199 | 0.002580105 | 47.43635345 |
|  |  | rs13336560 | C | T | 0.4563 | -0.0722586 | 0.0158485 | 5.30E-06 | 0.00819912 | 0.0152205 | 0.590101 | 0.00259071 | 47.63184926 |
|  |  | rs1549633 | A | C | 0.1561 | 0.101844 | 0.0219252 | 3.40E-06 | 0.0124297 | 0.0234265 | 0.595709 | 0.002732718 | 50.24989739 |
|  |  | rs2074723 | T | C | 0.0676 | 0.166302 | 0.0375751 | 8.75E-06 | 0.0143533 | 0.0332756 | 0.666216 | 0.003486373 | 64.15679016 |
|  |  | rs3730086 | A | G | 0.2386 | 0.0806746 | 0.0180043 | 8.65E-06 | -0.0182149 | 0.0171933 | 0.28941 | 0.002364759 | 43.46774749 |
|  |  | rs3754624 | C | T | 0.162 | 0.0964478 | 0.0200589 | 2.16E-06 | -0.00317178 | 0.0195514 | 0.871126 | 0.002525653 | 46.43269736 |
|  |  | rs56250014 | A | T | 0.0895 | 0.119153 | 0.025758 | 3.26E-06 | 0.00146724 | 0.025621 | 0.954332 | 0.002313891 | 42.53054881 |
|  |  | rs61933850 | G | A | 0.0905 | 0.165184 | 0.0361469 | 7.43E-06 | 0.0216852 | 0.0219325 | 0.322797 | 0.004491767 | 82.7416825 |
|  |  | rs7098347 | G | A | 0.4225 | 0.0705089 | 0.0158542 | 7.36E-06 | -0.02602 | 0.0150985 | 0.0848262 | 0.002426032 | 44.59677327 |
|  |  | rs74354280 | C | T | 0.1531 | -0.0903316 | 0.0205472 | 8.70E-06 | 0.025076 | 0.0166823 | 0.132801 | 0.002116005 | 38.88558493 |
|  |  | rs76784716 | A | G | 0.0716 | 0.139756 | 0.0286444 | 8.43E-07 | 0.00635328 | 0.0236345 | 0.788074 | 0.002596684 | 47.74195889 |
|  |  | rs9813022 | A | G | 0.3022 | -0.0822797 | 0.016401 | 5.10E-07 | 0.0149184 | 0.0154449 | 0.334088 | 0.002855228 | 52.50909965 |
| ebi-a-GCST90017088 | Gut microbiota abundance (genus Veillonella id.2198) | rs12679709 | C | G | 0.3211 | -0.0793499 | 0.0164818 | 1.78E-06 | -0.0222145 | 0.0156194 | 0.154958 | 0.002745168 | 50.47945954 |
|  |  | rs1882878 | A | G | 0.3062 | -0.0768966 | 0.0163908 | 2.98E-06 | -0.00291409 | 0.0163858 | 0.858846 | 0.002512371 | 46.18789855 |
|  |  | rs2013594 | C | T | 0.6421 | 0.0720698 | 0.0155153 | 3.42E-06 | -0.00595496 | 0.0152808 | 0.696756 | 0.002387267 | 43.8824617 |
|  |  | rs62376424 | C | T | 0.334 | -0.0762216 | 0.0163499 | 3.65E-06 | 0.00529761 | 0.0162358 | 0.744202 | 0.00258468 | 47.52069105 |
|  |  | rs6656807 | G | A | 0.5755 | -0.0703062 | 0.0153954 | 5.50E-06 | -0.00493816 | 0.0155797 | 0.751273 | 0.002415129 | 44.39585076 |
|  |  | rs742016 | A | G | 0.4235 | -0.0688571 | 0.0149769 | 4.66E-06 | -0.0319901 | 0.0158488 | 0.0435442 | 0.002315156 | 42.55384145 |
|  |  | rs7645873 | T | A | 0.7167 | -0.0761385 | 0.0164228 | 3.12E-06 | 0.0238 | 0.0177428 | 0.179795 | 0.002354088 | 43.27112123 |
| ebi-a-GCST90017089 | Gut microbiota abundance (genus Victivallis id.2256) | rs11899949 | G | A | 0.2773 | 0.130568 | 0.0276239 | 2.77E-06 | 0.0327863 | 0.0162519 | 0.0436556 | 0.006833 | 126.1656447 |
|  |  | rs12512543 | A | C | 0.1213 | -0.178036 | 0.0374344 | 2.54E-06 | 0.0276592 | 0.0271843 | 0.308931 | 0.006756894 | 124.7508428 |
|  |  | rs173120 | C | T | 0.7545 | -0.13381 | 0.0290127 | 7.65E-06 | 0.0318041 | 0.0191085 | 0.0960329 | 0.00663312 | 122.4503893 |
|  |  | rs1882775 | A | G | 0.2237 | -0.13827 | 0.031273 | 8.73E-06 | 0.0427296 | 0.0193334 | 0.0270951 | 0.006640205 | 122.5820504 |
|  |  | rs2546432 | C | T | 0.5338 | 0.110803 | 0.0249655 | 9.93E-06 | 0.00494435 | 0.0150128 | 0.741896 | 0.0061106 | 112.7451275 |
|  |  | rs342302 | A | G | 0.159 | -0.152754 | 0.0351572 | 8.16E-06 | -0.0311762 | 0.0217195 | 0.151173 | 0.006240341 | 115.1539671 |
|  |  | rs4764863 | G | A | 0.5 | 0.121561 | 0.0246058 | 8.22E-07 | 0.0222851 | 0.0150568 | 0.138855 | 0.007388538 | 136.4995486 |
|  |  | rs4895919 | C | T | 0.5179 | 0.116918 | 0.0247515 | 2.75E-06 | -0.00589124 | 0.0149866 | 0.694245 | 0.006826149 | 126.0382851 |
|  |  | rs56349194 | A | G | 0.16 | -0.158501 | 0.0315231 | 6.26E-07 | -0.00845713 | 0.0221814 | 0.703003 | 0.006752946 | 124.6774641 |
|  |  | rs592514 | T | A | 0.1282 | -0.181449 | 0.0391678 | 2.60E-06 | 0.0160301 | 0.0226501 | 0.479114 | 0.007359428 | 135.9577566 |
|  |  | rs6445926 | G | C | 0.3966 | 0.117031 | 0.0249764 | 2.96E-06 | -0.0256679 | 0.01517 | 0.0906421 | 0.006555259 | 121.0035457 |
|  |  | rs911666 | T | C | 0.3052 | -0.118576 | 0.0263146 | 7.65E-06 | 0.00271737 | 0.0162349 | 0.867072 | 0.005963043 | 110.0062509 |
| ebi-a-GCST90017091 | Gut microbiota abundance (order Bacillales id.1674) | rs10233278 | T | C | 0.4125 | -0.116319 | 0.0248527 | 3.51E-06 | 0.00583005 | 0.0151436 | 0.70025 | 0.006557875 | 121.0521581 |
|  |  | rs10410917 | C | T | 0.4036 | -0.11471 | 0.0250025 | 5.57E-06 | 0.0139439 | 0.0153307 | 0.363066 | 0.006334631 | 116.9050204 |
|  |  | rs11034576 | A | G | 0.0795 | 0.205765 | 0.0453311 | 8.86E-06 | -0.0349118 | 0.0226439 | 0.123129 | 0.006196749 | 114.3445532 |
|  |  | rs11207728 | A | G | 0.7972 | 0.14455 | 0.0316912 | 5.73E-06 | -0.00157157 | 0.0201271 | 0.937763 | 0.006756183 | 124.737641 |
|  |  | rs11844714 | A | G | 0.163 | -0.143245 | 0.0320272 | 5.06E-06 | 0.0121981 | 0.0185343 | 0.510452 | 0.005598891 | 103.2505491 |
|  |  | rs12522021 | T | A | 0.0924 | 0.174339 | 0.0400515 | 7.60E-06 | -0.00143309 | 0.0219044 | 0.947836 | 0.005097832 | 93.96305892 |
|  |  | rs1287018 | G | A | 0.16 | 0.141125 | 0.0319309 | 9.87E-06 | 0.00605179 | 0.019215 | 0.752799 | 0.005353492 | 98.70073357 |
|  |  | rs4617108 | G | A | 0.9394 | -0.248861 | 0.0525929 | 1.98E-06 | -0.0182701 | 0.026749 | 0.494593 | 0.007051262 | 130.2242905 |
|  |  | rs74420793 | A | G | 0.162 | -0.164413 | 0.0353763 | 3.07E-06 | -0.0059221 | 0.0244382 | 0.808525 | 0.007339413 | 135.585275 |
| ebi-a-GCST90017094 | Gut microbiota abundance (order Burkholderiales id.2874) | rs1511453 | A | G | 0.0636 | 0.0910999 | 0.0199479 | 8.00E-06 | 0.0494048 | 0.0320107 | 0.122738 | 0.000988517 | 18.14536904 |
|  |  | rs1928341 | G | A | 0.5785 | -0.0507782 | 0.0110587 | 4.52E-06 | -0.0258432 | 0.0152287 | 0.0896954 | 0.001257435 | 23.08787437 |
|  |  | rs2321387 | G | A | 0.5298 | -0.0508514 | 0.0109588 | 3.26E-06 | -0.0203423 | 0.0151093 | 0.178192 | 0.00128834 | 23.65605115 |
|  |  | rs2367850 | G | C | 0.2207 | 0.06333 | 0.0130595 | 1.24E-06 | -0.0275745 | 0.0183191 | 0.132266 | 0.001379609 | 25.33421896 |
|  |  | rs2613606 | T | C | 0.6064 | 0.0499561 | 0.0109264 | 4.13E-06 | -0.00964593 | 0.0152168 | 0.526144 | 0.001191301 | 21.87212517 |
|  |  | rs4033856 | T | C | 0.8708 | -0.0833445 | 0.0167489 | 5.67E-07 | 0.0196837 | 0.0265754 | 0.458893 | 0.001563021 | 28.70755011 |
|  |  | rs6087811 | T | G | 0.0815 | -0.101587 | 0.0198817 | 2.88E-07 | 0.0181971 | 0.0248295 | 0.463631 | 0.001545052 | 28.37700327 |
|  |  | rs62191117 | A | G | 0.2058 | 0.0679995 | 0.0132471 | 2.79E-07 | -0.00260432 | 0.0185192 | 0.888164 | 0.00151153 | 27.76039246 |
|  |  | rs62395635 | T | C | 0.0706 | 0.109911 | 0.0236375 | 2.90E-06 | -0.0251912 | 0.0313678 | 0.421922 | 0.00158533 | 29.11794344 |
|  |  | rs72747231 | C | G | 0.0417 | -0.133729 | 0.0290738 | 3.76E-06 | -0.0029339 | 0.0353796 | 0.93391 | 0.001429285 | 26.2477376 |
|  |  | rs75242906 | C | T | 0.0537 | -0.120974 | 0.0281273 | 9.75E-06 | 0.0657843 | 0.0272101 | 0.015621 | 0.001487364 | 27.31590579 |
|  |  | rs7638039 | T | C | 0.2326 | 0.0580987 | 0.012673 | 4.84E-06 | 0.00268798 | 0.0173938 | 0.877187 | 0.00120502 | 22.12432086 |
| ebi-a-GCST90017095 | Gut microbiota abundance (order Clostridiales id.1863) | rs10209007 | G | C | 0.0795 | 0.0795424 | 0.0182427 | 9.60E-06 | -0.0101484 | 0.0336244 | 0.762792 | 0.000926016 | 16.99701339 |
|  |  | rs10774377 | G | A | 0.666 | -0.0523003 | 0.0113859 | 3.81E-06 | -0.013015 | 0.0151568 | 0.390511 | 0.001216912 | 22.34291534 |
|  |  | rs112334273 | G | A | 0.2455 | 0.0639383 | 0.0127404 | 4.07E-07 | 0.0169935 | 0.0167255 | 0.30962 | 0.001514478 | 27.81461818 |
|  |  | rs13105690 | C | T | 0.7326 | 0.0527441 | 0.011818 | 9.37E-06 | -0.020048 | 0.0166909 | 0.229699 | 0.001089949 | 20.00928963 |
|  |  | rs13179700 | C | T | 0.6451 | -0.0510953 | 0.0109569 | 3.52E-06 | 0.0109695 | 0.0157711 | 0.486712 | 0.001195432 | 21.94807281 |
|  |  | rs1842454 | G | A | 0.2068 | -0.0543676 | 0.012734 | 9.92E-06 | -0.00977277 | 0.0191645 | 0.610093 | 0.000969714 | 17.79987177 |
|  |  | rs2273429 | A | G | 0.1074 | -0.0726191 | 0.0153105 | 4.17E-06 | 0.0387422 | 0.0243074 | 0.11097 | 0.001011097 | 18.56026567 |
|  |  | rs290772 | G | A | 0.0616 | 0.084254 | 0.0196297 | 1.00E-05 | 0.0337569 | 0.0332427 | 0.309883 | 0.000820691 | 15.06219619 |
|  |  | rs6442336 | T | C | 0.7565 | 0.0547729 | 0.0124169 | 9.63E-06 | 0.0137183 | 0.0172187 | 0.425618 | 0.001105273 | 20.29091411 |
|  |  | rs6814436 | C | T | 0.8678 | -0.0741858 | 0.0150814 | 9.06E-07 | -0.0237378 | 0.021395 | 0.267214 | 0.001262765 | 23.18586978 |
|  |  | rs6815608 | C | T | 0.9155 | -0.104089 | 0.0210883 | 3.72E-07 | -0.00785943 | 0.0208815 | 0.706632 | 0.001676312 | 30.79181705 |
|  |  | rs6934062 | G | C | 0.2843 | 0.0522397 | 0.0116116 | 7.09E-06 | 0.0151594 | 0.0160283 | 0.344256 | 0.001110553 | 20.3879596 |
|  |  | rs7269302 | A | T | 0.0706 | -0.0965346 | 0.0212006 | 8.84E-06 | -0.0394344 | 0.031611 | 0.212218 | 0.001222935 | 22.45364102 |
|  |  | rs72738886 | T | C | 0.0775 | 0.0865333 | 0.0190392 | 8.42E-06 | -0.000193078 | 0.0282506 | 0.994547 | 0.001070692 | 19.65539692 |
|  |  | rs76860606 | C | G | 0.0716 | 0.094122 | 0.0225117 | 8.58E-06 | 0.0257469 | 0.0266186 | 0.333417 | 0.00117777 | 21.62341146 |
|  |  | rs992074 | T | C | 0.9652 | -0.255441 | 0.0508788 | 8.95E-07 | 0.0370538 | 0.0495712 | 0.454769 | 0.004383366 | 80.73606694 |
| ebi-a-GCST90017098 | Gut microbiota abundance (order Enterobacteriales id.3468) | rs11026530 | T | C | 0.1342 | 0.0822408 | 0.0186379 | 9.43E-06 | 0.00626877 | 0.0209924 | 0.76523 | 0.001571718 | 28.86754421 |
|  |  | rs111229068 | A | T | 0.1054 | 0.110561 | 0.0241719 | 3.65E-06 | -0.0217281 | 0.0278432 | 0.435172 | 0.002305172 | 42.36992199 |
|  |  | rs2374342 | C | A | 0.3569 | 0.0582927 | 0.0126195 | 4.52E-06 | 0.00994865 | 0.0152209 | 0.513358 | 0.001559852 | 28.6492547 |
|  |  | rs35673018 | G | A | 0.0944 | 0.0899642 | 0.0202934 | 7.63E-06 | 0.0410929 | 0.0262109 | 0.116932 | 0.001383814 | 25.41155352 |
|  |  | rs4792380 | A | T | 0.0517 | 0.115598 | 0.0257683 | 9.49E-06 | -0.0431183 | 0.0438956 | 0.325957 | 0.001310289 | 24.05959555 |
|  |  | rs504442 | T | G | 0.1392 | 0.084159 | 0.0189479 | 5.17E-06 | 0.0129514 | 0.0245389 | 0.597646 | 0.001697355 | 31.1790134 |
|  |  | rs61973590 | C | G | 0.2942 | -0.0605392 | 0.0133696 | 8.54E-06 | 0.0106318 | 0.0157262 | 0.499005 | 0.001522046 | 27.95381981 |
|  |  | rs62210023 | A | G | 0.3519 | 0.0606754 | 0.0130125 | 3.13E-06 | 0.0130869 | 0.0157056 | 0.404697 | 0.001679255 | 30.84597147 |
|  |  | rs78143293 | A | G | 0.1441 | -0.0848521 | 0.0170415 | 1.20E-06 | 0.0225516 | 0.0229915 | 0.326659 | 0.001775997 | 32.62617436 |
|  |  | rs79757635 | C | A | 0.161 | 0.0758597 | 0.0171286 | 9.32E-06 | -0.0145046 | 0.0220736 | 0.511114 | 0.001554677 | 28.55405262 |
|  |  | rs80319214 | C | G | 0.1153 | 0.0990404 | 0.0215877 | 6.95E-06 | 0.00470847 | 0.0287738 | 0.870017 | 0.002001152 | 36.77071122 |
| ebi-a-GCST90017100 | Gut microbiota abundance (order Gastranaerophilales id.1591) | rs11150282 | T | C | 0.3211 | 0.098235 | 0.0197127 | 7.36E-07 | -0.00447128 | 0.0156208 | 0.774695 | 0.00420735 | 77.48036549 |
|  |  | rs113884518 | T | C | 0.0487 | -0.20591 | 0.0455376 | 7.74E-06 | -0.00587112 | 0.0483315 | 0.903314 | 0.003928541 | 72.32572624 |
|  |  | rs1221147 | A | T | 0.8439 | -0.126305 | 0.0279161 | 4.86E-06 | 0.0274885 | 0.0209781 | 0.190079 | 0.004203054 | 77.40092491 |
|  |  | rs16851659 | G | C | 0.3887 | -0.0895168 | 0.0186555 | 1.27E-06 | 0.0199539 | 0.0157364 | 0.204793 | 0.003808097 | 70.09983421 |
|  |  | rs28678345 | T | C | 0.0467 | 0.213083 | 0.0471377 | 8.06E-06 | -0.0329631 | 0.0349631 | 0.345786 | 0.004042724 | 74.43639538 |
|  |  | rs367480 | A | G | 0.5726 | 0.0841649 | 0.0185948 | 7.52E-06 | -0.0206672 | 0.0156938 | 0.187872 | 0.003467192 | 63.80258105 |
|  |  | rs4129395 | G | A | 0.4264 | 0.0904264 | 0.0185253 | 1.22E-06 | -0.009335 | 0.0149468 | 0.532266 | 0.003999879 | 73.64434334 |
|  |  | rs73074665 | A | T | 0.0855 | 0.164631 | 0.0356037 | 3.62E-06 | 0.0687311 | 0.0303448 | 0.023512 | 0.004238411 | 78.05480648 |
|  |  | rs789069 | A | C | 0.172 | -0.10413 | 0.0234459 | 6.50E-06 | 0.00231585 | 0.0214539 | 0.914039 | 0.00308845 | 56.81144774 |
|  |  | rs79790072 | T | C | 0.0567 | 0.226103 | 0.0488017 | 3.54E-06 | -0.0351942 | 0.043692 | 0.420528 | 0.005468592 | 100.8344663 |
|  |  | rs8028558 | A | G | 0.4046 | 0.08346 | 0.0188504 | 9.78E-06 | -0.00739528 | 0.015589 | 0.635221 | 0.003355996 | 61.74949005 |
|  |  | rs9864379 | T | C | 0.1133 | -0.160521 | 0.0292752 | 4.66E-08 | -0.0331073 | 0.0211651 | 0.117761 | 0.005177264 | 95.43476171 |
| ebi-a-GCST90017103 | Gut microbiota abundance (order Mollicutes RF9 id.11579) | rs10071529 | C | G | 0.9125 | -0.125105 | 0.0278674 | 8.64E-06 | -0.00261483 | 0.024051 | 0.913424 | 0.002499311 | 45.94719676 |
|  |  | rs11779863 | G | A | 0.2068 | -0.0772768 | 0.0172171 | 6.69E-06 | 0.0198621 | 0.0205852 | 0.334609 | 0.001959122 | 35.9969027 |
|  |  | rs12566890 | T | G | 0.0954 | -0.103134 | 0.0241775 | 8.11E-06 | 0.0279033 | 0.0222866 | 0.210563 | 0.001835856 | 33.72785182 |
|  |  | rs13100746 | C | T | 0.4095 | 0.0638979 | 0.0142685 | 7.29E-06 | -0.0233567 | 0.0150067 | 0.119608 | 0.00197459 | 36.28167629 |
|  |  | rs17235252 | T | C | 0.0944 | -0.122002 | 0.0255463 | 2.16E-06 | -0.0209725 | 0.0231656 | 0.365291 | 0.002544909 | 46.78761649 |
|  |  | rs3932485 | C | T | 0.4284 | 0.0626209 | 0.0141623 | 9.93E-06 | 0.00510654 | 0.0153008 | 0.738574 | 0.001920482 | 35.28556804 |
|  |  | rs515984 | C | T | 0.826 | 0.0875451 | 0.0190685 | 6.61E-06 | -0.0197828 | 0.0233624 | 0.397118 | 0.002203043 | 40.48860099 |
|  |  | rs62188991 | G | C | 0.1113 | -0.110864 | 0.0241355 | 5.27E-06 | -0.0258113 | 0.0322986 | 0.424206 | 0.002431428 | 44.69620376 |
|  |  | rs638542 | A | G | 0.7137 | 0.070592 | 0.0157049 | 5.17E-06 | -0.00465961 | 0.0164752 | 0.77731 | 0.00203647 | 37.4209933 |
|  |  | rs74603314 | T | C | 0.0457 | 0.230767 | 0.0489701 | 2.28E-06 | -0.0621365 | 0.0382965 | 0.104694 | 0.004644923 | 85.57609412 |
|  |  | rs7706512 | A | G | 0.496 | -0.0657351 | 0.0138979 | 2.27E-06 | 0.00869044 | 0.0149476 | 0.560974 | 0.002160413 | 39.70343696 |
|  |  | rs7801843 | A | G | 0.1292 | -0.0869495 | 0.0194509 | 9.47E-06 | -0.0398097 | 0.0208393 | 0.0560919 | 0.00170116 | 31.24902773 |
|  |  | rs7853673 | A | G | 0.4861 | 0.0624389 | 0.0139833 | 6.73E-06 | 0.037232 | 0.014983 | 0.0129572 | 0.001947802 | 35.78849487 |
|  |  | rs949341 | A | G | 0.6799 | -0.0656602 | 0.0147216 | 7.73E-06 | -0.0125214 | 0.0167211 | 0.453954 | 0.001876571 | 34.477267 |
| ebi-a-GCST90017104 | Gut microbiota abundance (order NB1n id.3953) | rs11251024 | G | A | 0.33 | 0.104201 | 0.0206737 | 6.63E-07 | -0.0137927 | 0.0169654 | 0.416224 | 0.004801341 | 88.47176632 |
|  |  | rs11606187 | A | G | 0.1074 | -0.154539 | 0.0326288 | 3.31E-06 | -0.00218922 | 0.0213321 | 0.91826 | 0.004578965 | 84.35532626 |
|  |  | rs13219468 | G | C | 0.2256 | 0.115344 | 0.0236958 | 1.41E-06 | -0.00401383 | 0.0165601 | 0.808486 | 0.004648624 | 85.64460269 |
|  |  | rs13385922 | T | C | 0.4056 | 0.0929494 | 0.0201348 | 3.97E-06 | 0.00712556 | 0.0157359 | 0.650676 | 0.004165814 | 76.71227485 |
|  |  | rs166849 | A | G | 0.6103 | -0.0911053 | 0.0202239 | 7.74E-06 | -0.00384592 | 0.0151759 | 0.799941 | 0.003948126 | 72.68772363 |
|  |  | rs2172426 | T | C | 0.5527 | 0.10212 | 0.0198947 | 3.17E-07 | -0.00588166 | 0.0152794 | 0.700282 | 0.005156321 | 95.04671126 |
|  |  | rs267959 | G | A | 0.6571 | -0.0988612 | 0.0209676 | 2.62E-06 | -0.000304482 | 0.0167005 | 0.985454 | 0.004404339 | 81.12405979 |
|  |  | rs2930903 | C | G | 0.3887 | 0.0908335 | 0.0209434 | 6.84E-06 | 0.0450507 | 0.0155209 | 0.00370109 | 0.003920948 | 72.18537069 |
|  |  | rs4383094 | C | T | 0.9036 | -0.149188 | 0.0320662 | 4.28E-06 | -0.00438522 | 0.0215402 | 0.838679 | 0.003877493 | 71.38225298 |
|  |  | rs55921101 | A | T | 0.2266 | -0.109245 | 0.0236201 | 4.32E-06 | 0.0333283 | 0.0175655 | 0.0577777 | 0.00418309 | 77.0317348 |
|  |  | rs60775321 | T | C | 0.3012 | -0.0962425 | 0.0213842 | 7.10E-06 | -0.0103233 | 0.0165335 | 0.532374 | 0.003899165 | 71.78278689 |
|  |  | rs72671304 | T | C | 0.0696 | 0.172322 | 0.0370028 | 3.80E-06 | -0.00854359 | 0.0284309 | 0.763792 | 0.003845833 | 70.79715442 |
|  |  | rs7911787 | G | T | 0.0656 | -0.223071 | 0.0470232 | 3.39E-06 | -0.0915447 | 0.0422916 | 0.030418 | 0.006100324 | 112.5543573 |
|  |  | rs8126061 | T | C | 0.0954 | -0.159135 | 0.0351904 | 7.36E-06 | 0.00129576 | 0.0225698 | 0.954218 | 0.004370855 | 80.50460765 |
|  |  | rs9542068 | T | C | 0.3211 | 0.0990709 | 0.0218149 | 6.52E-06 | -0.0183789 | 0.0158634 | 0.246631 | 0.004279257 | 78.81025636 |
| ebi-a-GCST90017106 | Gut microbiota abundance (order Rhodospirillales id.2667) | rs1035406 | G | A | 0.0994 | -0.114725 | 0.0247994 | 4.07E-06 | 0.00405851 | 0.023366 | 0.862107 | 0.002356484 | 43.31527135 |
|  |  | rs11591293 | G | T | 0.4195 | 0.0722755 | 0.0158186 | 4.69E-06 | -0.0190878 | 0.0151162 | 0.206682 | 0.002544172 | 46.7740193 |
|  |  | rs11630875 | T | C | 0.1819 | 0.0945198 | 0.0202139 | 3.70E-06 | 0.0272714 | 0.0224026 | 0.223477 | 0.002658978 | 48.89032915 |
|  |  | rs13336560 | C | T | 0.4563 | -0.0695313 | 0.0157065 | 9.75E-06 | 0.00819912 | 0.0152205 | 0.590101 | 0.002398836 | 44.09562647 |
|  |  | rs1549633 | A | C | 0.1561 | 0.0996735 | 0.0216667 | 3.88E-06 | 0.0124297 | 0.0234265 | 0.595709 | 0.00261748 | 48.12530762 |
|  |  | rs3730086 | A | G | 0.2386 | 0.0800354 | 0.0178737 | 7.98E-06 | -0.0182149 | 0.0171933 | 0.28941 | 0.002327435 | 42.78006949 |
|  |  | rs3754624 | C | T | 0.162 | 0.094128 | 0.0198389 | 2.68E-06 | -0.00317178 | 0.0195514 | 0.871126 | 0.002405618 | 44.22060343 |
|  |  | rs4278423 | T | C | 0.1064 | 0.105215 | 0.0233778 | 3.98E-06 | -0.0267214 | 0.0309006 | 0.387174 | 0.002105087 | 38.68452446 |
|  |  | rs4822789 | G | C | 0.3926 | 0.0727755 | 0.0159522 | 7.33E-06 | -0.00184669 | 0.0153518 | 0.904252 | 0.002525954 | 46.43824929 |
|  |  | rs61933850 | G | A | 0.0905 | 0.164663 | 0.0360285 | 7.00E-06 | 0.0216852 | 0.0219325 | 0.322797 | 0.004463477 | 82.21822496 |
|  |  | rs7001029 | C | T | 0.0934 | 0.120726 | 0.0260989 | 2.83E-06 | 0.00361712 | 0.0258535 | 0.888732 | 0.002468279 | 45.37529514 |
|  |  | rs76784716 | A | G | 0.0716 | 0.136039 | 0.0284399 | 1.31E-06 | 0.00635328 | 0.0236345 | 0.788074 | 0.002460396 | 45.23002553 |
|  |  | rs77304857 | C | A | 0.1123 | -0.0997433 | 0.0222165 | 6.02E-06 | 0.0117784 | 0.0189528 | 0.534299 | 0.001983551 | 36.44665758 |
|  |  | rs9813022 | A | G | 0.3022 | -0.0830794 | 0.0162642 | 3.07E-07 | 0.0149184 | 0.0154449 | 0.334088 | 0.002910999 | 53.53775632 |
| ebi-a-GCST90017107 | Gut microbiota abundance (order Selenomonadales id.2165) | rs1135612 | G | A | 0.2445 | 0.0529785 | 0.0119177 | 9.26E-06 | 0.0377751 | 0.0184197 | 0.0402865 | 0.001036914 | 19.03466208 |
|  |  | rs13086907 | G | A | 0.1998 | 0.0625312 | 0.0131735 | 1.95E-06 | -0.0147292 | 0.0179994 | 0.413177 | 0.00125031 | 22.95687996 |
|  |  | rs1447205 | C | G | 0.6382 | 0.0502053 | 0.0108382 | 2.93E-06 | 0.00156645 | 0.0155953 | 0.919992 | 0.001164004 | 21.37038147 |
|  |  | rs1643968 | T | C | 0.2803 | -0.0565284 | 0.0112298 | 4.15E-07 | 0.019778 | 0.0155953 | 0.204725 | 0.001289253 | 23.67283262 |
|  |  | rs1649999 | A | G | 0.0974 | 0.0748989 | 0.0166458 | 7.58E-06 | -0.0244992 | 0.0254109 | 0.334985 | 0.000986359 | 18.10571629 |
|  |  | rs2834062 | A | G | 0.3648 | 0.0489057 | 0.0108842 | 8.44E-06 | -0.0184384 | 0.016391 | 0.260626 | 0.001108445 | 20.34922626 |
|  |  | rs4463806 | C | T | 0.7962 | 0.0543966 | 0.0129365 | 7.81E-06 | -0.0241862 | 0.0189899 | 0.202793 | 0.000960284 | 17.62662132 |
|  |  | rs4722181 | T | G | 0.4761 | 0.0501306 | 0.0105798 | 2.00E-06 | 0.0176441 | 0.0149705 | 0.238561 | 0.001253668 | 23.01861301 |
|  |  | rs60274479 | T | C | 0.2227 | -0.0659623 | 0.0134137 | 1.16E-06 | -0.00698118 | 0.0188712 | 0.71143 | 0.001506366 | 27.66541117 |
|  |  | rs61249479 | A | C | 0.0875 | 0.0777075 | 0.0168628 | 2.95E-06 | 0.00838398 | 0.0206938 | 0.685371 | 0.000964266 | 17.69977484 |
|  |  | rs71405394 | G | A | 0.0517 | -0.114178 | 0.02405 | 2.17E-06 | 0.0558044 | 0.0295972 | 0.0593677 | 0.001278295 | 23.47138041 |
|  |  | rs73232831 | G | A | 0.0636 | -0.151829 | 0.0314932 | 1.87E-06 | -0.0497287 | 0.0406899 | 0.221655 | 0.002745731 | 50.48984575 |
|  |  | rs9423647 | G | A | 0.5149 | 0.047817 | 0.0105282 | 6.06E-06 | 0.0117977 | 0.0150397 | 0.432782 | 0.001142218 | 20.96993689 |
| ebi-a-GCST90017110 | Gut microbiota abundance (phylum Actinobacteria id.400) | rs10841473 | G | C | 0.2704 | -0.0596572 | 0.0117537 | 4.47E-07 | -0.00428621 | 0.0177846 | 0.80955 | 0.001404259 | 25.78751533 |
|  |  | rs11766971 | C | T | 0.495 | -0.0476376 | 0.010646 | 9.40E-06 | -0.0109025 | 0.0150332 | 0.468311 | 0.001134557 | 20.82913811 |
|  |  | rs12528285 | C | T | 0.0944 | 0.0809469 | 0.0180784 | 5.69E-06 | -0.0291777 | 0.0239127 | 0.222399 | 0.001120312 | 20.56731657 |
|  |  | rs13192624 | T | C | 0.2783 | -0.0522862 | 0.0117742 | 9.33E-06 | 0.0401581 | 0.0174336 | 0.021251 | 0.001098181 | 20.16058971 |
|  |  | rs1397793 | A | G | 0.6143 | 0.0521387 | 0.0111587 | 3.74E-06 | -0.0112868 | 0.0164043 | 0.49143 | 0.001288192 | 23.65333216 |
|  |  | rs4429415 | C | T | 0.3569 | 0.0581908 | 0.0111343 | 2.05E-07 | 0.0230247 | 0.0150467 | 0.125963 | 0.001554403 | 28.54902437 |
|  |  | rs55888705 | A | G | 0.3867 | 0.0534221 | 0.0109975 | 1.31E-06 | -0.00807341 | 0.0166211 | 0.627157 | 0.001353689 | 24.85760662 |
|  |  | rs6496870 | C | T | 0.6451 | -0.0510777 | 0.0113809 | 4.62E-06 | -0.00487305 | 0.0155972 | 0.754713 | 0.001194609 | 21.93293711 |
|  |  | rs6743026 | T | C | 0.174 | 0.0589446 | 0.0134622 | 9.88E-06 | 0.00893935 | 0.0190438 | 0.638777 | 0.000998728 | 18.3329886 |
|  |  | rs74037001 | G | A | 0.1113 | -0.0819391 | 0.0165334 | 6.71E-07 | -0.00491152 | 0.026277 | 0.851729 | 0.001328198 | 24.38888241 |
|  |  | rs75211493 | G | A | 0.0716 | 0.0840786 | 0.0184739 | 9.27E-06 | 0.0150156 | 0.0288348 | 0.602543 | 0.00093983 | 17.25080699 |
|  |  | rs7570971 | A | C | 0.5099 | 0.086653 | 0.0113623 | 1.41E-14 | 0.0232562 | 0.0153522 | 0.129811 | 0.003752899 | 69.07991809 |
|  |  | rs80124826 | T | C | 0.0626 | -0.124191 | 0.0278566 | 8.75E-06 | 0.00034626 | 0.0457743 | 0.993964 | 0.001810129 | 33.25434025 |
|  |  | rs857444 | C | T | 0.3608 | 0.0507315 | 0.0109885 | 3.80E-06 | 0.0187018 | 0.0154955 | 0.227463 | 0.001187104 | 21.79498252 |
|  |  | rs9833771 | C | T | 0.4423 | -0.0490077 | 0.0106564 | 4.07E-06 | 0.00664733 | 0.0150376 | 0.658455 | 0.001184885 | 21.75419835 |
| ebi-a-GCST90017113 | Gut microbiota abundance (phylum Euryarchaeota id.55) | rs10202904 | G | T | 0.5328 | 0.115986 | 0.0230271 | 6.19E-07 | 0.00541439 | 0.0152871 | 0.723204 | 0.00669743 | 123.6455804 |
|  |  | rs11022995 | A | G | 0.5467 | 0.10373 | 0.0228889 | 7.73E-06 | 0.00474509 | 0.0149458 | 0.750874 | 0.005333024 | 98.32134563 |
|  |  | rs34928225 | T | C | 0.0755 | 0.19977 | 0.0425421 | 4.33E-06 | -0.00499497 | 0.0256483 | 0.84559 | 0.005571144 | 102.736 |
|  |  | rs45498998 | G | A | 0.1849 | -0.131882 | 0.029229 | 5.32E-06 | 0.00498423 | 0.0206551 | 0.809317 | 0.005242626 | 96.64594797 |
|  |  | rs6064552 | T | C | 0.1978 | -0.123612 | 0.0275749 | 9.34E-06 | 0.0215998 | 0.0192593 | 0.262064 | 0.00484909 | 89.35589945 |
|  |  | rs6508769 | C | T | 0.835 | -0.150529 | 0.0337091 | 8.12E-06 | 0.0221915 | 0.0211458 | 0.293969 | 0.006243682 | 115.2160107 |
|  |  | rs7015093 | G | A | 0.2634 | -0.118111 | 0.0264389 | 7.20E-06 | 0.0233658 | 0.0170333 | 0.170136 | 0.005413251 | 99.80848732 |
|  |  | rs76029318 | T | C | 0.0646 | 0.214866 | 0.0438442 | 1.05E-06 | -0.0100264 | 0.0308017 | 0.74479 | 0.0055795 | 102.8909499 |
|  |  | rs7635189 | A | G | 0.669 | -0.119983 | 0.0259356 | 4.64E-06 | 0.000470836 | 0.0166756 | 0.977475 | 0.006375636 | 117.6666196 |
|  |  | rs77658038 | A | C | 0.1173 | -0.160157 | 0.0340856 | 4.75E-06 | -0.0100918 | 0.0184925 | 0.585256 | 0.005311693 | 97.92598318 |
|  |  | rs894996 | C | A | 0.0716 | 0.203577 | 0.0440033 | 5.12E-06 | -0.0376031 | 0.0292061 | 0.197917 | 0.005509797 | 101.5984375 |
| ebi-a-GCST90017116 | Gut microbiota abundance (phylum Proteobacteria id.2375) | rs10750258 | C | A | 0.6064 | 0.0491248 | 0.010824 | 8.72E-06 | 0.0227848 | 0.01549 | 0.141307 | 0.001151982 | 21.14941821 |
|  |  | rs11126162 | T | C | 0.0885 | -0.0770689 | 0.0187079 | 9.26E-06 | -0.015274 | 0.0272292 | 0.574837 | 0.000958271 | 17.58962577 |
|  |  | rs11715072 | G | A | 0.2893 | -0.0519289 | 0.0115246 | 6.90E-06 | -0.00534992 | 0.0162614 | 0.742159 | 0.001108876 | 20.35714197 |
|  |  | rs12150865 | C | T | 0.4751 | 0.0511615 | 0.0106407 | 1.54E-06 | 0.00277082 | 0.0152058 | 0.855409 | 0.001305504 | 23.97162354 |
|  |  | rs12467198 | C | T | 0.3419 | 0.0498267 | 0.011138 | 6.31E-06 | 0.0248336 | 0.0151016 | 0.100087 | 0.001117237 | 20.5108041 |
|  |  | rs2347697 | G | T | 0.3181 | 0.0502281 | 0.0108968 | 4.27E-06 | 0.0167524 | 0.0161412 | 0.29933 | 0.00109448 | 20.09256634 |
|  |  | rs2532663 | A | G | 0.9374 | 0.125578 | 0.0258034 | 7.47E-07 | -0.027866 | 0.0249228 | 0.263529 | 0.001850787 | 34.0026607 |
|  |  | rs312757 | G | C | 0.7018 | 0.0517269 | 0.0112854 | 4.84E-06 | 0.0188143 | 0.0151636 | 0.214698 | 0.001119912 | 20.55997181 |
|  |  | rs3890996 | G | T | 0.4612 | 0.0474614 | 0.0105645 | 6.95E-06 | -0.0283206 | 0.0149982 | 0.0589902 | 0.00111951 | 20.5525829 |
|  |  | rs4340090 | C | T | 0.1252 | -0.0668162 | 0.0153387 | 9.99E-06 | 0.0126296 | 0.0231278 | 0.585012 | 0.000977927 | 17.95078845 |
|  |  | rs6707783 | C | T | 0.1153 | 0.0849594 | 0.0187782 | 8.09E-06 | 0.0216321 | 0.0259366 | 0.404261 | 0.001472578 | 27.0439532 |
|  |  | rs72771021 | C | T | 0.0497 | 0.141726 | 0.0309242 | 7.18E-06 | -0.0190632 | 0.0298063 | 0.522454 | 0.001897344 | 34.85964266 |
|  |  | rs74757828 | A | T | 0.0577 | 0.0951285 | 0.0216921 | 9.25E-06 | 0.0109928 | 0.028275 | 0.697439 | 0.000984048 | 18.06324793 |
|  |  | rs922773 | C | T | 0.1402 | -0.0803778 | 0.0157851 | 3.68E-07 | -0.00719226 | 0.0248062 | 0.771863 | 0.00155757 | 28.60728352 |
| ebi-a-GCST90017117 | Gut microbiota abundance (phylum Tenericutes id.3919) | rs10108398 | G | A | 0.2913 | 0.0769142 | 0.0153953 | 1.09E-06 | -0.0112563 | 0.0167303 | 0.501067 | 0.002442564 | 44.90141482 |
|  |  | rs11890098 | A | G | 0.2555 | 0.074438 | 0.0153389 | 9.57E-07 | -0.0173315 | 0.0167206 | 0.299951 | 0.002108021 | 38.73855625 |
|  |  | rs12566890 | T | G | 0.0954 | -0.101147 | 0.0230978 | 3.65E-06 | 0.0279033 | 0.0222866 | 0.210563 | 0.001765798 | 32.43847946 |
|  |  | rs17214486 | C | A | 0.4016 | 0.06099 | 0.0135623 | 6.61E-06 | -0.0220373 | 0.0160201 | 0.168945 | 0.001787856 | 32.84442557 |
|  |  | rs2464826 | A | C | 0.1262 | 0.0944239 | 0.0211806 | 8.39E-06 | -0.00940638 | 0.0236645 | 0.691006 | 0.00196637 | 36.1303403 |
|  |  | rs28537087 | G | A | 0.164 | 0.0820873 | 0.0188312 | 8.07E-06 | -0.00937581 | 0.0174352 | 0.590749 | 0.001847703 | 33.94589169 |
|  |  | rs3768491 | G | A | 0.7137 | 0.0681052 | 0.0149061 | 4.23E-06 | -0.00487214 | 0.0164616 | 0.767252 | 0.001895517 | 34.82599615 |
|  |  | rs4885016 | C | T | 0.8429 | 0.0819606 | 0.0181628 | 7.27E-06 | 0.0360183 | 0.0220069 | 0.101697 | 0.001779068 | 32.68268953 |
|  |  | rs6043847 | T | C | 0.0696 | -0.114937 | 0.0248606 | 4.55E-06 | -0.00375842 | 0.0319114 | 0.906245 | 0.001710916 | 31.4285466 |
|  |  | rs72901605 | T | C | 0.1859 | -0.0841852 | 0.0178119 | 3.26E-06 | -0.0451736 | 0.023464 | 0.0542001 | 0.002145155 | 39.42241579 |
|  |  | rs74603314 | T | C | 0.0457 | 0.221639 | 0.0462918 | 1.56E-06 | -0.0621365 | 0.0382965 | 0.104694 | 0.00428473 | 78.91149744 |
|  |  | rs78169027 | A | G | 0.1083 | -0.108283 | 0.0237289 | 5.88E-06 | -0.0391958 | 0.0313705 | 0.211502 | 0.002264633 | 41.62309585 |

| Supplementary Table 3: All results of MR analysIs | | | | | | | | | |
| --- | --- | --- | --- | --- | --- | --- | --- | --- | --- |
| id.exposure | Gut microbiota | id.outcome | method | nsnp | se | pval | or | or_lci95 | or_uci95 |
| ebi-a-GCST90016908 | class Actinobacteria id.419 | finngen_R9_L12_PSORIASIS | Inverse variance weighted | 17 | 0.081461502 | 0.64707722 | 1.037999414 | 0.884821522 | 1.217695045 |
| ebi-a-GCST90016908 | class Actinobacteria id.419 | finngen_R9_L13_PSORIASIS | MR Egger | 17 | 0.252651992 | 0.725645316 | 0.913619435 | 0.556805622 | 1.499087722 |
| ebi-a-GCST90016908 | class Actinobacteria id.419 | finngen_R9_L14_PSORIASIS | Simple mode | 17 | 0.182426703 | 0.686868469 | 1.077765667 | 0.753771304 | 1.541022889 |
| ebi-a-GCST90016908 | class Actinobacteria id.419 | finngen_R9_L15_PSORIASIS | Weighted median | 17 | 0.094003695 | 0.35668755 | 1.090507038 | 0.907007572 | 1.31113084 |
| ebi-a-GCST90016908 | class Actinobacteria id.419 | finngen_R9_L16_PSORIASIS | Weighted mode | 17 | 0.128130946 | 0.282496424 | 1.153157264 | 0.897059555 | 1.482367216 |
| ebi-a-GCST90016909 | class Alphaproteobacteria id.2379 | finngen_R9_L17_PSORIASIS | Inverse variance weighted | 8 | 0.07524595 | 0.352239228 | 0.932395779 | 0.804543727 | 1.080565121 |
| ebi-a-GCST90016909 | class Alphaproteobacteria id.2379 | finngen_R9_L18_PSORIASIS | MR Egger | 8 | 0.282263148 | 0.831402994 | 0.939162748 | 0.540099301 | 1.633082408 |
| ebi-a-GCST90016909 | class Alphaproteobacteria id.2379 | finngen_R9_L19_PSORIASIS | Simple mode | 8 | 0.157380461 | 0.701790952 | 1.064806966 | 0.782178601 | 1.449558799 |
| ebi-a-GCST90016909 | class Alphaproteobacteria id.2379 | finngen_R9_L20_PSORIASIS | Weighted median | 8 | 0.095781359 | 0.923503565 | 1.009239484 | 0.836495279 | 1.217657005 |
| ebi-a-GCST90016909 | class Alphaproteobacteria id.2379 | finngen_R9_L21_PSORIASIS | Weighted mode | 8 | 0.146457379 | 0.699050098 | 1.060781825 | 0.796084251 | 1.413491197 |
| ebi-a-GCST90016911 | class Bacteroidia id.912 | finngen_R9_L22_PSORIASIS | Inverse variance weighted | 15 | 0.090990353 | 0.377550003 | 1.083603066 | 0.906604088 | 1.295158075 |
| ebi-a-GCST90016911 | class Bacteroidia id.912 | finngen_R9_L23_PSORIASIS | MR Egger | 15 | 0.200425023 | 0.509318922 | 1.145681421 | 0.773497023 | 1.696950189 |
| ebi-a-GCST90016911 | class Bacteroidia id.912 | finngen_R9_L24_PSORIASIS | Simple mode | 15 | 0.154645634 | 0.318854281 | 1.173322857 | 0.866523829 | 1.588746298 |
| ebi-a-GCST90016911 | class Bacteroidia id.912 | finngen_R9_L25_PSORIASIS | Weighted median | 15 | 0.109562325 | 0.409626445 | 1.094546087 | 0.88302443 | 1.356736117 |
| ebi-a-GCST90016911 | class Bacteroidia id.912 | finngen_R9_L26_PSORIASIS | Weighted mode | 15 | 0.143092196 | 0.49394715 | 1.105731528 | 0.835308993 | 1.463700526 |
| ebi-a-GCST90016914 | class Coriobacteriia id.809 | finngen_R9_L27_PSORIASIS | Inverse variance weighted | 16 | 0.06871917 | 0.515696282 | 1.04567962 | 0.913910581 | 1.196447323 |
| ebi-a-GCST90016914 | class Coriobacteriia id.809 | finngen_R9_L28_PSORIASIS | MR Egger | 16 | 0.173708399 | 0.546552162 | 1.11330947 | 0.792049508 | 1.564874371 |
| ebi-a-GCST90016914 | class Coriobacteriia id.809 | finngen_R9_L29_PSORIASIS | Simple mode | 16 | 0.160153315 | 0.870399387 | 0.973770007 | 0.711428304 | 1.33285114 |
| ebi-a-GCST90016914 | class Coriobacteriia id.809 | finngen_R9_L30_PSORIASIS | Weighted median | 16 | 0.098279045 | 0.46780056 | 1.073964254 | 0.885794572 | 1.302106895 |
| ebi-a-GCST90016914 | class Coriobacteriia id.809 | finngen_R9_L31_PSORIASIS | Weighted mode | 16 | 0.125549053 | 0.764734065 | 1.039004198 | 0.812358616 | 1.328883208 |
| ebi-a-GCST90016915 | class Deltaproteobacteria id.3087 | finngen_R9_L32_PSORIASIS | Inverse variance weighted | 13 | 0.079159812 | 0.222792828 | 1.101316766 | 0.94303988 | 1.286158354 |
| ebi-a-GCST90016915 | class Deltaproteobacteria id.3087 | finngen_R9_L33_PSORIASIS | MR Egger | 13 | 0.370817809 | 0.469158039 | 1.32049594 | 0.63839662 | 2.731389033 |
| ebi-a-GCST90016915 | class Deltaproteobacteria id.3087 | finngen_R9_L34_PSORIASIS | Simple mode | 13 | 0.177532196 | 0.475999325 | 1.139540593 | 0.80465802 | 1.613794593 |
| ebi-a-GCST90016915 | class Deltaproteobacteria id.3087 | finngen_R9_L35_PSORIASIS | Weighted median | 13 | 0.104595756 | 0.19820734 | 1.144057117 | 0.931995901 | 1.404369575 |
| ebi-a-GCST90016915 | class Deltaproteobacteria id.3087 | finngen_R9_L36_PSORIASIS | Weighted mode | 13 | 0.166832497 | 0.476245599 | 1.130525426 | 0.815210276 | 1.567801309 |
| ebi-a-GCST90016918 | class Lentisphaeria id.2250 | finngen_R9_L37_PSORIASIS | Inverse variance weighted | 10 | 0.049653059 | 0.211762963 | 0.939879706 | 0.852720561 | 1.03594765 |
| ebi-a-GCST90016918 | class Lentisphaeria id.2250 | finngen_R9_L38_PSORIASIS | MR Egger | 10 | 0.193564208 | 0.981833152 | 1.00455758 | 0.687400201 | 1.468047187 |
| ebi-a-GCST90016918 | class Lentisphaeria id.2250 | finngen_R9_L39_PSORIASIS | Simple mode | 10 | 0.123098564 | 0.294078462 | 0.871838061 | 0.684939499 | 1.109735394 |
| ebi-a-GCST90016918 | class Lentisphaeria id.2250 | finngen_R9_L40_PSORIASIS | Weighted median | 10 | 0.068160684 | 0.452159982 | 0.950046365 | 0.831237744 | 1.085836276 |
| ebi-a-GCST90016918 | class Lentisphaeria id.2250 | finngen_R9_L41_PSORIASIS | Weighted mode | 10 | 0.126665013 | 0.762072914 | 1.040324152 | 0.811613469 | 1.333484944 |
| ebi-a-GCST90016920 | class Methanobacteria id.119 | finngen_R9_L42_PSORIASIS | Inverse variance weighted | 11 | 0.043528374 | 0.263688151 | 0.952512189 | 0.874618063 | 1.037343623 |
| ebi-a-GCST90016920 | class Methanobacteria id.119 | finngen_R9_L43_PSORIASIS | MR Egger | 11 | 0.162025833 | 0.912443853 | 1.018491924 | 0.741375819 | 1.399190226 |
| ebi-a-GCST90016920 | class Methanobacteria id.119 | finngen_R9_L44_PSORIASIS | Simple mode | 11 | 0.087060176 | 0.222467106 | 0.892921017 | 0.752845619 | 1.059059018 |
| ebi-a-GCST90016920 | class Methanobacteria id.119 | finngen_R9_L45_PSORIASIS | Weighted median | 11 | 0.058399399 | 0.281955696 | 0.939099314 | 0.837531116 | 1.052984783 |
| ebi-a-GCST90016920 | class Methanobacteria id.119 | finngen_R9_L46_PSORIASIS | Weighted mode | 11 | 0.082186881 | 0.192171359 | 0.8914134 | 0.758787683 | 1.04722028 |
| ebi-a-GCST90016921 | class Mollicutes id.3920 | finngen_R9_L47_PSORIASIS | Inverse variance weighted | 12 | 0.079351762 | 0.362973592 | 0.930356555 | 0.796349733 | 1.086913556 |
| ebi-a-GCST90016921 | class Mollicutes id.3920 | finngen_R9_L48_PSORIASIS | MR Egger | 12 | 0.281337289 | 0.593133979 | 0.856183393 | 0.493273364 | 1.486092816 |
| ebi-a-GCST90016921 | class Mollicutes id.3920 | finngen_R9_L49_PSORIASIS | Simple mode | 12 | 0.132169862 | 0.227412316 | 0.844533952 | 0.651796248 | 1.094264656 |
| ebi-a-GCST90016921 | class Mollicutes id.3920 | finngen_R9_L50_PSORIASIS | Weighted median | 12 | 0.089549723 | 0.14438288 | 0.87747013 | 0.736217401 | 1.045824002 |
| ebi-a-GCST90016921 | class Mollicutes id.3920 | finngen_R9_L51_PSORIASIS | Weighted mode | 12 | 0.121464046 | 0.155732989 | 0.831013628 | 0.654961636 | 1.054387939 |
| ebi-a-GCST90016924 | family Acidaminococcaceae id.2166 | finngen_R9_L52_PSORIASIS | Inverse variance weighted | 8 | 0.12831447 | 0.92642938 | 0.988221594 | 0.768476972 | 1.2708018 |
| ebi-a-GCST90016924 | family Acidaminococcaceae id.2166 | finngen_R9_L53_PSORIASIS | MR Egger | 8 | 0.339352979 | 0.228927835 | 1.57545957 | 0.810110339 | 3.063870112 |
| ebi-a-GCST90016924 | family Acidaminococcaceae id.2166 | finngen_R9_L54_PSORIASIS | Simple mode | 8 | 0.270003414 | 0.595247976 | 0.86050281 | 0.506898198 | 1.460776718 |
| ebi-a-GCST90016924 | family Acidaminococcaceae id.2166 | finngen_R9_L55_PSORIASIS | Weighted median | 8 | 0.124114308 | 0.783001571 | 0.966395342 | 0.757716242 | 1.232545781 |
| ebi-a-GCST90016924 | family Acidaminococcaceae id.2166 | finngen_R9_L56_PSORIASIS | Weighted mode | 8 | 0.227853245 | 0.341744354 | 1.261595324 | 0.807174573 | 1.971844524 |
| ebi-a-GCST90016925 | family Actinomycetaceae id.421 | finngen_R9_L57_PSORIASIS | Inverse variance weighted | 4 | 0.110620789 | 0.939553279 | 1.008423805 | 0.811859305 | 1.252579806 |
| ebi-a-GCST90016925 | family Actinomycetaceae id.421 | finngen_R9_L58_PSORIASIS | MR Egger | 4 | 0.292891458 | 0.865145275 | 1.057992525 | 0.595893101 | 1.878437897 |
| ebi-a-GCST90016925 | family Actinomycetaceae id.421 | finngen_R9_L59_PSORIASIS | Simple mode | 4 | 0.137897692 | 0.776946582 | 0.958171452 | 0.731244044 | 1.25552138 |
| ebi-a-GCST90016925 | family Actinomycetaceae id.421 | finngen_R9_L60_PSORIASIS | Weighted median | 4 | 0.110647745 | 0.760139643 | 0.966783719 | 0.778294688 | 1.200921416 |
| ebi-a-GCST90016925 | family Actinomycetaceae id.421 | finngen_R9_L61_PSORIASIS | Weighted mode | 4 | 0.11255725 | 0.729509316 | 0.958171452 | 0.768479987 | 1.194686325 |
| ebi-a-GCST90016927 | family Bacteroidaceae id.917 | finngen_R9_L62_PSORIASIS | Inverse variance weighted | 11 | 0.135214748 | 0.323388006 | 0.875003662 | 0.671293981 | 1.140530722 |
| ebi-a-GCST90016927 | family Bacteroidaceae id.917 | finngen_R9_L63_PSORIASIS | MR Egger | 11 | 0.660807613 | 0.082848009 | 0.275501012 | 0.075445337 | 1.006037094 |
| ebi-a-GCST90016927 | family Bacteroidaceae id.917 | finngen_R9_L64_PSORIASIS | Simple mode | 11 | 0.208971071 | 0.978920675 | 0.994354895 | 0.660178915 | 1.497687423 |
| ebi-a-GCST90016927 | family Bacteroidaceae id.917 | finngen_R9_L65_PSORIASIS | Weighted median | 11 | 0.131107914 | 0.515368639 | 0.918249781 | 0.710165424 | 1.18730458 |
| ebi-a-GCST90016927 | family Bacteroidaceae id.917 | finngen_R9_L66_PSORIASIS | Weighted mode | 11 | 0.188293699 | 0.774455056 | 0.946070509 | 0.654100789 | 1.368366196 |
| ebi-a-GCST90016928 | family Bacteroidales S24 7group id.11173 | finngen_R9_L67_PSORIASIS | Inverse variance weighted | 10 | 0.062150999 | 0.464379775 | 0.955545375 | 0.845955106 | 1.079332647 |
| ebi-a-GCST90016928 | family Bacteroidales S24 7group id.11173 | finngen_R9_L68_PSORIASIS | MR Egger | 10 | 0.265796916 | 0.231531346 | 0.70883404 | 0.421011213 | 1.193425925 |
| ebi-a-GCST90016928 | family Bacteroidales S24 7group id.11173 | finngen_R9_L69_PSORIASIS | Simple mode | 10 | 0.135162455 | 0.425423203 | 0.893296784 | 0.685398526 | 1.164255705 |
| ebi-a-GCST90016928 | family Bacteroidales S24 7group id.11173 | finngen_R9_L70_PSORIASIS | Weighted median | 10 | 0.083561232 | 0.324776481 | 0.921012298 | 0.781873821 | 1.084911183 |
| ebi-a-GCST90016928 | family Bacteroidales S24 7group id.11173 | finngen_R9_L71_PSORIASIS | Weighted mode | 10 | 0.126642573 | 0.396132116 | 0.893296784 | 0.696940072 | 1.144975266 |
| ebi-a-GCST90016931 | family Clostridiaceae1 id.1869 | finngen_R9_L72_PSORIASIS | Inverse variance weighted | 11 | 0.09771713 | 0.179255842 | 1.140240237 | 0.941494662 | 1.380940169 |
| ebi-a-GCST90016931 | family Clostridiaceae1 id.1869 | finngen_R9_L73_PSORIASIS | MR Egger | 11 | 0.308326101 | 0.906556061 | 1.037926206 | 0.56717098 | 1.899411019 |
| ebi-a-GCST90016931 | family Clostridiaceae1 id.1869 | finngen_R9_L74_PSORIASIS | Simple mode | 11 | 0.161989325 | 0.14936401 | 1.287907213 | 0.937554404 | 1.769182655 |
| ebi-a-GCST90016931 | family Clostridiaceae1 id.1869 | finngen_R9_L75_PSORIASIS | Weighted median | 11 | 0.111179541 | 0.037324313 | 1.260486692 | 1.013678719 | 1.567386856 |
| ebi-a-GCST90016931 | family Clostridiaceae1 id.1869 | finngen_R9_L76_PSORIASIS | Weighted mode | 11 | 0.152287058 | 0.127603968 | 1.287907213 | 0.955553956 | 1.735856965 |
| ebi-a-GCST90016934 | family Defluviitaleaceae id.1924 | finngen_R9_L77_PSORIASIS | Inverse variance weighted | 12 | 0.070310444 | 0.622616982 | 0.965988916 | 0.841632863 | 1.108719283 |
| ebi-a-GCST90016934 | family Defluviitaleaceae id.1924 | finngen_R9_L78_PSORIASIS | MR Egger | 12 | 0.261153829 | 0.886031395 | 1.039143793 | 0.622840753 | 1.7337013 |
| ebi-a-GCST90016934 | family Defluviitaleaceae id.1924 | finngen_R9_L79_PSORIASIS | Simple mode | 12 | 0.127542221 | 0.804209559 | 1.032921151 | 0.804453675 | 1.326274137 |
| ebi-a-GCST90016934 | family Defluviitaleaceae id.1924 | finngen_R9_L80_PSORIASIS | Weighted median | 12 | 0.085128035 | 0.953967967 | 1.004926079 | 0.850494816 | 1.187398682 |
| ebi-a-GCST90016934 | family Defluviitaleaceae id.1924 | finngen_R9_L81_PSORIASIS | Weighted mode | 12 | 0.134688179 | 0.995071352 | 1.000851456 | 0.768636021 | 1.303222344 |
| ebi-a-GCST90016937 | family Erysipelotrichaceae id.2149 | finngen_R9_L82_PSORIASIS | Inverse variance weighted | 13 | 0.08595753 | 0.499793944 | 1.05972066 | 0.895411926 | 1.254180164 |
| ebi-a-GCST90016937 | family Erysipelotrichaceae id.2149 | finngen_R9_L83_PSORIASIS | MR Egger | 13 | 0.37199683 | 0.671746697 | 0.850493416 | 0.410223808 | 1.763279061 |
| ebi-a-GCST90016937 | family Erysipelotrichaceae id.2149 | finngen_R9_L84_PSORIASIS | Simple mode | 13 | 0.195888354 | 0.476062446 | 1.155011459 | 0.786760884 | 1.695625059 |
| ebi-a-GCST90016937 | family Erysipelotrichaceae id.2149 | finngen_R9_L85_PSORIASIS | Weighted median | 13 | 0.118318281 | 0.190580632 | 1.167497379 | 0.925851555 | 1.47221239 |
| ebi-a-GCST90016937 | family Erysipelotrichaceae id.2149 | finngen_R9_L86_PSORIASIS | Weighted mode | 13 | 0.195818334 | 0.403449075 | 1.184815936 | 0.807173612 | 1.739141099 |
| ebi-a-GCST90016938 | family Family XI id.1936 | finngen_R9_L87_PSORIASIS | Inverse variance weighted | 10 | 0.039333022 | 0.753819448 | 0.98774065 | 0.914454251 | 1.066900384 |
| ebi-a-GCST90016938 | family Family XI id.1936 | finngen_R9_L88_PSORIASIS | MR Egger | 10 | 0.203150075 | 0.607009407 | 1.114875429 | 0.748689098 | 1.660164713 |
| ebi-a-GCST90016938 | family Family XI id.1936 | finngen_R9_L89_PSORIASIS | Simple mode | 10 | 0.093906523 | 0.322857994 | 0.90644741 | 0.754063234 | 1.089626002 |
| ebi-a-GCST90016938 | family Family XI id.1936 | finngen_R9_L90_PSORIASIS | Weighted median | 10 | 0.053592807 | 0.488278104 | 0.96353882 | 0.867461279 | 1.070257637 |
| ebi-a-GCST90016938 | family Family XI id.1936 | finngen_R9_L91_PSORIASIS | Weighted mode | 10 | 0.099350155 | 0.355422311 | 0.907751327 | 0.747133712 | 1.102898261 |
| ebi-a-GCST90016940 | family Lachnospiraceae id.1987 | finngen_R9_L92_PSORIASIS | Inverse variance weighted | 16 | 0.083461132 | 0.688314096 | 1.034046694 | 0.878004215 | 1.217821676 |
| ebi-a-GCST90016940 | family Lachnospiraceae id.1987 | finngen_R9_L93_PSORIASIS | MR Egger | 16 | 0.278968023 | 0.200742024 | 0.687588756 | 0.397984713 | 1.187930796 |
| ebi-a-GCST90016940 | family Lachnospiraceae id.1987 | finngen_R9_L94_PSORIASIS | Simple mode | 16 | 0.208866483 | 0.460687372 | 1.171334611 | 0.777839936 | 1.763890883 |
| ebi-a-GCST90016940 | family Lachnospiraceae id.1987 | finngen_R9_L95_PSORIASIS | Weighted median | 16 | 0.108653139 | 0.385514618 | 1.098875136 | 0.888098074 | 1.359677045 |
| ebi-a-GCST90016940 | family Lachnospiraceae id.1987 | finngen_R9_L96_PSORIASIS | Weighted mode | 16 | 0.196204197 | 0.444395126 | 1.166627571 | 0.794181657 | 1.713738762 |
| ebi-a-GCST90016943 | family Oxalobacteraceae id.2966 | finngen_R9_L97_PSORIASIS | Inverse variance weighted | 15 | 0.040220362 | 0.871337279 | 0.993507077 | 0.918194531 | 1.074996941 |
| ebi-a-GCST90016943 | family Oxalobacteraceae id.2966 | finngen_R9_L98_PSORIASIS | MR Egger | 15 | 0.157709093 | 0.816667049 | 1.038016141 | 0.762007799 | 1.413998007 |
| ebi-a-GCST90016943 | family Oxalobacteraceae id.2966 | finngen_R9_L99_PSORIASIS | Simple mode | 15 | 0.103628652 | 0.272602564 | 0.888388673 | 0.725091014 | 1.088462578 |
| ebi-a-GCST90016943 | family Oxalobacteraceae id.2966 | finngen_R9_L100_PSORIASIS | Weighted median | 15 | 0.058665623 | 0.621227733 | 0.971428768 | 0.865912031 | 1.089803372 |
| ebi-a-GCST90016943 | family Oxalobacteraceae id.2966 | finngen_R9_L101_PSORIASIS | Weighted mode | 15 | 0.101762061 | 0.293075683 | 0.894786082 | 0.73298925 | 1.092297237 |
| ebi-a-GCST90016944 | family Pasteurellaceae id.3689 | finngen_R9_L102_PSORIASIS | Inverse variance weighted | 16 | 0.04854362 | 0.683003762 | 0.980371551 | 0.891393651 | 1.078231124 |
| ebi-a-GCST90016944 | family Pasteurellaceae id.3689 | finngen_R9_L103_PSORIASIS | MR Egger | 16 | 0.113023972 | 0.948656607 | 1.007437157 | 0.80725365 | 1.257262355 |
| ebi-a-GCST90016944 | family Pasteurellaceae id.3689 | finngen_R9_L104_PSORIASIS | Simple mode | 16 | 0.107723364 | 0.727047222 | 1.039056316 | 0.841284927 | 1.283320303 |
| ebi-a-GCST90016944 | family Pasteurellaceae id.3689 | finngen_R9_L105_PSORIASIS | Weighted median | 16 | 0.069074772 | 0.559933708 | 1.041088131 | 0.909263719 | 1.192024352 |
| ebi-a-GCST90016944 | family Pasteurellaceae id.3689 | finngen_R9_L106_PSORIASIS | Weighted mode | 16 | 0.091549489 | 0.604450851 | 1.049635074 | 0.877222648 | 1.255934045 |
| ebi-a-GCST90016947 | family Porphyromonadaceae id.943 | finngen_R9_L107_PSORIASIS | Inverse variance weighted | 10 | 0.117187584 | 0.371170873 | 0.900505704 | 0.715705546 | 1.133022548 |
| ebi-a-GCST90016947 | family Porphyromonadaceae id.943 | finngen_R9_L108_PSORIASIS | MR Egger | 10 | 0.55190988 | 0.646799524 | 0.768964002 | 0.260681874 | 2.268303607 |
| ebi-a-GCST90016947 | family Porphyromonadaceae id.943 | finngen_R9_L109_PSORIASIS | Simple mode | 10 | 0.261041988 | 0.462927383 | 1.221557119 | 0.732335949 | 2.037591898 |
| ebi-a-GCST90016947 | family Porphyromonadaceae id.943 | finngen_R9_L110_PSORIASIS | Weighted median | 10 | 0.142803147 | 0.658280407 | 0.938793068 | 0.709599632 | 1.242013644 |
| ebi-a-GCST90016947 | family Porphyromonadaceae id.943 | finngen_R9_L111_PSORIASIS | Weighted mode | 10 | 0.257262511 | 0.48665633 | 1.205157947 | 0.727876501 | 1.995401248 |
| ebi-a-GCST90016950 | family Rikenellaceae id.967 | finngen_R9_L112_PSORIASIS | Inverse variance weighted | 17 | 0.085429292 | 0.376644407 | 1.078453093 | 0.91218385 | 1.275029232 |
| ebi-a-GCST90016950 | family Rikenellaceae id.967 | finngen_R9_L113_PSORIASIS | MR Egger | 17 | 0.272615481 | 0.309749169 | 1.331903934 | 0.780580898 | 2.272625546 |
| ebi-a-GCST90016950 | family Rikenellaceae id.967 | finngen_R9_L114_PSORIASIS | Simple mode | 17 | 0.155762839 | 0.520735251 | 1.107696591 | 0.816268081 | 1.503172508 |
| ebi-a-GCST90016950 | family Rikenellaceae id.967 | finngen_R9_L115_PSORIASIS | Weighted median | 17 | 0.09713028 | 0.254980404 | 1.116911011 | 0.92329314 | 1.351131241 |
| ebi-a-GCST90016950 | family Rikenellaceae id.967 | finngen_R9_L116_PSORIASIS | Weighted mode | 17 | 0.151237682 | 0.555385968 | 1.095375469 | 0.81437962 | 1.473326923 |
| ebi-a-GCST90016953 | unknown family id.1000001214 | finngen_R9_L117_PSORIASIS | Inverse variance weighted | 12 | 0.057274009 | 0.477736844 | 0.960154063 | 0.858199591 | 1.074220768 |
| ebi-a-GCST90016953 | unknown family id.1000001214 | finngen_R9_L118_PSORIASIS | MR Egger | 12 | 0.175234396 | 0.29932035 | 1.211460094 | 0.859303505 | 1.707936194 |
| ebi-a-GCST90016953 | unknown family id.1000001214 | finngen_R9_L119_PSORIASIS | Simple mode | 12 | 0.111045859 | 0.24693014 | 0.873021405 | 0.702264555 | 1.085298082 |
| ebi-a-GCST90016953 | unknown family id.1000001214 | finngen_R9_L120_PSORIASIS | Weighted median | 12 | 0.068209131 | 0.162024692 | 0.909030672 | 0.795275774 | 1.039056878 |
| ebi-a-GCST90016953 | unknown family id.1000001214 | finngen_R9_L121_PSORIASIS | Weighted mode | 12 | 0.114150606 | 0.259241721 | 0.873021405 | 0.698004037 | 1.091922588 |
| ebi-a-GCST90016954 | unknown family id.1000005471 | finngen_R9_L122_PSORIASIS | Inverse variance weighted | 14 | 0.075953812 | 0.921770519 | 0.992568801 | 0.855278254 | 1.151897432 |
| ebi-a-GCST90016954 | unknown family id.1000005471 | finngen_R9_L123_PSORIASIS | MR Egger | 14 | 0.23068328 | 0.39899506 | 0.817308002 | 0.520024947 | 1.284539086 |
| ebi-a-GCST90016954 | unknown family id.1000005471 | finngen_R9_L124_PSORIASIS | Simple mode | 14 | 0.166722145 | 0.283813906 | 0.829949383 | 0.598597435 | 1.150716555 |
| ebi-a-GCST90016954 | unknown family id.1000005471 | finngen_R9_L125_PSORIASIS | Weighted median | 14 | 0.091913926 | 0.616621698 | 0.955026534 | 0.797584535 | 1.143547349 |
| ebi-a-GCST90016954 | unknown family id.1000005471 | finngen_R9_L126_PSORIASIS | Weighted mode | 14 | 0.142796552 | 0.241399853 | 0.839220005 | 0.634344164 | 1.110265148 |
| ebi-a-GCST90016956 | family Veillonellaceae id.2172 | finngen_R9_L127_PSORIASIS | Inverse variance weighted | 20 | 0.057447754 | 0.008940511 | 1.162051346 | 1.038304658 | 1.300546346 |
| ebi-a-GCST90016956 | family Veillonellaceae id.2172 | finngen_R9_L128_PSORIASIS | MR Egger | 20 | 0.116379722 | 0.789993517 | 0.969030595 | 0.771388339 | 1.217312016 |
| ebi-a-GCST90016956 | family Veillonellaceae id.2172 | finngen_R9_L129_PSORIASIS | Simple mode | 20 | 0.15108967 | 0.122793634 | 1.276358052 | 0.949210206 | 1.716258281 |
| ebi-a-GCST90016956 | family Veillonellaceae id.2172 | finngen_R9_L130_PSORIASIS | Weighted median | 20 | 0.085515305 | 0.253065265 | 1.102674397 | 0.932513642 | 1.303885296 |
| ebi-a-GCST90016956 | family Veillonellaceae id.2172 | finngen_R9_L131_PSORIASIS | Weighted mode | 20 | 0.098933416 | 0.356217642 | 1.09806846 | 0.904514625 | 1.333040185 |
| ebi-a-GCST90016957 | family Verrucomicrobiaceae id.4036 | finngen_R9_L132_PSORIASIS | Inverse variance weighted | 12 | 0.071742357 | 0.602899217 | 0.963364972 | 0.836994345 | 1.10881522 |
| ebi-a-GCST90016957 | family Verrucomicrobiaceae id.4036 | finngen_R9_L133_PSORIASIS | MR Egger | 12 | 0.259748176 | 0.5999011 | 0.868750228 | 0.52214704 | 1.445429928 |
| ebi-a-GCST90016957 | family Verrucomicrobiaceae id.4036 | finngen_R9_L134_PSORIASIS | Simple mode | 12 | 0.156981083 | 0.811568776 | 0.96239027 | 0.707499648 | 1.309110238 |
| ebi-a-GCST90016957 | family Verrucomicrobiaceae id.4036 | finngen_R9_L135_PSORIASIS | Weighted median | 12 | 0.092545127 | 0.483741023 | 0.937246276 | 0.781767697 | 1.123646558 |
| ebi-a-GCST90016957 | family Verrucomicrobiaceae id.4036 | finngen_R9_L136_PSORIASIS | Weighted mode | 12 | 0.130527617 | 0.673508033 | 0.945070984 | 0.731740476 | 1.220595543 |
| ebi-a-GCST90016960 | genus Adlercreutzia id.812 | finngen_R9_L137_PSORIASIS | Inverse variance weighted | 12 | 0.060118125 | 0.815742785 | 1.01410755 | 0.901385225 | 1.140926315 |
| ebi-a-GCST90016960 | genus Adlercreutzia id.812 | finngen_R9_L138_PSORIASIS | MR Egger | 12 | 0.292665527 | 0.99591796 | 1.001536327 | 0.564345128 | 1.777414147 |
| ebi-a-GCST90016960 | genus Adlercreutzia id.812 | finngen_R9_L139_PSORIASIS | Simple mode | 12 | 0.134693244 | 0.71364347 | 1.052032222 | 0.807933913 | 1.369879117 |
| ebi-a-GCST90016960 | genus Adlercreutzia id.812 | finngen_R9_L140_PSORIASIS | Weighted median | 12 | 0.07734788 | 0.640566638 | 1.036774283 | 0.890931618 | 1.206490928 |
| ebi-a-GCST90016960 | genus Adlercreutzia id.812 | finngen_R9_L141_PSORIASIS | Weighted mode | 12 | 0.128839669 | 0.690595401 | 1.054069373 | 0.818839255 | 1.35687467 |
| ebi-a-GCST90016963 | genus Allisonella id.2174 | finngen_R9_L142_PSORIASIS | Inverse variance weighted | 9 | 0.041504295 | 0.411813061 | 1.034649658 | 0.953815003 | 1.122334951 |
| ebi-a-GCST90016963 | genus Allisonella id.2174 | finngen_R9_L143_PSORIASIS | MR Egger | 9 | 0.271495884 | 0.834840479 | 0.942938574 | 0.55383633 | 1.605407785 |
| ebi-a-GCST90016963 | genus Allisonella id.2174 | finngen_R9_L144_PSORIASIS | Simple mode | 9 | 0.088234165 | 0.966914319 | 0.996231199 | 0.838018682 | 1.184313218 |
| ebi-a-GCST90016963 | genus Allisonella id.2174 | finngen_R9_L145_PSORIASIS | Weighted median | 9 | 0.052667039 | 0.562935079 | 1.030936096 | 0.929823797 | 1.1430437 |
| ebi-a-GCST90016963 | genus Allisonella id.2174 | finngen_R9_L146_PSORIASIS | Weighted mode | 9 | 0.085456155 | 0.712106647 | 1.033220336 | 0.873878789 | 1.221615946 |
| ebi-a-GCST90016966 | genus Anaerostipes id.1991 | finngen_R9_L147_PSORIASIS | Inverse variance weighted | 15 | 0.076482605 | 0.62725057 | 0.963541252 | 0.82940568 | 1.11936989 |
| ebi-a-GCST90016966 | genus Anaerostipes id.1991 | finngen_R9_L148_PSORIASIS | MR Egger | 15 | 0.229193578 | 0.323916563 | 1.264902743 | 0.807167417 | 1.982214488 |
| ebi-a-GCST90016966 | genus Anaerostipes id.1991 | finngen_R9_L149_PSORIASIS | Simple mode | 15 | 0.175869989 | 0.71818597 | 0.937286428 | 0.664001059 | 1.323048868 |
| ebi-a-GCST90016966 | genus Anaerostipes id.1991 | finngen_R9_L150_PSORIASIS | Weighted median | 15 | 0.100520686 | 0.698436677 | 0.961803708 | 0.78980797 | 1.171254796 |
| ebi-a-GCST90016966 | genus Anaerostipes id.1991 | finngen_R9_L151_PSORIASIS | Weighted mode | 15 | 0.177783882 | 0.471692552 | 1.140549682 | 0.804973368 | 1.616020639 |
| ebi-a-GCST90016967 | genus Anaerotruncus id.2054 | finngen_R9_L152_PSORIASIS | Inverse variance weighted | 14 | 0.077165173 | 0.420737631 | 1.064099537 | 0.914740553 | 1.237845881 |
| ebi-a-GCST90016967 | genus Anaerotruncus id.2054 | finngen_R9_L153_PSORIASIS | MR Egger | 14 | 0.234742804 | 0.372316103 | 1.243027485 | 0.784627676 | 1.969236334 |
| ebi-a-GCST90016967 | genus Anaerotruncus id.2054 | finngen_R9_L154_PSORIASIS | Simple mode | 14 | 0.194273458 | 0.253053376 | 1.261564224 | 0.862065884 | 1.846197976 |
| ebi-a-GCST90016967 | genus Anaerotruncus id.2054 | finngen_R9_L155_PSORIASIS | Weighted median | 14 | 0.110405473 | 0.22509057 | 1.143316415 | 0.920846841 | 1.419532941 |
| ebi-a-GCST90016967 | genus Anaerotruncus id.2054 | finngen_R9_L156_PSORIASIS | Weighted mode | 14 | 0.152364613 | 0.241469782 | 1.205624661 | 0.894369018 | 1.625202568 |
| ebi-a-GCST90016972 | genus Blautia id.1992 | finngen_R9_L157_PSORIASIS | Inverse variance weighted | 2 | 0.217399991 | 0.984345819 | 0.995743509 | 0.650268738 | 1.524762114 |
| ebi-a-GCST90016973 | genus Butyricicoccus id.2055 | finngen_R9_L158_PSORIASIS | Inverse variance weighted | 9 | 0.108116522 | 0.075809009 | 1.21162817 | 0.98025422 | 1.497614387 |
| ebi-a-GCST90016973 | genus Butyricicoccus id.2055 | finngen_R9_L159_PSORIASIS | MR Egger | 9 | 0.227915987 | 0.715632529 | 1.090322068 | 0.697507364 | 1.704357935 |
| ebi-a-GCST90016973 | genus Butyricicoccus id.2055 | finngen_R9_L160_PSORIASIS | Simple mode | 9 | 0.147809061 | 0.366438771 | 1.152000844 | 0.862253934 | 1.539112658 |
| ebi-a-GCST90016973 | genus Butyricicoccus id.2055 | finngen_R9_L161_PSORIASIS | Weighted median | 9 | 0.11734258 | 0.285594083 | 1.133491552 | 0.900604821 | 1.42660029 |
| ebi-a-GCST90016973 | genus Butyricicoccus id.2055 | finngen_R9_L162_PSORIASIS | Weighted mode | 9 | 0.139953281 | 0.394407022 | 1.134233583 | 0.862128226 | 1.492220974 |
| ebi-a-GCST90016976 | genus Candidatus Soleaferrea id.11350 | finngen_R9_L163_PSORIASIS | Inverse variance weighted | 11 | 0.053516909 | 0.030414755 | 1.122822043 | 1.01101225 | 1.246997097 |
| ebi-a-GCST90016976 | genus Candidatus Soleaferrea id.11350 | finngen_R9_L164_PSORIASIS | MR Egger | 11 | 0.279637085 | 0.202020832 | 1.469387794 | 0.8493849 | 2.541957704 |
| ebi-a-GCST90016976 | genus Candidatus Soleaferrea id.11350 | finngen_R9_L165_PSORIASIS | Simple mode | 11 | 0.121387704 | 0.134104242 | 1.218829911 | 0.960761969 | 1.546216857 |
| ebi-a-GCST90016976 | genus Candidatus Soleaferrea id.11350 | finngen_R9_L166_PSORIASIS | Weighted median | 11 | 0.070631591 | 0.046995541 | 1.150617071 | 1.001862195 | 1.32145883 |
| ebi-a-GCST90016976 | genus Candidatus Soleaferrea id.11350 | finngen_R9_L167_PSORIASIS | Weighted mode | 11 | 0.130475319 | 0.163708268 | 1.216676394 | 0.942133071 | 1.571223317 |
| ebi-a-GCST90016977 | genus Catenibacterium id.2153 | finngen_R9_L168_PSORIASIS | Inverse variance weighted | 4 | 0.094678377 | 0.571278435 | 1.055066741 | 0.876371149 | 1.270199082 |
| ebi-a-GCST90016977 | genus Catenibacterium id.2153 | finngen_R9_L169_PSORIASIS | MR Egger | 4 | 1.306816839 | 0.546094124 | 2.563675894 | 0.197914753 | 33.20840906 |
| ebi-a-GCST90016977 | genus Catenibacterium id.2153 | finngen_R9_L170_PSORIASIS | Simple mode | 4 | 0.126859262 | 0.663039497 | 0.940733784 | 0.733638234 | 1.206289436 |
| ebi-a-GCST90016977 | genus Catenibacterium id.2153 | finngen_R9_L171_PSORIASIS | Weighted median | 4 | 0.079823491 | 0.903248631 | 0.990343696 | 0.846913029 | 1.158065352 |
| ebi-a-GCST90016977 | genus Catenibacterium id.2153 | finngen_R9_L172_PSORIASIS | Weighted mode | 4 | 0.116042526 | 0.654756297 | 0.94420749 | 0.752125043 | 1.185345168 |
| ebi-a-GCST90016979 | genus Clostridium innocuum group id.14397 | finngen_R9_L173_PSORIASIS | Inverse variance weighted | 10 | 0.04452883 | 0.288307066 | 1.048418267 | 0.960795309 | 1.144032295 |
| ebi-a-GCST90016979 | genus Clostridium innocuum group id.14397 | finngen_R9_L174_PSORIASIS | MR Egger | 10 | 0.234382189 | 0.127073422 | 0.67096868 | 0.423830397 | 1.062214914 |
| ebi-a-GCST90016979 | genus Clostridium innocuum group id.14397 | finngen_R9_L175_PSORIASIS | Simple mode | 10 | 0.08403834 | 0.497782187 | 1.061167505 | 0.900013583 | 1.2511772 |
| ebi-a-GCST90016979 | genus Clostridium innocuum group id.14397 | finngen_R9_L176_PSORIASIS | Weighted median | 10 | 0.061235394 | 0.600699339 | 1.032569447 | 0.915787372 | 1.164243683 |
| ebi-a-GCST90016979 | genus Clostridium innocuum group id.14397 | finngen_R9_L177_PSORIASIS | Weighted mode | 10 | 0.079161826 | 0.559312964 | 1.049163995 | 0.898378752 | 1.22525726 |
| ebi-a-GCST90016982 | genus Coprobacter id.949 | finngen_R9_L178_PSORIASIS | Inverse variance weighted | 14 | 0.068265649 | 0.318226204 | 0.934132861 | 0.817146183 | 1.067867929 |
| ebi-a-GCST90016982 | genus Coprobacter id.949 | finngen_R9_L179_PSORIASIS | MR Egger | 14 | 0.212220322 | 0.101933757 | 0.686711082 | 0.453031573 | 1.040925484 |
| ebi-a-GCST90016982 | genus Coprobacter id.949 | finngen_R9_L180_PSORIASIS | Simple mode | 14 | 0.154271228 | 0.313641301 | 0.850689506 | 0.628713455 | 1.151037295 |
| ebi-a-GCST90016982 | genus Coprobacter id.949 | finngen_R9_L181_PSORIASIS | Weighted median | 14 | 0.079609454 | 0.332797429 | 0.925796044 | 0.792045964 | 1.082132039 |
| ebi-a-GCST90016982 | genus Coprobacter id.949 | finngen_R9_L182_PSORIASIS | Weighted mode | 14 | 0.148254949 | 0.295190411 | 0.850689506 | 0.636171069 | 1.137544082 |
| ebi-a-GCST90016983 | genus Coprococcus1 id.11301 | finngen_R9_L183_PSORIASIS | Inverse variance weighted | 13 | 0.089067137 | 0.956289684 | 0.99513011 | 0.835726916 | 1.184937229 |
| ebi-a-GCST90016983 | genus Coprococcus1 id.11301 | finngen_R9_L184_PSORIASIS | MR Egger | 13 | 0.236875821 | 0.88143852 | 1.036820726 | 0.651734785 | 1.649439685 |
| ebi-a-GCST90016983 | genus Coprococcus1 id.11301 | finngen_R9_L185_PSORIASIS | Simple mode | 13 | 0.182616918 | 0.449097199 | 1.153608078 | 0.806513477 | 1.650079799 |
| ebi-a-GCST90016983 | genus Coprococcus1 id.11301 | finngen_R9_L186_PSORIASIS | Weighted median | 13 | 0.109901888 | 0.86582403 | 1.018743079 | 0.821323588 | 1.263615798 |
| ebi-a-GCST90016983 | genus Coprococcus1 id.11301 | finngen_R9_L187_PSORIASIS | Weighted mode | 13 | 0.156708407 | 0.891728641 | 1.022027032 | 0.751743175 | 1.389489507 |
| ebi-a-GCST90016986 | genus Defluviitaleaceae UCG011 id.11287 | finngen_R9_L188_PSORIASIS | Inverse variance weighted | 10 | 0.067541318 | 0.28511968 | 0.930350229 | 0.814993487 | 1.062034927 |
| ebi-a-GCST90016986 | genus Defluviitaleaceae UCG011 id.11287 | finngen_R9_L189_PSORIASIS | MR Egger | 10 | 0.262289754 | 0.664614409 | 1.125295241 | 0.672978107 | 1.881620467 |
| ebi-a-GCST90016986 | genus Defluviitaleaceae UCG011 id.11287 | finngen_R9_L190_PSORIASIS | Simple mode | 10 | 0.127559134 | 0.825480992 | 1.029381845 | 0.801670638 | 1.321773472 |
| ebi-a-GCST90016986 | genus Defluviitaleaceae UCG011 id.11287 | finngen_R9_L191_PSORIASIS | Weighted median | 10 | 0.089178126 | 0.992815281 | 1.000803357 | 0.840308584 | 1.191951835 |
| ebi-a-GCST90016986 | genus Defluviitaleaceae UCG011 id.11287 | finngen_R9_L192_PSORIASIS | Weighted mode | 10 | 0.1157064 | 0.956125787 | 1.006566671 | 0.802326746 | 1.262797816 |
| ebi-a-GCST90016989 | genus Dorea id.1997 | finngen_R9_L193_PSORIASIS | Inverse variance weighted | 12 | 0.097960568 | 0.855484376 | 1.018001203 | 0.840161106 | 1.23348539 |
| ebi-a-GCST90016989 | genus Dorea id.1997 | finngen_R9_L194_PSORIASIS | MR Egger | 12 | 0.300319668 | 0.819544138 | 0.93207763 | 0.517386178 | 1.679149434 |
| ebi-a-GCST90016989 | genus Dorea id.1997 | finngen_R9_L195_PSORIASIS | Simple mode | 12 | 0.213278541 | 0.926678996 | 1.020284283 | 0.67169921 | 1.549771092 |
| ebi-a-GCST90016989 | genus Dorea id.1997 | finngen_R9_L196_PSORIASIS | Weighted median | 12 | 0.116769898 | 0.63176808 | 1.057556202 | 0.841214815 | 1.32953569 |
| ebi-a-GCST90016989 | genus Dorea id.1997 | finngen_R9_L197_PSORIASIS | Weighted mode | 12 | 0.175264752 | 0.626673025 | 1.091657834 | 0.774280223 | 1.539128588 |
| ebi-a-GCST90016992 | genus Enterorhabdus id.820 | finngen_R9_L198_PSORIASIS | Inverse variance weighted | 7 | 0.074947778 | 0.798106894 | 0.98101112 | 0.84698768 | 1.136241812 |
| ebi-a-GCST90016992 | genus Enterorhabdus id.820 | finngen_R9_L199_PSORIASIS | MR Egger | 7 | 0.201123595 | 0.783360998 | 0.943316997 | 0.636001002 | 1.399128231 |
| ebi-a-GCST90016992 | genus Enterorhabdus id.820 | finngen_R9_L200_PSORIASIS | Simple mode | 7 | 0.150054442 | 0.708488014 | 1.060611906 | 0.790364778 | 1.423263848 |
| ebi-a-GCST90016992 | genus Enterorhabdus id.820 | finngen_R9_L201_PSORIASIS | Weighted median | 7 | 0.097346239 | 0.929925528 | 0.991476051 | 0.819255635 | 1.199899907 |
| ebi-a-GCST90016992 | genus Enterorhabdus id.820 | finngen_R9_L202_PSORIASIS | Weighted mode | 7 | 0.125548099 | 0.87676089 | 1.020523713 | 0.797910905 | 1.305244284 |
| ebi-a-GCST90016995 | genus Escherichia Shigella id.3504 | finngen_R9_L203_PSORIASIS | Inverse variance weighted | 15 | 0.07324504 | 0.823658134 | 0.98381038 | 0.852244021 | 1.135687479 |
| ebi-a-GCST90016995 | genus Escherichia Shigella id.3504 | finngen_R9_L204_PSORIASIS | MR Egger | 15 | 0.233459081 | 0.949208019 | 0.984952996 | 0.623291385 | 1.556466892 |
| ebi-a-GCST90016995 | genus Escherichia Shigella id.3504 | finngen_R9_L205_PSORIASIS | Simple mode | 15 | 0.164934991 | 0.887016505 | 0.97641776 | 0.706708267 | 1.349059698 |
| ebi-a-GCST90016995 | genus Escherichia Shigella id.3504 | finngen_R9_L206_PSORIASIS | Weighted median | 15 | 0.091313232 | 0.834728554 | 1.019234319 | 0.85221004 | 1.218993616 |
| ebi-a-GCST90016995 | genus Escherichia Shigella id.3504 | finngen_R9_L207_PSORIASIS | Weighted mode | 15 | 0.147141728 | 0.978420424 | 1.004060003 | 0.75250623 | 1.339705174 |
| ebi-a-GCST90016996 | genus Eubacterium brachy group id.11296 | finngen_R9_L208_PSORIASIS | Inverse variance weighted | 11 | 0.044318391 | 0.689790096 | 0.982466297 | 0.900726801 | 1.071623519 |
| ebi-a-GCST90016996 | genus Eubacterium brachy group id.11296 | finngen_R9_L209_PSORIASIS | MR Egger | 11 | 0.161083147 | 0.553032341 | 1.104351109 | 0.805360691 | 1.514341816 |
| ebi-a-GCST90016996 | genus Eubacterium brachy group id.11296 | finngen_R9_L210_PSORIASIS | Simple mode | 11 | 0.090499974 | 0.677590418 | 1.039511547 | 0.870550931 | 1.241264833 |
| ebi-a-GCST90016996 | genus Eubacterium brachy group id.11296 | finngen_R9_L211_PSORIASIS | Weighted median | 11 | 0.057684245 | 0.891264218 | 1.007916881 | 0.900166608 | 1.128564902 |
| ebi-a-GCST90016996 | genus Eubacterium brachy group id.11296 | finngen_R9_L212_PSORIASIS | Weighted mode | 11 | 0.094687317 | 0.657588419 | 1.044200531 | 0.867330141 | 1.257139235 |
| ebi-a-GCST90016999 | genus Eubacterium fissicatena group id.14373 | finngen_R9_L213_PSORIASIS | Inverse variance weighted | 9 | 0.049176458 | 0.000165351 | 0.830906454 | 0.754557407 | 0.914980794 |
| ebi-a-GCST90016999 | genus Eubacterium fissicatena group id.14373 | finngen_R9_L214_PSORIASIS | MR Egger | 9 | 0.255210795 | 0.447444437 | 1.22798454 | 0.744651528 | 2.025035839 |
| ebi-a-GCST90016999 | genus Eubacterium fissicatena group id.14373 | finngen_R9_L215_PSORIASIS | Simple mode | 9 | 0.108844422 | 0.074849093 | 0.800266066 | 0.646523267 | 0.990568799 |
| ebi-a-GCST90016999 | genus Eubacterium fissicatena group id.14373 | finngen_R9_L216_PSORIASIS | Weighted median | 9 | 0.068558033 | 0.007703106 | 0.83302272 | 0.728281152 | 0.952828244 |
| ebi-a-GCST90016999 | genus Eubacterium fissicatena group id.14373 | finngen_R9_L217_PSORIASIS | Weighted mode | 9 | 0.10757311 | 0.085986022 | 0.810116382 | 0.656114046 | 1.00026597 |
| ebi-a-GCST90017000 | genus Eubacterium hallii group id.11338 | finngen_R9_L218_PSORIASIS | Inverse variance weighted | 14 | 0.084891157 | 0.693692473 | 0.967118446 | 0.818877343 | 1.142195588 |
| ebi-a-GCST90017000 | genus Eubacterium hallii group id.11338 | finngen_R9_L219_PSORIASIS | MR Egger | 14 | 0.183402117 | 0.72539447 | 0.936176235 | 0.653495465 | 1.341135462 |
| ebi-a-GCST90017000 | genus Eubacterium hallii group id.11338 | finngen_R9_L220_PSORIASIS | Simple mode | 14 | 0.161526272 | 0.646535536 | 1.078774777 | 0.786025879 | 1.480555603 |
| ebi-a-GCST90017000 | genus Eubacterium hallii group id.11338 | finngen_R9_L221_PSORIASIS | Weighted median | 14 | 0.099625835 | 0.502315898 | 1.069118013 | 0.879472824 | 1.299657357 |
| ebi-a-GCST90017000 | genus Eubacterium hallii group id.11338 | finngen_R9_L222_PSORIASIS | Weighted mode | 14 | 0.123747753 | 0.605749039 | 1.067647196 | 0.837705853 | 1.360704993 |
| ebi-a-GCST90017001 | genus Eubacterium nodatum group id.11297 | finngen_R9_L223_PSORIASIS | Inverse variance weighted | 11 | 0.040118191 | 0.993338339 | 1.000335013 | 0.924690033 | 1.082168189 |
| ebi-a-GCST90017001 | genus Eubacterium nodatum group id.11297 | finngen_R9_L224_PSORIASIS | MR Egger | 11 | 0.178799367 | 0.38570426 | 1.177046436 | 0.829080122 | 1.671054795 |
| ebi-a-GCST90017001 | genus Eubacterium nodatum group id.11297 | finngen_R9_L225_PSORIASIS | Simple mode | 11 | 0.092913819 | 0.830296576 | 0.979766377 | 0.816643842 | 1.175472224 |
| ebi-a-GCST90017001 | genus Eubacterium nodatum group id.11297 | finngen_R9_L226_PSORIASIS | Weighted median | 11 | 0.053293342 | 0.817616009 | 1.012365934 | 0.911954801 | 1.123832873 |
| ebi-a-GCST90017001 | genus Eubacterium nodatum group id.11297 | finngen_R9_L227_PSORIASIS | Weighted mode | 11 | 0.093879247 | 0.94492489 | 0.993372031 | 0.826419012 | 1.194052869 |
| ebi-a-GCST90017003 | genus Eubacterium rectale group id.14374 | finngen_R9_L228_PSORIASIS | Inverse variance weighted | 11 | 0.080104097 | 0.136018113 | 0.88743683 | 0.758492804 | 1.038301384 |
| ebi-a-GCST90017003 | genus Eubacterium rectale group id.14374 | finngen_R9_L229_PSORIASIS | MR Egger | 11 | 0.219804472 | 0.654766286 | 1.106983456 | 0.719514891 | 1.70310912 |
| ebi-a-GCST90017003 | genus Eubacterium rectale group id.14374 | finngen_R9_L230_PSORIASIS | Simple mode | 11 | 0.191013182 | 0.353001876 | 0.830225973 | 0.570955963 | 1.207229999 |
| ebi-a-GCST90017003 | genus Eubacterium rectale group id.14374 | finngen_R9_L231_PSORIASIS | Weighted median | 11 | 0.107370826 | 0.337306843 | 0.902106835 | 0.730906933 | 1.11340679 |
| ebi-a-GCST90017003 | genus Eubacterium rectale group id.14374 | finngen_R9_L232_PSORIASIS | Weighted mode | 11 | 0.156594397 | 0.395261922 | 0.870143431 | 0.640169537 | 1.182732927 |
| ebi-a-GCST90017004 | genus Eubacterium ruminantium group id.11340 | finngen_R9_L233_PSORIASIS | Inverse variance weighted | 19 | 0.045997049 | 0.382324353 | 0.960612654 | 0.87779848 | 1.051239769 |
| ebi-a-GCST90017004 | genus Eubacterium ruminantium group id.11340 | finngen_R9_L234_PSORIASIS | MR Egger | 19 | 0.153081527 | 0.617619602 | 1.08095625 | 0.800760221 | 1.459196378 |
| ebi-a-GCST90017004 | genus Eubacterium ruminantium group id.11340 | finngen_R9_L235_PSORIASIS | Simple mode | 19 | 0.110967676 | 0.159989109 | 0.849895381 | 0.68376659 | 1.056387032 |
| ebi-a-GCST90017004 | genus Eubacterium ruminantium group id.11340 | finngen_R9_L236_PSORIASIS | Weighted median | 19 | 0.063427451 | 0.961786964 | 1.003043511 | 0.885786865 | 1.135822086 |
| ebi-a-GCST90017004 | genus Eubacterium ruminantium group id.11340 | finngen_R9_L237_PSORIASIS | Weighted mode | 19 | 0.105655014 | 0.817677897 | 1.025024045 | 0.833294835 | 1.260867402 |
| ebi-a-GCST90017007 | genus Faecalibacterium id.2057 | finngen_R9_L238_PSORIASIS | Inverse variance weighted | 13 | 0.066425613 | 0.080041533 | 1.123304054 | 0.986176622 | 1.279498995 |
| ebi-a-GCST90017007 | genus Faecalibacterium id.2057 | finngen_R9_L239_PSORIASIS | MR Egger | 13 | 0.135785451 | 0.78862989 | 0.963385496 | 0.738273354 | 1.257138169 |
| ebi-a-GCST90017007 | genus Faecalibacterium id.2057 | finngen_R9_L240_PSORIASIS | Simple mode | 13 | 0.142366728 | 0.948548193 | 1.009425089 | 0.763640739 | 1.334317249 |
| ebi-a-GCST90017007 | genus Faecalibacterium id.2057 | finngen_R9_L241_PSORIASIS | Weighted median | 13 | 0.095609309 | 0.221123881 | 1.124099535 | 0.932009805 | 1.355779476 |
| ebi-a-GCST90017007 | genus Faecalibacterium id.2057 | finngen_R9_L242_PSORIASIS | Weighted mode | 13 | 0.108748368 | 0.328606835 | 1.11710811 | 0.902665253 | 1.382495367 |
| ebi-a-GCST90017008 | genus Family XIII AD3011 group id.11293 | finngen_R9_L243_PSORIASIS | Inverse variance weighted | 15 | 0.097753151 | 0.079994715 | 0.842705207 | 0.695771308 | 1.0206688 |
| ebi-a-GCST90017008 | genus Family XIII AD3011 group id.11293 | finngen_R9_L244_PSORIASIS | MR Egger | 15 | 0.466569314 | 0.393235585 | 1.509854915 | 0.605039042 | 3.767792996 |
| ebi-a-GCST90017008 | genus Family XIII AD3011 group id.11293 | finngen_R9_L245_PSORIASIS | Simple mode | 15 | 0.20237878 | 0.721645301 | 1.07634305 | 0.723906399 | 1.600364857 |
| ebi-a-GCST90017008 | genus Family XIII AD3011 group id.11293 | finngen_R9_L246_PSORIASIS | Weighted median | 15 | 0.106783528 | 0.599837995 | 0.94551813 | 0.766962071 | 1.165643737 |
| ebi-a-GCST90017008 | genus Family XIII AD3011 group id.11293 | finngen_R9_L247_PSORIASIS | Weighted mode | 15 | 0.189213815 | 0.72128627 | 1.071303875 | 0.739350933 | 1.552296672 |
| ebi-a-GCST90017010 | genus Flavonifractor id.2059 | finngen_R9_L248_PSORIASIS | Inverse variance weighted | 8 | 0.078846046 | 0.95767352 | 1.004193386 | 0.860403657 | 1.1720131 |
| ebi-a-GCST90017010 | genus Flavonifractor id.2059 | finngen_R9_L249_PSORIASIS | MR Egger | 8 | 0.285108471 | 0.150675016 | 0.625284338 | 0.357592433 | 1.093369063 |
| ebi-a-GCST90017010 | genus Flavonifractor id.2059 | finngen_R9_L250_PSORIASIS | Simple mode | 8 | 0.16947569 | 0.45748395 | 1.142543928 | 0.819619509 | 1.592698336 |
| ebi-a-GCST90017010 | genus Flavonifractor id.2059 | finngen_R9_L251_PSORIASIS | Weighted median | 8 | 0.109068015 | 0.42958618 | 1.089972889 | 0.880187359 | 1.349759102 |
| ebi-a-GCST90017010 | genus Flavonifractor id.2059 | finngen_R9_L252_PSORIASIS | Weighted mode | 8 | 0.178129178 | 0.478778263 | 1.142543928 | 0.805835303 | 1.619942218 |
| ebi-a-GCST90017013 | genus Haemophilus id.3698 | finngen_R9_L253_PSORIASIS | Inverse variance weighted | 13 | 0.053659592 | 0.776887018 | 0.984909074 | 0.886584577 | 1.094138009 |
| ebi-a-GCST90017013 | genus Haemophilus id.3698 | finngen_R9_L254_PSORIASIS | MR Egger | 13 | 0.131216311 | 0.624884602 | 0.936129449 | 0.723839583 | 1.210680331 |
| ebi-a-GCST90017013 | genus Haemophilus id.3698 | finngen_R9_L255_PSORIASIS | Simple mode | 13 | 0.126892614 | 0.300333009 | 0.871662989 | 0.679728427 | 1.117794015 |
| ebi-a-GCST90017013 | genus Haemophilus id.3698 | finngen_R9_L256_PSORIASIS | Weighted median | 13 | 0.074708711 | 0.485844852 | 0.94926531 | 0.81996303 | 1.098957632 |
| ebi-a-GCST90017013 | genus Haemophilus id.3698 | finngen_R9_L257_PSORIASIS | Weighted mode | 13 | 0.122623669 | 0.309200173 | 0.877911124 | 0.690352939 | 1.11642596 |
| ebi-a-GCST90017014 | genus Holdemanella id.11393 | finngen_R9_L258_PSORIASIS | Inverse variance weighted | 14 | 0.052882346 | 0.420278503 | 0.958275561 | 0.863925018 | 1.062930267 |
| ebi-a-GCST90017014 | genus Holdemanella id.11393 | finngen_R9_L259_PSORIASIS | MR Egger | 14 | 0.15924354 | 0.107354476 | 0.757949669 | 0.554740061 | 1.035598005 |
| ebi-a-GCST90017014 | genus Holdemanella id.11393 | finngen_R9_L260_PSORIASIS | Simple mode | 14 | 0.115635704 | 0.484127629 | 0.920088587 | 0.733497343 | 1.154145978 |
| ebi-a-GCST90017014 | genus Holdemanella id.11393 | finngen_R9_L261_PSORIASIS | Weighted median | 14 | 0.073074162 | 0.390220934 | 0.939143917 | 0.81382339 | 1.083762531 |
| ebi-a-GCST90017014 | genus Holdemanella id.11393 | finngen_R9_L262_PSORIASIS | Weighted mode | 14 | 0.106116005 | 0.502161828 | 0.929369546 | 0.754850019 | 1.144237574 |
| ebi-a-GCST90017017 | genus Hungatella id.11306 | finngen_R9_L263_PSORIASIS | Inverse variance weighted | 5 | 0.087019008 | 0.44037902 | 1.069444982 | 0.90175046 | 1.26832491 |
| ebi-a-GCST90017017 | genus Hungatella id.11306 | finngen_R9_L264_PSORIASIS | MR Egger | 5 | 0.579026352 | 0.688047133 | 0.773944714 | 0.248789907 | 2.407615436 |
| ebi-a-GCST90017017 | genus Hungatella id.11306 | finngen_R9_L265_PSORIASIS | Simple mode | 5 | 0.146686877 | 0.719076385 | 0.944937439 | 0.708827675 | 1.25969512 |
| ebi-a-GCST90017017 | genus Hungatella id.11306 | finngen_R9_L266_PSORIASIS | Weighted median | 5 | 0.099056365 | 0.605114635 | 1.05255236 | 0.866812627 | 1.278092215 |
| ebi-a-GCST90017017 | genus Hungatella id.11306 | finngen_R9_L267_PSORIASIS | Weighted mode | 5 | 0.142918464 | 0.834456361 | 0.968630931 | 0.731987526 | 1.28177851 |
| ebi-a-GCST90017020 | genus Lachnoclostridium id.11308 | finngen_R9_L268_PSORIASIS | Inverse variance weighted | 13 | 0.081084192 | 0.357763223 | 0.928144443 | 0.791763198 | 1.088017364 |
| ebi-a-GCST90017020 | genus Lachnoclostridium id.11308 | finngen_R9_L269_PSORIASIS | MR Egger | 13 | 0.275720458 | 0.538490672 | 0.839416585 | 0.488967005 | 1.441038347 |
| ebi-a-GCST90017020 | genus Lachnoclostridium id.11308 | finngen_R9_L270_PSORIASIS | Simple mode | 13 | 0.199664989 | 0.867952593 | 0.966654168 | 0.653601226 | 1.429648911 |
| ebi-a-GCST90017020 | genus Lachnoclostridium id.11308 | finngen_R9_L271_PSORIASIS | Weighted median | 13 | 0.109261059 | 0.424226824 | 0.916391533 | 0.739735016 | 1.135235487 |
| ebi-a-GCST90017020 | genus Lachnoclostridium id.11308 | finngen_R9_L272_PSORIASIS | Weighted mode | 13 | 0.165111013 | 0.611516637 | 0.917499398 | 0.663835474 | 1.268093043 |
| ebi-a-GCST90017023 | genus Lachnospiraceae ND3007 group id.11317 | finngen_R9_L273_PSORIASIS | Inverse variance weighted | 3 | 0.168331407 | 0.67680498 | 1.072685234 | 0.771233198 | 1.491965871 |
| ebi-a-GCST90017023 | genus Lachnospiraceae ND3007 group id.11317 | finngen_R9_L274_PSORIASIS | MR Egger | 3 | 2.92176906 | 0.744183966 | 3.461245502 | 0.011276449 | 1062.410704 |
| ebi-a-GCST90017023 | genus Lachnospiraceae ND3007 group id.11317 | finngen_R9_L275_PSORIASIS | Simple mode | 3 | 0.249784472 | 0.495602655 | 1.22918373 | 0.753348569 | 2.005569141 |
| ebi-a-GCST90017023 | genus Lachnospiraceae ND3007 group id.11317 | finngen_R9_L276_PSORIASIS | Weighted median | 3 | 0.217555017 | 0.425737748 | 1.189203751 | 0.776371709 | 1.82155731 |
| ebi-a-GCST90017023 | genus Lachnospiraceae ND3007 group id.11317 | finngen_R9_L277_PSORIASIS | Weighted mode | 3 | 0.256164217 | 0.50673394 | 1.228048504 | 0.743300007 | 2.028929251 |
| ebi-a-GCST90017026 | genus Lachnospiraceae UCG004 id.11324 | finngen_R9_L278_PSORIASIS | Inverse variance weighted | 13 | 0.078436664 | 0.559922585 | 1.046786675 | 0.897617994 | 1.220744628 |
| ebi-a-GCST90017026 | genus Lachnospiraceae UCG004 id.11324 | finngen_R9_L279_PSORIASIS | MR Egger | 13 | 0.331499883 | 0.666226668 | 1.158253698 | 0.604818823 | 2.218104956 |
| ebi-a-GCST90017026 | genus Lachnospiraceae UCG004 id.11324 | finngen_R9_L280_PSORIASIS | Simple mode | 13 | 0.17857758 | 0.933285743 | 0.98485027 | 0.694003889 | 1.397585909 |
| ebi-a-GCST90017026 | genus Lachnospiraceae UCG004 id.11324 | finngen_R9_L281_PSORIASIS | Weighted median | 13 | 0.103847289 | 0.863619425 | 1.017997749 | 0.830520239 | 1.2477955 |
| ebi-a-GCST90017026 | genus Lachnospiraceae UCG004 id.11324 | finngen_R9_L282_PSORIASIS | Weighted mode | 13 | 0.162723033 | 0.94563321 | 0.988733164 | 0.718731136 | 1.360165464 |
| ebi-a-GCST90017027 | genus Lachnospiraceae UCG008 id.11328 | finngen_R9_L283_PSORIASIS | Inverse variance weighted | 11 | 0.054230811 | 0.720857097 | 0.980809132 | 0.881906011 | 1.090803942 |
| ebi-a-GCST90017027 | genus Lachnospiraceae UCG008 id.11328 | finngen_R9_L284_PSORIASIS | MR Egger | 11 | 0.279091473 | 0.156071251 | 0.649219752 | 0.375685356 | 1.121913004 |
| ebi-a-GCST90017027 | genus Lachnospiraceae UCG008 id.11328 | finngen_R9_L285_PSORIASIS | Simple mode | 11 | 0.119658874 | 0.579018482 | 1.071025176 | 0.847118155 | 1.354114441 |
| ebi-a-GCST90017027 | genus Lachnospiraceae UCG008 id.11328 | finngen_R9_L286_PSORIASIS | Weighted median | 11 | 0.069181724 | 0.655200706 | 1.031375174 | 0.900591825 | 1.181150793 |
| ebi-a-GCST90017027 | genus Lachnospiraceae UCG008 id.11328 | finngen_R9_L287_PSORIASIS | Weighted mode | 11 | 0.108608557 | 0.541705723 | 1.071025176 | 0.865665699 | 1.325101513 |
| ebi-a-GCST90017030 | genus Lactobacillus id.1837 | finngen_R9_L288_PSORIASIS | Inverse variance weighted | 8 | 0.065107857 | 0.712285642 | 0.976274965 | 0.859312691 | 1.10915714 |
| ebi-a-GCST90017030 | genus Lactobacillus id.1837 | finngen_R9_L289_PSORIASIS | MR Egger | 8 | 0.178346753 | 0.631636117 | 0.913888731 | 0.644290164 | 1.296298873 |
| ebi-a-GCST90017030 | genus Lactobacillus id.1837 | finngen_R9_L290_PSORIASIS | Simple mode | 8 | 0.136387992 | 0.556384366 | 1.087883665 | 0.832696335 | 1.421275462 |
| ebi-a-GCST90017030 | genus Lactobacillus id.1837 | finngen_R9_L291_PSORIASIS | Weighted median | 8 | 0.081628959 | 0.806048374 | 0.980157391 | 0.83524108 | 1.150217027 |
| ebi-a-GCST90017030 | genus Lactobacillus id.1837 | finngen_R9_L292_PSORIASIS | Weighted mode | 8 | 0.109704482 | 0.787011521 | 0.969668881 | 0.78206188 | 1.202280488 |
| ebi-a-GCST90017032 | genus Marvinbryantia id.2005 | finngen_R9_L293_PSORIASIS | Inverse variance weighted | 10 | 0.103562128 | 0.151871891 | 1.159976141 | 0.946880545 | 1.421028929 |
| ebi-a-GCST90017032 | genus Marvinbryantia id.2005 | finngen_R9_L294_PSORIASIS | MR Egger | 10 | 0.415156638 | 0.296929618 | 1.589152188 | 0.704331359 | 3.585534907 |
| ebi-a-GCST90017032 | genus Marvinbryantia id.2005 | finngen_R9_L295_PSORIASIS | Simple mode | 10 | 0.204170075 | 0.990558011 | 1.002487174 | 0.671870771 | 1.495794396 |
| ebi-a-GCST90017032 | genus Marvinbryantia id.2005 | finngen_R9_L296_PSORIASIS | Weighted median | 10 | 0.115190225 | 0.80383066 | 1.029025666 | 0.82105889 | 1.289668542 |
| ebi-a-GCST90017032 | genus Marvinbryantia id.2005 | finngen_R9_L297_PSORIASIS | Weighted mode | 10 | 0.173811402 | 0.95617522 | 0.990227109 | 0.704341957 | 1.392150102 |
| ebi-a-GCST90017033 | genus Methanobrevibacter id.123 | finngen_R9_L298_PSORIASIS | Inverse variance weighted | 8 | 0.052767386 | 0.076361441 | 0.91072496 | 0.821241209 | 1.009959004 |
| ebi-a-GCST90017033 | genus Methanobrevibacter id.123 | finngen_R9_L299_PSORIASIS | MR Egger | 8 | 0.225565991 | 0.862058284 | 0.959917328 | 0.616919053 | 1.493617797 |
| ebi-a-GCST90017033 | genus Methanobrevibacter id.123 | finngen_R9_L300_PSORIASIS | Simple mode | 8 | 0.104338671 | 0.377068777 | 0.906291932 | 0.738674734 | 1.111944175 |
| ebi-a-GCST90017033 | genus Methanobrevibacter id.123 | finngen_R9_L301_PSORIASIS | Weighted median | 8 | 0.070066229 | 0.166722193 | 0.907657208 | 0.791189077 | 1.041270198 |
| ebi-a-GCST90017033 | genus Methanobrevibacter id.123 | finngen_R9_L302_PSORIASIS | Weighted mode | 8 | 0.09857384 | 0.372488246 | 0.910366551 | 0.750427163 | 1.104394002 |
| ebi-a-GCST90017036 | genus Oscillibacter id.2063 | finngen_R9_L303_PSORIASIS | Inverse variance weighted | 16 | 0.050422817 | 0.41510869 | 1.041947326 | 0.943897849 | 1.150181909 |
| ebi-a-GCST90017036 | genus Oscillibacter id.2063 | finngen_R9_L304_PSORIASIS | MR Egger | 16 | 0.178783304 | 0.630397257 | 0.915808852 | 0.645091645 | 1.300134424 |
| ebi-a-GCST90017036 | genus Oscillibacter id.2063 | finngen_R9_L305_PSORIASIS | Simple mode | 16 | 0.122640346 | 0.887987564 | 1.01772536 | 0.800270903 | 1.29426786 |
| ebi-a-GCST90017036 | genus Oscillibacter id.2063 | finngen_R9_L306_PSORIASIS | Weighted median | 16 | 0.074412877 | 0.538677129 | 1.046812808 | 0.904747743 | 1.211185178 |
| ebi-a-GCST90017036 | genus Oscillibacter id.2063 | finngen_R9_L307_PSORIASIS | Weighted mode | 16 | 0.130301893 | 0.833561673 | 1.028253779 | 0.79649876 | 1.327441909 |
| ebi-a-GCST90017037 | genus Oscillospira id.2064 | finngen_R9_L308_PSORIASIS | Inverse variance weighted | 9 | 0.110977445 | 0.824595924 | 1.024901783 | 0.824548755 | 1.273937604 |
| ebi-a-GCST90017037 | genus Oscillospira id.2064 | finngen_R9_L309_PSORIASIS | MR Egger | 9 | 0.387741689 | 0.05182615 | 2.477899351 | 1.158860803 | 5.298293963 |
| ebi-a-GCST90017037 | genus Oscillospira id.2064 | finngen_R9_L310_PSORIASIS | Simple mode | 9 | 0.214375346 | 0.30317897 | 1.266035831 | 0.831698693 | 1.927196398 |
| ebi-a-GCST90017037 | genus Oscillospira id.2064 | finngen_R9_L311_PSORIASIS | Weighted median | 9 | 0.115929806 | 0.565388344 | 1.068913812 | 0.851650186 | 1.341603345 |
| ebi-a-GCST90017037 | genus Oscillospira id.2064 | finngen_R9_L312_PSORIASIS | Weighted mode | 9 | 0.182598526 | 0.272445541 | 1.240109104 | 0.867019555 | 1.773743835 |
| ebi-a-GCST90017042 | genus Peptococcus id.2037 | finngen_R9_L313_PSORIASIS | Inverse variance weighted | 16 | 0.047321092 | 0.42459844 | 0.962920505 | 0.877626865 | 1.056503552 |
| ebi-a-GCST90017042 | genus Peptococcus id.2037 | finngen_R9_L314_PSORIASIS | MR Egger | 16 | 0.180285101 | 0.654793462 | 0.920938926 | 0.646798574 | 1.311271452 |
| ebi-a-GCST90017042 | genus Peptococcus id.2037 | finngen_R9_L315_PSORIASIS | Simple mode | 16 | 0.113628577 | 0.333143801 | 0.892583719 | 0.714375191 | 1.115248269 |
| ebi-a-GCST90017042 | genus Peptococcus id.2037 | finngen_R9_L316_PSORIASIS | Weighted median | 16 | 0.057288964 | 0.114689525 | 0.91359209 | 0.816557891 | 1.022157176 |
| ebi-a-GCST90017042 | genus Peptococcus id.2037 | finngen_R9_L317_PSORIASIS | Weighted mode | 16 | 0.098647403 | 0.302826626 | 0.900089694 | 0.741848845 | 1.092084274 |
| ebi-a-GCST90017043 | genus Phascolarctobacterium id.2168 | finngen_R9_L318_PSORIASIS | Inverse variance weighted | 9 | 0.09846064 | 0.328520723 | 0.908277184 | 0.748871005 | 1.101614881 |
| ebi-a-GCST90017043 | genus Phascolarctobacterium id.2168 | finngen_R9_L319_PSORIASIS | MR Egger | 9 | 0.415836199 | 0.920049097 | 0.95765545 | 0.423879454 | 2.163596166 |
| ebi-a-GCST90017043 | genus Phascolarctobacterium id.2168 | finngen_R9_L320_PSORIASIS | Simple mode | 9 | 0.182668105 | 0.861660611 | 0.967661907 | 0.676446438 | 1.384247906 |
| ebi-a-GCST90017043 | genus Phascolarctobacterium id.2168 | finngen_R9_L321_PSORIASIS | Weighted median | 9 | 0.107334296 | 0.583133566 | 0.942794924 | 0.763928017 | 1.163541916 |
| ebi-a-GCST90017043 | genus Phascolarctobacterium id.2168 | finngen_R9_L322_PSORIASIS | Weighted mode | 9 | 0.164968637 | 0.847026652 | 0.967661907 | 0.7003248 | 1.337050417 |
| ebi-a-GCST90017046 | genus Rikenellaceae RC9 gut group id.11191 | finngen_R9_L323_PSORIASIS | Inverse variance weighted | 12 | 0.038266912 | 0.69270019 | 0.984990946 | 0.913816059 | 1.061709471 |
| ebi-a-GCST90017046 | genus Rikenellaceae RC9 gut group id.11191 | finngen_R9_L324_PSORIASIS | MR Egger | 12 | 0.245509057 | 0.859114773 | 1.045731017 | 0.646306407 | 1.692004516 |
| ebi-a-GCST90017046 | genus Rikenellaceae RC9 gut group id.11191 | finngen_R9_L325_PSORIASIS | Simple mode | 12 | 0.088430093 | 0.53352157 | 1.05848973 | 0.890048011 | 1.25880907 |
| ebi-a-GCST90017046 | genus Rikenellaceae RC9 gut group id.11191 | finngen_R9_L326_PSORIASIS | Weighted median | 12 | 0.051704023 | 0.669811411 | 1.022291779 | 0.923769281 | 1.13132197 |
| ebi-a-GCST90017046 | genus Rikenellaceae RC9 gut group id.11191 | finngen_R9_L327_PSORIASIS | Weighted mode | 12 | 0.08972986 | 0.539360904 | 1.05848973 | 0.88778346 | 1.26202003 |
| ebi-a-GCST90017049 | genus Ruminiclostridium5 id.11355 | finngen_R9_L328_PSORIASIS | Inverse variance weighted | 14 | 0.069460904 | 0.172368017 | 0.909565076 | 0.79379336 | 1.042221653 |
| ebi-a-GCST90017049 | genus Ruminiclostridium5 id.11355 | finngen_R9_L329_PSORIASIS | MR Egger | 14 | 0.175515777 | 0.020136047 | 0.625056133 | 0.443115534 | 0.881700459 |
| ebi-a-GCST90017049 | genus Ruminiclostridium5 id.11355 | finngen_R9_L330_PSORIASIS | Simple mode | 14 | 0.194376807 | 0.704468461 | 1.078284666 | 0.73667605 | 1.578302731 |
| ebi-a-GCST90017049 | genus Ruminiclostridium5 id.11355 | finngen_R9_L331_PSORIASIS | Weighted median | 14 | 0.103048001 | 0.943809264 | 0.992763185 | 0.811202787 | 1.214959757 |
| ebi-a-GCST90017049 | genus Ruminiclostridium5 id.11355 | finngen_R9_L332_PSORIASIS | Weighted mode | 14 | 0.160104167 | 0.692608466 | 1.066860682 | 0.779514668 | 1.460128669 |
| ebi-a-GCST90017052 | genus Ruminococcaceae NK4A214 group id.11358 | finngen_R9_L333_PSORIASIS | Inverse variance weighted | 16 | 0.068401 | 0.118137901 | 0.898628145 | 0.78587941 | 1.027552743 |
| ebi-a-GCST90017052 | genus Ruminococcaceae NK4A214 group id.11358 | finngen_R9_L334_PSORIASIS | MR Egger | 16 | 0.200953507 | 0.190579918 | 0.758497369 | 0.511562853 | 1.124628687 |
| ebi-a-GCST90017052 | genus Ruminococcaceae NK4A214 group id.11358 | finngen_R9_L335_PSORIASIS | Simple mode | 16 | 0.163978473 | 0.411672607 | 0.87068486 | 0.63136383 | 1.200721501 |
| ebi-a-GCST90017052 | genus Ruminococcaceae NK4A214 group id.11358 | finngen_R9_L336_PSORIASIS | Weighted median | 16 | 0.096014917 | 0.316241594 | 0.908257613 | 0.75245321 | 1.096323173 |
| ebi-a-GCST90017052 | genus Ruminococcaceae NK4A214 group id.11358 | finngen_R9_L337_PSORIASIS | Weighted mode | 16 | 0.145651995 | 0.458560718 | 0.895104859 | 0.672809994 | 1.190845435 |
| ebi-a-GCST90017053 | genus Ruminococcaceae UCG002 id.11360 | finngen_R9_L338_PSORIASIS | Inverse variance weighted | 22 | 0.056647825 | 0.167046613 | 1.081418361 | 0.967774415 | 1.208407303 |
| ebi-a-GCST90017053 | genus Ruminococcaceae UCG002 id.11360 | finngen_R9_L339_PSORIASIS | MR Egger | 22 | 0.151156943 | 0.035805057 | 1.405263058 | 1.044937324 | 1.889839913 |
| ebi-a-GCST90017053 | genus Ruminococcaceae UCG002 id.11360 | finngen_R9_L340_PSORIASIS | Simple mode | 22 | 0.145860442 | 0.656814766 | 0.936373017 | 0.703541913 | 1.246257558 |
| ebi-a-GCST90017053 | genus Ruminococcaceae UCG002 id.11360 | finngen_R9_L341_PSORIASIS | Weighted median | 22 | 0.0839487 | 0.254300212 | 1.100427472 | 0.933475336 | 1.29723901 |
| ebi-a-GCST90017053 | genus Ruminococcaceae UCG002 id.11360 | finngen_R9_L342_PSORIASIS | Weighted mode | 22 | 0.12444887 | 0.401647107 | 1.112412582 | 0.871631417 | 1.419707606 |
| ebi-a-GCST90017055 | genus Ruminococcaceae UCG004 id.11362 | finngen_R9_L343_PSORIASIS | Inverse variance weighted | 12 | 0.063639951 | 0.130079454 | 0.90815752 | 0.801659168 | 1.028803904 |
| ebi-a-GCST90017055 | genus Ruminococcaceae UCG004 id.11362 | finngen_R9_L344_PSORIASIS | MR Egger | 12 | 0.365122922 | 0.840063835 | 0.927160479 | 0.453268987 | 1.896504235 |
| ebi-a-GCST90017055 | genus Ruminococcaceae UCG004 id.11362 | finngen_R9_L345_PSORIASIS | Simple mode | 12 | 0.158370084 | 0.129683796 | 0.77150776 | 0.565630685 | 1.052319542 |
| ebi-a-GCST90017055 | genus Ruminococcaceae UCG004 id.11362 | finngen_R9_L346_PSORIASIS | Weighted median | 12 | 0.087331766 | 0.268064701 | 0.907807814 | 0.76498975 | 1.077288979 |
| ebi-a-GCST90017055 | genus Ruminococcaceae UCG004 id.11362 | finngen_R9_L347_PSORIASIS | Weighted mode | 12 | 0.154325844 | 0.154500369 | 0.78981046 | 0.583657501 | 1.068778458 |
| ebi-a-GCST90017056 | genus Ruminococcaceae UCG005 id.11363 | finngen_R9_L348_PSORIASIS | Inverse variance weighted | 17 | 0.062549023 | 0.5030164 | 0.958973072 | 0.848327623 | 1.084049755 |
| ebi-a-GCST90017056 | genus Ruminococcaceae UCG005 id.11363 | finngen_R9_L349_PSORIASIS | MR Egger | 17 | 0.173034422 | 0.865420597 | 1.030282167 | 0.73394972 | 1.446258939 |
| ebi-a-GCST90017056 | genus Ruminococcaceae UCG005 id.11363 | finngen_R9_L350_PSORIASIS | Simple mode | 17 | 0.167028508 | 0.248639402 | 0.818748625 | 0.590164453 | 1.135868668 |
| ebi-a-GCST90017056 | genus Ruminococcaceae UCG005 id.11363 | finngen_R9_L351_PSORIASIS | Weighted median | 17 | 0.092298534 | 0.335362893 | 0.914921095 | 0.763514946 | 1.09635131 |
| ebi-a-GCST90017056 | genus Ruminococcaceae UCG005 id.11363 | finngen_R9_L352_PSORIASIS | Weighted mode | 17 | 0.133322096 | 0.227862564 | 0.846044846 | 0.651489356 | 1.098700807 |
| ebi-a-GCST90017059 | genus Ruminococcaceae UCG011 id.11368 | finngen_R9_L353_PSORIASIS | Inverse variance weighted | 8 | 0.048535316 | 0.091570853 | 1.085333466 | 0.986845352 | 1.193650788 |
| ebi-a-GCST90017059 | genus Ruminococcaceae UCG011 id.11368 | finngen_R9_L354_PSORIASIS | MR Egger | 8 | 0.235454849 | 0.440919369 | 0.823447755 | 0.519054351 | 1.30634914 |
| ebi-a-GCST90017059 | genus Ruminococcaceae UCG011 id.11368 | finngen_R9_L355_PSORIASIS | Simple mode | 8 | 0.10111063 | 0.854188843 | 1.019466797 | 0.836191946 | 1.242911457 |
| ebi-a-GCST90017059 | genus Ruminococcaceae UCG011 id.11368 | finngen_R9_L356_PSORIASIS | Weighted median | 8 | 0.06274784 | 0.302879831 | 1.066783107 | 0.943330964 | 1.206391226 |
| ebi-a-GCST90017059 | genus Ruminococcaceae UCG011 id.11368 | finngen_R9_L357_PSORIASIS | Weighted mode | 8 | 0.085901383 | 0.531904918 | 1.058101348 | 0.89414208 | 1.252125906 |
| ebi-a-GCST90017062 | genus Ruminococcus1 id.11373 | finngen_R9_L358_PSORIASIS | Inverse variance weighted | 13 | 0.072070205 | 0.701583633 | 0.972761718 | 0.844615552 | 1.120350386 |
| ebi-a-GCST90017062 | genus Ruminococcus1 id.11373 | finngen_R9_L359_PSORIASIS | MR Egger | 13 | 0.198807013 | 0.895917175 | 0.973735232 | 0.659497173 | 1.437701844 |
| ebi-a-GCST90017062 | genus Ruminococcus1 id.11373 | finngen_R9_L360_PSORIASIS | Simple mode | 13 | 0.169065849 | 0.906950758 | 1.020387879 | 0.732577432 | 1.421271498 |
| ebi-a-GCST90017062 | genus Ruminococcus1 id.11373 | finngen_R9_L361_PSORIASIS | Weighted median | 13 | 0.09449677 | 0.87109453 | 0.984783129 | 0.818282617 | 1.185162426 |
| ebi-a-GCST90017062 | genus Ruminococcus1 id.11373 | finngen_R9_L362_PSORIASIS | Weighted mode | 13 | 0.155472579 | 0.995325724 | 1.000930401 | 0.738011194 | 1.357515544 |
| ebi-a-GCST90017065 | genus Ruminococcus gnavus group id.14376 | finngen_R9_L363_PSORIASIS | Inverse variance weighted | 11 | 0.084099654 | 0.761372289 | 1.02586824 | 0.869970475 | 1.209702715 |
| ebi-a-GCST90017065 | genus Ruminococcus gnavus group id.14376 | finngen_R9_L364_PSORIASIS | MR Egger | 11 | 0.349523505 | 0.082602994 | 0.50533841 | 0.254719325 | 1.00254234 |
| ebi-a-GCST90017065 | genus Ruminococcus gnavus group id.14376 | finngen_R9_L365_PSORIASIS | Simple mode | 11 | 0.186322785 | 0.292670643 | 1.229930124 | 0.853648895 | 1.772072944 |
| ebi-a-GCST90017065 | genus Ruminococcus gnavus group id.14376 | finngen_R9_L366_PSORIASIS | Weighted median | 11 | 0.086131223 | 0.529432976 | 1.055659298 | 0.891676662 | 1.249798948 |
| ebi-a-GCST90017065 | genus Ruminococcus gnavus group id.14376 | finngen_R9_L367_PSORIASIS | Weighted mode | 11 | 0.1605987 | 0.351304528 | 1.170006917 | 0.854051493 | 1.602849708 |
| ebi-a-GCST90017066 | genus Ruminococcus torques group id.14377 | finngen_R9_L368_PSORIASIS | Inverse variance weighted | 11 | 0.094261721 | 0.531132326 | 1.060811397 | 0.881862712 | 1.276072572 |
| ebi-a-GCST90017066 | genus Ruminococcus torques group id.14377 | finngen_R9_L369_PSORIASIS | MR Egger | 11 | 0.338558177 | 0.785392599 | 0.909386668 | 0.468340855 | 1.765774014 |
| ebi-a-GCST90017066 | genus Ruminococcus torques group id.14377 | finngen_R9_L370_PSORIASIS | Simple mode | 11 | 0.228308041 | 0.446184775 | 1.198472838 | 0.766105303 | 1.874856028 |
| ebi-a-GCST90017066 | genus Ruminococcus torques group id.14377 | finngen_R9_L371_PSORIASIS | Weighted median | 11 | 0.126950754 | 0.275889434 | 1.148349308 | 0.895388164 | 1.472775927 |
| ebi-a-GCST90017066 | genus Ruminococcus torques group id.14377 | finngen_R9_L372_PSORIASIS | Weighted mode | 11 | 0.214422361 | 0.364014703 | 1.226199322 | 0.805454614 | 1.866728121 |
| ebi-a-GCST90017069 | genus Slackia id.825 | finngen_R9_L373_PSORIASIS | Inverse variance weighted | 9 | 0.078135224 | 0.887448378 | 1.011120138 | 0.867546405 | 1.178454463 |
| ebi-a-GCST90017069 | genus Slackia id.825 | finngen_R9_L374_PSORIASIS | MR Egger | 9 | 0.38586659 | 0.685398831 | 1.177028888 | 0.552498177 | 2.50751416 |
| ebi-a-GCST90017069 | genus Slackia id.825 | finngen_R9_L375_PSORIASIS | Simple mode | 9 | 0.133679161 | 0.655422176 | 1.063906249 | 0.818678454 | 1.382589832 |
| ebi-a-GCST90017069 | genus Slackia id.825 | finngen_R9_L376_PSORIASIS | Weighted median | 9 | 0.086076035 | 0.575349267 | 1.049400457 | 0.886485933 | 1.242254702 |
| ebi-a-GCST90017069 | genus Slackia id.825 | finngen_R9_L377_PSORIASIS | Weighted mode | 9 | 0.126065827 | 0.691400936 | 1.053265706 | 0.822675466 | 1.348488795 |
| ebi-a-GCST90017071 | genus Subdoligranulum id.2070 | finngen_R9_L378_PSORIASIS | Inverse variance weighted | 12 | 0.083689007 | 0.286290398 | 1.093339915 | 0.92793527 | 1.288227971 |
| ebi-a-GCST90017071 | genus Subdoligranulum id.2070 | finngen_R9_L379_PSORIASIS | MR Egger | 12 | 0.232219824 | 0.85959465 | 0.958726052 | 0.608170044 | 1.511346461 |
| ebi-a-GCST90017071 | genus Subdoligranulum id.2070 | finngen_R9_L380_PSORIASIS | Simple mode | 12 | 0.161138825 | 0.905966933 | 1.019669177 | 0.74352428 | 1.398374282 |
| ebi-a-GCST90017071 | genus Subdoligranulum id.2070 | finngen_R9_L381_PSORIASIS | Weighted median | 12 | 0.106712744 | 0.886889398 | 1.015294747 | 0.82367601 | 1.251491375 |
| ebi-a-GCST90017071 | genus Subdoligranulum id.2070 | finngen_R9_L382_PSORIASIS | Weighted mode | 12 | 0.144886062 | 0.895484259 | 1.019669177 | 0.767590844 | 1.354530528 |
| ebi-a-GCST90017072 | genus Sutterella id.2896 | finngen_R9_L383_PSORIASIS | Inverse variance weighted | 12 | 0.07425882 | 0.619462015 | 1.037566318 | 0.897026929 | 1.200124355 |
| ebi-a-GCST90017072 | genus Sutterella id.2896 | finngen_R9_L384_PSORIASIS | MR Egger | 12 | 0.321099972 | 0.677376021 | 1.147503467 | 0.61154468 | 2.153177438 |
| ebi-a-GCST90017072 | genus Sutterella id.2896 | finngen_R9_L385_PSORIASIS | Simple mode | 12 | 0.147443088 | 0.712799492 | 1.057277807 | 0.791923126 | 1.411546556 |
| ebi-a-GCST90017072 | genus Sutterella id.2896 | finngen_R9_L386_PSORIASIS | Weighted median | 12 | 0.097615658 | 0.591712108 | 1.053751659 | 0.870254235 | 1.275940424 |
| ebi-a-GCST90017072 | genus Sutterella id.2896 | finngen_R9_L387_PSORIASIS | Weighted mode | 12 | 0.155128187 | 0.717582937 | 1.059263762 | 0.781549225 | 1.43566097 |
| ebi-a-GCST90017075 | genus Tyzzerella3 id.11335 | finngen_R9_L388_PSORIASIS | Inverse variance weighted | 13 | 0.045446935 | 0.940513706 | 1.003397199 | 0.917883732 | 1.09687742 |
| ebi-a-GCST90017075 | genus Tyzzerella3 id.11335 | finngen_R9_L389_PSORIASIS | MR Egger | 13 | 0.257062547 | 0.88124536 | 0.961457409 | 0.580916876 | 1.591278181 |
| ebi-a-GCST90017075 | genus Tyzzerella3 id.11335 | finngen_R9_L390_PSORIASIS | Simple mode | 13 | 0.104489646 | 0.785357539 | 0.97131735 | 0.791439578 | 1.192077603 |
| ebi-a-GCST90017075 | genus Tyzzerella3 id.11335 | finngen_R9_L391_PSORIASIS | Weighted median | 13 | 0.060031052 | 0.722912425 | 0.978939774 | 0.870275006 | 1.101172703 |
| ebi-a-GCST90017075 | genus Tyzzerella3 id.11335 | finngen_R9_L392_PSORIASIS | Weighted mode | 13 | 0.097295271 | 0.902336815 | 0.987879945 | 0.816365724 | 1.195428418 |
| ebi-a-GCST90017078 | unknown genus id.1000005472 | finngen_R9_L393_PSORIASIS | Inverse variance weighted | 14 | 0.075953812 | 0.921770519 | 0.992568801 | 0.855278254 | 1.151897432 |
| ebi-a-GCST90017078 | unknown genus id.1000005472 | finngen_R9_L394_PSORIASIS | MR Egger | 14 | 0.23068328 | 0.39899506 | 0.817308002 | 0.520024947 | 1.284539086 |
| ebi-a-GCST90017078 | unknown genus id.1000005472 | finngen_R9_L395_PSORIASIS | Simple mode | 14 | 0.17070968 | 0.294718406 | 0.829949383 | 0.59393729 | 1.159745297 |
| ebi-a-GCST90017078 | unknown genus id.1000005472 | finngen_R9_L396_PSORIASIS | Weighted median | 14 | 0.090028695 | 0.60926168 | 0.955026534 | 0.800537104 | 1.139329677 |
| ebi-a-GCST90017078 | unknown genus id.1000005472 | finngen_R9_L397_PSORIASIS | Weighted mode | 14 | 0.146021731 | 0.251407167 | 0.839220005 | 0.6303469 | 1.117305753 |
| ebi-a-GCST90017079 | unknown genus id.1000005479 | finngen_R9_L398_PSORIASIS | Inverse variance weighted | 10 | 0.062150999 | 0.464379775 | 0.955545375 | 0.845955106 | 1.079332647 |
| ebi-a-GCST90017079 | unknown genus id.1000005479 | finngen_R9_L399_PSORIASIS | MR Egger | 10 | 0.265796916 | 0.231531346 | 0.70883404 | 0.421011213 | 1.193425925 |
| ebi-a-GCST90017079 | unknown genus id.1000005479 | finngen_R9_L400_PSORIASIS | Simple mode | 10 | 0.136805759 | 0.430806141 | 0.893296784 | 0.683194493 | 1.168011675 |
| ebi-a-GCST90017079 | unknown genus id.1000005479 | finngen_R9_L401_PSORIASIS | Weighted median | 10 | 0.081566849 | 0.313086715 | 0.921012298 | 0.784936141 | 1.080678553 |
| ebi-a-GCST90017079 | unknown genus id.1000005479 | finngen_R9_L402_PSORIASIS | Weighted mode | 10 | 0.131909792 | 0.414518124 | 0.893296784 | 0.68978205 | 1.156856929 |
| ebi-a-GCST90017081 | unknown genus id.1868 | finngen_R9_L403_PSORIASIS | Inverse variance weighted | 10 | 0.074217031 | 0.651406403 | 1.034100474 | 0.894103767 | 1.196017542 |
| ebi-a-GCST90017081 | unknown genus id.1868 | finngen_R9_L404_PSORIASIS | MR Egger | 10 | 0.21641144 | 0.69132322 | 0.914744409 | 0.59853097 | 1.39801844 |
| ebi-a-GCST90017081 | unknown genus id.1868 | finngen_R9_L405_PSORIASIS | Simple mode | 10 | 0.173284111 | 0.220610359 | 1.256201976 | 0.894452041 | 1.764257146 |
| ebi-a-GCST90017081 | unknown genus id.1868 | finngen_R9_L406_PSORIASIS | Weighted median | 10 | 0.098988498 | 0.451024763 | 1.077462126 | 0.887444705 | 1.308165598 |
| ebi-a-GCST90017081 | unknown genus id.1868 | finngen_R9_L407_PSORIASIS | Weighted mode | 10 | 0.151697623 | 0.229834247 | 1.215803064 | 0.903099517 | 1.636782062 |
| ebi-a-GCST90017082 | unknown genus id.2001 | finngen_R9_L408_PSORIASIS | Inverse variance weighted | 9 | 0.081955437 | 0.567665756 | 0.954242995 | 0.812637957 | 1.120523211 |
| ebi-a-GCST90017082 | unknown genus id.2001 | finngen_R9_L409_PSORIASIS | MR Egger | 9 | 0.229146472 | 0.45225526 | 0.833294976 | 0.531796346 | 1.30572638 |
| ebi-a-GCST90017082 | unknown genus id.2001 | finngen_R9_L410_PSORIASIS | Simple mode | 9 | 0.160756666 | 0.444173976 | 1.138125441 | 0.830522293 | 1.559656531 |
| ebi-a-GCST90017082 | unknown genus id.2001 | finngen_R9_L411_PSORIASIS | Weighted median | 9 | 0.103210467 | 0.897856565 | 1.013337234 | 0.827750549 | 1.240533579 |
| ebi-a-GCST90017082 | unknown genus id.2001 | finngen_R9_L412_PSORIASIS | Weighted mode | 9 | 0.110892569 | 0.643306277 | 1.054802959 | 0.848745879 | 1.310886226 |
| ebi-a-GCST90017084 | unknown genus id.2071 | finngen_R9_L413_PSORIASIS | Inverse variance weighted | 17 | 0.061502892 | 0.586151149 | 0.967070844 | 0.857246995 | 1.090964475 |
| ebi-a-GCST90017084 | unknown genus id.2071 | finngen_R9_L414_PSORIASIS | MR Egger | 17 | 0.320803609 | 0.696350136 | 0.880179304 | 0.469350849 | 1.650610859 |
| ebi-a-GCST90017084 | unknown genus id.2071 | finngen_R9_L415_PSORIASIS | Simple mode | 17 | 0.136861705 | 0.405283485 | 1.124106491 | 0.859623779 | 1.469963296 |
| ebi-a-GCST90017084 | unknown genus id.2071 | finngen_R9_L416_PSORIASIS | Weighted median | 17 | 0.084147964 | 0.606876181 | 1.044248295 | 0.885473527 | 1.231493057 |
| ebi-a-GCST90017084 | unknown genus id.2071 | finngen_R9_L417_PSORIASIS | Weighted mode | 17 | 0.12753729 | 0.429175957 | 1.108993823 | 0.863708501 | 1.423937935 |
| ebi-a-GCST90017085 | unknown genus id.2755 | finngen_R9_L418_PSORIASIS | Inverse variance weighted | 14 | 0.052073322 | 0.358278554 | 0.953289 | 0.860793294 | 1.055723741 |
| ebi-a-GCST90017085 | unknown genus id.2755 | finngen_R9_L419_PSORIASIS | MR Egger | 14 | 0.189192399 | 0.049619693 | 1.511377388 | 1.043107575 | 2.18986197 |
| ebi-a-GCST90017085 | unknown genus id.2755 | finngen_R9_L420_PSORIASIS | Simple mode | 14 | 0.129793671 | 0.874995082 | 1.021043402 | 0.791701741 | 1.316821191 |
| ebi-a-GCST90017085 | unknown genus id.2755 | finngen_R9_L421_PSORIASIS | Weighted median | 14 | 0.07196378 | 0.747451618 | 0.97709398 | 0.848554089 | 1.125105231 |
| ebi-a-GCST90017085 | unknown genus id.2755 | finngen_R9_L422_PSORIASIS | Weighted mode | 14 | 0.115547827 | 0.684580619 | 1.04917647 | 0.83655066 | 1.315845313 |
| ebi-a-GCST90017088 | genus Veillonella id.2198 | finngen_R9_L423_PSORIASIS | Inverse variance weighted | 7 | 0.092745286 | 0.510830729 | 1.062882092 | 0.886214208 | 1.274768933 |
| ebi-a-GCST90017088 | genus Veillonella id.2198 | finngen_R9_L424_PSORIASIS | MR Egger | 7 | 2.012149766 | 0.614409726 | 0.339541488 | 0.006578341 | 17.52545516 |
| ebi-a-GCST90017088 | genus Veillonella id.2198 | finngen_R9_L425_PSORIASIS | Simple mode | 7 | 0.166287539 | 0.943834831 | 0.98786033 | 0.713097205 | 1.368492296 |
| ebi-a-GCST90017088 | genus Veillonella id.2198 | finngen_R9_L426_PSORIASIS | Weighted median | 7 | 0.123473688 | 0.776816508 | 1.035620845 | 0.81301369 | 1.319178936 |
| ebi-a-GCST90017088 | genus Veillonella id.2198 | finngen_R9_L427_PSORIASIS | Weighted mode | 7 | 0.167883182 | 0.944367549 | 0.98786033 | 0.710870507 | 1.372778898 |
| ebi-a-GCST90017089 | genus Victivallis id.2256 | finngen_R9_L428_PSORIASIS | Inverse variance weighted | 12 | 0.052946478 | 0.696871536 | 0.979586101 | 0.883026349 | 1.086704752 |
| ebi-a-GCST90017089 | genus Victivallis id.2256 | finngen_R9_L429_PSORIASIS | MR Egger | 12 | 0.366281168 | 0.739478537 | 0.88228198 | 0.43035077 | 1.808807017 |
| ebi-a-GCST90017089 | genus Victivallis id.2256 | finngen_R9_L430_PSORIASIS | Simple mode | 12 | 0.114969768 | 0.654856808 | 0.948547849 | 0.757172794 | 1.188292856 |
| ebi-a-GCST90017089 | genus Victivallis id.2256 | finngen_R9_L431_PSORIASIS | Weighted median | 12 | 0.058782388 | 0.58736667 | 0.968604778 | 0.863197211 | 1.086883975 |
| ebi-a-GCST90017089 | genus Victivallis id.2256 | finngen_R9_L432_PSORIASIS | Weighted mode | 12 | 0.106444807 | 0.704337258 | 0.959386869 | 0.778728593 | 1.181956297 |
| ebi-a-GCST90017091 | order Bacillales id.1674 | finngen_R9_L433_PSORIASIS | Inverse variance weighted | 9 | 0.042334266 | 0.405657552 | 0.965408968 | 0.888537321 | 1.048931151 |
| ebi-a-GCST90017091 | order Bacillales id.1674 | finngen_R9_L434_PSORIASIS | MR Egger | 9 | 0.17697659 | 0.776392323 | 1.053635406 | 0.744808873 | 1.490513351 |
| ebi-a-GCST90017091 | order Bacillales id.1674 | finngen_R9_L435_PSORIASIS | Simple mode | 9 | 0.083846276 | 0.92271119 | 1.008430181 | 0.855607227 | 1.188549369 |
| ebi-a-GCST90017091 | order Bacillales id.1674 | finngen_R9_L436_PSORIASIS | Weighted median | 9 | 0.055025017 | 0.844013181 | 0.989231525 | 0.888095579 | 1.101884788 |
| ebi-a-GCST90017091 | order Bacillales id.1674 | finngen_R9_L437_PSORIASIS | Weighted mode | 9 | 0.085799876 | 0.888052589 | 1.012546527 | 0.855816508 | 1.197979309 |
| ebi-a-GCST90017094 | order Burkholderiales id.2874 | finngen_R9_L438_PSORIASIS | Inverse variance weighted | 12 | 0.099805558 | 0.45389292 | 0.92797689 | 0.763099124 | 1.128478702 |
| ebi-a-GCST90017094 | order Burkholderiales id.2874 | finngen_R9_L439_PSORIASIS | MR Egger | 12 | 0.284121138 | 0.097608453 | 0.595014402 | 0.34094057 | 1.038427721 |
| ebi-a-GCST90017094 | order Burkholderiales id.2874 | finngen_R9_L440_PSORIASIS | Simple mode | 12 | 0.198461631 | 0.401130532 | 0.840861275 | 0.569889167 | 1.240675775 |
| ebi-a-GCST90017094 | order Burkholderiales id.2874 | finngen_R9_L441_PSORIASIS | Weighted median | 12 | 0.11954358 | 0.242460419 | 0.869593345 | 0.687952825 | 1.0991925 |
| ebi-a-GCST90017094 | order Burkholderiales id.2874 | finngen_R9_L442_PSORIASIS | Weighted mode | 12 | 0.196025558 | 0.443273269 | 0.855648941 | 0.582686953 | 1.256481044 |
| ebi-a-GCST90017095 | order Clostridiales id.1863 | finngen_R9_L443_PSORIASIS | Inverse variance weighted | 16 | 0.072286939 | 0.310151784 | 1.076122469 | 0.933963308 | 1.239919769 |
| ebi-a-GCST90017095 | order Clostridiales id.1863 | finngen_R9_L444_PSORIASIS | MR Egger | 16 | 0.199724198 | 0.910687312 | 0.977446246 | 0.660821575 | 1.445777801 |
| ebi-a-GCST90017095 | order Clostridiales id.1863 | finngen_R9_L445_PSORIASIS | Simple mode | 16 | 0.177059186 | 0.143803825 | 1.314029415 | 0.928729552 | 1.859177733 |
| ebi-a-GCST90017095 | order Clostridiales id.1863 | finngen_R9_L446_PSORIASIS | Weighted median | 16 | 0.101825342 | 0.143576313 | 1.160591687 | 0.950613493 | 1.416951342 |
| ebi-a-GCST90017095 | order Clostridiales id.1863 | finngen_R9_L447_PSORIASIS | Weighted mode | 16 | 0.1601507 | 0.134642397 | 1.2882182 | 0.941166375 | 1.763244178 |
| ebi-a-GCST90017098 | order Enterobacteriales id.3468 | finngen_R9_L448_PSORIASIS | Inverse variance weighted | 11 | 0.083541262 | 0.967986883 | 1.00335841 | 0.851813144 | 1.18186495 |
| ebi-a-GCST90017098 | order Enterobacteriales id.3468 | finngen_R9_L449_PSORIASIS | MR Egger | 11 | 0.38286568 | 0.49642747 | 0.76234552 | 0.359956488 | 1.614558179 |
| ebi-a-GCST90017098 | order Enterobacteriales id.3468 | finngen_R9_L450_PSORIASIS | Simple mode | 11 | 0.186023679 | 0.57136729 | 1.115011918 | 0.774342252 | 1.605558231 |
| ebi-a-GCST90017098 | order Enterobacteriales id.3468 | finngen_R9_L451_PSORIASIS | Weighted median | 11 | 0.114798669 | 0.644795069 | 1.054348196 | 0.841909596 | 1.320391316 |
| ebi-a-GCST90017098 | order Enterobacteriales id.3468 | finngen_R9_L452_PSORIASIS | Weighted mode | 11 | 0.186713585 | 0.572766524 | 1.115011918 | 0.773295881 | 1.607730763 |
| ebi-a-GCST90017100 | order Gastranaerophilales id.1591 | finngen_R9_L453_PSORIASIS | Inverse variance weighted | 12 | 0.057274009 | 0.477736844 | 0.960154063 | 0.858199591 | 1.074220768 |
| ebi-a-GCST90017100 | order Gastranaerophilales id.1591 | finngen_R9_L454_PSORIASIS | MR Egger | 12 | 0.175234396 | 0.29932035 | 1.211460094 | 0.859303505 | 1.707936194 |
| ebi-a-GCST90017100 | order Gastranaerophilales id.1591 | finngen_R9_L455_PSORIASIS | Simple mode | 12 | 0.11160961 | 0.249180621 | 0.873021405 | 0.701489015 | 1.086497947 |
| ebi-a-GCST90017100 | order Gastranaerophilales id.1591 | finngen_R9_L456_PSORIASIS | Weighted median | 12 | 0.072073316 | 0.185727218 | 0.909030672 | 0.789275264 | 1.046956366 |
| ebi-a-GCST90017100 | order Gastranaerophilales id.1591 | finngen_R9_L457_PSORIASIS | Weighted mode | 12 | 0.118537651 | 0.276278013 | 0.873021405 | 0.692027905 | 1.101352083 |
| ebi-a-GCST90017103 | order Mollicutes RF9 id.11579 | finngen_R9_L458_PSORIASIS | Inverse variance weighted | 14 | 0.075953812 | 0.921770519 | 0.992568801 | 0.855278254 | 1.151897432 |
| ebi-a-GCST90017103 | order Mollicutes RF9 id.11579 | finngen_R9_L459_PSORIASIS | MR Egger | 14 | 0.23068328 | 0.39899506 | 0.817308002 | 0.520024947 | 1.284539086 |
| ebi-a-GCST90017103 | order Mollicutes RF9 id.11579 | finngen_R9_L460_PSORIASIS | Simple mode | 14 | 0.15500026 | 0.250608597 | 0.829949383 | 0.612509348 | 1.124580352 |
| ebi-a-GCST90017103 | order Mollicutes RF9 id.11579 | finngen_R9_L461_PSORIASIS | Weighted median | 14 | 0.090110493 | 0.609586587 | 0.955026534 | 0.800408769 | 1.139512354 |
| ebi-a-GCST90017103 | order Mollicutes RF9 id.11579 | finngen_R9_L462_PSORIASIS | Weighted mode | 14 | 0.14809392 | 0.257767546 | 0.839220005 | 0.627791944 | 1.121852907 |
| ebi-a-GCST90017104 | order NB1n id.3953 | finngen_R9_L463_PSORIASIS | Inverse variance weighted | 15 | 0.048432424 | 0.7827043 | 1.013447107 | 0.921668142 | 1.114365347 |
| ebi-a-GCST90017104 | order NB1n id.3953 | finngen_R9_L464_PSORIASIS | MR Egger | 15 | 0.221256333 | 0.781630552 | 1.064618228 | 0.690012097 | 1.642597249 |
| ebi-a-GCST90017104 | order NB1n id.3953 | finngen_R9_L465_PSORIASIS | Simple mode | 15 | 0.079190608 | 0.98936178 | 1.001075472 | 0.857153127 | 1.169163442 |
| ebi-a-GCST90017104 | order NB1n id.3953 | finngen_R9_L466_PSORIASIS | Weighted median | 15 | 0.055616673 | 0.977977735 | 0.998465917 | 0.895346988 | 1.113461263 |
| ebi-a-GCST90017104 | order NB1n id.3953 | finngen_R9_L467_PSORIASIS | Weighted mode | 15 | 0.077659967 | 0.98944571 | 0.998954746 | 0.857907206 | 1.16319175 |
| ebi-a-GCST90017106 | order Rhodospirillales id.2667 | finngen_R9_L468_PSORIASIS | Inverse variance weighted | 14 | 0.0534074 | 0.592269623 | 0.971802844 | 0.875219266 | 1.079044765 |
| ebi-a-GCST90017106 | order Rhodospirillales id.2667 | finngen_R9_L469_PSORIASIS | MR Egger | 14 | 0.20693859 | 0.147673429 | 1.377485649 | 0.918200234 | 2.066506457 |
| ebi-a-GCST90017106 | order Rhodospirillales id.2667 | finngen_R9_L470_PSORIASIS | Simple mode | 14 | 0.11964137 | 0.749824715 | 0.961780759 | 0.760738322 | 1.215953241 |
| ebi-a-GCST90017106 | order Rhodospirillales id.2667 | finngen_R9_L471_PSORIASIS | Weighted median | 14 | 0.072696823 | 0.666475906 | 0.96915417 | 0.840450391 | 1.117567218 |
| ebi-a-GCST90017106 | order Rhodospirillales id.2667 | finngen_R9_L472_PSORIASIS | Weighted mode | 14 | 0.115325019 | 0.976560447 | 0.996551768 | 0.794937941 | 1.249299316 |
| ebi-a-GCST90017107 | order Selenomonadales id.2165 | finngen_R9_L473_PSORIASIS | Inverse variance weighted | 13 | 0.09998499 | 0.756839063 | 0.969515533 | 0.796977076 | 1.179407033 |
| ebi-a-GCST90017107 | order Selenomonadales id.2165 | finngen_R9_L474_PSORIASIS | MR Egger | 13 | 0.36475216 | 0.688954559 | 0.860777855 | 0.421121854 | 1.75943972 |
| ebi-a-GCST90017107 | order Selenomonadales id.2165 | finngen_R9_L475_PSORIASIS | Simple mode | 13 | 0.230731007 | 0.173651192 | 0.716232981 | 0.455671763 | 1.125787737 |
| ebi-a-GCST90017107 | order Selenomonadales id.2165 | finngen_R9_L476_PSORIASIS | Weighted median | 13 | 0.124833891 | 0.696175668 | 1.049953782 | 0.822071192 | 1.341006662 |
| ebi-a-GCST90017107 | order Selenomonadales id.2165 | finngen_R9_L477_PSORIASIS | Weighted mode | 13 | 0.244259559 | 0.532563043 | 1.169953152 | 0.724853997 | 1.888367016 |
| ebi-a-GCST90017110 | phylum Actinobacteria id.400 | finngen_R9_L478_PSORIASIS | Inverse variance weighted | 15 | 0.076529438 | 0.464929391 | 1.057517518 | 0.910215858 | 1.228657235 |
| ebi-a-GCST90017110 | phylum Actinobacteria id.400 | finngen_R9_L479_PSORIASIS | MR Egger | 15 | 0.335529111 | 0.476172894 | 1.279114685 | 0.662676149 | 2.468980331 |
| ebi-a-GCST90017110 | phylum Actinobacteria id.400 | finngen_R9_L480_PSORIASIS | Simple mode | 15 | 0.175487977 | 0.487696697 | 1.133242738 | 0.80342356 | 1.598458355 |
| ebi-a-GCST90017110 | phylum Actinobacteria id.400 | finngen_R9_L481_PSORIASIS | Weighted median | 15 | 0.103941619 | 0.287636138 | 1.116862447 | 0.91100927 | 1.369230551 |
| ebi-a-GCST90017110 | phylum Actinobacteria id.400 | finngen_R9_L482_PSORIASIS | Weighted mode | 15 | 0.158353555 | 0.249671616 | 1.209473136 | 0.886753599 | 1.649641196 |
| ebi-a-GCST90017113 | phylum Euryarchaeota id.55 | finngen_R9_L483_PSORIASIS | Inverse variance weighted | 11 | 0.041843436 | 0.22383994 | 0.950376034 | 0.87554329 | 1.031604738 |
| ebi-a-GCST90017113 | phylum Euryarchaeota id.55 | finngen_R9_L484_PSORIASIS | MR Egger | 11 | 0.185393258 | 0.543167337 | 0.889451615 | 0.618461118 | 1.279181752 |
| ebi-a-GCST90017113 | phylum Euryarchaeota id.55 | finngen_R9_L485_PSORIASIS | Simple mode | 11 | 0.092496382 | 0.997010791 | 1.000355352 | 0.834487412 | 1.199192241 |
| ebi-a-GCST90017113 | phylum Euryarchaeota id.55 | finngen_R9_L486_PSORIASIS | Weighted median | 11 | 0.05313892 | 0.581485325 | 0.971133779 | 0.875077059 | 1.077734592 |
| ebi-a-GCST90017113 | phylum Euryarchaeota id.55 | finngen_R9_L487_PSORIASIS | Weighted mode | 11 | 0.085251426 | 0.912368951 | 1.00966859 | 0.854301891 | 1.193290888 |
| ebi-a-GCST90017116 | phylum Proteobacteria id.2375 | finngen_R9_L488_PSORIASIS | Inverse variance weighted | 14 | 0.079028674 | 0.47872132 | 1.057577382 | 0.905819339 | 1.234760476 |
| ebi-a-GCST90017116 | phylum Proteobacteria id.2375 | finngen_R9_L489_PSORIASIS | MR Egger | 14 | 0.218895843 | 0.253024752 | 0.768870249 | 0.500639548 | 1.180812549 |
| ebi-a-GCST90017116 | phylum Proteobacteria id.2375 | finngen_R9_L490_PSORIASIS | Simple mode | 14 | 0.194432842 | 0.376687435 | 1.194788525 | 0.816180974 | 1.749023397 |
| ebi-a-GCST90017116 | phylum Proteobacteria id.2375 | finngen_R9_L491_PSORIASIS | Weighted median | 14 | 0.110626964 | 0.404920481 | 1.096515476 | 0.882769242 | 1.362016406 |
| ebi-a-GCST90017116 | phylum Proteobacteria id.2375 | finngen_R9_L492_PSORIASIS | Weighted mode | 14 | 0.188727998 | 0.657949799 | 1.089274465 | 0.752469338 | 1.576833501 |
| ebi-a-GCST90017117 | phylum Tenericutes id.3919 | finngen_R9_L493_PSORIASIS | Inverse variance weighted | 12 | 0.079351762 | 0.362973592 | 0.930356555 | 0.796349733 | 1.086913556 |
| ebi-a-GCST90017117 | phylum Tenericutes id.3919 | finngen_R9_L494_PSORIASIS | MR Egger | 12 | 0.281337289 | 0.593133979 | 0.856183393 | 0.493273364 | 1.486092816 |
| ebi-a-GCST90017117 | phylum Tenericutes id.3919 | finngen_R9_L495_PSORIASIS | Simple mode | 12 | 0.139780915 | 0.252073648 | 0.844533952 | 0.642145136 | 1.110710892 |
| ebi-a-GCST90017117 | phylum Tenericutes id.3919 | finngen_R9_L496_PSORIASIS | Weighted median | 12 | 0.097423435 | 0.179695478 | 0.87747013 | 0.724942965 | 1.062088835 |
| ebi-a-GCST90017117 | phylum Tenericutes id.3919 | finngen_R9_L497_PSORIASIS | Weighted mode | 12 | 0.113168533 | 0.130167935 | 0.831013628 | 0.665697837 | 1.037383046 |

| Supplementary Table 4: Correlation disease or trait of SNPs in significant IVW results retrievaled by PhenoScanner | | | |
| --- | --- | --- | --- |
| snp | a1 | a2 | trait |
| rs111810795 | C | T | Mean corpuscular hemoglobin |
| rs12668619 | A | G | Total cholesterol |
| rs12668619 | A | G | Total cholesterol |
| rs12668619 | A | G | Total cholesterol |
| rs1442060 | A | G | Treatment with glyceryl trinitrate |
| rs4461038 | G | A | Hematocrit |
| rs4461038 | G | A | Hemoglobin concentration |
| rs4461038 | G | A | Mean corpuscular hemoglobin concentration |
| rs4461038 | G | A | Platelet count |
| rs4461038 | G | A | Plateletcrit |
| rs4461038 | G | A | Red blood cell count |
| rs4461038 | G | A | Red cell distribution width |
| rs4461038 | G | A | Reticulocyte count |
| rs4461038 | G | A | Reticulocyte fraction of red cells |
| rs79535861 | A | C | Self-reported malignant melanoma |
| rs11153159 | G | C | Mean corpuscular hemoglobin |
| rs11153159 | G | C | Mean corpuscular volume |
| rs11153159 | G | C | Red blood cell count |
| rs11153159 | G | C | Birth weight |
| rs11153159 | G | C | Self-reported hypothyroidism or myxoedema |
| rs4294381 | C | T | White blood cell count |
| rs6489992 | A | G | Diastolic blood pressure |
| rs6489992 | A | G | Diastolic blood pressure |
